# Linking gas, particulate, and toxic endpoints to air emissions in the Community Regional Atmospheric Chemistry Multiphase Mechanism (CRACMM)

**DOI:** 10.5194/acp-23-5043-2023

**Published:** 2023-05-04

**Authors:** Havala O. T. Pye, Bryan K. Place, Benjamin N. Murphy, Karl M. Seltzer, Emma L. D’Ambro, Christine Allen, Ivan R. Piletic, Sara Farrell, Rebecca H. Schwantes, Matthew M. Coggon, Emily Saunders, Lu Xu, Golam Sarwar, William T. Hutzell, Kristen M. Foley, George Pouliot, Jesse Bash, William R. Stockwell

**Affiliations:** 1 Office of Research and Development, U.S. Environmental Protection Agency, Research Triangle Park, North Carolina, USA; 2 Oak Ridge Institute for Science and Engineering (ORISE), Office of Research and Development, U.S. Environmental Protection Agency, Research Triangle Park, North Carolina, USA; 3 Office of Air and Radiation, U.S. Environmental Protection Agency, Research Triangle Park, North Carolina, USA; 4 General Dynamics Information Technology, Research Triangle Park, North Carolina, USA; 5 Chemical Sciences Laboratory, National Oceanic and Atmospheric Administration, Boulder, Colorado, USA; 6 Cooperative Institute for Research in Environmental Science (CIRES), University of Colorado Boulder, Boulder, Colorado, USA; 7 Office of Chemical Safety and Pollution Prevention, U.S. Environmental Protection Agency, Washington, DC, USA; 8 Department of Physics, University of Texas at El Paso, El Paso, Texas, USA

## Abstract

Chemical mechanisms describe the atmospheric transformations of organic and inorganic species and connect air emissions to secondary species such as ozone, fine particles, and hazardous air pollutants (HAPs) like formaldehyde. Recent advances in our understanding of several chemical systems and shifts in the drivers of atmospheric chemistry warrant updates to mechanisms used in chemical transport models such as the Community Multiscale Air Quality (CMAQ) modeling system. This work builds on the Regional Atmospheric Chemistry Mechanism version 2 (RACM2) and develops the Community Regional Atmospheric Chemistry Multiphase Mechanism (CRACMM) version 1.0, which demonstrates a fully coupled representation of chemistry leading to ozone and secondary organic aerosol (SOA) with consideration of HAPs. CRACMMv1.0 includes 178 gas-phase species, 51 particulate species, and 508 reactions spanning gas-phase and heterogeneous pathways. To support estimation of health risks associated with HAPs, nine species in CRACMM cover 50 % of the total cancer and 60 % of the total non-cancer emission-weighted toxicity estimated for primary HAPs from anthropogenic and biomass burning sources in the US, with the coverage of toxicity higher (>80 %) when secondary formaldehyde and acrolein are considered. In addition, new mechanism species were added based on the importance of their emissions for the ozone, organic aerosol, or atmospheric burden of total reactive organic carbon (ROC): sesquiterpenes, furans, propylene glycol, alkane-like low- to intermediate-volatility organic compounds (9 species), low- to intermediate-volatility oxygenated species (16 species), intermediate-volatility aromatic hydrocarbons (2 species), and slowly reacting organic carbon. Intermediate- and lower-volatility organic compounds were estimated to increase the coverage of anthropogenic and biomass burning ROC emissions by 40 % compared to current operational mechanisms. Autoxidation, a gas-phase reaction particularly effective in producing SOA, was added for C_10_ and larger alkanes, aromatic hydrocarbons, sesquiterpenes, and monoterpene systems including second-generation aldehydes. Integrating the radical and SOA chemistry put additional constraints on both systems and enabled the implementation of previously unconsidered SOA pathways from phenolic and furanone compounds, which were predicted to account for ~ 30 % of total aromatic hydrocarbon SOA under typical atmospheric conditions. CRACMM organic aerosol species were found to span the atmospherically relevant range of species carbon number, number of oxygens per carbon, and oxidation state with a slight high bias in the number of hydrogens per carbon. In total, 11 new emitted species were implemented as precursors to SOA compared to current CMAQv5.3.3 representations, resulting in a bottom-up prediction of SOA, which is required for accurate source attribution and the design of control strategies. CRACMMv1.0 is available in CMAQv5.4.

## 1 Introduction

Reactive organic carbon (ROC) (Safieddine et al., 2017) includes all atmospheric organic species excluding methane and is abundant throughout the troposphere. Particulate forms of ROC are found in fine particles (PM_2.5_), and gaseous ROC is a major precursor to ozone (O_3_) and secondary organic aerosol (SOA) (Heald and Kroll, 2020). Recent work indicates that preferentially controlling emissions of ROC could yield significant health benefits by mitigating the mortality associated with ambient air pollution in the US (Pye et al., 2022). These predicted benefits come primarily from reductions in SOA, which is strongly associated with cardiorespiratory mortality (Pye et al., 2021; Pond et al., 2022). ROC also includes hazardous air pollutants (HAPs) such as benzene and formaldehyde that result in cancer and non-cancer risks to health (Scheffe et al., 2016).

Atmospheric chemical mechanisms connect ROC emissions to endpoints like SOA, O_3_, and secondary HAPs and are used to inform air quality management strategies to mitigate the impacts of air pollution. Chemical mechanisms were traditionally designed for estimating ambient O_3_ although not necessarily the lower levels of O_3_ observed today (Kaduwela et al., 2015) or sources of growing importance around the globe such as volatile chemical products (VCPs, also referred to as solvents) (Coggon et al., 2021; Karl et al., 2018; McDonald et al., 2018; Zheng et al., 2018) and biomass burning (Jaffe and Wigder, 2012) that are changing the composition of emissions towards increasingly oxygenated ROC (Venecek et al., 2018). While mechanisms may predict O_3_ reasonably well on broad spatial and temporal scales (Simon et al., 2012; Xing et al., 2015; Young et al., 2018), regional biases in predicted O_3_ can exceed 10 ppb (Young et al., 2018; Solazzo et al., 2017) or 20 % (Appel et al., 2012, 2021). Global model estimates of chemical production and loss of ozone also vary by a factor of ~ 2 (Young et al., 2018), and emerging chemical pathways missing from standard models, such as particulate nitrate photolysis, can increase free-tropospheric ozone by 5 ppb (Shah et al., 2023), indicating a continued need for model development for ozone prediction. Furthermore, even when mechanisms are relatively similar in their O_3_ predictions, they can differ substantially in terms of predicted intermediates like the hydroxyl radical (HO) and nitrate radical (NO_3_) as well as products like formaldehyde and SOA (Knote et al., 2015). Model representations of organic aerosol are particularly diverse and span a factor of 10 in their estimates of global SOA source strength (Tsigaridis et al., 2014). Given parts of 22 different states are in marginal attainment to extreme non-attainment for the current US 8 h (2012) O_3_ standard (as of August 2022) (U.S. Environmental Protection Agency, 2022d) as well as recent work demonstrating health effects below the current fine-particle standards (Makar et al., 2017), increasingly accurate representations of emissions and how they connect to chemistry will be needed to inform air quality management strategies going forward. In addition, future implementation of global air quality guidelines, such as those from the World Health Organization, may need to account for the speciation of ambient aerosol since different species have different anthropogenic contributions (Pai et al., 2022).

In most chemical transport models used for air quality prediction, SOA algorithms are disconnected from the gas-phase radical chemistry leading to O_3_ formation (Pye et al., 2010; Ahmadov et al., 2012; Koo et al., 2014; Tilmes et al., 2015), leading to duplication of mass in the O_3_ and SOA representations. Gas-phase chemical mechanisms also typically exclude non-traditional species with saturation concentrations $\left(C_{i}^{*}\right)$ in the low-volatility organic compound (LVOC; $10^{-2.5}\leq C_{i}^{*}<10^{-0.5}\;\mu \text{g}\;{\text{m}}^{-3}$) and semivolatile organic compound (SVOC; $10^{-0.5}\leq C_{i}^{*}<10^{2.5}\;\mu \text{g}\;{\text{m}}^{-3}$) range. In addition, some gas-phase mechanisms also exclude intermediate-volatility organic compounds (IVOCs; $10^{2.5}\leq C_{i}^{*}<10^{6.5}\;\mu \text{g}\;{\text{m}}^{-3}$) (Shah et al., 2020), which are potent SOA precursors but are somewhat less important for O_3_ formation than volatile organic compounds (VOCs; $C_{i}^{*}\geq 10^{6.5}\;\mu \text{g}\;{\text{m}}^{-3}$). Recent studies have noted that the magnitude of VCP emissions exerts significant impact on model-predicted O_3_ but predicted SOA mass is relatively insensitive to VCP emissions due to a lack of suitable SOA precursors in standard mechanisms (Qin et al., 2021; Pennington et al., 2021; Zhu et al., 2019). This conclusion is consistent with the ROC budget analysis for Pasadena, California, by Heald et al. (2020) that suggests SOA formation requires consideration of precursors beyond traditional, non-oxygenated volatile hydrocarbons represented in most current SOA treatments.

Due to the challenges in representing SOA chemistry in mechanisms, some chemical transport models have opted to use empirical representations of anthropogenic SOA. These parameterizations are not tied to the behavior of specific parent hydrocarbon compounds or emission sources and fall into two classes: multigenerational and simplified. Multigenerational anthropogenic SOA treatments (Robinson et al., 2007) generally leverage the volatility basis set (VBS) framework and add IVOC and SVOC emissions thought to be missed by current measurement techniques (Koo et al., 2014; Ahmadov et al., 2012). Species throughout the $C_{i}^{*}<10^{6.5}\;\mu \text{g}\;{\text{m}}^{-3}$ volatility range are chemically processed over multiple HO reactions, leading to the production of lower-volatility species and SOA mass. Simplified representations use CO (Hodzic and Jimenez, 2011; Kim et al., 2015), primary organic aerosol (Murphy et al., 2017), or C_4_H_10_ (Dunne et al., 2020) as a surrogate for anthropogenic activity and precursor emissions that oxidize in one step to SOA. Since the SOA predicted from traditional anthropogenic hydrocarbon precursors has typically been small compared to observed SOA in urban locations (Woody et al., 2016), these schemes can be implemented in parallel to, or as a replacement for, explicit SOA precursor schemes based on traditional VOC precursors. The simplified surrogate approaches are fit to ambient data and thus have the advantage of reproducing observed levels of SOA (Qin et al., 2021; Nault et al., 2018; Murphy et al., 2017). For applications like the calculation of present-day aerosol optical depth or PM_2.5_ mass (e.g., Pye et al., 2021), empirical representations of anthropogenic SOA may be sufficient. However, the policy applications of empirical approaches are limited because they add emissions external to the regulatory reporting and model platform framework, do not allow for the separation of individual anthropogenic source contributions, and do not consider the representativeness of the emitted proxy in the context of a changing emission or chemical regime, all of which are needed for the design of regulatory control strategies.

In this work, the first version of the Community Regional Atmospheric Chemistry Multiphase Mechanism (CRACMM) is developed and presented. CRACMMv1.0 builds off the history of the Regional Atmospheric Chemistry Mechanism (RACM) development (Stockwell et al., 1997). RACM version 2 (Goliff et al., 2013) was chosen as a framework since it is implemented in regional models such as the Community Multiscale Air Quality (CMAQ) modeling system (Sarwar et al., 2013), provides a competitive computational speed with mechanisms used in regulatory applications (Sarwar et al., 2013), retains the carbon backbone of emitted species, represents individual peroxy radicals, and relies minimally on aggregated species for radical cycling (operators). Because of these features, RACM2 facilitates comparison with observations, provides transparency in emission mapping, and is relatively easy to modify and expand.

The purpose of the CRACMM version 1.0 effort described here is to demonstrate a coupled representation of ${\text{NO}}_{x}-\text{ROC}-{\text{O}}_{3}$ chemistry including SOA and the consideration of HAPs. In addition, this work includes the development of rules for mapping emitted ROC to mechanism species and updates to rate constants leading to a publicly available mechanism upon which further developments can be built. CRACMM is expected to become the default option in CMAQ in the future (U.S. Environmental Protection Agency, 2021c). While the mechanism is presented in the context of US conditions, it is informed by conditions outside the US (e.g., the work of Zhao et al., 2016, for China) and is meant to be generally relevant for tropospheric chemistry. CRACMM is available in the public release of CMAQv5.4 (U.S. EPA Office of Research and Development, 2022) and is distributed as a stand-alone mechanism (U.S. Environmental Protection Agency, 2022b). In this work, the aggregation of individual organic species to mechanism species (Sect. 2) and the chemistry (Sect. 3) and representation of HAPs (Sect. 4) are described for atmospheric ROC. The paper continues with a characterization of ROC in terms of oxidation state and van Krevelen space as well as estimated implications for O_3_ and fine-particle mass (Sect. 5). The paper concludes with a discussion on the importance of mechanism development with recommendations for future work (Sect. 6).

## 2 ROC emissions

Various aspects of the development of CRACMM are related to the identity of ROC emissions. The methods behind characterizing emitted ROC and how it maps to mechanism species are described in the following section.

### 2.1 Individual emitted species

To inform the aggregation of individual species to mechanism species as well as estimate the contributions of mechanism species to endpoints like O_3_ and SOA, an emission inventory of individual ROC species was created for 2017 US conditions. Total ROC emissions from wildland fires, oil and gas extraction, vehicles, volatile chemical products, residential wood combustion, and other non-biogenic sectors were obtained following the Environmental Protection Agency’s (EPA) Air QUAlity TimE Series (EQUATES) methods (Foley et al., 2023) based on the US National Emissions Inventory (NEI). The HAPs naphthalene, benzene, acetaldehyde, formaldehyde, and methanol (NBAFM) were included as specific species when available in the NEI. In the case of mobile emissions estimated with the MOVES model (MOtor Vehicle Emission Simulator; U.S. Environmental Protection Agency, 2020) and solvents estimated with the volatile chemical products in Python (VCPy) model (Seltzer et al., 2021), total ROC and individual HAPs (e.g., ethyl benzene, acrolein, styrene, and others in addition to NBAFM) were estimated consistently. For the remaining sectors, HAP species were estimated as a fraction of total ROC based on speciation profiles for different sources. In addition to the base EQUATES emissions, L/S/IVOC (LVOC, SVOC, and/or IVOC) emissions missing from the mobile-sector inventoried ROC mass, estimated at 4.6 % of non-methane organic gas (NMOG) for gasoline vehicles and 55 % of NMOG from diesel vehicles, were added using the volatility distribution from the work of Lu et al. (2020). An additional 20 % of NMOG from wood-burning sources (wildland, prescribed, and residential) was estimated to be an IVOC (assigned a $C_{i}^{*}$ of $10^{4}\;\mu \text{g}\;{\text{m}}^{-3}$) following the estimates of Jathar et al. (2014). L/S/IVOC emissions inventoried as part of primary PM_2.5_ were estimated using published volatility profiles for vehicles (Lu et al., 2020) and wood burning (May et al., 2013; Woody et al., 2016). Other sources of primary organic aerosol (POA) were assumed to behave as a species with a $C_{i}^{*}$ of $10^{-2}\;\mu \text{g}\;{\text{m}}^{-3}$.

The identity of the individual species within inventoried ROC as well as the L/S/IVOCs (Jathar et al., 2014; Lu et al., 2020) were characterized using the EPA SPECIATE database version 5.2 (Simon et al., 2010) (pre-release version; see “Code and data availability”). To provide chemical structure information and facilitate automated property estimation, compounds in the SPECIATE database were assigned a unique Distributed Structure-Searchable Toxicity Database Substance Identifier (DTXSID) (Grulke et al., 2019) using the U.S. EPA’s Chemicals Dashboard (referred to as the Dashboard; U.S. Environmental Protection Agency, 2021d; Williams et al., 2017). DTXSIDs allowed for each emitted species to be associated with structural identifiers like Simplified Molecular Input Line Entry System (SMILES) and IUPAC (International Union of Pure and Applied Chemistry) International Chemical Identifier (InChI) representations. In about two-thirds of cases, the emitted SPECIATE species could be exactly matched to a representative compound with a DTXSID in the Dashboard. In the other cases, an isomer or generally representative compound with similar functionality (e.g., presence of aromaticity or other functional groups) and carbon number (e.g., undecane for “isomers of undecane”) was manually selected. For the small number of cases in which the SPECIATE species was indicated as “unknown,” “unidentified”, or similarly undefined, n-decane was assigned as the representative compound. If the unidentified compound was also indicated as exempt from the regulatory definition of VOC (Code of Federal Regulations, 1986) (e.g., “aggregated exempt compounds”, “other, lumped, exempts, individually < 2 % of category”), acetone was used as the representative compound. The representative compound’s preferred name from the Dashboard, DTXSID identifier, and a degree of assignment confidence score (1: species not well defined, 2: species manually mapped, 3: species automatically matched in the Dashboard but some properties inconsistent, 4: exact match in the Dashboard) were added to SPECIATEv5.2 (U.S. Environmental Protection Agency, 2022e). A logical (true/false) field in the SPECIATE database was also used to identify individual compounds classified as HAPs (see Sect. 4).

By mapping each emitted species (i) to a unique structural identifier, properties of the emissions could be estimated in a traceable manner. The batch feature of the Dashboard (Lowe and Williams, 2021) was used to obtain molecular weights, SMILES strings, and molecular formulas as well as perform OPEn structure–activity/property Relationship App (OPERA) (Mansouri et al., 2018) calculations for the Henry’s Law coefficient, rate constant for atmospheric reaction with $\text{HO}\left(k_{\text{OH}}\right)$, and vapor pressure of each ROC species. Vapor pressures $\left(P_{i}^{\text{vap}}\right)$ and molecular weights $\left(M_{i}\right)$ were used to calculate pure-species saturation concentrations (Donahue et al., 2006) at a temperature (T) of 298 K $\left(C_{i}^{*}=P_{i}^{\text{vap}}M_{i}/(RT)\right.$, where R is the gas constant and $C_{i}^{*}$ is reported in $\mu \text{g}\;{\text{m}}^{-3}$).

While actual mechanism calculations are required to estimate the contribution of any species to O_3_ and SOA in a specific location, two simple structure–activity relationships (SARs) were created for screening-level analysis of organic aerosol (OA) and O_3_ formation potentials of individual ROC species. In the case of OA potential, several sources, largely following high-${\text{NO}}_{x}$ conditions outlined in the work of Seltzer et al. (2021), were aggregated to estimate the SOA yield of individual species. In this work, exponential or quadratic polynomial fits depending on what was most applicable were applied to data on the yield of SOA vs. ${\text{log}}_{10}\left(C_{i}^{*}\right)$ by chemical class for oxygenated hydrocarbons, polycyclic aromatic hydrocarbons (PAHs), substituted aromatics, and alkenes and to the yield of SOA vs. the number of carbons for normal, branched, and cyclic alkanes. Most systems showed a good correlation between predicted and expected SOA yield with a coefficient of determination $\left(r^{2}\right)$ of 0.67 in the case of oxygenated hydrocarbons and greater for the other species types. Explicit yield assignments were made based on published data in the case of sesquiterpenes, monoterpenes, benzene, toluene, and xylene (Pye et al., 2010; Ng et al., 2007). Published single-ring aromatic yields were scaled up by the vapor wall loss factor (Zhang et al., 2014). An OA concentration of $10\;\mu \text{g}\;{\text{m}}^{-3}$ and equal low-${\text{NO}}_{x}$ vs. high-${\text{NO}}_{x}$ behavior, typical of Northern Hemisphere July conditions (Porter et al., 2021), were assumed for these explicit yield assignments. While this OA concentration is on the high end of the atmospherically relevant range, it is on the low end of concentrations probed in laboratory studies (Porter et al., 2021), thus providing a bridge between observations and ambient conditions.

A second simple SAR was created to estimate the role of individual ROC species in O_3_ formation as indicated by maximum incremental reactivity (MIR). Input data for regression fits were obtained from the SAPRC database (Carter, 2019), which contains MIR data for over 1000 compounds. In the case of ill-defined compounds in the SAPRC database, representative compound structures with DTXSIDs were assigned. Compounds were filtered into various chemical classes (halocarbons, oxygenated, aromatic, alkenes, etc.). Within a given class, the MIR was fit as a function of the number of carbons per molecule, HO rate constant (from OPERA), number of oxygens, number of double bonds, number of ring structures, number of double bonded oxygen, and/or number of branches depending on the chemical class. The overall $r^{2}$ between SAPRC-estimated and simple-SAR-predicted MIRs (Fig. S8) was 0.72. The MIRs are most appropriate for comparing species under a given set of conditions as changes in chemical (or meteorological) regime, such as those in the US between 1988 and 2010, have been found to decrease species MIRs by about 20 % on average (Venecek et al., 2018). The SARs were used to estimate average SOA yields and MIR for all ROC species in the SPECIATE database.

### 2.2 Mechanism species

CRACMM species were designed to leverage the original RACM2 chemistry while also considering the properties of present-day emitted species, including properties indicative of SOA formation potential, with a goal of maintaining a reasonable mechanism size (by species count) for computational efficiency. New explicit species were added for multiple reasons. First, certain species are known to contribute significantly to cancer and non-cancer health risk (Scheffe et al., 2016). Second, recent advances in measurement techniques, particularly for VOCs, have increased the number of measured species available, which motivates adding these newly measured species explicitly into models for direct comparison. Third, some individual species are emitted in significant quantities, and explicit representation facilitates better conservation of mass and the representation of product distributions. New lumped species were also added when existing RACM2 species did not provide a good fit in terms of molecular properties, SOA yields, or O_3_ formation potential for emissions.

A Python mapper (see “Code and data availability”) was developed to automate mapping of individual, emitted ROC species to mechanism species. Once initial rules were created with the intent of following RACM2, properties of the mechanism species were visualized and mapping rules were manually adjusted to better preserve mass (minimize the spread in the number of carbons per molecule, molecular weight, and molar oxygen–carbon ratio within the model species), estimate SOA (minimize spread in the saturation concentration, SOA yield, and Henry’s law coefficient within the model species), and predict O_3_ (minimize spread in the HO rate constant and O_3_ formation potential within each model species). A decision tree summarizing the final mapper is provided schematically in Supplement Figs. S1–S4. The mapper uses as input the SMILES string for the ROC species, HO rate constant, and pure component $C_{i}^{*}$. Both $k_{\text{OH}}$ and $C_{i}^{*}$ can be estimated from a SMILES string prior to mapper input using OPERA algorithms (Mansouri et al., 2018) available for any organic species through the EPA Chemical Transformation Simulator (U.S. Environmental Protection Agency, 2022f). This emission mapping follows a hierarchy of rules in which explicit species are mapped first followed by lumped biogenic VOCs (α-pinene and other monoterpenes with one double bond, API; limonene and other monoterpenes with two or more double bonds, LIM; and sesquiterpenes, SESQ). Other lumped species and mapping rules were created to consider volatility, functional groups (parsed in Python using the work of RDKit, 2022), and $k_{\text{OH}}$. For L/SVOCs, mechanism assignment was based purely on volatility except in the case of PAHs (more than one aromatic ring), which were grouped with naphthalene into a NAPH species (Sect. 3.5). For IVOCs, assignments considered volatility and the presence of specific functional groups (aromatic, oxygenated, alkane). For VOCs, mapping considered only functional groups and $k_{\text{OH}}$.

**Figure 1.**
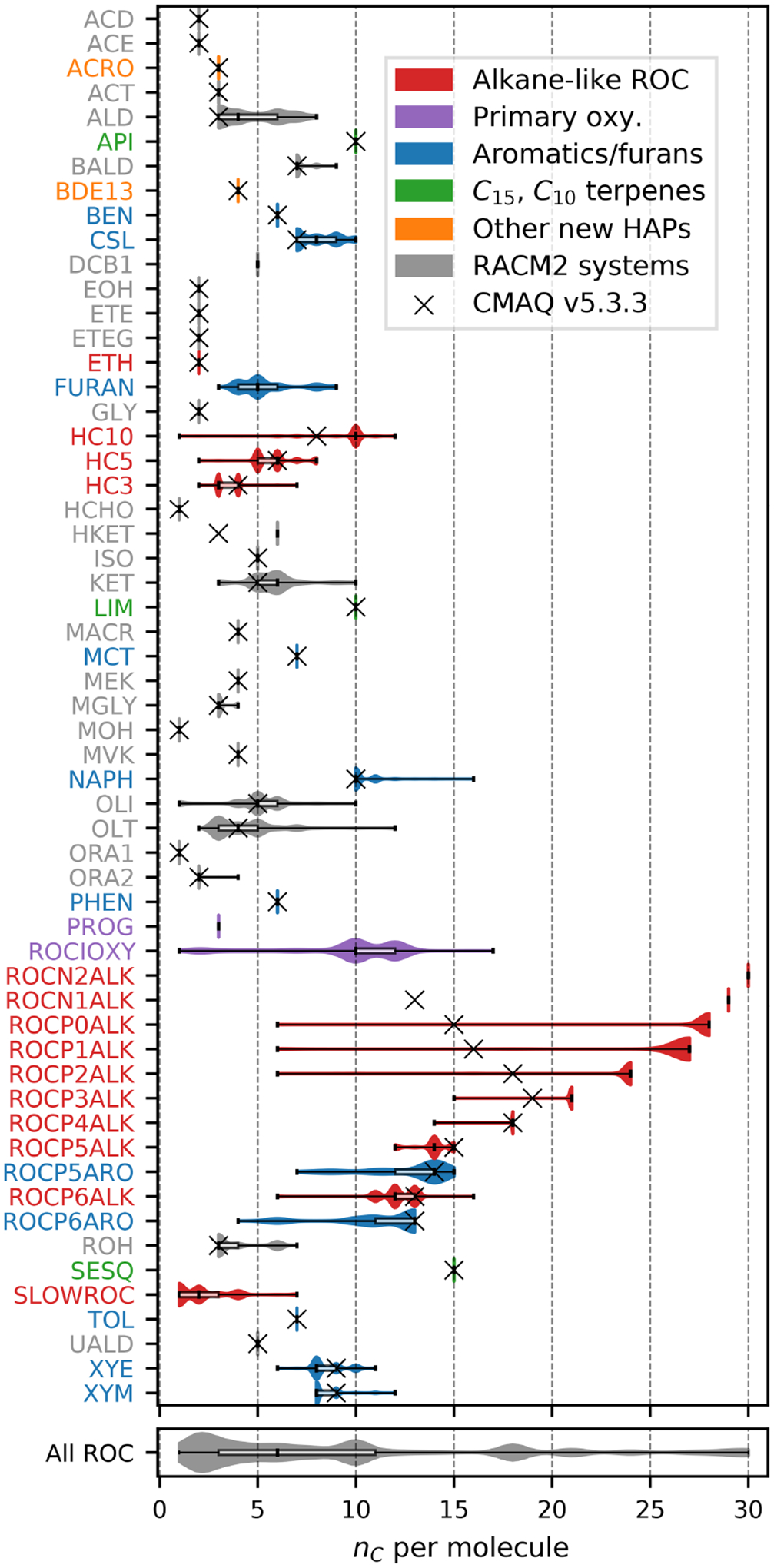
Emission-weighted number of carbon atoms per molecule of individual ROC species grouped by CRACMM species. Violin plots (with shaded colors for families of species in Sect. 3 that are either new or substantially updated compared to RACM2) are weighted by the magnitude of US anthropogenic and biomass burning emissions in 2017. Overlaid boxplots indicate the 25th percentile, median, and 75th percentile values. Whiskers extend from the minimum to the maximum properties for species with emissions > 100 Mg yr^−1^. CMAQv5.3.3 values are for RACM2 with the aerosol module AERO6 or represent an individual HAP from CMAQ. In some cases, the CMAQv5.3.3 values represent similar species from RACM2 (e.g., HC8 values at CRACMM HC10). Emission magnitudes by species are available in Table D2 (Pye, 2022) in the Supplement. Species names and abbreviations can be found in Appendices A and B.

**Figure 2.**
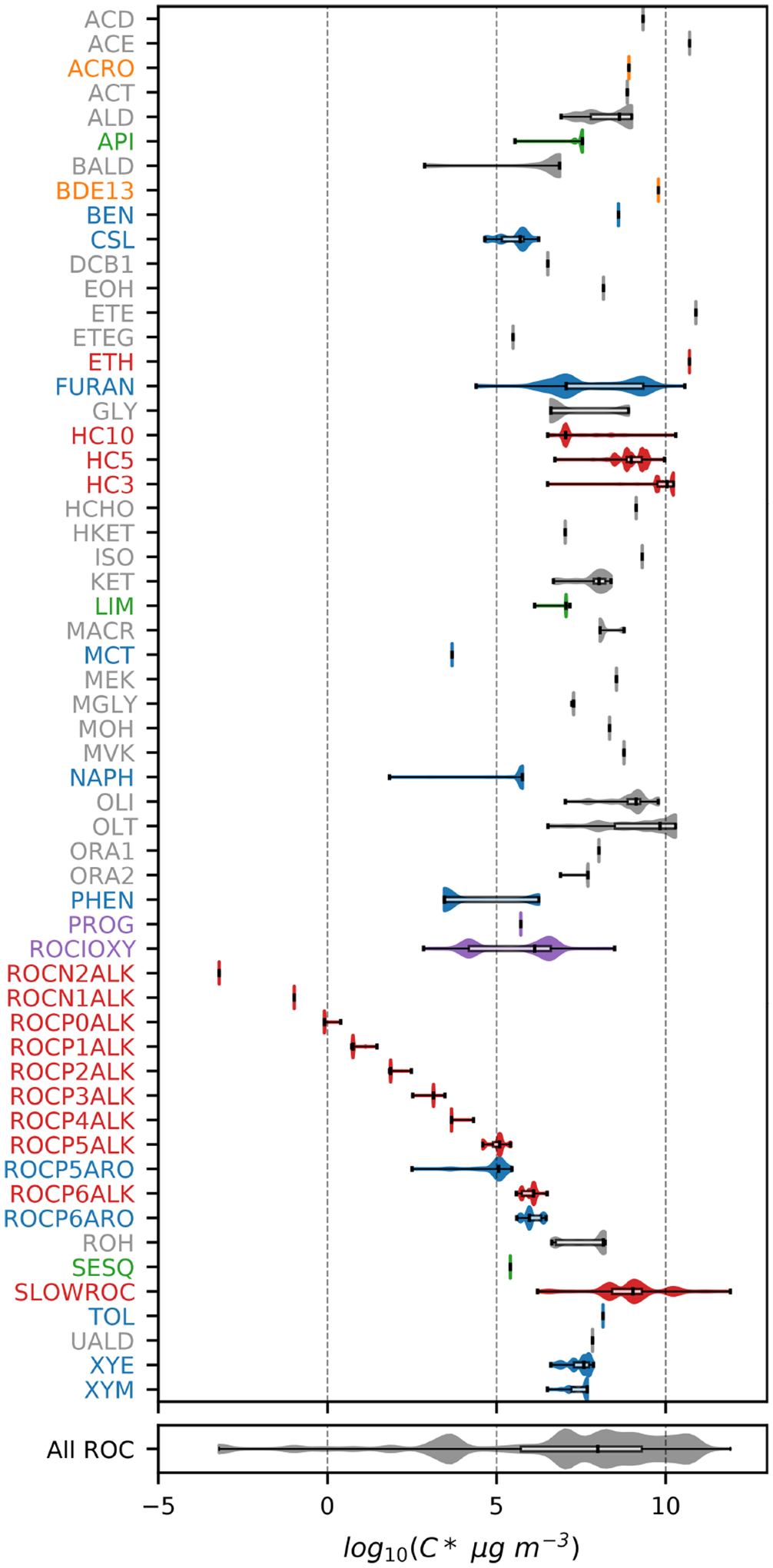
Same as Fig. 1 except the property displayed is the saturation concentration in ${\text{log}}_{10}\left(C_{i}^{*}\right)$.

**Figure 3.**
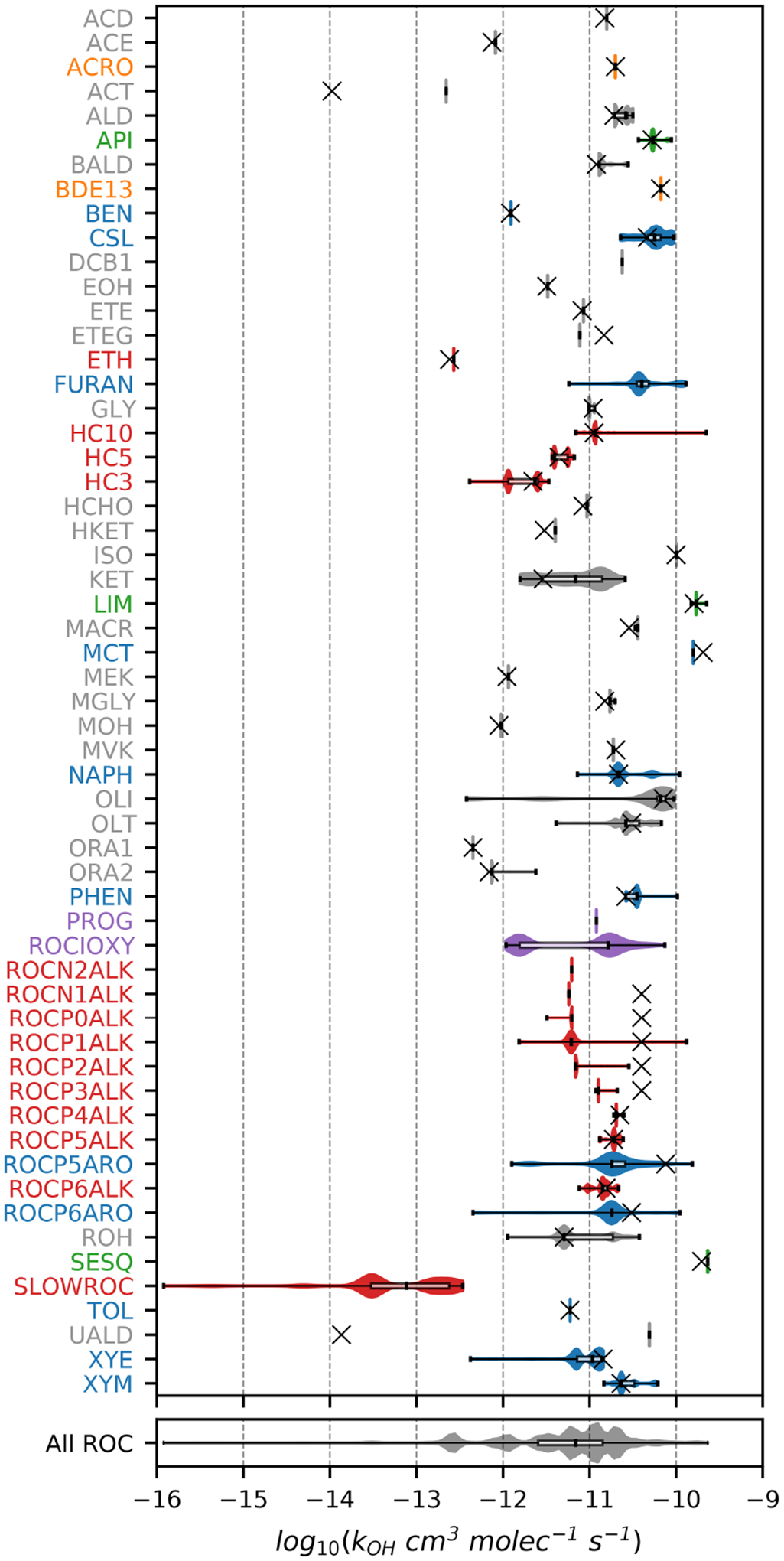
Same as Fig. 1 except the property displayed is the HO rate constant estimated by OPERA.

Figures 1–3 (and Supplement Figs. S5–S6) show the final 2017 US emission-weighted distributions of compound properties for all emitted ROC species in CRACMMv1.0. Looking across multiple properties illustrates the hierarchy of emission-mapping rules. For example, three classes of alkane-like species (discussed in Sect. 3.1) were inherited from RACM2: HC3, HC5, and HC10 (formerly HC8). In carbon number space (Fig. 1), these species overlap in their coverage of individual compounds with all three classes including species with two to eight carbons per molecule. Their saturation concentration distributions (Fig. 2) also show overlap. The ${\text{log}}_{10}\left(k_{\text{OH}}\right)$ (Fig. 3) highlights that HC3, HC5, and HC10 are defined by distinct and mutually exclusive ranges of the HO rate constant. Indeed, the HO rate constant is the classifying property for the HC3, HC5, and HC10 species and is implemented after volatility, functional-group identity, and other features of the species have been considered. As another example, SLOWROC is multimodal in the number of carbons per molecule ($n_{\text{C}}$) and $C_{i}^{*}$ (Figs. 1–2), which could necessitate separation into more species. However, SLOWROC reacts so slowly (Fig. 3) that additional speciation is not warranted. The systems in Figs. 1–3 indicated by color coding will be further discussed in the next section.

## 3 ROC chemistry

Multiple data sources were used to build the chemistry of CRACMM. As CRACMM will be a community mechanism in which different chemical systems are developed by different investigators, individual systems are expected to evolve at different rates and will be informed by different sources of data. Development of CRACMMv1.0 leveraged existing chemical mechanisms including the Generator for Explicit Chemistry and Kinetics of Organics in the Atmosphere (GECKO-A; Aumont et al., 2005), the Master Chemical Mechanism (MCM; Jenkin et al., 1997), the SAPRC-18 MechGen system (mechanism generation; Carter, 2020b), and RACM2, as well as literature. ROC systems not previously represented in RACM2 (such as furans and L/S/IVOCs), precursors to SOA, and systems with new kinetic data (Sect. 3.10) were targeted for development in this initial CRACMM version. Future work will continue to expand this initial representation by extending it to new chemical systems and/or updating these parameterizations with new data.

CRACMMv1.0 includes 178 gas-phase species (ROC species in Appendix A) and 508 reactions spanning gas-phase and heterogeneous pathways (Appendix B). In the CMAQv5.4 modal aerosol implementation, CRACMM includes 51 different chemical species in the particulate phase (81 model species across Aitken, accumulation, and coarse modes). These 51 particulate species in CRACMM include inorganic aerosol species such as sulfate, nitrate, ammonium, calcium, and other trace metals as in previous versions of CMAQ. To fully describe the state of atmospheric aerosol in CMAQ, CRACMM interacts with ISORROPIA II (Fountoukis and Nenes, 2007) and other algorithms describing nucleation and condensation. CRACMM specifically builds on the implementation of RACM2 chemistry coupled with aerosol chemistry of aerosol module 6 (AERO6) (411 reactions) in the CMAQv5.3.3 model, which differs slightly from the original RACM2 implementation (Goliff et al., 2013) (363 reactions) due to SOA pathways, parameterized effects of halogens on ozone (Sarwar et al., 2015), and other minor updates (see the work of Sarwar et al., 2013, and the “Code and data availability” section for the CMAQ implementation of RACM2).

In contrast to almost all SOA representations in current chemical transport models, SOA systems in CRACMM are integrated with the gas-phase radical chemistry. Specifically, all condensible or soluble precursors to SOA are formed directly as gas-phase products with the ability to condense (systems in Sect. 3.1–3.7) or react heterogeneously (Sect. 3.8) and form SOA. Formation of SOA thus removes mass from the gas phase, sequestering RO_2_, NO, and/or hydrogen oxide $\left({\text{HO}}_{x}\right)$ radicals with implications for ozone and species modulated by oxidant abundance such as sulfate.

All CRACMM species (both primary and secondary) have a representative structure (ROC species in Appendix A) based on the most abundantly emitted species or likely oxidation product. Representative structures were used to obtain properties such as the molecular weight, rate coefficient, solubility, and/or volatility of species except in two cases (SLOWROC in Sect. 3.1, VROCIOXY in Sect. 3.3). These representative structures can enable future prediction of other properties such as aerosol viscosity and the propensity to phase separate as well as deviations from ideal partitioning. They can also be used to synthesize CRACMM chemistry as demonstrated in Sect. 5. The species and chemistry of the major ROC systems updated compared to RACM2, reactions for two additional new HAPs, and rate constant updates (including many for inorganic reactions) are described in this section. Table 1 summarizes the SOA pathways.

### 3.1 Alkane-like ROC

CRACMM includes 14 classes of alkane-like species ranging from low-volatility compounds to ethane (Figs. 1–3 red series). Methane reaction with HO is from RACM2 and assumes a fixed background concentration (1.85 ppm for the late 2010s, Dlugokencky, 2022). After remapping all ROC species, the RACM2 alkane class HC8 (alkanes and other species with $k_{\text{OH}}>6.8\times 10^{-12}{\;\text{cm}}^{3}\;{\text{molec}.}^{-1}\;{\text{s}}^{-1}$) was renamed to HC10 based on the $n_{\text{C}}$ (Fig. 1) and is consistent with a $C_{i}^{*}\sim 10^{7}\;\mu \text{g}\;{\text{m}}^{-3}$ (Fig. 2). Nine new alkane-like mechanism species with high OA formation potential span the L/S/IVOC range and are grouped by ${\text{log}}_{10}\left(C_{i}^{*}\right)$ into ROCN2ALK, ROCN1ALK, ROCP0ALK, ROCP1ALK, ROCP2ALK, ROCP3ALK, ROCP4ALK, ROCP5ALK, and ROCP6ALK, where the numbers indicate the negative (N) or positive (P) ${\text{log}}_{10}\left(C_{i}^{*}\left[\mu \text{g}\;{\text{m}}^{-3}\right]\right)$ value (Fig. 2). When the species reside in the gas phase as a vapor, it is prepended with a “V” (as in Appendix B), and when in the particulate aerosol phase, it is prepended an “A.” For example, VROCN2ALK is an alkane-like vapor species with a $C_{i}^{*}$ of $10^{-2}\;\mu \text{g}\;{\text{m}}^{-3}$, and AROCN2ALK is a particulate species of the same volatility.

The nine new alkane-like model species roughly correspond to carbon numbers of 30, 29, 28, 27, 24, 21, 18, 14, and 12 (Fig. 1) and are not represented in traditional atmospheric chemical mechanisms due to low ozone formation potential per unit mass (Fig. S5). For example, ~ C_8_ is the largest alkane category in RACM2 and SAPRC-18, and n-dodecane (C_12_) is the largest alkane in MCM (Jenkin et al., 1997). Conceptually, for deposition and other processes, the gas-phase paraffinic species in the Carbon Bond version 6 (CB6) revision 3 is equivalent to a C_4_ species. Regardless of the chemical mechanism, regional modeling emission infrastructure previously used by CMAQ did not classify species with ~ 20 or more carbons (Pye and Pouliot, 2012), and S/IVOC emissions were not propagated to model-ready species for CMAQ mechanisms (Shah et al., 2020). The CRACMM species with ${\text{log}}_{10}\left(C_{i}^{*}\right)\leq 3$ can exist in the gas or particle phase based on the local organic aerosol loading and absorptive partitioning theory (Pankow, 1994), while ROCP4ALK–ROCP6ALK exist meaningfully in the gas phase only (Appendix A). The low-volatility alkanes, $C_{i}^{*}\leq 1\;\mu \text{g}\;{\text{m}}^{-3}$, are assumed to be primarily in the particulate phase and have a minor potential to react and contribute to O_3_ formation (Fig. S5) and so do not participate in gas-phase radical chemistry (Appendix B). Most of the L/S/IVOC emissions are expected to be unresolved at the individual-species level (Robinson et al., 2007) and are characterized through other means such as volatility analysis (e.g., Lu et al., 2018).

Gas-phase chemistry for the alkane species with $10\;\mu \text{g}\;{\text{m}}^{-3}\leq C_{i}^{*}\leq 10^{7}\;\mu \text{g}\;{\text{m}}^{-3}$ (ROCP1ALK–ROCP6ALK and HC10) is based on GECKO-A predictions for C_10_–C_26_ n-alkanes (Lannuque et al., 2018) and known H-shift pathways (Praske et al., 2018). The chemical reactions representing the major product channels and types of functionalities added to the parent hydrocarbon (RH) are the following:

(R1) $${\text{RH}}_{\text{M}=7,6,5,4,3,2,1},n_{\text{O}=0}+\text{HO}\to {\text{RO}}_{2}+{\text{H}}_{2}\text{O},$$

(R2) $${\text{RO}}_{2}+\text{NO}\to \left(1-\beta_{1}\right)\text{R}\left(\text{OH}\right){\text{O}}_{2}+\beta_{1}{\text{RNIT}}_{\text{M}-2.15,3}+\left(1-\beta_{1}\right){\text{NO}}_{2},$$

(R3) $${\text{RO}}_{2}+{\text{NO}}_{3}\to \text{R}\left(\text{OH}\right){\text{O}}_{2}+{\text{NO}}_{2},$$

(R4) $${\text{RO}}_{2}+{\text{HO}}_{2}\to {\text{ROOH}}_{\text{M}-3.02,2},$$

(R5) $$\text{R}(\text{OH}){\text{O}}_{2}\to \text{R}(\text{O}){\text{OOH}}_{\text{M}-3.40,3}+{\text{HO}}_{2},$$

(R6) $$\text{R}\left(\text{OH}\right){\text{O}}_{2}+\text{NO}\to \beta_{2}\text{R}\left(\text{OH}\right){\text{NIT}}_{\text{M}-4.33,4}+\left(1-\beta_{2}\right)\text{R}\left(\text{OH}\right){\text{KET}}_{\text{M}-2.96,2}+\left(1-\beta_{2}\right){\text{NO}}_{2}+\left(1-\beta_{2}\right){\text{HO}}_{2},$$

(R7) $$\text{R}(\text{OH}){\text{O}}_{2}+{\text{NO}}_{3}\to \text{R}(\text{OH}){\text{KET}}_{\text{M}-2.96,2}+{\text{NO}}_{2}+{\text{HO}}_{2},$$

(R8) $$\text{R}\left(\text{OH}\right){\text{O}}_{2}+{\text{HO}}_{2}\to \text{R}\left(\text{OH}\right){\text{OOH}}_{\text{M}-5.38,3},$$

where stable products are subscripted with their saturation concentration in ${\text{log}}_{10}\left(C_{i}^{*}\right)$ (relative to a parent hydrocarbon with ${\text{log}}_{10}\left.\left(C_{i}^{*}\right)=\text{M}\right)$ and the number of oxygens per molecule ($n_{\text{O}}$). For chemical reactions such as Reactions (R1)–(R9), RNIT, ROOH, ROH, and RKET indicate a compound with specific functionality rather than a mechanism species. The products in Reactions (R1)–(R9) are mapped to mechanism species based on their properties. The initial product, RO_2_, is the prompt peroxy radical resulting from hydrogen abstraction followed by an O_2_ addition (Reaction R1). RO_2_ reactions lead to stable products like organic nitrates (nitrate functionality generally indicated as RNIT in the above reactions) and peroxides (peroxide functionality generally indicated as ROOH in the above reactions) (Reactions R2, R4) that can further react (following Sect. 3.2 for S/IVOCs and RACM2 for VOCs). The alkoxy radical generated from the prompt RO_2_ can also undergo a 1,5 H shift followed by addition of O_2_ leading to a new hydroxy peroxy radical, R(OH)O_2_ (Reactions R2, R3). The R(OH)O_2_ can undergo standard bimolecular peroxy radical fates leading to multifunctional nitrates (R(OH)NIT), ketones (R(OH)KET), and peroxides (R(OH)OOH) or a 1,6 H shift at a rate of 0.188 s^−1^ (Vereecken and Nozière, 2020) producing a keto-hydroperoxide (R(O)OOH) and HO_2_ (Reaction R5) as described by Praske et al. (2018). Following GECKO-A (Lannuque et al., 2018), the yield of organic nitrates in Reaction (R2), $\beta_{1}$, is 0.28 for S/IVOC alkanes and 0.26 for HC10, consistent with the plateau at ~ 0.3 observed for C_13_ and larger alkanes (Yeh and Ziemann, 2014). The yield of organic nitrates for the hydroxy peroxy radical, $\beta_{2}$, is 0.14 for S/IVOC alkanes and 0.12 for HC10 (Lannuque et al., 2018). Rate constants are provided in Appendix B.

Products are often 2–3 orders of magnitude lower in $C_{i}^{*}$ than their parent and can be 4–5 orders of magnitude lower in the case of the multifunctional nitrates and peroxides. For the alkane systems, product $C_{i}^{*}$ is based on vapor pressures obtained from GECKO-A output using the Nannoolal method (Nannoolal et al., 2008, 2004). With one exception, all stable products from the VOC, HC10 (M=7), are expected to remain in the gas phase and thus map to the standard gas-phase species ONIT (organic nitrate), OP2 (organic peroxide), and KET (ketone) inherited from RACM2. The hydroxyhydroperoxide from HC10 oxidation is predicted to be sufficiently functionalized to be semivolatile. That C_10_ multifunctional peroxide along with all the stable products from alkane-like S/IVOCs are mapped to new CRACMM species of a matching $C_{i}^{*}$ and ratio of molar oxygen to carbon ($n_{\text{O}}:n_{\text{C}}$) (secondary oxygenated L/S/IVOC species, Sect. 3.2).

According to the SOA SAR (Fig. S5), as well as the prompt (one HO reaction) mechanism predictions (Table 1), SVOCs of $C_{i}^{*}=100\;\mu \text{g}\;{\text{m}}^{-3}$ and lower volatility have SOA yields that are near 100 % by mole (up to 150 % by mass), and the atmospherically relevant SOA yields will depend on competition between phase partitioning, reaction, and deposition. Much of the alkane-like L/SVOC contribution to ambient OA will be in the form of direct emission of the lower-volatility species as primary organic aerosol (POA). The mechanism-predicted prompt SOA yields for ROC3PALK and ROCP4ALK by mass (Table 1) are very similar to the emission-weighted SAR-based prediction of 0.83 and 0.55 by mass (Fig. S5). The mechanism-based prompt SOA yields for the more volatile alkane-like ROC species (ROCP5ALK, ROCP6ALK, and HC10) are lower than those predicted by the SOA SAR (28 %, 18 %, and 6 % by mass). Note that the HC10 class is estimated to contain substantial emissions (shown in Sect. 4 and accompanying Fig. 6b), some of which are poorly identified in SPECIATE (representative compound score of 1, Sect. 2.1).

The alkane-like ROC species differ from the previous CMAQ S/IVOC species implemented in AERO6–7 (× symbols in Figs. 1, 3) in terms of the trend in $n_{\text{C}}$ with volatility as they are all conceptualized as alkane-like structures because those are the representative structures currently populated with emissions in the S/IVOC range. SVOCs with ${\text{log}}_{10}\left(C_{i}^{*}\left[\mu \text{g}\;{\text{m}}^{-3}\right]\right)<2.5$ are lumped into ROCN2ALK–ROCP2ALK species based on volatility regardless of their functionality resulting in some higher $n_{\text{O}}:n_{\text{C}}$ species being included (Fig. S6). CMAQ AERO6–7 previously assumed a slight increase in $n_{\text{O}}:n_{\text{C}}$ and corresponding decrease in $n_{\text{C}}$ as volatility decreased (Figs. 1, S6). CRACMM alkane-like SVOCs with $k_{\text{OH}}$ from OPERA are also less reactive than AERO6–7 SVOCs (Fig. 3).

The reaction products of ethane (ETH), C_3_ alkanes and other slowly reacting species $\left(3.5\times 10^{-13}\leq k_{\text{OH}}<3.4\times \right.10^{-12}\;{\text{cm}}^{3}\;{\text{molec}.}^{-1}\;{\text{s}}^{-1},\text{HC}3)$, and C_5_ alkanes and other moderately reacting species $\left(3.4\times 10^{-12}\leq k_{\text{OH}}\leq 6.8\times \right.10^{-12}\;{\text{cm}}^{3}\;{\text{molec}.}^{-1}\;{\text{s}}^{-1}$, HC5) (Fig. 3) are obtained directly from RACM2 with the addition of a very small yield of SOA from HC3 ($2.8\times 10^{-5}$ by mole) and HC5 ($1.3\times 10^{-3}$ by mole) (Table 1). Ethane is the only explicit alkane in CRACMM; its rate constant with the hydroxyl radical is updated to follow recent recommendations (Burkholder et al., 2019). In addition, CRACMM includes a new species called SLOWROC with a lifetime of about 1 month ($k_{\text{OH}}<3.5\times 10^{-13}\;{\text{cm}}^{3}\;{\text{molec}.}^{-1}\;{\text{s}}^{-1}$) to prevent loss of emitted carbon that may contribute to the ambient atmospheric ROC burden (effective carbons per molecule of 2.1). SLOWROC also contains many HAPs (Sect. 4). Due to the highly empirical nature of SLOWROC, the molecular weight is based on an emission-weighted value rather than a representative compound. Oxidation of SLOWROC produces the ethylperoxy radical (ETHP) and a small yield of SOA (0.10 % by mole).

Effective SOA yields for the alkane-like VOC $\left({\text{log}}_{10}\left(C_{i}^{*}\left[\mu \text{g}\;{\text{m}}^{-3}\right]\right)\geq 6.5\right)$ systems except HC10 use the simple SAR for SOA and are driven by isopropyl acetate and methyl butanoate (estimated SOA yields of 2.8 % and 2.2 % by mass) in the case of HC3, by isopentane (estimated SOA yield of 1.9 % by mass) in the case of HC5, and by two long-lived aromatic species in the case of SLOWROC. The SOA from HC3, HC5, and SLOWROC is mapped to the species ASOAT, a general, non-volatile SOA species with a molecular weight of $200\;\text{g}\;{\text{mol}}^{-1}$ (Table 1). HC3, HC5, and SLOWROC are estimated to contribute 0.003 %, 0.062 %, and 0.0002 % by mass, respectively, of the total OA potential for anthropogenic and biomass burning emissions in the US for 2017 conditions.

### 3.2 Secondary oxygenated L/S/IVOCs

Gas-phase oxidation of S/IVOC alkanes readily leads to oxygenated L/S/IVOC products with $n_{\text{O}}:n_{\text{C}}$ ratios up to 0.3 (Reactions R1–R8). The products of these prompt reactions continue to be processed in the atmosphere, resulting in further functionalization as well as fragmentation (cleaving of the carbon backbone) with implications for increasing or decreasing SOA, respectively. Functionalization products of the secondary oxygenated L/S/IVOC chemistry can sequester radicals, but fragmentation products, like formaldehyde, can eventually release radicals via photolysis (Edwards et al., 2014).

The chemistry of secondary oxygenated L/S/IVOCs is parameterized using the 2-D VBS framework (Donahue et al., 2012) with some modifications. The decrease in ${\text{log}}_{10}\left(C_{i}^{*}\right)$ per oxygen in the 2-D VBS box model was calculated using the parameterization from Donahue et al. (2011) with the oxygen–oxygen interaction term set to 2.3, the carbon–oxygen interaction parameter set to −0.3 to correct for the behavior of diacids, and the carbon–carbon interaction term set to 0.475. As identified in Donahue et al. (2011), the resulting decrease in ${\text{log}}_{10}C^{*}$ per oxygen is 1.7 as $n_{\text{O}}:n_{\text{C}}$ approaches 0 and is 1.93 as $n_{\text{O}}:n_{\text{C}}$ approaches 0.6. These values are consistent with the effect of adding carboxylic acids to an alkane-like molecule (Pankow and Asher, 2008). Homogeneous, gas-phase HO reaction rate constants were specified based on the parameterization proposed by Donahue et al. (2013): $k_{\text{OH}}\left({\text{cm}}^{3}\right.\;{\text{molec}.}^{-1}\left.\;{\text{s}}^{-1}\right)≃1.2\times 10^{-12}\left(n_{\text{C}}+9n_{\text{O}}-10{\left(n_{\text{O}}:n_{\text{C}}\right)}^{2}\right).$ Following the reaction with HO, the probability of functionalization was parameterized as $f^{\text{func}}=1-{\left(n_{\text{O}}:n_{\text{C}}\right)}^{0.4}$, with subsequent probabilities of adding one, two, or three oxygens set at 30 %, 50 %, and 20 %, respectively, following the 2-D VBS functionalization kernel derived for photo-oxidation of POA and IVOCs (Zhao et al., 2016). The sensitivity of yields to ${\text{NO}}_{x}$ and formation of organic nitrates were not explicitly addressed in the 2-D-VBS-based aging mechanism, although both are addressed by CRACMM more broadly and some products mapped to secondary L/S/IVOCs contain nitrate functionality. Rather than recycling hydroxyl radicals as is standard practice for VBS-style reactions that are only meant to capture SOA, CRACMM sequesters ${\text{HO}}_{x}$ in oxygenated L/S/IVOC products as might be expected when peroxides form. For example, Reaction R1 followed by Reaction R4 sequester two ${\text{HO}}_{x}$ molecules for each initiating reaction.

L/S/IVOC products predicted by the 2-D VBS were lumped into a reduced series of 15 mechanism species spanning a $C_{i}^{*}$ of $10^{-2}$ through $10^{6}\;\mu \text{g}\;{\text{m}}^{-3}$ and $n_{\text{O}}:n_{\text{C}}$ of 0.1 through 0.8 for use in CRACMM: ROCN2OXY2, ROCN2OXY4, ROCN2OXY8, ROCN1OXY1, ROCN1OXY3, ROCN1OXY6, ROCP0OXY2, ROCP0OXY4, ROCP1OXY1, ROCP1OXY3, ROCP2OXY2, ROCP3OXY2, ROCP4OXY2, ROCP5OXY1, and ROCP6XY1. These species follow a naming convention similar to the S/IVOC alkanes, where numbers after “N” and “P” indicate the negative or positive ${\text{log}}_{10}\left(C_{i}^{*}\right)$ value and the name ends in $10\times n_{\text{O}}:n_{\text{C}}$ (e.g., ROCN2OXY2 is $C_{i}^{*}=10^{-2}\;\mu \text{g}\;{\text{m}}^{-3}$ with $n_{\text{O}}:n_{\text{C}}=0.2$). VBS products of a known $n_{\text{C}}$ and $n_{\text{O}}$ were mapped to the available CRACMM model species, first by interpolating to the two nearest ${\text{log}}_{10}\left(C_{i}^{*}\right)$ points and then to the two nearest species in $n_{\text{O}}:n_{\text{C}}$ space. The number of $n_{\text{O}}:n_{\text{C}}$ levels represented at a given volatility in CRACMM increases with decreasing $C_{i}^{*}$ to reflect increasing diversity in the chemical functionality and size of products with lower saturation concentrations.

The portion of reacted mass following the fragmentation pathway, $f^{\text{frag}}={\left(n_{\text{O}}:n_{\text{C}}\right)}^{0.4}$, was assumed to form fragments of sizes varying from one up to $n_{\text{C}}$ carbons. The distribution of fragments was estimated assuming the probability of attack on any carbon as $1/n_{\text{C}}$. Fragments with greater than seven carbons were functionalized using the same oxygen addition probabilities and remapping to lumped model species as above. Stable fragmentation products with six or fewer carbons were mapped back to existing gas-phase species from RACM2 based on their carbon number as follows: C_1_ to formaldehyde (HCHO), C_2_ to acetaldehyde (ACD), C_3_ to higher aldehyde species (ALD), C_4_ to methyl ethyl ketone (MEK), C_5_ to a dicarbonyl (DCB1), C_6_ from ${\text{low-}n}_{\text{O}}:n_{\text{C}}$ reactants to a hydroxy ketone (HKET), and C_6_ from $\text{high-}n_{\text{O}}:n_{\text{C}}$ reactants to a higher-carbon-number ketone (KET) species. The choice of functionality of the product species (e.g., aldehydes vs. ketones) is entirely determined by the RACM2 species that were already available at each carbon number. Future measurements of the low-molecular-weight species produced by the oxidation of larger compounds would help constrain this choice and motivate the addition of new CRACMM species. A new semivolatile peroxide (OP3), equivalent to a C_8_H_16_O_4_ species with a $C_{i}^{*}$ of $\sim 10\mu \text{g}\;{\text{m}}^{-3}$, in CRACMM provides an oxygenated peroxide species between the L/S/IVOC oxygenated series and RACM2’s higher organic peroxide species (OP2). In addition, radical products are mapped to RACM2 peroxy radical species as follows: C_1_ to methylperoxy radical (MO2), C_2_ to ethylperoxy radicals (ETHP), C_3_ to isopropylperoxy radicals (HC3P), C_4_ to peroxy radicals from methyl ethyl ketone (MEKP), C_5_ to pentan-3-ylperoxy radicals (HC5P), and C_6_ to ketone-derived peroxy radicals (KETP). OP3 can photolyze or react with HO.

**Figure 4.**
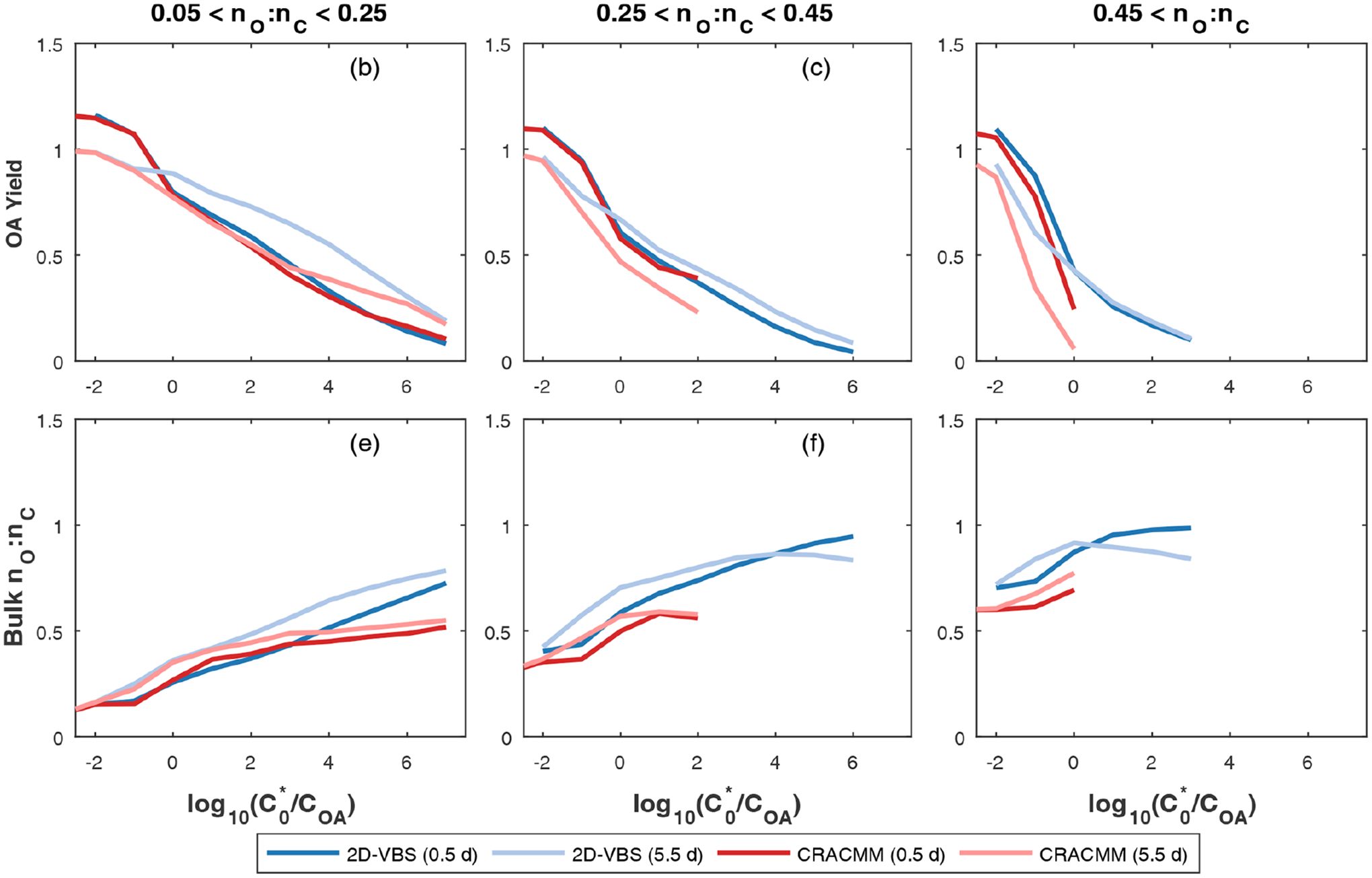
Organic aerosol yield and bulk $n_{O}:n_{C}$ predicted by the CRACMM oxygenated ROC aging mechanism (Sect. 3.2) and the 2-D VBS configuration reported by Zhao et al. (2016). The x axis is defined as ${\text{log}}_{10}\left(C_{0}^{*}/C_{\text{OA}}\right)$, where $C_{\text{OA}}$ is the background OA concentration and $C_{0}^{*}$ is the saturation concentration of the precursor. The aging of each species is simulated at a constant HO concentration of $10^{6}\text{molec}.{\text{cm}}^{-3}$ for 12 h (darker colors) and 2.5 d (lighter colors) at four different $C_{\text{OA}}$ conditions (0.1, 1, 10, and $100\;\mu \text{g}\;{\text{m}}^{-3}$). In cases where multiple predictions are present for the same saturation ratio, values are averaged.

Overall, the CRACMM scheme performs similarly to the medium-yield 2-D VBS scheme optimized for S/IVOCs by Zhao et al. (2016) (Fig. 4). For precursors with $n_{O}:n_{\text{C}}>0.05$ and 12 h of chemical processing, the 2-D VBS and CRACMM aging schemes are almost the same in terms of OA yield (Fig. 4a–c) with values ranging from near 0.1 to above 1 as a function of volatility (Table 1). Some deviations occur between the schemes for the most oxygenated and volatile precursors ($n_{\text{O}}:n_{\text{C}}>0.45$ and ${\text{log}}_{10}\left(C_{\text{OA}}/C_{i}^{*}\right)\leq 0$, where $C_{\text{OA}}$ is the mass-based concentration of the condensed-phase partitioning medium), for which CRACMM predicts a stronger dependence of yield on precursor volatility and also predicts less OA formation. Both CRACMM and the 2-D VBS predict consistent trends in OA yield as a function of precursor properties with more oxygenated and volatile precursors having lower yields due to an increased likelihood of fragmentation. At very long processing times CRACMM predicts OA yields will decrease (which has been observed in experimental systems in the work by He et al., 2022), while the 2-D VBS indicates yields continue to increase from 2.5 d (Fig. 4) to 5.5 d (Fig. S7). In CRACMM $n_{\text{O}}:n_{\text{C}}$ ratios are predicted to increase with time, which can be due to both functionalization (Heald et al., 2010) and fragmentation (Kroll et al., 2009) reactions. CRACMM generally predicts lower $n_{\text{O}}:n_{\text{C}}$ ratios in OA products from oxygenated ROC (0.1 to 0.5 for the least oxygenated and 0.6 to 0.7 for the most oxygenated precursors) than the 2-D VBS (Fig. 4d–f).

### 3.3 Primary oxygenated IVOCs

Volatile chemical products emit significant amounts of oxygenated IVOCs (Seltzer et al., 2021; McDonald et al., 2018). Many of these oxygenated species are structurally different than what is conceptualized in the secondary oxygenated L/S/IVOCs (Sect. 3.2) since they include siloxanes and ethers, while secondary oxygenated species are primarily alcohols, peroxides, nitrates, and ketones. Emitted oxygenated IVOCs have a significantly lower potential to form SOA than hydrocarbon IVOCs of a similar volatility (Pennington et al., 2021). In addition, oxygenated species generally differ from hydrocarbon-like emissions in their ability to form O_3_, peroxyacetyl nitrate (PAN), and formaldehyde (Coggon et al., 2021) and should be represented separately from hydrocarbon-like species.

Two new types of oxygenated IVOCs with direct emissions are included as distinct species in CRACMM (Figs. 1–3, purple): propylene glycol (PROG) and oxygenated IVOC species (VROCIOXY). 1,2-Propylene glycol is one of the most prevalent species in consumer product purchases (Stanfield et al., 2021) and is associated with increased allergic symptoms when inhaled (Choi et al., 2010). Propylene glycol is represented in CRACMM with chemistry based on MCM following the work of Coggon et al. (2021). The VROCIOXY class includes non-aromatic, saturated IVOCs with $n_{\text{O}}:n_{\text{C}}>0.1$ and all species containing silicon. Decamethylcyclopentasiloxane is the most abundant individual species in VROCIOXY, and VROCIOXY has an emission-weighted effective carbon number of 9.5. Due to the highly aggregated nature of VROCIOXY, the $k_{\text{OH}}$ and molecular weight are emission-weighted properties rather than based on a representative compound. VROCIOXY produces the ethylperoxy radical with an 85.2 % molar yield and SOA with a 14.9 % molar yield (Table 1) upon reaction with HO in CRACMM. While the SOA yield may appear high, the lifetime of VROCIOXY is 40 h at typical daytime HO concentrations, which should limit the amount of SOA in urban source regions, similar to siloxane behavior in the work of Pennington et al. (2021). Future versions of CRACMM emission processing could redirect alcohols, carbonyls, and other oxygenated S/IVOCs from VROCIOXY to the secondary oxygenated L/S/IVOC series (Sect. 3.2) and readjust the effective VROCIOXY SOA yield.

### 3.4 Furans

FURAN is a new lumped ROC species introduced in CRACMM with the most abundant individual species in the category being furfural followed by furan. Furans were not previously an independent category in RACM2, and Carter (2020a) recommended mapping 2-furfural to $\sim {\text{C}}_{8}$ hydrocarbons (now HC10) and furan to the lumped o-xylene (XYO in RACM2). Given the abundance of furans ($140\;\text{Gg}\;{\text{yr}}^{-1}$ of emission, primarily from wood burning for 2017 US conditions), unique functional-group structure, HO reactivity (Koss et al., 2018), and O_3_ formation potential (Coggon et al., 2019), FURAN was implemented in CRACMM as a new species (Figs. 1–3, blue). Furans have been shown to form SOA with yields between 1.85 % and 8.5 % by mass depending on the structure (Gómez Alvarez et al., 2009), and the simple SAR predicts a yield of 2.6 % by mass (Fig. S5). The furan SOA yield is about a factor of 4 lower than that of xylenes, but products such as furanone (FURANONE, a new species in CRACMM) are also formed in aromatic systems like benzene (Sect. 3.5). The CRACMM species, FURAN, includes small amounts of other species with two double bonds (Fig. S3) including $2.4\;\text{Gg}\;{\text{yr}}^{-1}$ of anthropogenic dienes.

The FURAN chemistry in CRACMM is based on a fivespecies weighted average using furan emission factors reported by Koss et al. (2018) and the furan chemistry outlined by Wang et al. (2021) and Coggon et al. (2019). FURAN will predominantly react with hydroxyl radicals, leading to gas-phase products including dicarbonyls (DCB1, DCB3), organic nitrates (ONIT), peroxides (OP2), furanones (FURANONE), and aldehydes (ALD) in addition to radicals (Appendix B). CRACMM assigns SOA from FURAN to further reactions in the ring-retaining product channel, FURANONE, consistent with products detected by Jiang et al. (2019). The effective SOA yield from FURAN is approximately 5 % by mass (Bruns et al., 2016) when branching between high- and low-${\text{NO}}_{x}$ reactions is equal. The yield of SOA from FURANONE in CRACMM is set to 4 % by mole or 8 % by mass (Table 1).

### 3.5 Aromatics

Aromatic hydrocarbons (Figs. 1–3, blue) were reorganized to reduce the number of aromatic VOC model species and increase the number of aromatic IVOC species in CRACMM. Instead of four aromatic VOC categories based on reactivity $\left(k_{\text{OH}}\right)$, CRACMM uses two categories of xylene-like hydrocarbon species based on reactivity: m-xylene and more reactive aromatics (XYM) and aromatics less reactive than m-xylene (XYE). Toluene (TOL), a HAP (Sect. 4), is now explicit in CRACMM, and benzene (BEN) was already explicit in RACM2. The three new IVOC aromatic hydrocarbons ($n_{\text{O}}:n_{\text{C}}=0$) are naphthalene and other polycyclic aromatic hydrocarbons (NAPH), single-ring aromatics of ${\text{log}}_{10}\left(C_{i}^{*}\right)\approx 5$ (ROCP5ARO), and single-ring aromatics of ${\text{log}}_{10}\left(C_{i}^{*}\right)\approx 6$ (ROCP6ARO). The ROCP5ARO and ROCP6ARO categories were previously found to be important for representing SOA from vehicle combustion sources (Lu et al., 2020), and the emissions for 2017 indicated insufficient mass and SOA formation potential to warrant another aromatic species at ${\text{log}}_{10}\left(C_{i}^{*}\right)\approx 4$.

MCMv3.3.1 chemistry (Bloss et al., 2005; Jenkin et al., 2003) was used to obtain a basic mechanism for aromatic reaction for seven hydrocarbon-like aromatics in CRACMM (BEN, TOL, XYE, XYM, NAPH, ROCP6ARO, and ROCP5ARO). The MCM epoxide yield (which includes unidentified species mass, Birdsall and Elrod, 2011) was set to 0, and product mass was redirected to the bicyclic peroxy channel following Xu et al. (2020). In addition, the organic nitrate yield (β, Reaction R11) from ${\text{RO}}_{2}+\text{NO}$ is 0.2 % in CRACMM (Xu et al., 2020). A fraction of the bicyclic peroxy radical channel is assumed to undergo autoxidation (Wang et al., 2017; Molteni et al., 2018; Xu et al., 2020). The following reactions describe this chemistry for a parent aromatic species (BEN, TOL, etc.), generally indicated as AROM:

(R9) $${\text{AROM}}_{\text{M},0}+\text{HO}\to \left(1-\alpha_{\text{PL}}\right){\text{ARO}}_{2}+\alpha_{\text{PL}}{\text{PL}}_{\text{M-}2.14,1}+\alpha_{\text{PL}}{\text{HO}}_{2},$$

(R10) $${\text{ARO}}_{2}+{\text{HO}}_{2}\to \alpha_{\text{H}}/\left(1-\alpha_{\text{PL}}\right){\text{ROOH}}_{\text{M}-2.48,2}^{\text{H}}+\left(1-\alpha_{\text{H}}-\alpha_{\text{PL}}-\alpha_{\text{A}}\right)/\left(1-\alpha_{\text{PL}}\right){\text{ROOH}}_{\text{M-}4.529,5}^{\text{B}}+\alpha_{\text{A}}/\left(1-\alpha_{\text{PL}}\right){\text{ROOH}}_{\text{M-}7.558,7}^{\text{A}},$$

(R11) $${\text{ARO}}_{2}+\text{NO}\to \beta \alpha_{\text{H}}/\left(1-\alpha_{\text{PL}}\right){\text{RNIT}}_{\text{M-}2.23,3}^{\text{H}}+\beta \left(1-\alpha_{\text{H}}-\alpha_{\text{PL}}-\alpha_{\text{A}}\right)/\left(1-\alpha_{\text{PL}}\right){\text{RNIT}}_{\text{M-}4.279,6}^{\text{B}}+\beta \alpha_{\text{A}}/\left(1-\alpha_{\text{PL}}\right){\text{RNIT}}_{\text{M-}7.308,8}^{\text{A}}+(1-\beta ){\text{NO}}_{2}+(1-\beta )\alpha_{\text{H}}/\left(1-\alpha_{\text{PL}}\right){\text{ROP}}_{\text{M-}1.35,1}^{\text{H}}+(1-\beta )\left(1-\alpha_{\text{H}}-\alpha_{\text{PL}}\right)/\left(1-\alpha_{\text{PL}}\right){\text{ROP}}^{\text{B}},$$

(R12) $${\text{ARO}}_{2}+{\text{NO}}_{3}\to {\text{NO}}_{2}+\alpha_{\text{H}}/\left(1-\alpha_{\text{PL}}\right){\text{ROP}}_{\text{M-}1.35,1}^{\text{H}}+\left(1-\alpha_{\text{H}}-\alpha_{\text{PL}}\right)/\left(1-\alpha_{\text{PL}}\right){\text{ROP}}^{\text{B}},$$

(R13) $${\text{ARO}}_{2}+{\text{RRO}}_{2}\to {\text{RRO}}_{2}\text{P}+\alpha_{\text{H}}/\left(1-\alpha_{\text{PL}}\right){\text{ROP}}_{\text{M-}1.35,1}^{\text{H}}+\left(1-\alpha_{\text{H}}-\alpha_{\text{PL}}\right)/\left(1-\alpha_{\text{PL}}\right){\text{ROP}}^{\text{B}}.$$

Stable, individual species are subscripted with their ${\text{log}}_{10}\left(C_{i}^{*}\right)$ relative to the parent volatility of M (estimated with SIMPOL (simple $p_{L}^{o}$ prediction method; Pankow and Asher, 2008) based on expected functionality) and number of oxygens per molecule. The phenolic product (PL) yield ($\alpha_{\text{PL}};53\%$ for benzene and 16 %–18 % otherwise) is from MCM (o-xylene if a species was not available) and independent of NO level, in good agreement with experimental data for conditions below a few hundred parts per billion of NO (Bates et al., 2021). The PL product is mapped to phenol (for benzene), cresols (for toluene and xylenes), or a lumped secondary oxygenated product (described in Sect. 3.2) based on volatility and $n_{\text{O}}:n_{\text{C}}$ (for all other aromatics). Aromatic peroxy radical $\left({\text{ARO}}_{2}\right)$ products included peroxides, organic nitrates, and alkoxy radical decomposition products (ROPs). ROPs are produced by H abstraction (H), traditional HO addition resulting in bicylic peroxy radicals (B), and/or autoxidation (A). The fraction of all AROM + HO through the H-abstraction route $\left(\alpha_{\text{H}}\right)$ is from MCM with the product mapped to benzaldehyde in the case of toluene and xylenes or a product based on expected volatility and $n_{\text{O}}:n_{\text{C}}$ (H abstraction is not applicable for benzene). The ROP^B^ from the bicylic peroxy radical alkoxy radical decomposition channel follows MCM and includes glyoxal and/or methylglyoxal, furanones, dicarbonyl(s), and ${\text{HO}}_{2}.\alpha_{\text{A}}$ is the fraction of products undergoing autoxidation and is a subset of the bicyclic RO_2_ products. Coefficients in Reactions (R9)–(R13) $\left(\alpha_{\text{H}},\alpha_{\text{PL}},\alpha_{\text{A}}\right)$ are relative to total AROM + HO except for the fraction of RO_2_ + NO branching to organic nitrates (β) in Reaction (R11).

Aromatic peroxy radicals can react with other organic peroxy radicals (RRO_2_), with methylperoxy radicals and acetylperoxy radicals being the most abundant and always represented in RACM2 (Stockwell et al., 1990). The RRO_2_ product (RRO_2_ P) is based on MCM at yields specified independently of the ARO_2_ product channels. Specifically, methylperoxy radicals (RRO_2_ as RACM 2 species MO_2_) result in 0.68 formaldehyde, 0.37 HO_2_, and 0.32 higher alcohols (RRO_2_ P=0.68 HCHO + 0.37 HO_2_ + 0.32 MOH). Acetylperoxy radicals (RRO_2_ as RACM2 species ACO3) result in 0.7 methylperoxy radicals and 0.3 acetic acid (RRO_2_ P = 0.7 MO2 + 0.3 ORA2).

Reactions (R9)–(R13) produce condensible gases and SOA precursors. In the case of volatile aromatics like benzene, toluene, and xylenes, further reaction of the phenolic product along with autoxidation is proposed as the major SOA channels in CRACMM since traditional bimolecular RO_2_ products are generally not of sufficiently low volatility. For aromatic IVOCs, peroxides, nitrates, and aldehydes from bimolecular RO_2_ reactions can be semivolatile and partition based on their saturation concentration. Further oxidation of furanone produced from aromatic oxidation (e.g., Reaction 477, Appendix B) also results in small amounts of SOA (Sect. 3.4). For products in Reactions (R9)–(R13) that are mapped to a corresponding surrogate of matching volatility and $n_{\text{O}}:n_{\text{C}}$, further chemical processing follows the secondary oxygenated S/IVOC chemistry in Sect. 3.2.

CRACMM retains the three phenolic species of RACM2 (hydroxy-substituted benzene like phenol and benzene diols, PHEN; cresol-like species, CSL; and methylcatechols and similar species, MCT) with the same gas-phase chemistry as RACM2 except for the addition of one non-volatile SOA product for PHEN and CSL. The yield of SOA from phenols and cresols is set to reproduce the $\text{high-}{\text{NO}}_{x}$ SOA yields from benzene and toluene oxidation observed in chamber experiments by Ng et al. (2007) with wall loss corrections based on Zhang et al. (2014) (see the Supplement for a detailed derivation). The molar SOA yield using this method is estimated as 15 % by mole for phenols and 20 % by mole for cresols (Table 1), within the range of 24 %–52 % by mass for phenols and 27 %–49 % by mass for cresols as summarized by Bruns et al. (2016). Future work should expand upon this phenolic SOA treatment as improvements in the phenoxy–phenylperoxy radical chemistry have been shown to modulate O_3_ formation and could improve predictions for laboratory conditions over MCM, RACM2, and SAPRC by breaking the catalytic radical cycles (Bates et al., 2021). Products like methylcatechols could also lead to SOA with implications for O_3_ and HO production in aromatic systems.

The bicyclic peroxy radical fate in aromatic hydrocarbon systems is not well characterized but includes autoxidation. Molteni et al. (2018) estimate molar yields of autoxidation products from aromatic oxidation of just under 3 % by mole, and that value is used for the aromatic IVOC systems in CRACMM ($\alpha_{\text{A}}=0.03$). Higher values are not needed to produce significant SOA in IVOCs systems since traditional bimolecular RO_2_ fates result in sufficiently functionalized products to contribute to SOA. Specifically, with $\alpha_{\text{A}}=0.03$, CRACMM predicts SOA yields for ROCP5ARO, ROCP6ARO, and NAPH of 37 %, 21 %, and 21 % by mole, respectively (Table 1). However, such low levels of autoxidation, even when combined with phenolic (PHEN and CSL) SOA, are insufficient to explain observed SOA production for the more volatile aromatics, particularly in RO_2_ + HO_2_-dominant conditions, where SOA yields are around 27 % by mole based on chamber experiments. Xu et al. (2020) indicate bicyclic peroxy radicals in the benzene system may predominantly form alkoxy radicals (even in RO_2_ + HO_2_ conditions) that continue to highly oxygenated organic molecules in addition to other products. Given the current lack of carbon closure for gas-phase aromatic chemistry (Xu et al., 2020) and low volatility of laboratory-generated RO_2_ + HO_2_ aromatic SOA (Ng et al., 2007), the amount of autoxidation in the benzene, toluene, and xylene aromatic systems is set in CRACMM to reproduce observed RO_2_ + HO_2_ chamber SOA yields when combined with the phenolic channel (see the Supplement for molar yield derivation). The resulting estimates for the fraction of AROM + HO reaction leading to autoxidation $\left(\alpha_{\text{A}}\right)$ are 19 % by mole for benzene and 23 % by mole for toluene and xylenes. This results in the phenolic channel contributing 30 % of the SOA in the benzene system and 13 % in the toluene systems for RO_2_ + HO_2_ conditions, similar to the previously published estimate of 20 % for low-${\text{NO}}_{x}$ conditions for benzene, toluene, and m-xylene (Nakao et al., 2011) and 20 %–40 % for toluene (Schwantes et al., 2017) as well as the relative abundance of phenolic products in benzene vs. toluene systems.

In general, autoxidation of the bicyclic RO_2_ in the aromatic systems is assumed to involve one H shift followed by O_2_ addition and result in peroxides and nitrates about seven ${\text{log}}_{10}\left(C_{i}^{*}\right)$ values lower in volatility than the parent aromatic (products in Reactions R10–R11). The autoxidation product in benzene and toluene systems with only one H shift would have a $C_{i}^{*}$ of $10\;\mu \text{g}\;{\text{m}}^{-3}$, making it semivolatile according to SIMPOL (Pankow and Asher, 2008). To improve consistency with Ng et al. (2007) yields and non-volatile partitioning behaviors under $\text{low-}{\text{NO}}_{x}$ conditions at low organic aerosol concentrations ($<10\;\mu \text{g}\;{\text{m}}^{-3}$), the products from autoxidation in the toluene and benzene systems are assumed to result from two H shifts followed by O_2_ addition leading to two additional hydroperoxide functional groups and autoxidation products with $C_{i}^{*}=0.01\;\mu \text{g}\;{\text{m}}^{-3}$. Xylene-like (XYM and XYE) autoxidation products assume one H shift with O_2_ addition resulting in autoxidation products with $C_{i}^{*}=1\;\mu \text{g}\;{\text{m}}^{-3}$. ROOH^B^ products from XYM and XYE are slightly lower in volatility than those from benzene and toluene and mapped to the new multifunctional C_8_ peroxide (OP3; see Sect. 3.2 and Table 1), resulting in SOA from channels other than autoxidation and phenolic routes for xylenes. SOA yields for benzene, toluene, and xylenes summarized in Table 1 generally reproduce wall-loss-corrected laboratory values (Ng et al., 2007; Zhang et al., 2014) due to the imposed autoxidation channel. Benzene and toluene are predicted to have lower SOA yields than the IVOC aromatics NAPH, ROCP5ARO, and ROCP6ARO. However, the amount of autoxidation for aromatic IVOCs was not adjusted to match literature SOA yields, since many traditional bimolecular products were already in the S/IVOC range and thus SOA for aromatic IVOCs could be underestimated compared to laboratory work (Srivastava et al., 2022).

**Figure 5.**
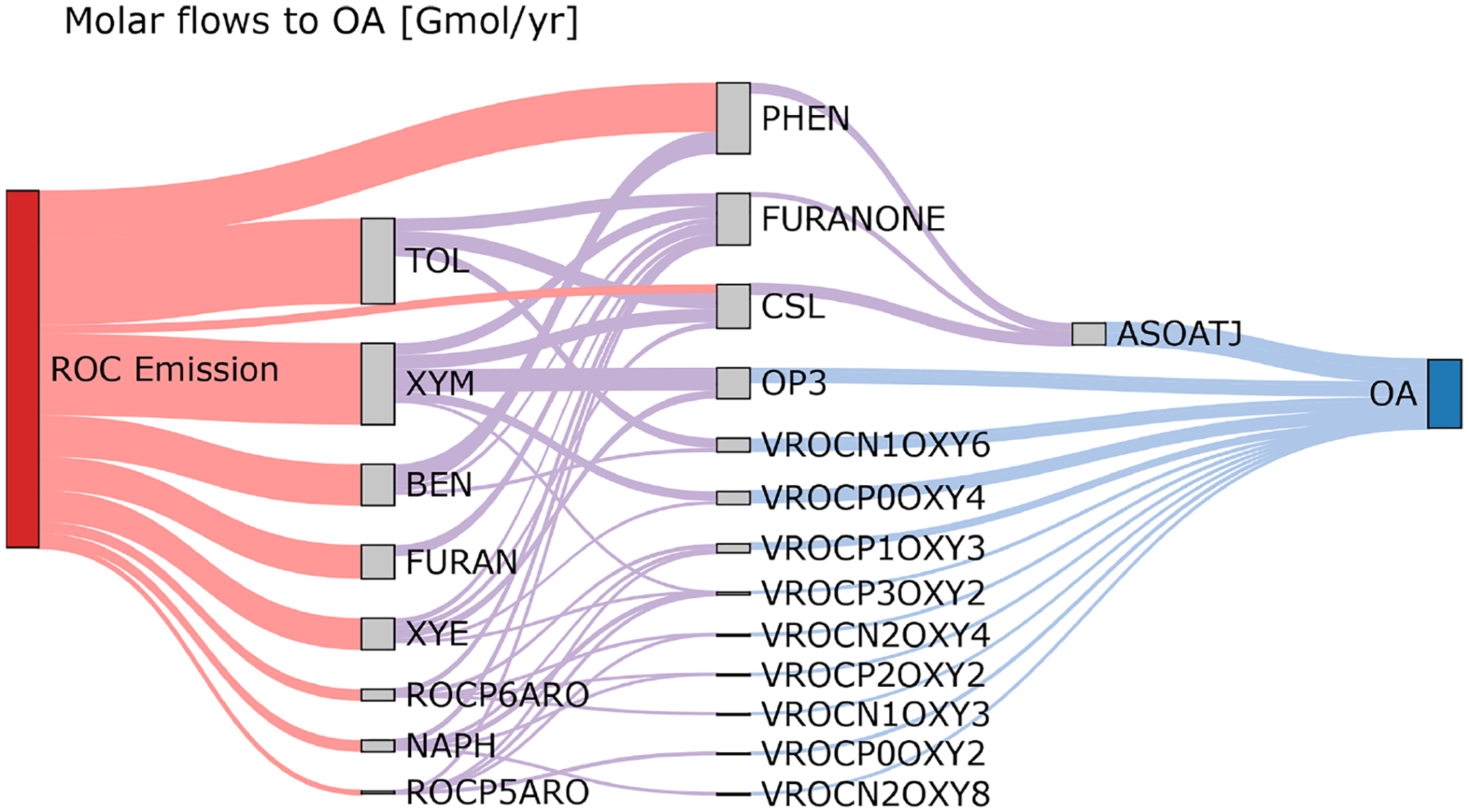
Molar flows to organic aerosol in the aromatic–phenolic–furan systems for 2017 US emissions. Bimolecular ${\text{RO}}_{2}$ reactions are split equally between ${\text{RO}}_{2}+\text{NO}$ and ${\text{RO}}_{2}+{\text{HO}}_{2}$ with the fraction of products undergoing autoxidation as specified in CRACMM. Partitioning of semivolatile species is calculated for $10\;\mu \text{g}\;{\text{m}}^{-3}$ of organic aerosol. Precursor species include the following: toluene (TOL), m-xylene and more reactive aromatic VOCs (XYM), benzene (BEN), ethylbenzene and less reactive aromatic VOCs (XYE), phenolic species (PHEN), cresols (CSL), naphthalene and PAHs (NAPH), and other IVOC aromatics of higher (ROCP6ARO) and lower (ROCP5ARO) volatility. Aqueous pathways to SOA from glyoxal and methylglyoxal are not shown. Products that do not lead to OA are not shown but are indicated by the outflow from a species being smaller than the inflow. Red flows indicate emissions. Purple flows indicate hydroxyl radical oxidation chemistry. Blue flows indicate partitioning to the condensed phase.

Figure 5 shows the molar flows to organic aerosol in the combined aromatic, phenolic, and furan systems based on anthropogenic and biomass burning emissions in the US for 2017 and equal RO_2_ + HO_2_ vs. RO_2_ + NO branching. Most (69 %) phenol mass is directly emitted with the balance from benzene oxidation. In contrast, cresols are predominantly chemically produced (80 % of the source) rather than directly emitted. Approximately 22 % of furanone is produced directly from furan oxidation, but most furanone is predicted to be from oxidation of aromatic hydrocarbons like toluene and xylenes with smaller contributions from IVOC aromatics. About 32 % of the aromatic system SOA is predicted to come from phenols, cresols, and furanone through fixed yields and the formation of an empirical SOA species (ASOATJ). Peroxide species (specifically OP3) may be a substantial contributor to SOA mass. Autoxidation, leading to species such as ROCN1OXY6, also make meaningful contributions to the predicted SOA mass. By acknowledging further oxidation of phenolic species as contributors to overall aromatic hydrocarbon SOA, all phenolic emissions can now be considered SOA precursors. In addition, adding phenolic sources of SOA increases the overall amount of SOA from ROC emissions compared to previous CMAQ aerosol representations that did not include phenols or cresols as SOA precursors.

### 3.6 Sesquiterpenes

Sesquiterpenes (C_15_H_24_) are a new radical system in CRACMM (previously only considered for SOA formation in CMAQ; Figs. 1–3, green) with chemistry built using β-caryophyllene from MCM (Jenkin et al., 2012) and autoxidation based on literature. β-Caryophyllene is an IVOC $\left({\text{log}}_{10}\left(C_{i}^{*}\right)\right.$ of $\left.5.05\;\mu \text{g}\;{\text{m}}^{-3}\right)$, and MCM chemistry readily predicts sesquiterpene products that are S/IVOCs, consistent with the semivolatile nature of observed SOA (Griffin et al., 1999). Sesquiterpene species (SESQ) react with NO_3_, O_3_, and HO :

(R14) $${\text{SESQ}}_{5.05,0}+{\text{NO}}_{3}\to {\text{SESQNRO}}_{2},$$

(R15) $${\text{SESQNRO}}_{2}+{\text{HO}}_{2}\to {\text{ROOH}}_{0.34,5},$$

(R16) $${\text{SESQNRO}}_{2}+{\text{NO}}_{2}\to {\text{RKET}}_{2.72,2}+2{\text{NO}}_{2},$$

(R17) $${\text{SESQNRO}}_{2}+{\text{NO}}_{3}\to {\text{RKET}}_{2.72,2}+2{\text{NO}}_{2},$$

(R18) $${\text{SESQ}}_{5.05,0}+{\text{O}}_{3}\to \left(1-\alpha_{\text{A}}\right){\text{RKET}}_{2.72,2}+\alpha_{\text{A}}{\text{PA}}_{-2,3},$$

(R19) $${\text{SESQ}}_{5.05,0}+\text{HO}\to {\text{SESQRO}}_{2},$$

(R20) $${\text{SESQRO}}_{2}+{\text{HO}}_{2}\to {\text{ROOH}}_{0.34,3},$$

(R21) $${\text{SESQRO}}_{2}+{\text{NO}}_{3}\to {\text{RKET}}_{2.72,2},$$

(R22) $${\text{SESQRO}}_{2}+{\text{NO}}_{2}\to \beta {\text{RNIT}}_{0.59,4}+(1-\beta ){\text{RKET}}_{2.72,2}+(1-\beta ){\text{NO}}_{2},$$

where $\alpha_{\text{A}}$ is the fraction of ozonolysis products undergoing autoxidation and β is the fraction of RO_2_ + NO products resulting in organic nitrates (β=0.25). The ozonolysis Reaction (R18) is highly simplified and predicted to result in a ketone (ketone functionality indicated by RKET) and autoxidation product (PA) of specified volatility and degree of oxygenation. Autoxidation is based on Richters et al. (2016) and $\alpha_{A}$ is set to 1.8 % by mole. Observations indicate sesquiterpenes are not major contributors by mass to ambient SOA in the Amazon (Yee et al., 2018), southeastern US, or boreal forest (Lee et al., 2020). As a result, CRACMM does not retain the unique identity of sesquiterpene products, and all stable products in Reactions (R14)–(R22) are mapped to the corresponding secondary oxygenated S/IVOC of corresponding volatility and degree of oxygenation with further chemistry specified in Sect. 3.2.

CRACMM predicts prompt (first-generation) sesquiterpene SOA that is less volatile than previous CMAQ work (Carlton et al., 2010; Griffin et al., 1999), is ${\text{NO}}_{x}$ and oxidant dependent, and has the potential for higher yields through multigenerational chemistry. The yield of prompt SOA under RO_2_ + HO_2_-dominant conditions is predicted to be 50 % $\left(\text{OA}=1\;\mu \text{g}\;{\text{m}}^{-3}\right)$ to 91 % $\left(\text{OA}=10\;\mu \text{g}\;{\text{m}}^{-3}\right)$ by mole for HO and NO_3_ oxidation. These low-NONO_3_ yields are within the range of those observed in NO_3_ oxidation experiments (SOA yields of 56 %–109 % by mole of C, Jaoui et al., 2013), although laboratory values corresponded to a higher concentration of organic aerosol $\left(60-110\;\mu \text{g}\;{\text{m}}^{-3}\right)$ and the RO_2_ fate was not characterized. Under higher-${\text{NO}}_{x}$ conditions (RO_2_ + NO dominant) and moderate organic aerosol loading $\left(\text{OA}=10\;\mu \text{g}\;{\text{m}}^{-3}\right)$, prompt SOA yields are expected to be ~ 12 % by mole from HO oxidation, similar to the carbon-based yields of aerosol from laboratory work (19 % by mole for β-caryophyllene, Jaoui et al., 2013). Nitrate oxidation is not expected to produce significant SOA when RO_2_ reacts with NO or NO_3_ (Reactions R16–R17), and prompt SOA yields from ozonolysis are 2.7 % by mole, lower than the observed yield of 28 % by mole C for ozonolysis (Jaoui et al., 2013). Thus, further chemical processing of first-generation sesquiterpene-derived ketones (mapped to CRACMM species ROCP3OXY2; chemistry in Sect. 3.2) likely results in lower-volatility species that increase SOA yields beyond the prompt values, especially under $\text{high-}{\text{NO}}_{x}$ and ozonolysis conditions.

### 3.7 Monoterpenes

CRACMM retains the two monoterpene categories of RACM2 with α-pinene and Δ-limonene as the major representative compounds in each class (API and LIM, respectively; Figs. 1–3, green). The two classes differ in the number of double bonds per species, which is expected to influence reactivity and SOA formation potential (Hoffmann et al., 1997). In addition, species with two double bonds in their initial structure likely experience faster autoxidation (Møller et al., 2020). The two classes of monoterpenes (API vs. LIM) have different sources of emissions, with α-pinene being predominantly from vegetation but limonene having the potential for significant anthropogenic emissions from volatile chemical products (Coggon et al., 2021) in addition to biogenic sources. A new representation of API and LIM reaction with HO, NO_3_, and O_3_ was created to account for autoxidation leading to highly oxygenated molecules and SOA. In addition, bimolecular peroxy radical reactions leading to dimers of extremely low volatility (CRACMM species ELHOM) with the potential to contribute to new particle formation via nucleation (Bianchi et al., 2019) were added.

When a monoterpene (MT) species reacts with an oxidant like HO (or NO_3_), it directly forms a collection of peroxy radicals (generally indicated as MRO_2_ and ${\text{MRO}}_{2}^{\text{A}}$; see Appendices A and B for specific model species), a fraction of which $\left(\alpha_{\text{A}}\right)$ can undergo autoxidation and form highly oxygenated molecules:

(R23) $$\text{MT}+\text{Oxidant}\to \left(1-\alpha_{\text{A}}\right){\text{MRO}}_{2}+\alpha_{\text{A}}{\text{MRO}}_{2}^{\text{A}}.$$

Autoxidation is implemented as a fixed yield rather than competitive fate since autoxidation in monoterpene + HO systems proceeds rapidly (rates of 3 to > 10 s^−1^) and only via specific peroxy radical isomers (Piletic and Kleindienst, 2022; Zhao et al., 2018; Berndt et al., 2016; Xu et al., 2019). This assumption of a fixed yield is valid for bimolecular RO_2_ lifetimes (timescale for RO_2_ reaction with NO or HO_2_) greater than ~ 1 s (NO < ~ 1 ppb), which is consistent with most current conditions near earth’s surface except for select urban locations, more often in winter (Porter et al., 2021), and episodically near sources. The fraction of prompt API + HO peroxy radicals undergoing autoxidation and forming monoterpene-derived highly oxygenated molecules (tracked as CRACMM species HOM) $\left(\alpha_{\text{A}}\right)$ is set to 2.5 % by mole (Berndt et al., 2016; Piletic and Kleindienst, 2022) with the uncertainty in the yield around a factor of 2. Limonene is expected to have rapid H-shift reactions (Møller et al., 2020) and higher amounts of autoxidation products than α-pinene (Jokinen et al., 2015), and $\alpha_{\text{A}}$ is 5.5 % for LIM + HO (Piletic and Kleindienst, 2022) (Table S7).

The peroxy radicals from monoterpene (API and LIM) reactions with HO undergo traditional bimolecular RO_2_ fates leading to peroxides, alkoxy radical products, and nitrates:

(R24) $${\text{MRO}}_{2}+{\text{HO}}_{2}\to \text{ROOH},$$

(R25) $${\text{MRO}}_{2}+\text{NO}\to (1-\beta ){\text{NO}}_{2}+\alpha_{\text{ALD}}\times (1-\beta ){\text{ROP}}_{\text{ALD}}+\left(1-\alpha_{\text{ALD}}\right)\times (1-\beta ){\text{ROP}}_{\text{FRAG}}+\beta \text{RNIT}.$$

MRO_2_ also reacts with MO2 and ACO3 (see Sect. 3.5) (Appendix B). Peroxides from an MRO_2_ reaction with HO_2_ (Reaction R24) map to a new organic peroxide, OPB, added specifically to represent the C_10_ hydroperoxides from monoterpene oxidation. Further reaction or photolysis of OPB is assumed to produce products like existing organic peroxide reactions in RACM2 with products fed back to the lumped aldehyde (ALD), ketone (KET), and saturated C_10_ RO_2_ (HC10P). To better conserve carbon and track the identity of monoterpene-derived nitrates, CRACMM includes a new C_10_ organic nitrate, TRPN (Reaction R25, RNIT product). The OPB peroxides and TRPN nitrates are assumed to remain in the gas phase (see representative structures in Appendix A).

The yield of organic nitrates (β, Reaction R25) is 18 % for API (Nozière et al., 1999) and 23 % for LIM based on MCMv3.3.1 (Saunders et al., 2003). Further reaction of the terpene nitrates produces LVOCs with a 100 % molar yield (Zare et al., 2019; Browne et al., 2014), with products mapped to then new lumped CRACMM species, HOM. While the yield of SOA from the TRPN reaction is 100 % by mole, chemical sinks will compete with deposition, resulting in less than 100 % of TRPN converted to SOA in chemical transport models.

In addition to terpene nitrates, major organic products from RO_2_ + NO (Reaction R25) are alkoxy radicals which decompose to either aldehydes and HO_2_ (ROPALD) with a yield of $\alpha_{\text{ALD}}$ or other smaller-carbon-number fragmentation products and HO_2_ (ROPFRAG). In the case of LIM $\left(\alpha_{\text{ALD}}=64\%\right)$, the alkoxy radical decomposition products are assumed to be smaller fragments (HCHO and UALD), but $\alpha_{\text{ALD}}=1$ for α-pinene according to MCM. Since the aldehydes from API and LIM could undergo autoxidation as hinted by Rolletter et al. (2020), new aldehydes, PINAL and LIMAL, were added for the monoterpene systems. Autoxidation for PINAL and LIMAL is added as competitive fate with plausible autoxidation rate constant for terpene systems $\left(k=1\;{\text{s}}^{-1}\right)$ for HO-initiated peroxy radicals formed at a yield of 23 % (PINALP) or 70 % (LIMALP) based on MCMv3.3.1. LIMAL and PINAL can also be lost via photolysis, and LIMAL can react with O_3_. In general, rate constants in monoterpene systems (Appendix B) are from RACM2.

In the case of an API and LIM reaction with nitrate radicals, reactions analogous to Reactions (R23)–(R25) generally apply, but products are multifunctional and can release NO_2_. Nitrate radical reactions are assumed to behave similarly in terms of autoxidation and use the same $\alpha_{\text{A}}$ as HO reactions, which is likely in the case of limonene (J. Chen et al., 2021) but an overestimate in the case of α-pinene (Kurtén et al., 2017). For reactions where multifunctional peroxy nitrates (or other multifunctional nitrates) are expected, the nitrate identity is prioritized for tracking and the product is mapped to TRPN. Reaction of nitrate-derived MRO_2_ with NO is expected to predominantly release all the nitrate as ${\text{NO}}_{2}(\beta =0)$ and convert NO to NO_2_ (additional NO_2_ product alongside aldehyde production) while yielding a terpene aldehyde (PINAL or LIMAL) $\left(\alpha_{\text{ALD}}=1\right)$.

${\text{MRO}}_{2}^{\text{A}}$ from autoxidation in monoterpene + HO systems is implemented using two new peroxy radicals (labeled APIP2 and LIMP2) that are assumed to result in C_10_O_7_ radicals (Berndt et al., 2016) that can undergo traditional bimolecular fates. For all API and LIM reactions with HO and NO_3_, the ${\text{MRO}}_{2}^{\text{A}}+{\text{HO}}_{2}$ product is mapped to HOM. In the case of ${\text{MRO}}_{2}^{\text{A}}+\text{NO}$, all products that release ${\text{NO}}_{2}(1-\beta )$ are also assumed to re-release HO via different fragmentation routes and the highly oxidized terpene nitrate as well as other carbon-containing products were mapped to HOM. ${\text{MRO}}_{2}^{\text{A}}+\text{MO}2$ and ${\text{MRO}}_{2}^{\text{A}}+\text{ACO}3$ aldehydes, ketones, and alcohols are also mapped to HOM. As a result, under all conditions, the yield of HOM from the initial API or LIM reaction with HO or NO_3_ is $\alpha_{\text{A}}$.

The speciation of HOM changes slightly when ${\text{MRO}}_{2}^{\text{A}}$ cross-react with other monoterpene or isoprene RO_2_. In addition to the traditional peroxy radical cross-reactions with other organic peroxy radicals (MO2 and ACO3), the monoterpene-derived peroxy radicals undergoing autoxidation, ${\text{MRO}}_{2}^{\text{A}}$, react with the most abundant ${\text{MRO}}_{2}$ from α-pinene and limonene + HO to produce C_20_ dimers. These reactions followed the basic form of

(R26) $${\text{MRO}}_{2}^{\text{A}}+{\text{MRO}}_{2}\to \left(1-\alpha_{\text{dim}}\right)\text{HOM}+0.5\times \left(1-\alpha_{\text{dim}}\right)\text{ROH}+0.5\times \left(1-\alpha_{\text{dim}}\right){\text{ROP}}_{\text{ALD}}+0.5\times \left(1-\alpha_{\text{dim}}\right)\text{HO}+0.5\times \left(1-\alpha_{\text{dim}}\right){\text{HO}}_{2}+\alpha_{\text{dim}}\text{ELHOM},$$

where $\alpha_{\text{dim}}$ is the fraction of ${\text{MRO}}_{2}^{\text{A}}$ incorporated in dimers and set to 4 % based on the work of Zhao et al. (2018). Other products include highly oxygenated monomers (mapped to HOM), aldehydes (mapped to PINAL or LIMAL), and alcohols with branching between those products also as specified by Zhao et al. (2018). In the case of nitrate-initiated ${\text{MRO}}_{2}^{\text{A}},{\text{NO}}_{2}$ rather than HO is released. The same approach is used for monoterpene ${\text{MRO}}_{2}^{\text{A}}$ + isoprene RO_2_ with HCHO and MVK produced rather than PINAL or LIMAL. Dimer reactions are assumed to proceed quickly, and the rate constant was set to $1\times 10^{-10}\;{\text{cm}}^{3}\text{molec}.^{-1}\;{\text{s}}^{-1}$ based on the work of Molteni et al. (2019). In both the monoterpene and isoprene cross-reactions, the dimer products are predicted to have a ${\text{log}}_{10}\left(C_{i}^{*}\right)<-3$ and are mapped to ELHOM.

The ozonolysis of monoterpenes in CRACMM also mimics Reaction (R23), where the oxidant in these reactions is O_3_. Initially, the ozonolysis reaction will break a monoterpene double bond and yield Criegee intermediates that selfreact to release hydroxyl radicals and produce peroxy radicals which were classified into the same two types of peroxy radical categories as with HO reactions: either autoxidizable or non-autoxidizable. The yield of peroxy radicals able to undergo autoxidation $\left({\text{MRO}}_{2}^{\text{A}}\right)$ for ozonolysis is set to 5 % and 11 %, respectively, in the API and LIM systems. These yields are doubled compared to HO to fall within the uncertainty in laboratory and computational studies that indicated autoxidation yields from O_3_-initiated reactions are universally higher than autoxidation from HO-initiated chemistry (Jokinen et al., 2015; Ehn et al., 2014; J. Chen et al., 2021). The formation of HO, H_2_O_2_, CO, and aldehyde products from the ozonolysis reactions alongside ${\text{MRO}}_{2}^{\text{A}}$ were prescribed following MCM and RACM2, and further reaction of the MRO_2_ and ${\text{MRO}}_{2}^{\text{A}}$ peroxy radicals is the same as in the HO system.

Predicted SOA in the monoterpene systems comes from HOM and ELHOM products that are either promptly produced or from a further reaction of terpene nitrates or terpene aldehydes. The yield of SOA from an API reaction with HO or NO_3_ is expected to be 2.5 % by mole (4.6 % by mass) from the initial autoxidation HOM but is further increased to 11 % by mole (21 % by mass) when the terpene nitrates further react under typical ambient conditions (Table 1). Under high-${\text{NO}}_{x}$ conditions (RO_2_ + NO as the dominant bimolecular fate), the yield of SOA from API + HO approaches 37 % by mass with most of the mass from terpene nitrate products, highlighting the importance of the terpene nitrate fate which is currently assumed to be a reaction with HO and functionalization. LIM SOA yields from HO and NO_3_ are similar with values of 16 % by mole or 30 % by mass for typical conditions but as much as 50 % by mass if RO_2_ + NO dominates and terpene nitrates react further. Yields also increase compared to the typical values if the terpene aldehydes react with HO, which is estimated to yield SOA of 21 % by mole (31 % by mass) or 64 % by mole (95 % by mass) for PINAL and LIMAL, respectively. Terpene aldehyde photolysis, OPB (and OP3) reaction with HO, or LIMAL reaction with O_3_ can also lead to trace amounts of SOA via a C_10_ RO_2_ product (<1 % molar yield; chemistry in Sect. 3.1 for the HC10 peroxy radical).

The autoxidation-derived HOM yield for α-pinene from CRACMM is similar to the computed yield predicted by Weber et al. (2020) using a more detailed CRI-HOM (Common Representative Intermediates approach for highly oxygenated organic molecules) mechanism that invoked multigenerational peroxy radical chemistry in a global atmospheric chemistry model. Other models have applied numerous autoxidation mechanisms of varying complexity including a steady-state HOM yield assumption similar to CRACMM (Gordon et al., 2016), a volatility basis set model (Schervish and Donahue, 2020), and a near-explicit autoxidation mechanism involving 1773 reactions (Roldin et al., 2019). While the fixed HOM yields implemented in CRACMM consolidate the mechanism, additional species and reactions are considered here including NO_3_ oxidation chemistry, the chemistry of reactive monoterpenes like limonene, and many accretion reactions that may produce ELHOM. Further refinements to the autoxidation mechanism will be considered in future CRACMM versions including an implementation of the temperature dependence of H-shift reactions, potentially revised volatilities for HOM and ELHOM, and fragmentation reactions of highly oxidized peroxy radicals that may limit HOM production.

The CRACMM approach to monoterpene organic nitrates differs from previous CMAQ approaches where organic nitrates were incorporated into the particle via heterogenous uptake driven by hydrolysis reactions (Pye et al., 2015; Zare et al., 2019). CRACMM indicates a potentially significant role for TRPN in forming SOA but via a different mechanism than previous work which assumed a 3 h lifetime against condensed-phase hydrolysis ($k_{\text{HET}}$ (defined in a footnote in Appendix B) of $1.13\times 10^{-7}\;{\text{s}}^{-1}$). TRPN could also release ${\text{NO}}_{x}$ upon chemical reaction (Saunders et al., 2003) and fragment into smaller molecules (Weber et al., 2020) which are not considered here. Future versions of CRACMM should incorporate monoterpene nitrate hydrolysis and release ${\text{NO}}_{x}$ upon reaction where appropriate.

Note that the identity of terpene nitrates when they are lumped into HOM or ELHOM is not retained. Lowervolatility nitrates, peroxides, ketones, and alcohols from terpene oxidation are lumped together based on volatility with HOM having an effective ${\text{log}}_{10}\left(C_{i}^{*}\right)$ of 0 to −3 and a representative structure with ${\text{log}}_{10}\left(C_{i}^{*}\right)$ of −2.2. ELHOM species are nominally highly oxygenated C_20_ dimers with an effective ${\text{log}}_{10}\left(C_{i}^{*}\right)$ of −5, but species with ${\text{C}}_{15}$ structures are also mapped to ELHOM based on their volatility (estimated as $\left.{\text{log}}_{10}\left(C_{i}^{*}\right)<-3\right)$). Given the importance of volatility as a driver of new particle formation events (McFiggans et al., 2019), the resolution in volatility for highly oxidized products should be investigated in future work in the context of predicting new particle formation events.

### 3.8 Isoprene and aqueous aerosol pathways

The treatment of isoprene chemistry in CRACMM version 1.0 is the same as in RACM2–AERO6 as implemented in CMAQv5.3.3. Notably, the CMAQ implementation includes formation of isoprene epoxydiols (IEPOX) as a tracer. An investigation of isoprene chemistry in CRACMM using the Automated MOdel REduction (AMORE) condensation of a detailed isoprene mechanism (Wennberg et al., 2018) with isoprene nitrate hydrolysis (Vasquez et al., 2020) is available in the work of Wiser et al. (2023) and as CRACMM1AMORE in CMAQv5.4.

Precursors to SOA from aqueous reactions include IEPOX, glyoxal (GLY), and methylglyoxal (MGLY) and follow CMAQ AERO7. GLY is a lumped species, and emissions include glycolaldehyde (total 2017 US GLY emissions: $418\;\text{Gg}\;{\text{yr}}^{-1}$). MGLY is also lumped and includes 2-oxobutanal and other carbonyl aldehydes (total 2017 US MGLY emissions: $1129\;\text{Gg}\;{\text{yr}}^{-1}$). SOA from IEPOX uptake follows the reactive uptake formulation of Pye et al. (2013) with the Henry’s law coefficient for IEPOX $\left(3.0\times 10^{7}\text{M}\;{\text{atm}}^{-1}\right)$ and an organosulfate condensed-phase formation rate constant ($\left.8.83\times 10^{-3}{\text{M}}^{-2}\;{\text{s}}^{-1}\right)$) from the work of Pye et al. (2017). New in CRACMM compared to the standard AERO7 in CMAQ are separate species for the organosulfate (AISO3OS) vs. non-sulfated (2-methyltetrol, AISO3NOS) IEPOX-derived SOA to facilitate tracking of sulfur. Reactive uptake of GLY and MGLY on aqueous particles uses a fixed uptake coefficient $\left(2.9\times 10^{-3}\right)$ (Liggio et al., 2005) as in CMAQ version 5.2–5.3.3 (Pye et al., 2015). Cloud-processed SOA from GLY and MGLY is based on the reaction with aqueous HO and the work of Carlton et al. (2008). Glyoxal SOA may include formation of salt-like structures in the aerosol phase (Paciga et al., 2014), but, for simplicity, the oligomeric structure of Loeffler et al. (2006) is used as the representative structure of all glyoxal and methylglyoxal SOA. Note that the molecular weight of GLY and MGLY SOA specified in CRACMM differs from the representative structure. Aqueous reaction products leading to SOA in CRACMM, as implemented in CMAQ, are not currently allowed to volatilize to the gas phase, which likely occurs for a subset of IEPOX products (Riedel et al., 2015; D’Ambro et al., 2019).

### 3.9 Acrolein and 1,3-butadiene

Acrolein (ACRO) is a major oxidation product of 1,3-butadiene (BDE13), and both species were added explicitly in CRACMM due to their importance for health (Scheffe et al., 2016) (see Sect. 4). For a BDE13 reaction with HO, which is likely its dominant removal pathway (Agency for Toxic Substances and Disease Registry, 2012; Tuazon et al., 1999), the SAPRC-18 MechGen utility (Carter, 2020b) was used to generate products that are mapped to the analogous CRACMM species. SAPRC-18 MechGen is convenient since the products are already aggregated to a similar degree as RACM2 and CRACMM. A peroxy radical specific to the BDE13 reaction with HO (BDE13P) is used so that formation of acrolein (from all channels except BDE13P + HO_2_) could be explicitly predicted. For BDE 13 + O_3_, a Criegee biradical is predicted to be a significant product in SAPRC-18 and MCMv3.3.1. Criegee biradicals are not implemented in CRACMM due to their short lifetime, so MCMv3.3.1 was used to determine the likely products from Criegee decomposition. For simplicity, the BDE13 reaction with nitrate follows the diene + NO_3_ products from RACM2 with acrolein instead of MACR specified as the product. Products from a reaction of ACRO with HO and NO_3_ are taken from RACM2’s lumped MACR species. In the case of ACRO ozonolysis, prompt products as well as the expected Criegee biradical products are from MCM. ACRO photolysis products are from SAPRC-18 MechGen.

### 3.10 Additional rate constant updates

The inorganic chemistry of RACM2 is retained in CRACMM with updated rate constants for some reactions. In CRACMM, rate expressions for 26 inorganic reactions and 2 organic reactions (carbon monoxide and methane with HO; ethane as mentioned in Sect. 3.1) were updated compared to RACM2 values (IUPAC, 2010; Sander et al., 2011; Goliff et al., 2013) to follow the NASA JPL (Jet Propulsion Laboratory) evaluation number 19 (Burkholder et al., 2019) and IUPAC recommendations (Atkinson et al., 2004). Photolysis rate coefficients were updated for five chemical species: C_3_ and higher aldehydes (ALD), acetone (ACT), methyl ethyl ketone (MEK), higher ketones (KET), and formaldehyde (HCHO). The photolysis rate coefficient for ALD is set to that of propionaldehyde from the NASA JPL evaluation number 19 recommendation (Burkholder et al., 2019). CRACMM adds the acetone photolysis pathway producing a methylperoxy radical and carbon monoxide in addition to the existing RACM2 pathway that produces methyl peroxy and acetyl peroxy radicals. Quantum yields of ACT are updated following the NASA JPL evaluation number 19 recommendation (Burkholder et al., 2019). In addition, the temperature and pressure effects on ACT photolysis rate coefficients now follow Blitz et al. (2004). Photolysis rate coefficients and products of MEK and KET use quantum yield from Raber and Moortgat (1996) and absorption cross-sections from Brewer et al. (2019). The photolysis pathway for formaldehyde in RACM2 contained an error in quantum yield data resulting in overestimated photolysis rate coefficients, which are now corrected in CRACMM using data from the NASA JPL evaluation number 19 recommendation. These general kinetic updates are expected to lead to minor decreases in O_3_ formation compared to RACM2–AERO6.

## 4 ROC hazardous air pollutants

Hazardous air pollutants are known or suspected to cause serious adverse health or environmental effects and are therefore a priority to represent in chemical mechanisms. However, the number of HAPs routinely considered should be moderated for computational efficiency. While 189 substances are designated as HAPs by the U.S. EPA, HAP species such as polycyclic organic matter (POM) and glycol ethers contain many individual compounds such that the actual number of individual species meeting the definition of a HAP is well over 3000 (U.S. Environmental Protection Agency, 2022c). The SPECIATE database, which includes a HAP identifier, was used as the initial source of identification for the species-level emission inventory and supplemented with additional data sources. POM was identified based on species with more than one benzene ring and $n_{\text{O}}:n_{\text{C}}=0$ in their representative structure (an additional 56 species on top of the HAP category in SPECIATE). The POM requirement of a boiling point above 100°C was found to be duplicative with the aromaticity criteria based on the work of Achten and Andersson (2015). The identifier of 1-bromopropane, a newly designated HAP (U.S. Environmental Protection Agency, 2022a), was updated. SPECIATE was also cross-referenced with individual glycol ethers (U.S. Environmental Protection Agency, 2022c) (four additional HAPs). CAS (Chemical Abstracts Service) numbers of individual species and their representative structures were cross-referenced with the toxicity value file input to the Human Exposure Model (U.S. Environmental Protection Agency, 2021a) identifying an additional 39 HAPs. Overall, 491 HAPs were identified in SPECIATE, of which 188 had non-zero ROC emissions in the 2017 inventory used here.

To assess the coverage of HAPs and their toxicity in CRACMM, toxicity potentials were estimated using chronic inhalation metrics from the U.S. Environmental Protection Agency (2021b). The EPA’s process for estimating a cancer risk is based on the unit risk estimate (URE), which is the estimated number of excess tumors per person due to inhalation of $1\;\mu \text{g}\;{\text{m}}^{-3}$ of the pollutant over a lifetime. Non-cancer (mutagenicity, developmental toxicity, neurotoxicity, and/or reproductive toxicity) risk uses a reference concentration (RfC), which is an estimate of the concentration that could be inhaled over a lifetime without an appreciable risk. Species in SPECIATE were matched to the inhalation RfC and URE values (U.S. Environmental Protection Agency, 2021a) by the CAS number. A few SPECIATE species (2,4-toluene diisocyanate, an m- and p-xylene mixture, an m- and p-cresol mixture, and a chrysene mixture) were manually mapped to relevant exposure risk values. In cases where a species in SPECIATE did not have a CAS or unique structure, a representative structure was used for mapping. A relative non-cancer toxicity potential was estimated based on the emitted mass of a species divided by the RfC, and a relative cancer toxicity potential was estimated as the product of the emissions and URE (Simon et al., 2010). For species designated as HAPs but not included in the toxicity value table (U.S. Environmental Protection Agency, 2021a), an RfC of $20\;\text{mg}\;{\text{m}}^{-3}$ and URE of $1\times 10^{-8}\;{\mu \text{g}}^{-1}\;{\text{m}}^{3}$, corresponding to the maximum RfC and minimum URE values for known HAPs, were used to provide what is potentially a conservative underestimate of risk potential.

**Figure 6.**
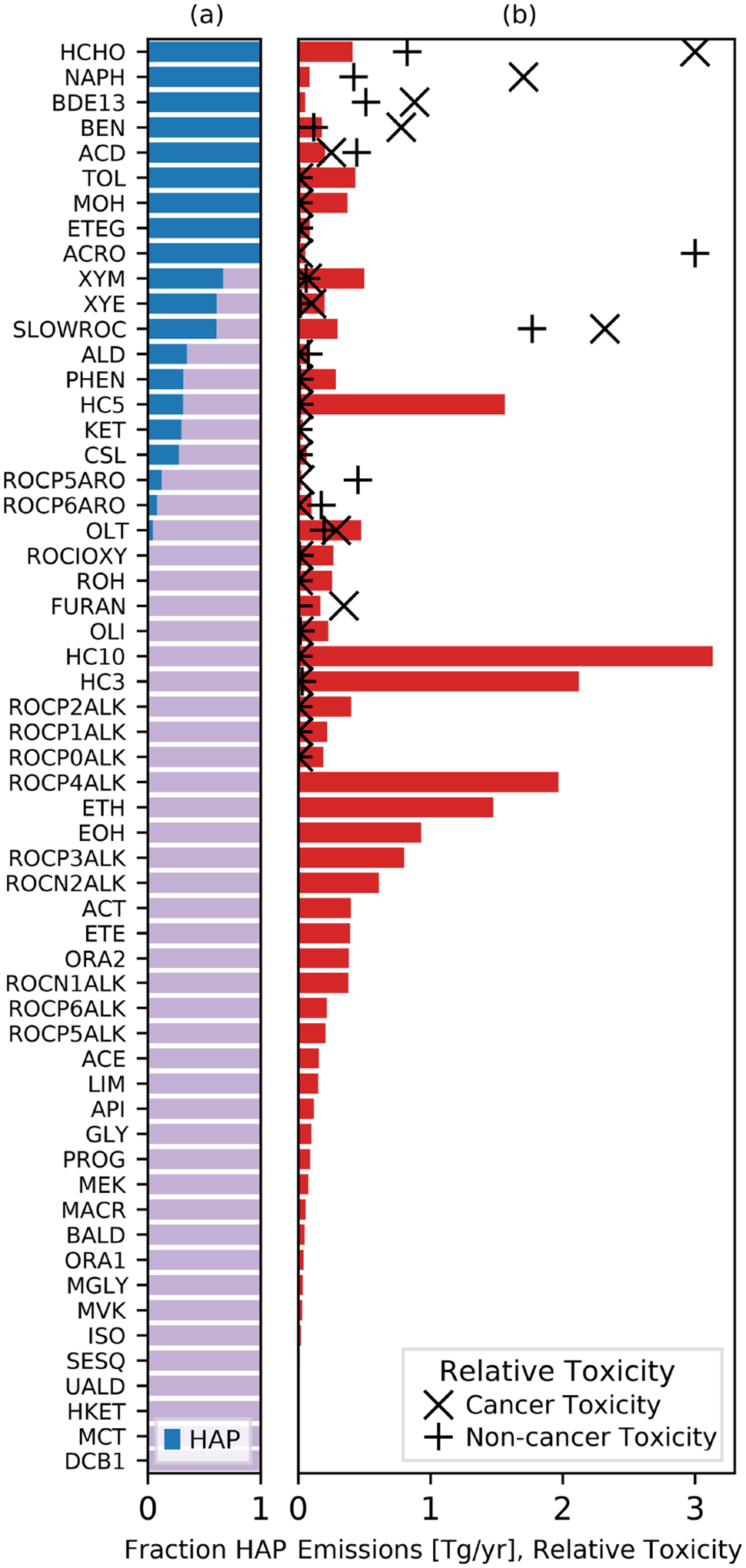
Distribution of hazardous air pollutants (HAPs) across CRACMM emitted species. Panel (**a**) indicates the mass fraction of 2017 US anthropogenic and biomass burning ROC emissions by CRACMM species that are HAPs (blue). Panel (**b**) indicates the magnitude of emissions in teragrams per year by CRACMM species (bars) and the emission-weighted toxicity for cancer (×) or non-cancer (+) health effects. Cancer and non-cancer toxicity are normalized for purposes of display such that the species with the maximum value in each category is 3. Health risks are only shown for CRACMM species that contain non-zero emissions of HAPs. These data are available in the Supplement as Table D3 (Pye, 2022).

Nine species in CRACMM cover 50 % of the total cancer and 60 % of the total non-cancer emission-weighted toxicity estimated for the anthropogenic and biomass burning emissions for 2017 US conditions (Fig. 6a: ACD, ETEG, ACRO, TOL, NAPH, MOH, HCHO, BDE13, and BEN). Toluene (chemistry in Sect. 3.5) is now separated from other aromatics and explicit due to its role as a HAP and significant emissions on an individual basis ($430\;\text{Gg}\;{\text{yr}}^{-1}$ in 2017, Fig. 6b) as well as to facilitate comparison with routine measurements. Ethylene glycol, toluene, and methanol are, however, not particularly strong drivers of cancer and non-cancer inhalation toxicity risk potential (Fig. 6b). NAPH (chemistry in Sect. 3.5), ACRO (chemistry in Sect. 3.9), and BDE13 (chemistry in Sect.3.9) are new mechanism species and are estimated to carry significant emission-weighted toxicity (Scheffe et al., 2016) (Fig. 6b). NAPH emissions are dominated by naphthalene (74 %) but include POM as well, making it an aggregate of HAPs. Naphthalene alone accounts for 70 % of the cancer and 98 % of the non-cancer emission-weighted toxicity of NAPH. In the case of ACRO, significant secondary production (not shown in Fig. 6b) is expected, and acrolein has been previously shown to be the largest contributor to non-cancer inhalation risk in the US (Scheffe et al., 2016). Given acetaldehyde and formaldehyde are also produced by oxidation of biogenic and anthropogenic emissions, the actual coverage of toxicity by the nine major HAP species is likely much higher than estimated based on the emissions alone. Previous work including secondary production estimated that acetaldehyde, benzene, formaldehyde, methanol, acrolein, 1,3-butadiene, and naphthalene represented over 84 % of the cancer risk and 93 % of the non-cancer respiratory risk effects in the US in 2011 (Scheffe et al., 2016).

The lumped, slowly reacting ROC (SLOWROC, Sect. 3.1) is 61 % HAP by mass with enough emission-weighted toxicity to make it the second leading contributor to cancer and non-cancer health risk potential out of all CRACMM species (Fig. 6b). Species within SLOWROC have a lifetime against chemical reaction of about 1 month and are typically discarded from chemical transport model calculations for that reason. SLOWROC includes ethylene oxide and 1,2-dibromoethane, among many other species, that individually contribute to high levels of potential cancer risk (2nd and 10th highest emission-weighted toxicity out of all 188 individual HAPs in this work). Hydrogen cyanide is the most abundant individual species in SLOWROC and is the second largest contributor to non-cancer health risk potential for all HAPs considered. In standard CRACMM applications, SLOWROC concentrations could be used to indicate areas warranting additional investigation, but individual compound tracers would be required for studies specifically addressing the health impacts of these longer-lived pollutants. In CMAQv5.4, additional individual HAPs needed for air toxic assessments (e.g., Scheffe et al., 2016) can be added to a chemical mechanism as tracers with reactive decay.

In total, 29 ROC species in CRACMM contain some amount of HAP emissions (Fig. 6a). In terms of species with significant HAP emissions by mass, the two lumped, single-ring aromatic hydrocarbon categories (XYE and XYM) are 61 % and 67 % HAP by mass, with ethylbenzene (in XYE) and indene (in XYM) being the largest contributors to cancer toxicity and m-xylene (in XYM) and o-xylene (in XYE) being the largest contributors to non-cancer toxicity potential. The gas-phase chemistry of XYE is based on ethylbenzene (Sect. 3.5), so XYE could become an explicit HAP in CRACMM with changes only to emission mapping (redirecting single-ring species in XYE other than ethylbenzene to XYM). The two aromatic IVOCs are about 10 % HAP by emitted mass, with 2,4-toluene diisocyanate (ROCP5ARO) and aniline (ROCP6ARO) being the largest HAP contributors by mass as well as in terms of non-cancer health risk potential (5th and 10th out of 188 species). ALD (35 % HAP) includes the HAP propionaldehyde. OLT (5 % HAP by mass) includes acrylonitrile resulting in moderate cancer and non-cancer toxicity potential. Despite the low contributions by mass of HAPs to FURAN, FURAN shows moderate contributions to cancer potential due to the inclusion of chloroprene.

HAPs added in CRACMM provide greater explicit coverage of species contributing to chronic inhalation health risks, and many of the species classified as HAPs also contribute substantially to criteria pollutant formation. In total, HAPs are estimated to account for about 8 % of the total OA formation potential for 2017 US anthropogenic and biomass burning emissions (using SAR methods from Sect. 2.1). HAPs, with major contributors being formaldehyde, toluene, acetaldehyde, m-xylene, 1,3-butadiene, ethylbenzene, o-xylene, acrolein, ethylene glycol, and phenol, are predicted to contribute 31 % of the O_3_ formation potential for 2017 US anthropogenic and biomass burning emissions. Based on their potential for emission-weighted cancer toxicity (C), non-cancer toxicity (N), and O_3_ formation potential (O), priority HAPs to consider for purposes of protecting public health are the following: formaldehyde (CNO), ethylene oxide (C), naphthalene (C), 1,3-butadiene (CN), benzene (C), acrolein (N), hydrogen cyanide (N), toluene 2,4-diisocyanate (N), acetaldehyde (O), toluene (O), m-xylene (O), and methanol (O).

## 5 Implications for the chemical evolution of ROC

**Figure 7.**
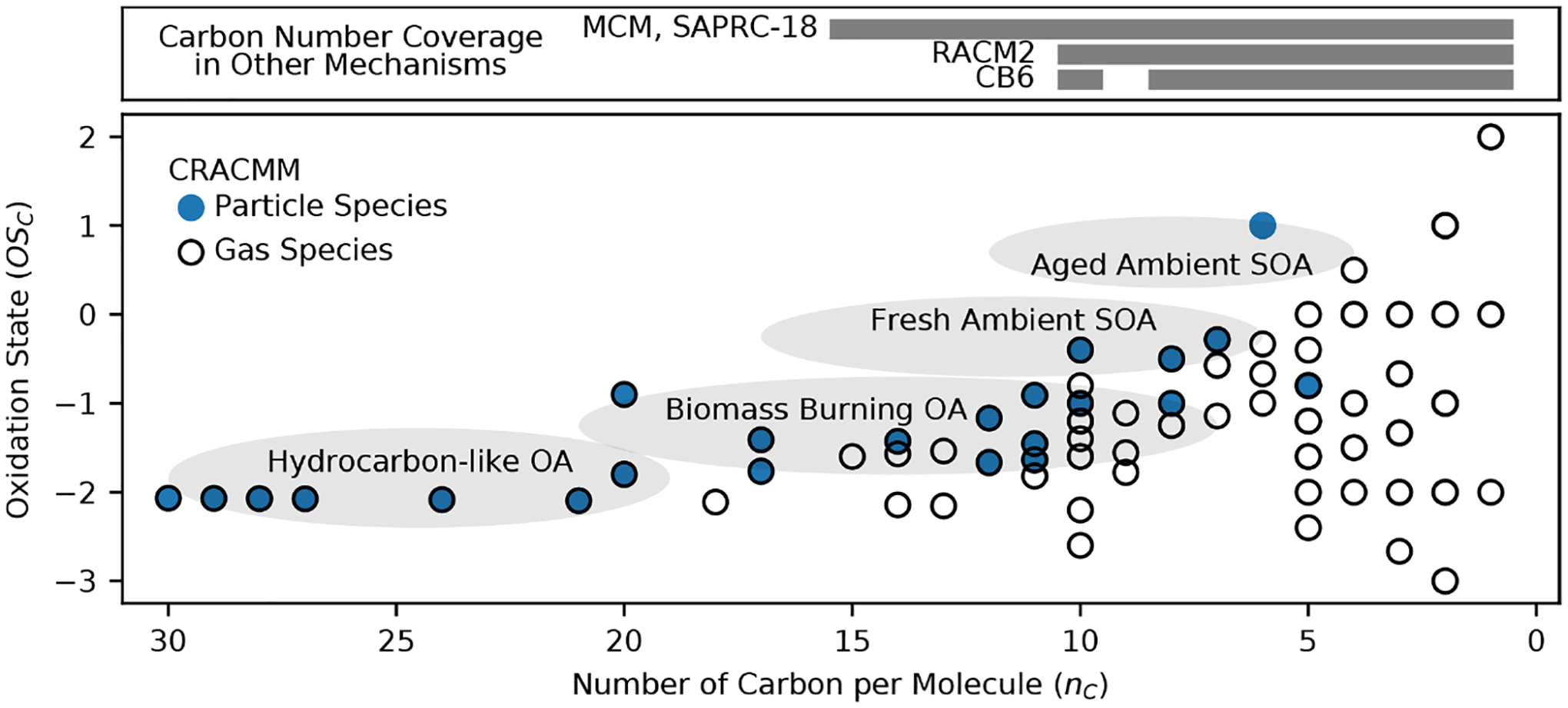
Mean carbon oxidation state (OS_C_) and number of carbon atoms per molecule $\left(n_{\text{C}}\right)$ for all stable ROC species. Filled circles indicate at least one particulate species present in CRACMM. Black circles indicate the presence of at least one gas species in CRACMM. Grey ellipses indicate approximate ranges of observation-based bulk OS_C_ and $n_{\text{C}}$ from the work by Kroll et al. (2011) for hydrocarbon-like OA (vehicle emissions and ambient hydrocarbon-like organic aerosol), biomass burning OA, fresh ambient SOA, and aged ambient SOA. Grey bars indicate $n_{\text{C}}$ coverage in mechanisms other than CRACMM.

In this section, CRACMM ROC species are visualized in terms of the carbon oxidation state and degree of oxygenation to understand if there are critical gaps in the atmospheric representation of ROC. The mean carbon oxidation state (OS_C_) of a species increases upon oxidation, and compounds generally move towards lower $n_{\text{C}}$ and higher OS_C_ as they are chemically processed in the atmosphere (Kroll et al., 2011). This view emphasizes SOA as a chemical intermediate on the path toward smaller and more functionalized compounds with carbon dioxide (OS_C_ = 4) as the ultimate endpoint. Using the CRACMM representative structures (Appendix A), each stable ROC species was plotted in the OS_C_ vs. $n_{\text{C}}$ space (Fig. 7) using the OS_C_ definition of Kroll et al. (2011) considering the number of carbons, hydrogens $\left(n_{\text{H}}\right)$, and oxygens ($n_{\text{O}}$) per molecule and expanded to include nitrogen $\left(n_{\text{N}}\right)$ and sulfur ($n_{\text{S}}$) (assuming sulfate and nitrate functionality) as follows:

(1) $${\text{OS}}_{\text{C}}=2\times n_{\text{O}}:n_{\text{C}}-n_{\text{H}}:n_{\text{C}}-5\times n_{\text{N}}:n_{\text{C}}-6\times n_{\text{S}}:n_{\text{C}}.$$

CRACMM species cover the atmospherically relevant range of ROC oxidation state and $n_{\text{C}}$ (Fig. 7). The largest $n_{\text{C}}$ species in CRACMM are alkane like with 20 to 30 carbons and a low-oxidation state consistent with observations of particulate vehicle exhaust and ambient hydrocarbon-like organic aerosol (Kroll et al., 2011). Other OA species in CRACMM generally fall in the range of $n_{\text{C}}$ and ${\text{OS}}_{\text{C}}$ reported for ambient observations of biomass burning organic aerosol, fresh ambient (less oxygenated) SOA, and aged (more oxygenated) ambient SOA. These ambient observations are based on bulk analysis (Kroll et al., 2011), and thus the observed ranges shown do not identify each possible SOA contributor at the molecular level. Monoterpene SOA monomers (AHOM) and dimers (AELHOM) have an oxidation state of −0.4 and −0.9, respectively, similar to laboratory data (Kroll et al., 2011). Monoterpene SOA has also been linked with the less oxidized (fresh ambient SOA) aerosol mass spectrometer (AMS) surrogate (Xu et al., 2018).

Two species in CRACMM, the glyoxal and methylglyoxal SOA from uptake in aqueous particles (AGLY) and clouds (AORGC), have overlap with the observed ambient aged SOA, which is often identified via positive matrix factorization analysis as a more oxidized oxygenated organic aerosol (MO-OOA) (Zhang et al., 2011). The MO-OOA factor has been linked to SOA from aqueous processing (Xu et al., 2017), and 10 % by mass of the MO-OOA in the southeastern US has been attributed to low-molecular-weight carboxylic acids, of which dicarboxylic acids are primarily from aqueous processing (Y. Chen et al., 2021). Aqueous isoprene SOA species such as isoprene-derived organosulfates and 2-methyltetrols ($n_{\text{C}}=5$) match properties of known major isoprene SOA constituents (Kroll et al., 2011; Surratt et al., 2010), and aqueous isoprene SOA (not shown in Fig. 7) is often resolved separately from MO-OOA. If the aged SOA region described by MO-OOA does represent an intermediate through which significant amounts of carbon should pass, additional chemical pathways beyond those from glyoxal and methylglyoxal may be needed in CRACMM.

Other mechanisms besides CRACMM (top of Fig. 7) focus on the more volatile range of ROC. MCM and SAPRC-18 include a sesquiterpene species with 15 carbons but otherwise focus on smaller-carbon-number species. The range in $n_{\text{C}}$ for alkane-like species in current mechanisms was highlighted in Sect. 3.1 and never exceeds 12. In terms of aromatics, the largest aromatic in MCM is a C_11_ diethyltoluene. SAPRC-18 includes some naphthalene-like species with 12 carbons, and RACM2 represents single-ring aromatics with ~9 carbons (Fig. 1, XYM). CB6 has a xylene species with 8 carbons, and RACM2 and CB6 both include monoterpenes as their largest species by $n_{\text{C}}$. CRACMM S/IVOCs with alkane, aromatic, and oxygenated structures populate the higher-carbonnumber ($n_{\text{C}}>10$) space that includes known organic aerosol species as well as precursors with high SOA yields and is not covered by current mechanisms due to their focus on gas-phase endpoints.

**Figure 8.**
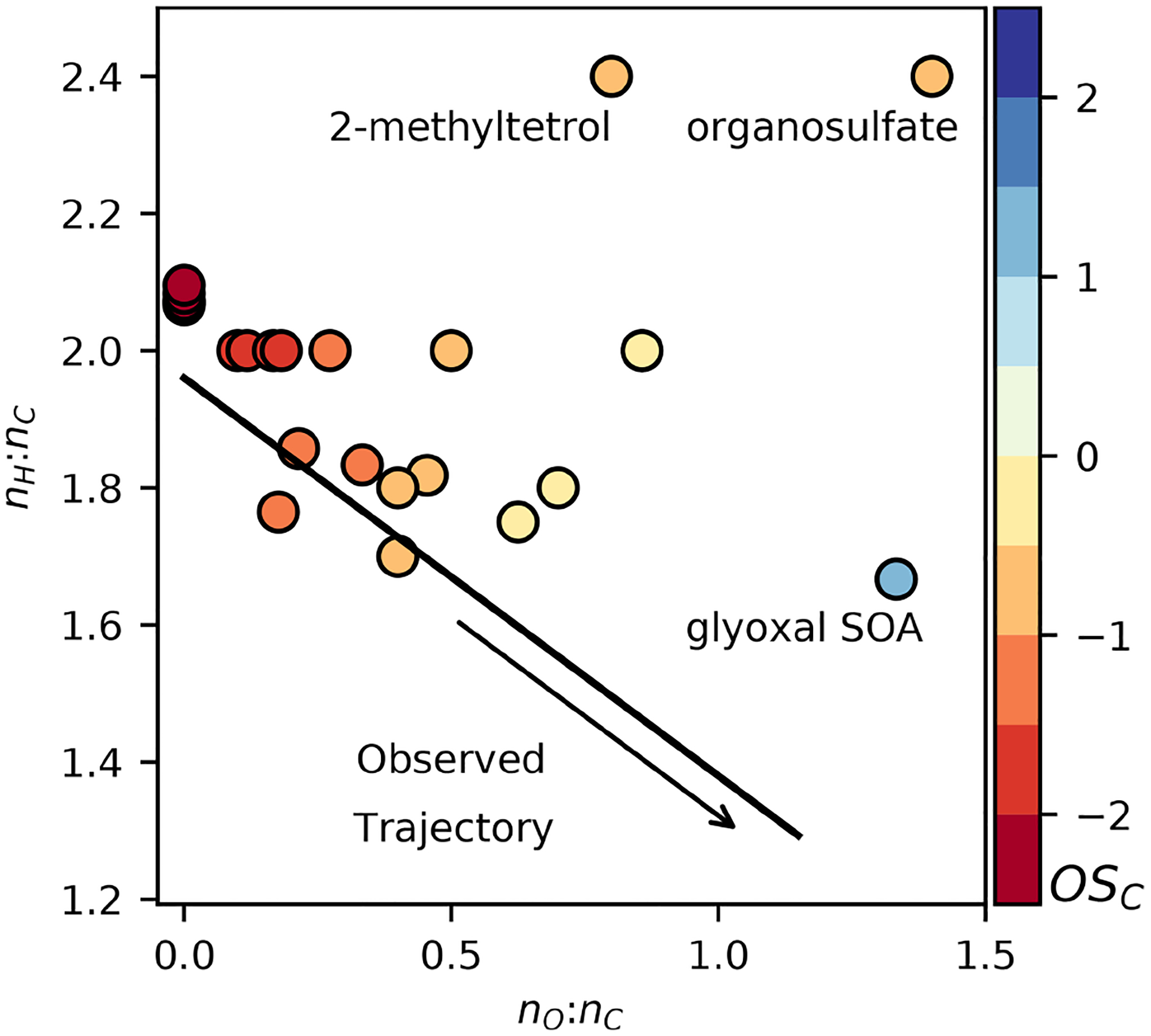
Molar ratios of hydrogen to carbon $\left(n_{\text{H}}:n_{\text{C}}\right)$ and oxygen to carbon ($n_{\text{O}}:n_{\text{C}}$) of CRACMM particulate ROC species. Color indicates the mean carbon oxidation state (OS_C_). The observed trajectory trend line with a slope of −0.6 is based on ambient measurements assembled by Chen et al. (2015) and extended to laboratory systems with $n_{\text{O}}:n_{\text{C}}$ near 0. Three CRACMM species are labeled glyoxal SOA (AGLY), isoprene-derived organosulfates (AISO3OS), and non-sulfated isoprene SOA represented as 2-methyltetrols (AISO3NOS).

As a complement to OS_C_, van Krevelen diagrams of $n_{\text{H}}:n_{\text{C}}$ vs. $n_{\text{O}}:n_{\text{C}}$ for individual and bulk species have been used to provide insight into the evolution of ambient organic aerosol (Heald et al., 2010). Since hydrogen and oxygen are generally the most abundant non-carbon elements in organic aerosol, these diagrams can help identify types of chemical functionalization. Primary emissions, particularly for alkane-like sources like vehicles tend to reside near an $n_{\text{H}}:n_{\text{C}}$ of 2 and $n_{\text{O}}:n_{\text{C}}$ of 0. Atmospheric processing generally moves OA towards higher $n_{\text{O}}:n_{\text{C}}$ and lower $n_{\text{H}}:n_{\text{C}}$ with the trajectory determined by the abundance of alcohol and peroxide (slope of 0) vs. ketone and aldehyde (slope of −2) groups (Heald et al., 2010). Mean atmospheric transformation of OA has been observed to occur along a slope of −0.5 (Ng et al., 2011) to −0.6 (Chen et al., 2015), which reflects either carboxylic acids or a combination of alcohols, peroxides, ketones, and aldehydes. Figure 8 (black line) shows the observed trend and range in $n_{\text{O}}:n_{\text{C}}$ from the ambient atmosphere from multiple field campaigns extended to an $n_{\text{O}}:n_{\text{C}}$ of 0 for primary source measurements.

The 26 individual particulate organic species in CRACMM span the full range of observed $n_{\text{O}}:n_{\text{C}}$ in bulk OA with excellent coverage for $n_{\text{O}}:n_{\text{C}}<0.5$ (Fig. 8). The highest observed $n_{\text{O}}:n_{\text{C}}$ conditions (~ 1.2) were only present in remote regions sampled by aircraft as described in the work by Chen et al. (2015). While CRACMM includes species with high $n_{\text{O}}:n_{\text{C}}$, those species (glyoxal SOA, isoprene organosulfate SOA, and non-sulfated isoprene SOA) tend to have much higher $n_{\text{H}}:n_{\text{C}}$ than the ambient trend suggests. Note that $n_{\text{O}}:n_{\text{C}}$ based on measurement techniques may not include all the oxygen in organosulfate compounds and oxidation state is likely a more robust way to measure degree of oxidation than $n_{\text{O}}:n_{\text{C}}$ based on techniques like use of an AMS (Canagaratna et al., 2015). Particularly for the $n_{\text{O}}:n_{\text{C}}>0.5$ OA species, CRACMM indicates more hydrogen than ambient observations suggest. If the ambient observations are correct, future versions of CRACMM could resolve the overestimate in $n_{\text{H}}:n_{\text{C}}$ by the following: (1) shifting the representative compound structures (for species like ROCN2OXY8) to reflect more ketones; (2) adjusting the assumed change in volatility per oxygen in the secondary oxygenated chemistry (Sect. 3.2); and/or (3) adding more chemical channels resulting in condensible ketones; carboxylic acids; or other $\text{high-}n_{\text{O}}:n_{\text{C}}$, $\text{low-}n_{\text{H}}:n_{\text{C}}$ products (e.g., photolysis of SOA, Baboomian et al., 2020). Combined with the information from the oxidation state plot (Fig. 7), CRACMM may need SOA species that are both lower in H and higher in O and at smaller carbon numbers with implications for aerosol hygroscopicity and mass (Pye et al., 2017).

Chen et al. (2015) noted that SOA produced in laboratory experiments was generally too low in $n_{\text{H}}:n_{\text{C}}$ at a given $n_{\text{O}}:n_{\text{C}}$ and tended to reside below the black ambient line in Fig. 8. CRACMM species are above the ambient trend line, suggesting that our conceptual picture of atmospheric processing to SOA, informed by known gas-phase chemistry and 2-D VBS approaches, does not match what is observed in laboratory experiments. One possible reason is the preferential sampling of certain chemical space in laboratory experiments (Porter et al., 2021).

**Figure 9.**
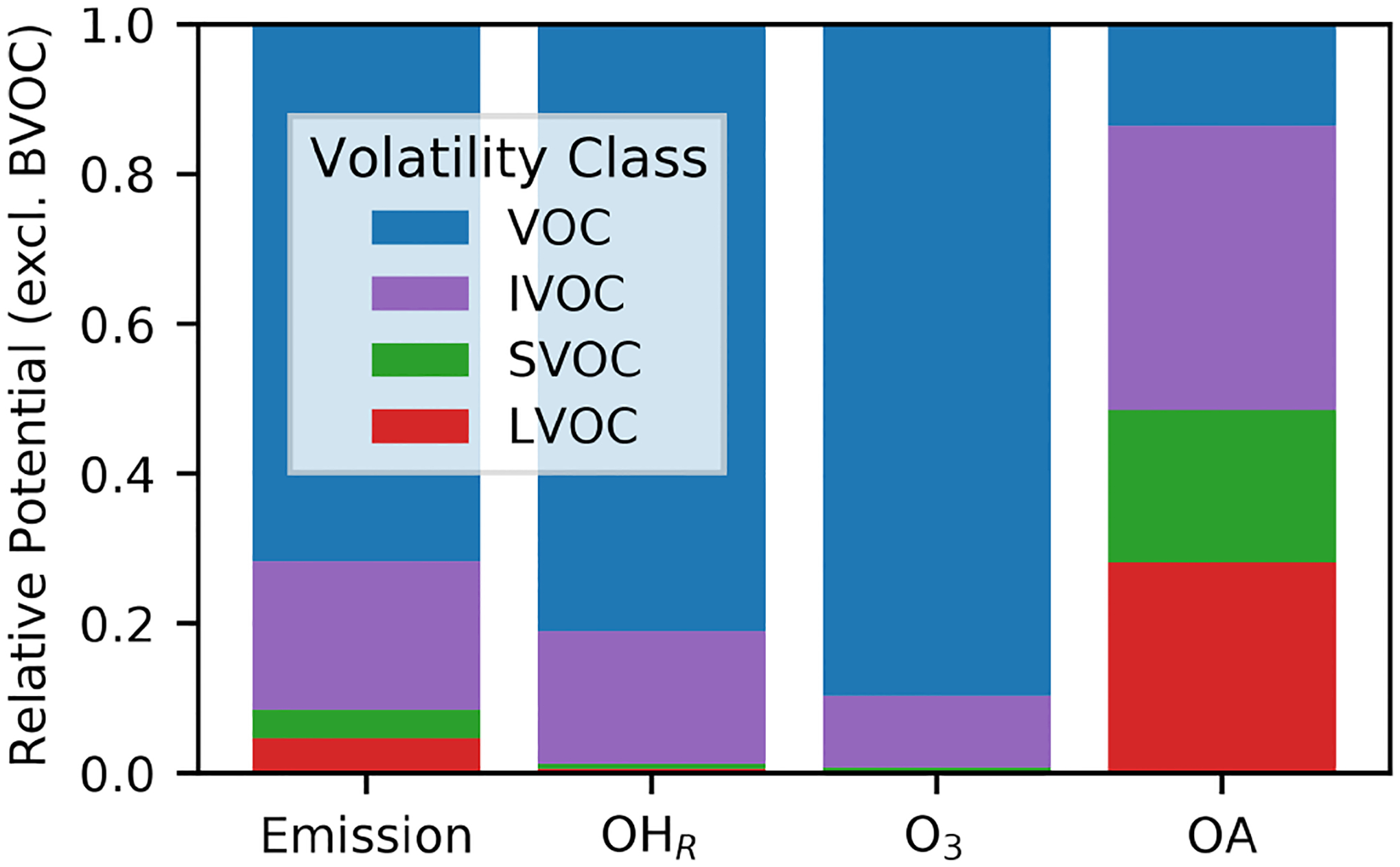
Anthropogenic and wood-burning ROC emissions and their relative potential HO reactivity (OH_R_), ozone (O_3_) formation, and OA for 2017 US conditions by volatility class. Biogenic VOCs (BVOCs) are not considered here. Ozone and OA formation potentials are calculated using the MIR and OA simple-SAR approaches from Sect. 2.1. Metrics are aggregated from the individual-species level to the following volatility classes: low-volatility organic compounds (LVOCs), semivolatile organic compounds (SVOCs), intermediate-volatility organic compounds (IVOCs), and volatile organic compounds (VOCs).

Figures 7 and 8 suggest that chemistry leading to OA needs to be considered in mechanism development to obtain an accurate representation of gas and particulate ROC including the correct properties of OA. Accurate properties of OA are critical for estimating hygroscopicity with implications for climate (Haywood and Boucher, 2000) as well as fine-particle mass (Pye et al., 2017). The linkages between gas and particulate endpoints are further emphasized by examining emissions from anthropogenic and biomass burning sources of ROC by volatility class and their propagation to endpoints (Fig. 9). Total emissions of ROC in 2017 (excluding biogenic VOCs) are estimated at $21\;\text{Tg}\;{\text{yr}}^{-1}$, with VOCs as the most abundantly emitted volatility class of compounds. VOCs dominate ROC HO reactivity, accounting for 81 % of the total. In addition, the total US O_3_ formation potential is estimated as $47\;\text{Tg}\;{\text{yr}}^{-1}$, with VOCs accounting for 90 % of it (based on the MIR SAR, Fig. 9). Thus, across all anthropogenic and biomass burning sources and locations for 2017, VOCs are the dominant contributors to gas-phase endpoints such as HO reactivity and O_3_; however, emitted IVOCs (generally excluded from mechanism development) make appreciable contributions to estimated gas-phase endpoints (18 % of HO reactivity and 10 % of the O_3_ formation potential). As a class, the O_3_ from IVOCs (about $4.5\;\text{Tg}\;{\text{yr}}^{-1}$) exceeds the O_3_ estimated for any individual CRACMM species in Fig. 1. In terms of effective MIR, IVOCs (effective MIR of $1.1\;\text{g}{\text{O}}_{3}\;{\text{g}}^{-1}$ ROC) are comparable to HC 10 and exceed that of BEN, HC3, and ETH. L/SVOCs are not substantial contributors to HO reactivity or O_3_ formation (~1%) due to slower reaction rates ($k_{\text{OH}}$, Fig. 3) and alkane-like structures with less potential for O_3_ formation (effective MIR of 0.14 to $0.27\;\text{g}{\text{O}}_{3}\;{\text{g}}^{-1}$ ROC). The OA potential from ROC emissions in the US (excluding biogenic emissions) is estimated as $5\;\text{Tg}\;{\text{yr}}^{-1}$ and emphasizes the need to consider L/S/IVOCs. Traditional VOCs (effective SOA yield of 5 %) are important (14 % of total) contributors to OA potential, but OA potential is dominated by IVOCs (38 %) and S/IVOCs (48 %) due to their initially lower volatility and ability to become condensible with only small additions in functionality.

## 6 Discussion

CRACMM provides an integrated approach to the representation of O_3_, organic aerosol, and many HAPs in air. These endpoints are linked as O_3_, SOA, and secondary HAPs such as formaldehyde and acrolein are products of gas-phase precursor emissions including primary HAPs. This section highlights reasons why mechanism development remains important and provides specific recommendations for future work based on lessons from CRACMM development.

First, the magnitude and compound identity of ROC emissions is an active area of research, and mechanisms need to interface with this emerging information. Improving emission characterization without the accompanying mechanism linkages hinders accurate source apportionment and effective air quality management decisions. Much of the work on emission speciation is identifying new species in the IVOC range, which has been historically neglected by gas-phase mechanisms but is necessary for both O_3_ and SOA prediction. Emission speciation work should continue to characterize source profiles in databases and other forums at the highest level of individual compound detail available using representative structures when necessary so that compounds can be easily mapped to mechanisms. In addition, efforts to accurately determine the emissions of individual HAPs, especially formaldehyde, acetaldehyde, toluene, m-xylene, and methanol, which are important for O_3_, should be leveraged in the preparation of emission inputs for regional chemical transport models even when HAPs are not the primary objective. The development of emissions and mechanisms should continue to be an iterative process in which new measurement techniques better quantify and identify emissions resulting in new or refined mechanism species. Simultaneously, mechanisms can indicate which emitted species constitute a high priority to constrain due to their role in secondary pollutant formation or health impacts.

Second, current chemical transport model mechanisms do not characterize the full range of atmospheric ROC, and such analysis could help identify missing sources of SOA, HO reactivity, formaldehyde, and other secondary HAPs. The ability to account for all reactive tropospheric carbon and perform a ROC budget analysis in current mechanisms is limited due to the focus on the more volatile range of ROC, which excludes lower-volatility primary ROC. In addition, some carbon in secondary ROC, including species in the volatile range, is discarded in mechanisms like SAPRC-07 and RACM2 because of product lumping for computational efficiency. For example, the largest organic peroxide in RACM2 is OP2 with two carbons. So, peroxides formed from RO_2_ + HO_2_ reactions for xylene-like aromatics $\left(n_{\text{C}}=9\right)$ result in a loss of seven carbons per reaction. In the RACM2 monoterpene system, eight carbons or 80 % of the parent carbon is lost each time a peroxide is formed, and SAPRC-07 loses four carbons for each monoterpene peroxide formed. While conservation of emitted mass is a priority in the design of CRACMM and more secondary mechanism species were added at the higher carbon numbers (e.g., a C_8_ and C_10_ peroxide), the chemical scheme in CRACMM is like RACM2 and SAPRC-07 in that it does not conserve mass upon reaction for all chemical systems. However, by curating structural identifiers (SMILES) for all species in CRACMM, conservation of carbon can now be calculated, and the importance of lost (or gained) carbon can be examined. The CMAQv5.4 implementation of CRACMM includes an updated chemical mechanism processor that creates an optional diagnostic file containing the elemental balance for each CRACMM reaction. Future work will aim to calculate mass balance across the mechanism and use it as a diagnostic tool to guide development.

Third, current gas-phase mechanisms do not couple radical chemistry with SOA formation, and linking the development provides additional constraints for ozone-forming reactions as well as secondary inorganic aerosol production. Particles and ozone are inherently linked systems (Ivatt et al., 2022; Womack et al., 2019). Molar yields for SOA are often comparable to molar yields of existing gas-phase product channels, and SOA mass should be removed from volatile gas-phase products. Properly sequestering products like peroxides in the particle will remove them as a potential photolytic source of radicals that releases ${\text{HO}}_{x}$ back to the atmosphere. Similarly, sequestering one organic nitrate in the particle phase could remove one ${\text{HO}}_{x}$ and one NO from the gas-phase system. Autoxidation, implemented in CRACMM primarily to produce SOA, effectively sequesters radicals since they are generally of sufficiently low volatility to condense. CRACMMv1.0 targeted SOA systems for development, but CRACMM updates impact O_3_ is demonstrated for the northeastern US in companion work (Place et al., 2023). Future versions of CRACMM should continue to consider chemical channels that lead to both gas-phase and particulate products to better constrain O_3_.

Fourth, linking gas-phase chemistry with SOA formation for the first time enabled the treatment of new SOA precursors with implications for the magnitude and source attribution of OA. Organic aerosol is dynamic with properties that evolve as a function of the precursor and chemical regime and need to be considered part of a holistic treatment of atmospheric chemistry. The interconnected nature of aromatic, phenolic, and furan systems highlights why mechanism development should consider SOA production alongside gas-phase chemistry. Developing phenolic and furanone gas-phase chemistry without consideration of SOA (as in CMAQv5.3.3) neglects a significant SOA source. Specifying SOA yields for phenolic and aromatic hydrocarbon precursors without recognizing they are also secondary would duplicate SOA mass. As a result, both phenolic and non-phenolic routes to SOA need to be specified consistently. The attribution of aromatic SOA to these two routes will affect how much SOA is predicted overall and how it is attributed to various sources. In the case of benzene SOA, the more SOA comes from phenol vs. non-phenol channels, the higher the total SOA potential of US emissions (as phenol > benzene emissions) and larger the attribution to sources with high ratios of phenol to benzene such as wildland fires and residential wood combustion. Previous work estimated oxidation of phenol, naphthalene, and benzene alone can account for 80 % of the SOA from residential wood combustion (Bruns et al., 2016). The importance of connecting SOA with multigenerational gas-phase chemistry also applies to the monoterpene system, where the fate of terpene nitrates and aldehydes will significantly modulate SOA formation. In the case of monoterpene SOA, the allocation of SOA between initial autoxidation, terpene nitrate, and aldehyde channels will affect the ${\text{NO}}_{x}$ dependence of total monoterpene SOA and therefore how much is considered controllable vs. non-controllable. The allocation of SOA among different later-generation species should continue to be evaluated and revised as new information becomes available which will improve source apportionment of fine-particle mass.

Fifth, new measurement techniques, observational studies, and computational methods are continually improving the characterization of many chemical systems, and their results need to be translated to model mechanisms. Autoxidation was determined to be an atmospherically relevant chemical pathway just under a decade ago (Crounse et al., 2013) and will be considered in CMAQ for the first time in CRACMMv1.0. Just this year, a new class of atmospherically relevant compounds, hydrotrioxides, were identified (Berndt et al., 2022). Even for traditional systems, information continues to emerge. For example, benzene mechanisms have been historically built on data that characterized about half of the product mass with recent work used to inform CRACMMv1.0 reaching ~ 80 % carbon closure (Xu et al., 2020). Measurement techniques and the availability of observational data will only further improve, providing more complete data to design and evaluate mechanisms going forward.

Finally, the chemistry of the atmosphere in the US and elsewhere is changing, and previously acceptable representations of chemistry may need modification. Autoxidation is one example of a pathway likely to grow in importance, but indications of change can be seen in multiple systems. Deposition of nitrogen has shifted from primarily oxidized nitrogen (nitrate) to reduced nitrogen (ammonia) (Li et al., 2016).

Fine-particle mass is no longer dominated by summertime sulfate (Chan et al., 2018), and the temperature dependence of summertime urban northeastern US PM_2.5_ is now being modulated by organic aerosol (Vannucci and Cohen, 2022). Particulate sulfur is also becoming increasingly recognized as organic (Riva et al., 2019; Moch et al., 2018). At the same time that sulfate and nitrate in cloud water have been decreasing at a mountaintop site in the northeastern US, total organic carbon in cloud water may be increasing (Lawrence et al., 2023). Organic compounds in air are changing with total US emissions of anthropogenic ROC going from ~ 30 % lower than ${\text{NO}}_{x}$ in 2002 to exceeding ${\text{NO}}_{x}$ by ~ 40 % in 2019 (Pye et al., 2022). The composition of ROC is also changing to more oxygenated forms, resulting in an average reduction in the O_3_ formation potential of an individual VOC of about 20 % due to mixture effects (Venecek et al., 2018). Questions chemical transport modeling and mechanisms are being asked to answer are also changing with increasing interest in wildland fires (McClure and Jaffe, 2018), volatile chemical products (Seltzer et al., 2022), and per- and polyfluoroalkyl substances (D’Ambro et al., 2021) among others. Changes in air pollution sources and questions of interest as well as chemical regimes over time require continued mechanism development, and CRACMM is now available as a community framework for further development.

## Supplementary Material

Supplement1

## Figures and Tables

**Table 1.** Pathways to SOA in CRACMM by system. Some systems include a representation of autoxidation (Auto?: Yes). Actual SOA formation in CRACMM is modulated by the oxidant concentration (HO, NO_3_, O_3_), RO_2_ bimolecular fate (NO/HO_2_), bimolecular RO_2_ lifetime $\left(\tau_{{\text{RO}}_{2}}\right)$, abundance of the partitioning medium (OA), photolysis (hν), and/or aqueous environment (see heterogeneous reactions in Appendix B). When autoxidation is represented but $\tau_{{\text{RO}}_{2}}$ is not listed here, autoxidation is assumed to be sufficiently fast so that it is not modulated by ambient conditions. SOA is modulated by temperature through gas-phase reaction rates and the effect of temperature on volatility (not explicitly listed). For estimated yield calculations, typical population-weighted values (Porter et al., 2021) of the bimolecular RO_2_ fate (rates of RO_2_ + HO_2_ and RO_2_+NO), the bimolecular lifetime (10 s), and the amount of organic partitioning medium ($10\;\mu \text{g}\;{\text{m}}^{-3}$) are assumed (if applicable). Estimated yields exclude multigenerational oxidation of secondary oxygenated ROC species unless explicitly mentioned. Species names and abbreviations can be found in Appendices A and B. L/S/IVOC: LVOC, SVOC, and/or IVOC.

System	Precursor	Main SOA species	Scientific basis	Auto?	Factors affecting SOA	Est. yield (mole frac.)	Est. yield (mass frac.)
Alkane-like systems (Sect. 3.1)							
~C27 SVOCs^a,b^	ROCP1ALK	Secondary oxygenated L/S/IVOCs	GECKO (Lannuque et al., 2018) and literature (Praske et al., 2018; Vereecken and Nozière, 2020)	Yes	HO, HO_2_ */*NO, $\tau_{{\text{RO}}_{2}}$, OA	1.0	0.75
~C24 SVOCs^a,b^	ROCP2ALK	Secondary oxygenated L/S/IVOCs	GECKO (Lannuque et al., 2018) and literature (Praske et al., 2018; Vereecken and Nozière, 2020)	Yes	HO, HO_2_ */*NO, $\tau_{{\text{RO}}_{2}}$, OA	0.98	0.87
~C21 IVOCs^a,b^	ROCP3ALK	Secondary oxygenated L/S/IVOCs	GECKO (Lannuque et al., 2018) and literature (Praske et al., 2018; Vereecken and Nozière, 2020)	Yes	HO, HO_2_ */*NO, $\tau_{{\text{RO}}_{2}}$, OA	0.86	0.72
~C18 IVOCs^a^	ROCP4ALK	Secondary oxygenated L/S/IVOCs	GECKO (Lannuque et al., 2018) and literature (Praske et al., 2018; Vereecken and Nozière, 2020)	Yes	HO, HO_2_ */*NO, $\tau_{{\text{RO}}_{2}}$, OA	0.48	0.51
~C14 IVOCs^a^	ROCP5ALK	Secondary oxygenated L/S/IVOCs	GECKO (Lannuque et al., 2018) and literature (Praske et al., 2018; Vereecken and Nozière, 2020)	Yes	HO, HO_2_ */*NO, $\tau_{{\text{RO}}_{2}}$, OA	0.13	0.15
~C12 IVOCs^a^	ROCP6ALK	Secondary oxygenated L/S/IVOCs	GECKO (Lannuque et al., 2018) and literature (Praske et al., 2018; Vereecken and Nozière, 2020)	Yes	HO, HO_2_ */*NO, $\tau_{{\text{RO}}_{2}}$, OA	0.040	0.043
~C10 VOCs	HC10	Secondary oxygenated L/S/IVOCs	GECKO (Lannuque et al., 2018) and literature (Praske et al., 2018; Vereecken and Nozière, 2020)	Yes	HO, HO_2_ */*NO, $\tau_{{\text{RO}}_{2}}$, OA	0.0059	0.0083
~C_5_ VOCs	HC5	ASOAT	Emission-based SAR	No	HO	0.0013	0.0037
~C_3_ VOCs	HC3	ASOAT	Emission-based SAR	No	HO	2.8 × 10^−5^	0.00013
Long-lived species^a^	SLOWROC	ASOAT	Emission-based SAR	No	HO	0.0010	0.0027
Oxygenated L/S/IVOCs (Sect. 3.2–3.3)							
Secondary oxygenated L/SVOCs^c^	ROCP0OXY02 ROCN1OXY06 ROCN1OXY03 ROCN1OXY01	Secondary oxygenated L/S/IVOCs	Multigenerational 2-D VBS	No	HO, OA	^d^	1.02–1.16^d^
Secondary oxygenated SVOCs^c^	ROCP1OXY01 ROCP0OXY04	Secondary oxygenated L/S/IVOCs	Multigenerational 2-D VBS	No	HO, OA	^d^	0.85–0.89^d^
Secondary oxygenated SVOCs^c^	ROCP2OXY02 ROCP1OXY03	Secondary oxygenated L/S/IVOCs	Multigenerational 2-D VBS	No	HO, OA	^d^	0.63–0.64^d^
Secondary oxygenated IVOCs^c^	ROCP3OXY02	Secondary oxygenated L/S/IVOCs	Multigenerational 2-D VBS	No	HO, OA	^d^	0.52^d^
Secondary oxygenated IVOCs^c^	ROCP4OXY02	Secondary oxygenated L/S/IVOCs	Multigenerational 2-D VBS	No	HO, OA	^d^	0.37^d^
Secondary oxygenated IVOCs^c^	ROCP5OXY01	Secondary oxygenated L/S/IVOCs	Multigenerational 2-D VBS	No	HO, OA	^d^	0.36^d^
Secondary oxygenated IVOCs^c^	ROCP6OXY01	Secondary oxygenated L/S/IVOCs	Multigenerational 2-D VBS	No	HO, OA	^d^	0.23^d^
Multifunctional ~C8 peroxides	OP3	AOP3	New lumped, semivolatile species; chemistry like RACM OP2	No	OA, *hν*, HO	0.50^e^	0.50^e^
Emitted oxygenated IVOCs^a^	VROCIOXY	ASOAT	Emission-based SAR	No	HO	0.15	0.12
Aromatics and furans (Sect. 3.4–3.5)							
Furanone^a^	FURANONE	ASOAT	Literature on furans (Bruns et al., 2016)	No	HO	0.040	0.080
Less volatile aromatic IVOCs^a^	ROCP5ARO	Secondary oxygenated L/S/IVOCs ASOAT	MCM (Bloss et al., 2005) and literature (Xu et al., 2020; Molteni et al., 2018)	Yes	HO, HO_2_, NO, OA	0.37^f^	0.47^f^
More volatile aromatic IVOCs^a^	ROCP6ARO	Secondary oxygenated L/S/IVOCs, ASOAT	MCM (Bloss et al., 2005) and literature (Xu et al., 2020; Molteni et al., 2018)	Yes	HO, HO_2_, NO, OA	0.21^f^	0.25^f^
Naphthalene and PAHs	NAPH	Secondary oxygenated L/S/IVOCs ASOAT	MCM (Bloss et al., 2005) and literature (Xu et al., 2020; Molteni et al., 2018)	Yes	HO, HO_2_, NO, OA	0.21^f^	0.34^f^
Benzene	BEN	AROCN1OXY6, ASOAT	MCM (Bloss et al., 2005) and literature (Xu et al., 2020; Molteni et al., 2018; Ng et al., 2007)	Yes	HO, HO_2_, NO, OA	0.18^f,g^	0.44^f,g^
Toluene	TOL	AROCN1OXY6, ASOAT	MCM (Bloss et al., 2005) and literature (Xu et al., 2020; Molteni et al., 2018; Ng et al., 2007)	Yes	HO, HO_2_, NO, OA	0.15f,g	0.33f,g
More reactive aromatic VOCs	XYM	AROCP0OXY4, ASOAT, AOP3	MCM (Bloss et al., 2005) and literature (Xu et al., 2020; Molteni et al., 2018; Ng et al., 2007)	Yes	HO, HO_2_, NO, OA	0.28f,g	0.54f,g
Less reactive aromatic VOCs	XYE	AROCP0OXY4, ASOAT, AOP3	MCM (Bloss et al., 2005) and literature (Xu et al., 2020; Molteni et al., 2018; Ng et al., 2007)	Yes	HO, HO_2_, NO, OA	0.28f,g	0.50f,g
Phenol and aromatic diols^a^	PHEN	ASOAT	Literature including benzene constraints (Bruns et al., 2016; Ng et al., 2007; Zhang et al., 2014)	No	HO	0.15	0.28
Cresols^a^	CSL	ASOAT	Literature including xylene and toluene constraints (Bruns et al., 2016; Ng et al., 2007; Zhang et al., 2014)	No	HO	0.20	0.29
Sesquiterpenes (Sect. 3.6) and monoterpenes (Sect. 3.7)							
Sesquiterpenes	SESQ	Secondary oxygenated L/S/IVOCs	MCM (Jenkin et al., 2012) and literature (Richters et al., 2016)	Yes	HO, NO_3_, O_3_, HO_2_, NO, OA	HO: 0.52, O_3_: 0.028, NO_3_: 0.46	HO: 0.60, O_3_: 0.034, NO_3_: 0.45
α-Pinene and similar	API	AHOM, AELHOM	Literature (Nozière et al., 1999; Berndt et al., 2016; Piletic and Kleindienst, 2022; Zhao et al., 2018; Jokinen et al., 2015)	Yes	HO, NO_3_, O_3_, HO_2_, NO	HO, NO_3_: 0.11,^h^ O_3_: 0.13^h^	HO, NO_3_: 0.21,^h^ O_3_: 0.24^h^
Limonene and similar	LIM	AHOM, AELHOM	Literature (Piletic and Kleindienst, 2022; Zhao et al., 2018; Jokinen et al., 2015)	Yes	HO, NO_3_, O_3_, HO_2_, NO	HO, NO_3_: 0.16,^h^ O_3_: 0.21^h^	HO, NO_3_: 0.30,^h^ O_3_: 0.38^h^
Pinonaldehyde^a^	PINAL	AHOM	MCM (Saunders et al., 2003) and RACM2 photolysis and assumed autoxidation	Yes	HO, $\tau_{{\text{RO}}_{2}}$	Phot: see HC10, HO: 0.21	HO: 0.31
Limonene-like aldehydes^a^	LIMAL	AHOM	MCM (Saunders et al., 2003) and RACM2 photolysis and assumed autoxidation	Yes	HO, O_3_, $\tau_{{\text{RO}}_{2}}$	Phot: see HC10, HO: 0.64, O_3_: <1%	HO: 0.95
Terpene peroxides	OPB	See HC10	New volatile biogenic peroxide; chemistry like RACM2 OP2	No	HO, *hν*	HO: <1%	–
Terpene nitrates	TRPN	AHOM	Literature (Zare et al., 2019)	No	HO, NO_3_, O_3_	1.0	1.16
Aqueous systems (Sect. 3.8)							
Isoprene epoxydiols	IEPOX	AISO_3_NOS, AISO_3_OS	CMAQ AERO_6_–7 (Pye et al., 2017, 2013)	No	Particle pH, liquid water, sulfate, size distribution	Variable	Variable
Glyoxal and methylglyoxal uptake to particles	GLY, MGLY	AGLY	CMAQ AERO_6_–7 (Pye et al., 2015)	No	Particle size distribution	Variable	Variable
Glyoxal and methylglyoxal uptake in clouds	GLY, MGLY	AORGC	CMAQ AERO_5_–7 (Carlton et al., 2008)	No	HO	Variable	Variable

a New SOA precursor system compared to CMAQ AERO6–7 (Appel et al., 2021).

b ROCN2ALK, ROCN1ALK, ROCP0ALK, ROCP1ALK, ROCP2ALK, and ROCP3ALK can partition directly to particles and form POA (see Sect. 3.1). Yields here are for chemical reaction.

c While these species are envisioned as secondary oxygenated semivolatile emissions, those from sources such as biomass burning could be mapped to this system based on volatility.

d Calculated for 12 h of reaction time across multiple generations. Only mass-based yields are provided. See Fig. 4.

e Based on semivolatile partitioning of OP3. Further reaction of OP3 with HO produces < 1 % molar yield of SOA.

f SOA yield includes furanone route contributions.

g SOA yield includes phenolic (PHEN or CSL) route contributions.

h SOA yield includes complete further reaction of TRPN but not aldehydes (PINAL or LIMAL).

## Data Availability

The EPA’s Chemicals Dashboard is available at https://comptox.epa.gov/dashboard (U.S. Environmental Protection Agency, 2021d). OPERA predictions of species properties can be obtained from the Chemicals Dashboard or for any species with a SMILES record using the EPA’s Chemical Transformation Simulator at https://qed.epa.gov/cts/ (U.S. Environmental Protection Agency, 2022f). SPECIATE is distributed at https://www.epa.gov/air-emissions-modeling/speciate (U.S. Environmental Protection Agency, 2022g). RDKit version 2020.09.01 was used in Python (RDKit, 2020). The implementation of RACM2–AERO6 is available in CMAQv5.3.3 (https://doi.org/10.5281/zenodo.3585898, U.S. Environmental Protection Agency Office of Research and Development, 2019). RACM2 and CRACMMv1.0 in CMAQv5.4 (released October 2022) are available on GitHub (https://github.com/USEPA/CMAQ, last access: 21 April 2023) and Zenodo (https://doi.org/10.5281/zenodo.7218076, U.S. EPA Office of Research and Development, 2022). Supporting data for CRACMM, including the SPECIATE database mapped to CRACMM, input to the Speciation Tool, profile files output from the Speciation Tool for input to SMOKE, Python code for mapping species to CRACMM, chemical mechanism, and mechanism metadata, are available at https://github.com/USEPA/CRACMM (last access: 21 April 2023). Specific analyses and scripts used in this paper such as the 2017 US species-level inventory and code for figures are archived at https://doi.org/10.23719/1527956 (Pye, 2022).

## Appendix A

**Table A1.** ROC species in CRACMM and their description, phase (Phs) in which they can exist (G: gas, P: particle), and SMILES for representative compound structure. Appendix A along with additional ROC species information is also available in csv format in the data archive associated with this work (Table D1; Pye, 2022). Species properties such as molecular weights are determined from the representative structure except in the case of highly empirical species (SLOWROC, VROCIOXY, ASOAT). In CMAQ, aerosol species reside in Aitken, accumulation, and/or coarse modes and are appended with the letter to indicate the size mode. Five non-volatile, organic aerosol species start with the letter A (AISO3NOS, AISO3OS, AORGC, ASOAT, and AGLY). Some gas-phase species inherited from RACM2 (all indicated here) start with an A. In all other cases, an prepended A in CMAQ indicates a particulate form of the species below. A prepended V (if present) will indicate a gas-phase species.

Species	Description	Phs	Representative compound
ACD	Acetaldehyde	G	CC=O
ACE	Acetylene	G	C#C
ACO3	Acetyl peroxy radicals	G	CC(=O)O[O]
ACRO	Acrolein	G	C=CC=O
ACT	Acetone	G	CC(C)=O
ACTP	Peroxy radicals formed from ACT	G	CC(=O)CO[O]
ADCN	Aromatic NO_3_ adduct from PHEN	G	OC1=C[C]C(O[N+]([O−])=O)C=C1
ADDC	Aromatic HO adduct from CSL	G	CC1=CC(O)=CC([O])C1
AGLY	SOA from reactive uptake of glyoxal on particles	P	OC2OC(C1OC(O)C(O)O1)OC2O
AISO3NOS	Non-sulfated SOA from IEPOX uptake	P	C(O)C(O)(C)C(O)CO
AISO3OS	Organosulfate SOA from IEPOX uptake	P	C(O)C(OS(O)(=O)(=O))(C)C(O)CO
ALD	C3 and higher aldehydes	G	CCC=O
AORGC	SOA from cloud processing of GLY and MGLY	P	OC2OC(C1OC(O)C(O)O1)OC2O
API	α-Pinenes and cyclic terpenes with one double bond	G	CC1=CCC2CC1C2(C)C
APINP1	Peroxy radicals from API + NO_3_ that do not undergo autoxidation	G	[O]OC1(C)C(ON(=O)=O)CC2CC1C2(C)C
APINP2	Peroxy radicals from API + NO_3_ that undergo autoxidation	G	[O]OC1(C)C(ON(=O)=O)CC2CC1C2(C)C
APIP1	Peroxy radicals from API + HO that do not undergo autoxidation	G	[O]OC1(C)C(O)CC2CC1C2(C)C
APIP2	Peroxy radicals from API + HO that undergo autoxidation	G	[O]OC1(C)C(O)CC2CC1C2(C)C
ASOAT	An empirical SOA	P	CC(=O)C(C(C(C(CO)O)O)O)O
BAL1	Peroxy radicals formed from BALD	G	[O]OC1=CC=C(C)C=C1
BAL2	Peroxy radicals formed from BALD	G	[O]OC1=CC=CC=C1
BALD	Benzaldehyde and other aromatic aldehydes	G	O=CC1=CC=CC=C1
BALP	Peroxy radicals formed from BALD	G	O=C(O[O])C1=CC=CC=C1
BDE13	1,3-Butadiene	G	C=CC=C
BDE13P	Peroxy radicals from BDE13	G	C=CC(O[O])CO
BEN	Benzene	G	C1=CC=CC=C1
BENP	Peroxy radicals formed from benzene	G	[O]OC1C=CC2OOC1C2O
CHO	Phenoxy radical formed from CSL	G	[O]C1C=C(C)C(O)C(=C1)C
CO	Carbon monoxide	G	[C−]#[O+]
CSL	Cresol and other hydroxy-substituted aromatics	G	CC(C)(O)C1=CC=CC=C1
DCB1	Unsaturated dicarbonyls	G	O=CC=C(C)C=O
DCB2	Unsaturated dicarbonyls	G	O=CC(=CC(=O)C)C
DCB3	Unsaturated dicarbonyls	G	O=CC=CC=O
ELHOM	Extremely low-volatility highly oxygenated molecules from terpenes	GP	OC1CC2C(OOC2(C)C)C(OOC3(C)C4C(C)(C)C(C4)CC3O)C1(C)OO
EOH	Ethanol	G	CCO
ETE	Ethene	G	C=C
ETEG	Ethylene glycol	G	OCCO
ETEP	Peroxy radicals formed from ETE	G	OCCO[O]
ETH	Ethane	G	CC
ETHP	Peroxy radicals formed from ethane and other species	G	CCO[O]
FURAN	Furans and other dienes	G	O=CC1=CC=CO1
FURANO2	Peroxy radicals from FURAN oxidation	G	OC1C=CC(O1)(O[O])(C=O)
FURANONE	Ring-retaining ketone product from FURAN oxidation	G	C1=CC(=O)OC1O
GLY	Glyoxal and glycoaldehydes	G	O=CC=O
HC10	Alkanes and other species with HO rate constant greater than 6.8 × 10^−12^ molec.cm^−3^ s^−1^	G	CCCCCCCCCC
HC10P	Peroxy radicals formed from HC10	G	CCCCCCCC(CC)O[O]
HC10P2	Hydroxy peroxy radicals from HC10P alkoxy product	G	CCCCC(O[O])CCC(O)CC
HC3	Alkanes and other species with HO rate constant less than 3.4 × 10^−12^ molec.cm^−3^ s^−1^	G	CCC
HC3P	Peroxy radicals formed from HC3	G	CC(C)O[O]
HC5	Alkanes and other species with HO rate constant between 3.4 × 10^−12^ and 6.8 × 10^−12^ molec.cm^−3^ s^−1^	G	CCCCC
HC5P	Peroxy radicals formed from HC5	G	CCC(O[O])CC
HCHO	Formaldehyde	G	C=O
HKET	Hydroxy ketone	G	CC(=O)CO
HOM	Highly oxygenated molecules from terpenes	GP	OC1CC2C(OOC2(C)C)C(OO)C1(C)OO
IEPOX	Isoprene epoxydiols	G	OCC1OC1(C)CO
ISHP	β-Hydroxyhydroperoxides from ISOP + HO_2_	G	C=CC(OO)(CO)C
ISO	Isoprene	G	CC(=C)C=C
ISON	β-Hydroxyalkylnitrates from ISOP + NO alkylnitrates from ISO + NO_3_	G	OCC(C)(C=C)ON(=O)=O
ISOP	Peroxy radicals formed from ISO + HO	G	OCC(O[O])C(C)=C
KET	Ketones	G	CCC(=O)CC
KETP	Peroxy radicals formed from KET	G	CCC(C(C)O[O])=O
LIM	Δ-Limonene and other cyclic diene terpenes	G	CC(=C)[C@@H]1CCC(C)=CC1
LIMAL	Limonene aldehyde and similar LIM-derived aldehydes	G	O=CCC(CCC(=O)C)C(=C)C
LIMALP	Peroxy radicals from LIMAL	G	O=CCC(CCC(=O)C)C(C)(CO)O[O]
LIMNP1	Peroxy radicals from LIM + NO_3_ that do not undergo autoxidation	G	[O−][N+](=O)OC1CC(CCC1(C)O[O])C(=C)C
LIMNP2	Peroxy radicals from LIM + NO_3_ that undergo autoxidation	G	[O−][N+](=O)OC1CC(CCC1(C)O[O])C(=C)C
LIMP1	Peroxy radicals from LIM + HO that do not undergo autoxidation	G	[O]OC1(C)CCC(CC1O)C(=C)C
LIMP2	Peroxy radicals from LIM + HO that undergo autoxidation	G	[O]OC1(C)CCC(CC1O)C(=C)C
MACP	Peroxy radicals formed from MACR + HO	G	CC(=C)C(=O)O[O]
MACR	Methacrolein and other C_4_ aldehydes	G	CC(=C)C=O
MAHP	Hydroperoxides from MACP + HO_2_	G	C=C(C)C(OO)=O
MCP	Peroxy radical formed from MACR + HO which does not form MPAN	G	OCC(C)(O[O])C=O
MCT	Methylcatechols and similar species	G	CC1=CC(O)=C(O)C=C1
MCTO	Alkoxy radical formed from MCT + HO and MCT + NO_3_	G	CC1=CC(O)=CC([O])=C1
MCTP	Radical formed from MCT + O_3_ reaction	G	CC(/C=C\[C](O[O])O)=C/C(O)=O
MEK	Methyl ethyl ketone	G	CCC(C)=O
MEKP	Peroxy radicals formed from MEK	G	[O]OCCC(=O)C
MGLY	Methylglyoxal and other α-carbonyl aldehydes	G	CC(=O)C=O
MO2	Methylperoxy radical	G	CO[O]
MOH	Methanol	G	CO
MPAN	Peroxymethacryloylnitrate and other higher peroxyacylnitrates from isoprene oxidation	G	O=N(=O)OOC(=O)C(=C)C
MVK	Methyl vinyl ketone	G	CC(=O)C=C
MVKP	Peroxy radicals formed from MVK	G	CC(=O)C(O)CO[O]
NALD	Nitrooxyacetaldehyde	G	O=CCON(=O)=O
NAPH	Naphthalene and other PAHs	G	C1=CC2=CC=CC=C2C=C1
NAPHP	Peroxy radicals from NAPH oxidation	G	C12=CC=CC=C1C3OOC(C3O[O])C2(O)
OLI	Internal alkenes	G	CC=C(C)C
OLIP	Peroxy radicals formed from OLI	G	[O]OC(C)(C)C(C)O
OLND	NO_3_-alkene adduct reacting via decomposition	G	CC(O[O])CO[N+]([O−])=O
OLNN	NO_3_-alkene adduct reacting to form carbonitrates + HO_2_	G	CC(O[O])CO[N+]([O−])=O
OLT	Terminal alkenes	G	CC=C
OLTP	Peroxy radicals formed from OLT	G	CC(CO)O[O]
ONIT	Organic nitrates	G	CCC(C)O[N+](=O)[O−]
OP1	Methyl hydrogen peroxide	G	COO
OP2	Higher organic peroxides	G	CCOO
OP3	Semivolatile organic peroxide	GP	CCC(=O)CC(OO)C(O)CC
OPB	Terpene-derived peroxides	G	OOC1(C)C(O)CC2CC1C2(C)C
ORA1	Formic acid	G	OC=O
ORA2	Acetic acid and higher acids	G	CC(O)=O
ORAP	Peroxy radical formed from ORA2 + HO reaction	G	[O]OCC(=O)O
PAA	Peroxyacetic acids and higher analogs	G	CC(=O)OO
PAN	Peroxyacetyl nitrate and more highly saturated PANs	G	CC(=O)OON(=O)=O
PHEN	Phenol and benzene diols	G	OC1=CC(O)=CC=C1
PINAL	Pinonaldehyde and similar API-derived aldehydes	G	O=CCC1CC(C(=O)C)C1(C)C
PINALP	Peroxy radicals from PINAL oxidation	G	O=CCC1(O[O])CC(C(=O)C)C1(C)C
PPN	Peroxypropionyl nitrate	G	CCC(=O)OO[N+](=O)[O−]
PROG	Propylene glycol and other three-carbon dialcohols	G	CC(O)CO
RCO3	Acyl peroxy radicals of carbon numbers of C_3_ and greater	G	CCC(=O)O[O]
VROCIOXY	Intermediate-volatility oxygenated ROC species (directly emitted)	G	C[Si]1(C)O[Si](C)(C)O[Si](C)(C)O[Si](C)(C)O[Si](C)(C)O1
ROCN1ALK	Alkane-like ROC, $C_{i}^{*}=10^{-1}\mu \text{g}\;{\text{m}}^{-3}$	GP	CCCCCCCCCCCCCCCCCCC(C)CCCC(C)CCCC
ROCN1OXY1	Oxygenated ROC, $C_{i}^{*}=10^{-1}\mu \text{g}\;{\text{m}}^{-3}$ and $n_{\text{O}}:n_{\text{C}}$ of 0.1	GP	CCCCCCCCCCCCCCCCCCCC(=O)O
ROCN1OXY3	Oxygenated ROC, $C_{i}^{*}=10^{-1}\mu \text{g}\;{\text{m}}^{-3}$ and $n_{\text{O}}:n_{\text{C}}$ of 0.3	GP	C(CCCCCC(=O)O)CCCCC(=O)O
ROCN1OXY6	Oxygenated ROC, $C_{i}^{*}=10^{-1}\mu \text{g}\;{\text{m}}^{-3}$ and $n_{\text{O}}:n_{\text{C}}$ of 0.6	GP	C(CCC(C(=O)O)O)CCC(=O)O
ROCN2ALK	Alkane-like ROC, $C_{i}^{*}=10^{-2}\mu \text{g}\;{\text{m}}^{-3}$	GP	CCCCCCCCCCCCCCCCCCCCCCCCCCCCCC
ROCN2OXY2	Oxygenated ROC, $C_{i}^{*}=10^{-2}\mu \text{g}\;{\text{m}}^{-3}$ and $n_{\text{O}}:n_{\text{C}}$ of 0.2	GP	C#CCCC[C@H](CCCCCCCCCCC(=O)O)O
ROCN2OXY4	Oxygenated ROC, $C_{i}^{*}=10^{-2}\mu \text{g}\;{\text{m}}^{-3}$ and $n_{\text{O}}:n_{\text{C}}$ of 0.4	GP	C(CCCCC(=O)O)CCCC(C(=O)O)O
ROCN2OXY8	Oxygenated ROC, $C_{i}^{*}=10^{-2}\mu \text{g}\;{\text{m}}^{-3}$ and $n_{\text{O}}:n_{\text{C}}$ of 0.8	GP	CC(=O)C(C(C(C(CO)O)O)O)O
ROCP0ALK	Alkane-like ROC, $C_{i}^{*}=10^{0}\mu \text{g}\;{\text{m}}^{-3}$	GP	CCCCCCCCCCCCCCCCC(C)CCCCCCCCCC
ROCP0OXY2	Oxygenated ROC, $C_{i}^{*}=10^{0}\mu \text{g}\;{\text{m}}^{-3}$ and $n_{\text{O}}:n_{\text{C}}$ of 0.2	GP	CCCCCCCCCCCC(=O)CC(=O)O
ROCP0OXY4	Oxygenated ROC, $C_{i}^{*}=10^{0}\mu \text{g}\;{\text{m}}^{-3}$ and $n_{\text{O}}:n_{\text{C}}$ of 0.4	GP	C(CCCCC(=O)O)CCCC(=O)O
ROCP1ALK	Alkane-like ROC, $C_{i}^{*}=10^{1}\mu \text{g}\;{\text{m}}^{-3}$	GP	CCCCCCCCCCCCCCCCCCCCCCCCCCC
ROCP1ALKP	Peroxy radicals from ROCP1ALK oxidation	G	CCCCCCCCCCCCCCCCCCCCCCCCC(CC)O[O]
ROCP1ALKP2	Hydroxy peroxy radicals from ROCP1ALK alkoxy product	G	CCCCCCCCCCCCCCCCCCCCCC(O[O])CCC(O)CC
ROCP1OXY1	Oxygenated ROC, $C_{i}^{*}=10^{1}\mu \text{g}\;{\text{m}}^{-3}$ and $n_{\text{O}}:n_{\text{C}}$ of 0.1	GP	CCCCCCCCCCCCCCCCC(=O)O
ROCP1OXY3	Oxygenated ROC, $C_{i}^{*}=10^{1}\mu \text{g}\;{\text{m}}^{-3}$ and $n_{\text{O}}:n_{\text{C}}$ of 0.3	GP	C(CCCCCO)CCCCC(=O)O
ROCP2ALK	Alkane-like ROC, $C_{i}^{*}=10^{2}\mu \text{g}\;{\text{m}}^{-3}$	GP	CCCCCCCCCCCCCCCCCCCCCCCC
ROCP2ALKP	Peroxy radicals from ROCP2ALK oxidation	G	CCCCCCCCCCCCCCCCCCCCCC(CC)O[O]
ROCP2ALKP2	Hydroxy peroxy radicals from ROCP2ALK alkoxy product	G	CCCCCCCCCCCCCCCCCCC(O[O])CCC(O)CC
ROCP2OXY2	Oxygenated ROC, $C_{i}^{*}=10^{2}\mu \text{g}\;{\text{m}}^{-3}$ and $n_{\text{O}}:n_{\text{C}}$ of 0.2	GP	CCCCCCCCCCCC(=O)O
ROCP3ALK	Alkane-like ROC, $C_{i}^{*}=10^{3}\mu \text{g}\;{\text{m}}^{-3}$	GP	CCCCCCCCCCCCCCCCCCCCC
ROCP3ALKP	Peroxy radicals from ROCP3ALK oxidation	G	CCCCCCCCCCCCCCCCCCC(CC)O[O]
ROCP3ALKP2	Hydroxy peroxy radicals from ROCP3ALK alkoxy product	G	CCCCCCCCCCCCCCCC(O[O])CCC(O)CC
ROCP3OXY2	Oxygenated ROC, $C_{i}^{*}=10^{3}\mu \text{g}\;{\text{m}}^{-3}$ and $n_{\text{O}}:n_{\text{C}}$ of 0.2	GP	C(CCCCCO)CCCCC=O
ROCP4ALK	Alkane-like ROC, $C_{i}^{*}=10^{4}\mu \text{g}\;{\text{m}}^{-3}$	G	CCCCCCCCCCCCCCCCCC
ROCP4ALKP	Peroxy radicals from ROCP4ALK oxidation	G	CCCCCCCCCCCCCCCC(CC)O[O]
ROCP4ALKP2	Hydroxy peroxy radicals from ROCP4ALK alkoxy product	G	CCCCCCCCCCCCC(O[O])CCC(O)CC
ROCP4OXY2	Oxygenated ROC, $C_{i}^{*}=10^{4}\mu \text{g}\;{\text{m}}^{-3}$ and $n_{\text{O}}:n_{\text{C}}$ of 0.2	G	CCCCCC(CC)C(=O)O
ROCP5ALK	Alkane-like ROC, $C_{i}^{*}=10^{5}\mu \text{g}\;{\text{m}}^{-3}$	G	CCCCCCCCCCCCCC
ROCP5ALKP	Peroxy radicals from ROCP5ALK oxidation	G	CCCCCCCCCCCC(CC)O[O]
ROCP5ALKP2	Hydroxy peroxy radicals from ROCP5ALK alkoxy product	G	CCCCCCCCC(O[O])CCC(O)CC
ROCP5ARO	Aromatic ROC, $C_{i}^{*}=10^{5}\mu \text{g}\;{\text{m}}^{-3}$	G	CCCCCCCCC1=CC=CC=C1
ROCP5AROP	Peroxy radicals from ROCP5ARO oxidation	G	CCCCCCCCC1(OO2)C=CC(O[O])C2C1O
ROCP5OXY1	Oxygenated ROC, $C_{i}^{*}=10^{5}\mu \text{g}\;{\text{m}}^{-3}$ and $n_{\text{O}}:n_{\text{C}}$ of 0.1	G	CCCCCCCCCCC=O
ROCP6ALK	Alkane-like ROC, $C_{i}^{*}=10^{6}\mu \text{g}\;{\text{m}}^{-3}$	G	CCCCCCCCCCCCC
ROCP6ALKP	Peroxy radicals from ROCP6ALK oxidation	G	CCCCCCCCCCC(CC)O[O]
ROCP6ALKP2	Hydroxy peroxy radicals from ROCP6ALK alkoxy product	G	CCCCCCCC(O[O])CCC(O)CC
ROCP6ARO	Aromatic ROC, $C_{i}^{*}=10^{6}\mu \text{g}\;{\text{m}}^{-3}$	G	CCCCCCC1=CC=C(C)C=C1
ROCP6AROP	Peroxy radicals from ROCP6ARO oxidation	G	OC1C2C(CCCCCC)(O[O])C=CC1(C)OO2
ROCP6OXY1	Oxygenated ROC, $C_{i}^{*}=10^{6}\mu \text{g}\;{\text{m}}^{-3}$ and $n_{\text{O}}:n_{\text{C}}$ of 0.1	G	CCCCCCCCC=O
ROH	C3 and higher alcohols	G	CCCO
SESQ	Sesquiterpenes	G	C/C1=C/CCC(=C)C2CC(C)(C)C2CC\1
SESQNRO2	Peroxy radicals from SESQ reaction with nitrate radicals	G	[O]OC1(C)CCC2C(CC2(C)C)C(=C)CCC1O[N+](=O)[O−]
SESQRO2	Peroxy radicals from SESQ reaction with HO	G	[O]OC1(C)CCC2C(CC2(C)C)C(=C)CCC1O
SLOWROC	Slowly reacting ROC with $k_{\text{OH}}<3.5\times 10^{-13}\;\text{molec}.{\text{cm}}^{-3}\;{\text{s}}^{-1}$	G	C#N
TOL	Toluene	G	CC1=CC=CC=C1
TOLP	Peroxy radicals formed from TOL	G	[O]OC1C=CC2(C)OOC1C2O
TRPN	Terpene nitrates	G	O=N(=O)OC1(C)C(O)CC2CC1C2(C)C
UALD	Unsaturated aldehydes	G	CC=C(C)C=O
UALP	Peroxy radicals formed from UALD	G	CC(O[O])C(C)(O)C=O
XYE	O- and p-xylene and other less reactive volatile aromatics with $k_{\text{OH}}<1.46\times 10^{-11}\;\text{molec}.{\text{cm}}^{-3}\;{\text{s}}^{-1}$	G	CCC1=CC=CC=C1
XYEP	Peroxy radicals formed from XYE	G	[O]OC1C=CC2(CC)OOC1C2O
XYM	M-xylene and other more reactive volatile aromatics with $k_{\text{OH}}<1.46\times 10^{-11}\;\text{molec}.{\text{cm}}^{-3}\;{\text{s}}^{-1}$	G	CC1=CC(C)=CC=C1
XYMP	Peroxy radicals formed from XYM	G	[O]OC1C=CC2(C)OOC1(C)C2O

## Appendix B

**Table B1.** Chemistry of CRACMMv1.0. For photolysis and heterogenous reactions (rate constant values not provided), rates depend on radiation, predicted concentrations, and/or other conditions, so a reference to the underlying data and formulation is provided. Rate constant values (k), if provided, are specified at 298.15 K, $\text{M}=2.4615\times 10^{19}\;\text{molec}.{\text{cm}}^{-3}$, and 1.00 atm. This information is also available in the supporting data archive and in CMAQv5.4. Partitioning of condensible organics is not listed here, and CMAQ assumes equilibrium partitioning calculated via operator splitting separate from the kinetic chemistry. Some coefficients have been rounded to the thousandths for brevity. Please see the "Code availability" section for where to find the mechanism files.

N	CMAQ label	Reaction	Rate constant formula^a,b,c^	$k(\text{molec}.{\text{cm}}^{-3}\;{\text{s}}^{-1}\;\text{or}\;{\text{s}}^{-1})$
1	R001	O3 → O3P	σ from Sander et al. (2011); ϕ=1.0 – ϕ of O_3_ (Reaction 2)	Not applicable
2	R002	O3 → O1D	σ and ϕ from Sander et al. (2011)	Not applicable
3	R003	H2O2 → 2.000 HO	σ from Sander et al. (2011); ϕ=1.0	Not applicable
4	R004	NO2 → O3P + NO	σ and ϕ from Sander et al. (2011)	Not applicable
5	R005	NO3 → NO	σ and ϕ from Sander et al. (2011)	Not applicable
6	R006	NO3 → O3P + NO2	σ and ϕ from Sander et al. (2011)	Not applicable
7	R007	HONO → HO + NO	σ from Sander et al. (2011); ϕ=1.0	Not applicable
8	R008	HNO3 → HO + NO2	σ from Sander et al. (2011); ϕ=1.0	Not applicable
9	R009	HNO4 → 0.200 HO + 0.800 HO2 + 0.800 NO2 + 0.200 NO3	σ from Sander et al. (2011); ϕ=1.0	Not applicable
10	R010	HCHO → CO	σ and ϕ from Sander et al. (2011)	Not applicable
11	R011	HCHO → 2.000 HO2 + CO	σ and ϕ from Sander et al. (2011)	Not applicable
12	R012	ACD → HO2 + MO2 + CO	σ and ϕ from Sander et al. (2011)	Not applicable
13	R013	ALD → HO2 + ETHP + CO	σ from Burkholder et al. (2019); ϕ from Heicklen et al. (1986) and IUPAC data sheet P3 (updated 16 May 2002)	Not applicable
14	R014	ACT → MO2 + ACO3	σ and ϕ from Burkholder et al. (2019)	Not applicable
15	R014a	ACT → 2.000 MO2 + CO	σ and ϕ from Burkholder et al. (2019)	Not applicable
16	R015	UALD → 1.220 HO2 + 0.784 ACO3 + 1.220 CO + 0.350 HCHO + 0.434 ALD + 0.216 KET	σ and ϕ from Magneron et al. (2002); uses crotonaldehyde	Not applicable
17	TRP01	PINAL → HO2 + HC10P + CO	Uses data for ALD (Reaction 13)	Not applicable
18	TRP02	LIMAL → HO2 + HC10P + CO	Uses data for ALD (Reaction 13)	Not applicable
19	R016	MEK → 0.100 MO2 + ETHP + 0.900 ACO3 + 0.100 CO	σ from Brewer et al. (2019); ϕ from IUPAC data sheet P8 (5 December 2005)	Not applicable
20	R017	KET → 1.500 ETHP + 0.500 ACO3 + 0.500 CO	σ from Brewer et al. (2019); ϕ from IUPAC data sheet P8 (5 December 2005)	Not applicable
21	R018	HKET → HO2 + ACO3 + HCHO	σ from Yujing and Mellouki (2000); ϕ from IUPAC data sheet P8 (5 December 2005)	Not applicable
22	R019	MACR → 0.340 HO + 0.660 HO2 + 0.670 ACO3 + 0.330 MACP + 0.340 XO2 + 0.670 CO + 0.670 HCHO	σ and ϕ from Sander et al. (2011)	Not applicable
23	R020	MVK → 0.300 MO2 + 0.300 MACP + 0.700 CO + 0.700 UALD	σ and ϕ from Sander et al. (2011)	Not applicable
24	R021	GLY → 2.000 CO	σ and ϕ from Sander et al. (2011)	Not applicable
25	R022	GLY → HCHO + CO	σ and ϕ from Sander et al. (2011)	Not applicable
26	R023	GLY → 2.000 HO2 + 2.000 CO	σ and ϕ from Sander et al. (2011)	Not applicable
27	R024	MGLY → HO2 + ACO3 + CO	σ and ϕ from Sander et al. (2011)	Not applicable
28	R025	DCB1 → 1.500 HO2 + 0.250 ACO3 + 0.200 XO2 + CO + 0.500 GLY + 0.500 MGLY	Uses data for MGLY (Reaction 27)	Not applicable
29	R026	DCB2 → 1.500 HO2 + 0.250 ACO3 + 0.200 XO2 + CO + 0.500 GLY + 0.500 MGLY	Uses data for MGLY (Reaction 27)	Not applicable
30	R027	BALD → CHO + HO2 + CO	σ and ϕ from SAPRC-07 (Carter, 2010)	Not applicable
31	R028	OP1 → HO + HO2 + HCHO	σ from Sander et al. (2011); ϕ=1.0	Not applicable
32	R029	OP2 → HO + HO2 + ALD	Uses data for OP1 (Reaction 31)	Not applicable
33	TRP03	OPB → HO + HO2 + ALD	Uses data for OP1 (Reaction 31)	Not applicable
34	R029a	OP3 → HO + HO2 + ALD	Uses data for OP1 (Reaction 31)	Not applicable
35	R030	PAA → HO + MO2	σ from Sander et al. (2011); ϕ=1.0	Not applicable
36	R031	ONIT → HO2 + NO2 + 0.200 ALD + 0.800 KET	σ from Talukdar et al. (1997); ϕ=1.0	Not applicable
37	R032	PAN → ACO3 + NO2	σ and ϕ from Sander et al. (2011)	Not applicable
38	R033	PAN → MO2 + NO3	σ from Sander et al. (2011); ϕ=1.0 – Reaction 37 ϕ	Not applicable
39	R034	O3 + HO → HO2	1.70 × 10^−12^exp(−940.00*/T*)	7.26 × 10^−14^
40	R035	O3 + HO2 → HO	1.00 × 10^−14^exp(−490.00*/T*)	1.93 × 10^−15^
41	R036	O3 + NO → NO2	3.00 × 10^−12^exp(−1500.00*/T*)	1.96 × 10^−14^
42	R037	O3 + NO2 → NO3	1.20 × 10^−13^exp(−2450.00*/T*)	3.24 × 10^−17^
43	R038	O3P + O2 + M → O3	6.10 × 10−34(*T/*300)^−2.40^	6.19 × 10^−34^
44	R039	O3P + O3 →	8.00 × 10^−12^exp(−2060.00*/T*)	7.99 × 10^−15^
45	R040	O1D + O2 → O3P	3.30 × 10^−11^exp(55.00*/T*)	3.97 × 10^−11^
46	R041	O1D + N2 → O3P	2.15 × 10^−11^exp(110.00*/T*)	3.11 × 10^−11^
47	R042	O1D + H2O → 2.000 HO	1.63 × 10^−10^exp(60.00*/T*)	1.99 × 10^−10^
48	R043	HO + H2 → HO2	2.80 × 10^−12^exp(−1800.00*/T*)	6.69 × 10^−15^
49	R044	HO + HO2 →	4.80 × 10^−11^exp(250.00*/T*)	1.11 × 10^−10^
50	R045	HO2 + HO2 → H2O2	$k_{0}=3.00\times 10^{-13}\text{exp}\left(460.0/T\right)$; $k_{1}=2.10\times 10^{-33}\text{exp}\left(920.0/T\right)$	2.53 × 10^−12^
51	R046	HO2 + HO2 + H2O → H2O2	$k_{0}=4.20\times 10^{-34}\text{exp}\left(2660.0/T\right)$; $k_{1}=2.94\times 10^{-54}\text{exp}\left(3120.0/T\right)$	5.68 × 10^−30^
52	R047	H2O2 + HO → HO2	1.80 × 10^−12^exp(0.00*/T*)	1.80 × 10^−12^
53	R048	NO + O3P → NO2	$k_{o}=9.10\times 10^{-32}\text{exp}\left(0.0/T\right){\left(T/300\right)}^{-1.50}$; $k_{i}=3.00\times 10^{-11}\text{exp}\left(0.0/T\right){\left(T/300\right)}^{-0.00}$; n=1.0; F=0.60	1.68 × 10^−12^
54	R049	NO + HO → HONO	$k_{o}=7.10\times 10^{-31}\text{exp}\left(0.0/T\right){\left(T/300\right)}^{-2.60}$; $k_{i}=3.60\times 10^{-11}\text{exp}\left(0.0/T\right){\left(T/300\right)}^{-0.10}$; n=1.0; F=0.60	7.46 × 10^−12^
55	R050	NO + HO2 → NO2 + HO	3.44 × 10^−12^exp(260.00*/T*)	8.23 × 10^−12^
56	R051	NO + HO2 → HNO3	$k_{0}=6.0950\times 10^{-14}\text{exp}\left(270.0/T\right){\left(T/300\right)}^{-1.00}$; $k2=6.8570\times 10^{-34}\text{exp}\left(270.0/T\right){\left(T/300\right)}^{-1.00}$; $k3=-5.9680\times 10^{-14}\text{exp}\left(2700.0/T\right)$	4.56 × 10^−14^
57	R052	NO + NO + O2 → 2.000 NO2	4.25 × 10^−39^exp(663.50*/T*)	3.93 × 10^−38^
58	R053	HONO + HO → NO2	3.00 × 10^−12^exp(250.00*/T*)	6.94 × 10^−12^
59	R054	NO2 + O3P → NO	5.30 × 10^−12^exp(200.00*/T*)	1.04 × 10^−11^
60	R055	NO2 + O3P → NO3	$k_{o}=3.40\times 10^{-31}\text{exp}\left(0.0/T\right){\left(T/300\right)}^{-1.60}$; $k_{i}=2.30\times 10^{-11}\text{exp}\left(0.0/T\right){\left(T/300\right)}^{-0.20}$; n=1.00; F=0.60	4.02 × 10^−12^
61	R056	NO2 + HO → HNO3	$k_{o}=1.80\times 10^{-30}\text{exp}\left(0.0/T\right){\left(T/300\right)}^{-3.00}$; $k_{i}=2.80\times 10^{-11}\text{exp}\left(0.0/T\right){\left(T/300\right)}^{-0.00}$; n=1.00; F=0.60	1.06 × 10^−11^
62	R057	HNO3 + HO → NO3	$k_{0}=2.40\times 10^{-14}\text{exp}\left(460.0/T\right)$; $k_{1}=2.70\times 10^{-17}\text{exp}\left(2199.0/T\right)$; $k3=6.50\times 10^{-34}\text{exp}\left(1335.0/T\right)$	1.54 × 10^−13^
63	R058	NO3 + HO → HO2 + NO2	2.00 × 10^−11^	2.00 × 10^−11^
64	R059	NO3 + HO2 → 0.700 HO + 0.700 NO2 + 0.300 HNO3	3.50 × 10^−12^	3.50 × 10^−12^
65	R060	NO3 + NO → 2.000 NO2	1.70 × 10^−11^exp(125.00*/T*)	2.59 × 10^−11^
66	R061	NO3 + NO2 → NO + NO2	4.35 × 10^−14^exp(−1335.00*/T*)	4.94 × 10^−16^
67	R062	NO3 + NO3 → 2.000 NO2	8.50 × 10^−13^exp(−2450.00*/T*)	2.29 × 10^−16^
68	R063	NO3 + NO2 → N2O5	$k_{o}=2.40\times 10^{-30}\text{exp}\left(0.0/T\right){\left(T/300\right)}^{-3.00}$; $k_{i}=1.60\times 10^{-12}\text{exp}\left(0.0/T\right){\left(T/300\right)}^{-0.10}$; n=1.00; F=0.60	1.35 × 10^−12^
69	R064	N2O5 → NO2 + NO3	1.72 × 10^26^exp(−10840.00*/T*) R063	3.76 × 10^−28^
70	R065	N2O5 + H2O → 2.000 HNO3	1.00 × 10^−22^	1.00 × 10^−22^
71	R066	NO2 + HO2 → HNO4	$k_{o}=1.90\times 10^{-31}\text{exp}\left(0.0/T\right){\left(T/300\right)}^{-3.40}$; $k_{i}=4.00\times 10^{-12}\text{exp}\left(0.0/T\right){\left(T/300\right)}^{-0.30}$; n=1.00; F=0.60	1.31 × 10^−12^
72	R067^d^	HNO4 → HO2 + NO2	4.76 × 10^26^exp(−10900.00*/T*) R066	8.28 × 10^−28^
73	R068	HNO4 + HO → NO2	4.50 × 10^−13^exp(610.00*/T*)	3.48 × 10^−12^
74	R069^f^	SO2 + HO → HO2 + SULF	$k_{o}=2.90\times 10^{-31}\text{exp}\left(0.0/T\right){\left(T/300\right)}^{-4.10}$; $k_{i}=1.70\times 10^{-12}\text{exp}\left(0.0/T\right){\left(T/300\right)}^{0.20}$; n=1.00; F=0.60	9.58 × 10^−13^
75	R070	CO + HO → HO2	$k_{0}=1.44\times 10^{-13}\text{exp}\left(0.0/T\right)$; $k_{1}=2.74\times 10^{-33}\text{exp}\left(0.0/T\right)$; 2.45 × 10^−12^exp(−1775.00*/T*)	2.11 × 10^−13^
76	R071	HO + CH4 → MO2	2.45 × 10^−12^exp(−1775.00*/T*)	6.36 × 10^−15^
77	R072	ETH + HO → ETHP	7.66 × 10^−12^exp(−1020.00*/T*)	2.50 × 10^−13^
78	R073	HC3 + HO → HC3P + 2.81 × 10^−5^ ASOATJ	7.68 × 10^−12^exp(−370.00*/T*)	2.22 × 10^−12^
79	R074	HC5 + HO → HC5P + 0.001 ASOATJ	1.01 × 10^−11^exp(−245.00*/T*)	4.44 × 10^−12^
80	R076	ETE + HO → ETEP	$k_{o}=1.00\times 10^{-28}\text{exp}\left(0.0/T\right){\left(T/300\right)}^{-4.50}$; $k_{i}=8.80\times 10^{-12}\text{exp}\left(0.0/T\right){\left(T/300\right)}^{-0.85}$; n=1.00; F=0.60	8.20 × 10^−12^
81	R077	OLT + HO → OLTP	5.72 × 10^−12^exp(500.00*/T*)	3.06 × 10^−11^
82	R078	OLI + HO → OLIP	1.33 × 10^−11^exp(500.00*/T*)	7.11 × 10^−11^
83	R080	ACE + HO → 0.650 HO + 0.350 HO2 + 0.350 CO + 0.650 GLY + 0.350 ORA1	$k_{o}=5.50\times 10^{-30}\text{exp}\left(0.0/T\right){\left(T/300\right)}^{0.00}$; $k_{i}=8.30\times 10^{-13}\text{exp}\left(0.0/T\right){\left(T/300\right)}^{2.00}$; n=1.00; F=0.60	7.47 × 10^−13^
84	ROCARO31	BEN + HO → 0.470 BENP + 0.530 PHEN + 0.530 HO2	2.33 × 10^−12^exp(−193.00*/T*)	1.22 × 10^−12^
85	ROCARO41	TOL + HO → 0.820 TOLP + 0.180 CSL + 0.180 HO2	1.81 × 10^−12^exp(354.00*/T*)	5.93 × 10^−12^
86	ROCARO51	XYM + HO → 0.830 XYMP + 0.170 CSL + 0.170 HO2	2.33 × 10^−11^	2.33 × 10^−11^
87	ROCARO61	XYE + HO → 0.820 XYEP + 0.180 CSL + 0.180 HO2	7.16 × 10^−12^	7.16 × 10^−12^
88	R086	ISO + HO → ISOP	2.70 × 10^−11^exp(390.00*/T*)	9.99 × 10^−11^
89	R087	API + HO → 0.975 APIP1 + 0.025 APIP2	1.21 × 10^−11^exp(440.00*/T*)	5.29 × 10^−11^
90	R088	LIM + HO → 0.945 LIMP1 + 0.055 LIMP2	4.20 × 10^−11^exp(401.00*/T*)	1.61 × 10^−10^
91	TRP04	PINAL + HO → 0.230 PINALP + 0.770 RCO3	5.20 × 10^−12^exp(600.00*/T*)	3.89 × 10^−11^
92	TRP05	LIMAL + HO → 0.700 LIMALP + 0.300 RCO3	1.00 × 10^−10^	1.00 × 10^−10^
93	R089	HCHO + HO → HO2 + CO	5.50 × 10^−12^exp(125.00*/T*)	8.36 × 10^−12^
94	R090	ACD + HO → ACO3	4.70 × 10^−12^exp(345.00*/T*)	1.50 × 10^−11^
95	R091	ALD + HO → RCO3	4.90 × 10^−12^exp(405.00*/T*)	1.91 × 10^−11^
96	R092	ACT + HO → ACTP	4.56 × 10^−14^exp(−427.00*/T*)(*T/*300)^3.65^	1.06 × 10^−14^
97	R093	MEK + HO → MEKP	1.50 × 10^−12^exp(−90.00*/T*)	1.11 × 10^−12^
98	R094	KET + HO → KETP	2.80 × 10^−12^exp(10.00*/T*)	2.90 × 10^−12^
99	R095	HKET + HO → HO2 + MGLY	3.00 × 10^−12^	3.00 × 10^−12^
100	R096	MACR + HO → 0.570 MACP + 0.430 MCP	8.00 × 10^−12^exp(380.00*/T*)	2.86 × 10^−11^
101	R097	MVK + HO → MVKP	2.60 × 10^−12^exp(610.00*/T*)	2.01 × 10^−11^
102	R098	UALD + HO → 0.313 ACO3 + 0.687 UALP	5.77 × 10^−12^exp(533.00*/T*)	3.45 × 10^−11^
103	R099	GLY + HO → HO2 + 2.000 CO	1.10 × 10^−11^	1.10 × 10^−11^
104	R100	MGLY + HO → ACO3 + CO	9.26 × 10^−13^exp(830.00*/T*)	1.50 × 10^−11^
105	R101	DCB1 + HO → 0.520 HO2 + 0.330 CO + 0.400 ALD + 0.780 KET + 0.100 GLY + 0.010 MGLY	2.80 × 10^−11^exp(175.00*/T*)	5.04 × 10^−11^
106	R102	DCB2 + HO → 0.520 HO2 + 0.330 CO + 0.130 MEK + 0.100 GLY + 0.010 MGLY + 0.780 OP2	2.80 × 10^−11^exp(175.00*/T*)	5.04 × 10^−11^
107	R103	DCB3 + HO → 0.560 HO2 + 0.210 MACP + 0.110 CO + 0.270 GLY + 0.010 MGLY + 0.790 OP2	1.00 × 10^−11^	1.00 × 10^−11^
108	R104	BALD + HO → BALP	5.32 × 10^−12^exp(243.00*/T*)	1.20 × 10^−11^
109	R105	PHEN + HO → 0.152 ASOATJ + 0.619 HO2 + 0.170 ADDC + 0.059 CHO + 0.619 MCT	6.75 × 10^−12^exp(405.00*/T*)	2.63 × 10^−11^
110	R106	CSL + HO → 0.200 ASOATJ + 0.584 HO2 + 0.160 ADDC + 0.056 CHO + 0.584 MCT	4.65 × 10^−11^exp(0.00*/T*)	4.65 × 10^−11^
111	R108	MCT + HO → MCTO	2.05 × 10^−10^exp(0.00*/T*)	2.05 × 10^−10^
112	R109	MOH + HO → HO2 + HCHO	2.85 × 10^−12^exp(−345.00*/T*)	8.96 × 10^−13^
113	R110	EOH + HO → HO2 + ACD	3.00 × 10^−12^exp(20.00*/T*)	3.21 × 10^−12^
114	R111	ROH + HO → HO2 + 0.719 ALD + 0.184 ACD	2.60 × 10^−12^exp(200.00*/T*)	5.09 × 10^−12^
115	R112	ETEG + HO → HO2 + ALD	1.47 × 10^−11^	1.47 × 10^−11^
116	R113	OP1 + HO → 0.350 HO + 0.650 MO2 + 0.350 HCHO	2.90 × 10^−12^exp(190.00*/T*)	5.48 × 10^−12^
117	R114	OP2 + HO → 0.010 HO + 0.440 HC3P + 0.070 XO2 + 0.080 ALD + 0.410 KET	3.40 × 10^−12^exp(190.00*/T*)	6.43 × 10^−12^
118	TRP06	OPB + HO → 0.010 HO + 0.440 HC10P + 0.070 XO2 + 0.080 ALD + 0.410 KET	3.40 × 10^−12^exp(190.00*/T*)	6.43 × 10^−12^
119	R114a	OP3 + HO → 0.010 HO + 0.440 HC10P + 0.070 XO2 + 0.080 ALD + 0.410 KET	3.40 × 10^−12^exp(190.00*/T*)	6.43 × 10^−12^
120	R115	ISHP + HO → HO + MACR + 0.904 IEPOX	1.00 × 10^−10^	1.00 × 10^−10^
121	R116	MAHP + HO → MACP	3.00 × 10^−11^	3.00 × 10^−11^
122	R117	ORA1 + HO → HO2	4.50 × 10^−13^	4.50 × 10^−13^
123	R118	ORA2 + HO → 0.640 MO2 + 0.360 ORAP	4.00 × 10^−14^exp(850.00*/T*)	6.92 × 10^−13^
124	R119	PAA + HO → 0.350 HO + 0.650 ACO3 + 0.350 XO2 + 0.350 HCHO	2.93 × 10^−12^exp(190.00*/T*)	5.54 × 10^−12^
125	R120	PAN + HO → XO2 + NO3 + HCHO	4.00 × 10^−14^	4.00 × 10^−14^
126	R121	PPN + HO → XO2 + NO3 + HCHO	4.00 × 10^−14^	4.00 × 10^−14^
127	R122	MPAN + HO → NO2 + HKET	3.20 × 10^−11^	3.20 × 10^−11^
128	R123	ONIT + HO → HC3P + NO2	5.31 × 10^−12^exp(−260.00*/T*)	2.22 × 10^−12^
129	TRP07	TRPN + HO → HOM	4.80 × 10^−12^	4.80 × 10^−12^
130	R124	NALD + HO → NO2 + XO2 + HKET	5.60 × 10^−12^exp(270.00*/T*)	1.39 × 10^−11^
131	R125	ISON + HO → NALD + 0.070 HKET + 0.070 HCHO	1.30 × 10^−11^	1.30 × 10^−11^
132	R126	ETE + O3 → 0.080 HO + 0.150 HO2 + 0.430 CO + HCHO + 0.370 ORA1	9.14 × 10^−15^exp(−2580.00*/T*)	1.60 × 10^−18^
133	R127	OLT + O3 → 0.220 HO + 0.320 HO2 + 0.080 MO2 + 0.060 ETHP + 0.040 HC3P + 0.020 HC5P + 0.068 H2O2 + 0.430 CO + 0.020 ETH + 0.015 HC3 + 0.006 HC5 + 0.032 BEN + 0.560 HCHO + 0.010 ACD + 0.440 ALD + 0.030 ACT + 0.020 BALD + 0.060 MEK + 0.010 HKET + 0.030 ORA1 + 0.060 ORA2	4.33 × 10^−15^exp(−1800.00*/T*)	1.03 × 10^−17^
134	R128	OLI + O3 → 0.460 HO + 0.070 HO2 + 0.320 MO2 + 0.070 ETHP + 0.040 HC3P + 0.090 ACO3 + 0.370 CO + 0.026 H2O2 + 0.010 ETH + 0.010 HC3 + 0.090 HCHO + 0.457 ACD + 0.730 ALD + 0.110 ACT + 0.017 KET + 0.044 HKET + 0.017 ORA2	4.40 × 10^−15^exp(−845.00*/T*)	2.59 × 10^−16^
135	R130	ISO + O3 → 0.250 HO + 0.250 HO2 + 0.080 MO2 + 0.100 ACO3 + 0.100 MACP + 0.090 H2O2 + 0.140 CO + 0.580 HCHO + 0.461 MACR + 0.189 MVK + 0.280 ORA1 + 0.153 OLT	7.86 × 10^−15^exp(−1913.00*/T*)	1.29 × 10^−17^
136	R131	API + O3 → 0.900 HO + 0.900 APIP1 + 0.050 APIP2 + 0.050 PINAL + 0.050 H2O2 + 0.140 CO	5.00 × 10^−16^exp(−530.00*/T*)	8.45 × 10^−17^
137	R132	LIM + O3 → 0.840 HO + 0.840 LIMP1 + 0.110 LIMP2 + 0.050 LIMAL + 0.050 H2O2 + 0.140 CO	2.95 × 10^−15^exp(−783.00*/T*)	2.13 × 10^−16^
138	TRP08	LIMAL + O3 → 0.040 HO + 0.670 HC10P + 0.790 HCHO + 0.330 KET + 0.040 HO2 + 0.200 CO	8.30 × 10^−18^	8.30 × 10^−18^
139	TRP09	TRPN + O3 → HOM	1.67 × 10^−16^	1.67 × 10^−16^
140	R132	MACR + O3 → 0.190 HO + 0.140 HO2 + 0.100 ACO3 + 0.220 CO + 0.500 MGLY + 0.450 ORA1	1.36 × 10^−15^exp(−2112.00*/T*)	1.14 × 10^−18^
141	R134	MVK + O3 → 0.160 HO + 0.110 HO2 + 0.280 ACO3 + 0.010 XO2 + 0.560 CO + 0.100 HCHO + 0.540 MGLY + 0.070 ORA1 + 0.070 ORA2 + 0.100 ALD	8.50 × 10^−16^exp(−1520.00*/T*)	5.19 × 10^−18^
142	R135	UALD + O3 → 0.100 HO + 0.072 HO2 + 0.008 MO2 + 0.002 ACO3 + 0.100 XO2 + 0.243 CO + 0.080 HCHO + 0.420 ACD + 0.028 KET + 0.491 GLY + 0.003 MGLY + 0.044 ORA1	1.66 × 10^−18^	1.66 × 10^−18^
143	R136	DCB1 + O3 → 0.050 HO + HO2 + 0.600 RCO3 + 0.600 XO2 + 1.500 CO + 0.050 HCHO + 0.050 GLY + 0.080 MGLY + 0.650 OP2	2.00 × 10^−16^	2.00 × 10^−16^
144	R137	DCB2 + O3 → 0.050 HO + HO2 + 0.600 RCO3 + 0.600 XO2 + 1.500 CO + 0.050 HCHO + 0.050 GLY + 0.080 MGLY + 0.700 DCB1 + 0.650 OP2	2.00 × 10^−16^	2.00 × 10^−16^
145	R138	DCB3 + O3 → 0.050 HO + HO2 + 1.500 CO + 0.480 GLY + 0.700 DCB1 + 0.250 ORA1 + 0.250 ORA2 + 0.110 PAA	9.00 × 10^−17^	9.00 × 10^−17^
146	R140	MCTO + O3 → MCTP	2.86 × 10^−13^	2.86 × 10^−13^
147	R141	ETE + NO3 → 0.800 OLNN + 0.200 OLND	4.39 × 10^−13^exp(−2282.00*/T*)(*T/*300)^2.00^	2.06 × 10^−16^
148	R142	OLT + NO3 → 0.430 OLNN + 0.570 OLND	1.79 × 10^−13^exp(−450.00*/T*)	3.96 × 10^−14^
149	R143	OLI + NO3 → 0.110 OLNN + 0.890 OLND	8.64 × 10^−13^exp(450.00*/T*)	3.91 × 10^−12^
150	R145	ISO + NO3 → ISON	3.03 × 10^−12^exp(−446.00*/T*)	6.79 × 10^−13^
151	R146	API + NO3 → 0.975 APINP1 + 0.025 APINP2	1.19 × 10^−12^exp(490.00*/T*)	6.16 × 10^−12^
152	R147	LIM + NO3 → 0.945 LIMNP1 + 0.055 LIMNP2	1.22 × 10^−11^	1.22 × 10^−11^
153	TRP10	TRPN + NO3 → HOM	3.15 × 10^−14^exp(−448.00*/T*)	7.01 × 10^−15^
154	R148	HCHO + NO3 → HO2 + CO + HNO3	2.00 × 10^−12^exp(−2440.00*/T*)	5.58 × 10^−16^
155	R149	ACD + NO3 → ACO3 + HNO3	1.40 × 10^−12^exp(−1900.00*/T*)	2.39 × 10^−15^
156	R150	ALD + NO3 → RCO3 + HNO3	3.76 × 10^−12^exp(−1900.00*/T*)	6.42 × 10^−15^
157	R151	MACR + NO3 → 0.680 HCHO + 0.320 MACP + 0.680 XO2 + 0.680 MGLY + 0.320 HNO3 + 0.680 NO2	3.40 × 10^−15^	3.40 × 10^−15^
158	R152	UALD + NO3 → HO2 + XO2 + 0.668 CO + 0.332 HCHO + 0.332 ALD + ONIT	5.02 × 10^−13^exp(−1076.00*/T*)	1.36 × 10^−14^
159	R153	GLY + NO3 → HO2 + 2.000 CO + HNO3	2.90 × 10^−12^exp(−1900.00*/T*)	4.95 × 10^−15^
160	R154	MGLY + NO3 → ACO3 + CO + HNO3	3.76 × 10^−12^exp(−1900.00*/T*)	6.42 × 10^−15^
161	R155	PHEN + NO3 → 0.152 ASOATJ + 0.339 CHO + 0.850 ADDC + 0.424 ADCN + 0.424 HNO3	3.78 × 10^−12^	3.78 × 10^−12^
162	R156	CSL + NO3 → 0.200 ASOATJ + 0.320 CHO + 0.080 ADDC + 0.400 ADCN + 0.400 HNO3	1.06 × 10^−12^	1.06 × 10^−12^
163	R158	MCT + NO3 → MCTO + HNO3	2.01 × 10^−10^	2.01 × 10^−10^
164	R159	MPAN + NO3 → MACP + NO2	2.20 × 10^−14^exp(−500.00*/T*)	4.11 × 10^−15^
165	TRP11	PINALP → HOM	1.00	1.00
166	TRP12	LIMALP → HOM	1.00	1.00
167	R166	ACO3 + NO2 → PAN	$k_{o}=9.70\times 10^{-29}\text{exp}\left(0.0/T\right){\left(T/300\right)}^{-5.60}$; $k_{i}=9.30\times 10^{-12}\text{exp}\left(0.0/T\right){\left(T/300\right)}^{-1.50}$; n=1.00; F=0.60	8.68 × 10^−12^
168	R167	PAN → ACO3 + NO2	1.11 × 10^28^exp−(14000.00*/T*) R166	3.90 × 10^−48^
169	R168	RCO3 + NO2 → PPN	$k_{o}=9.70\times 10^{-29}\text{exp}\left(0.0/T\right){\left(T/300\right)}^{-5.60}$; $k_{i}=9.30\times 10^{-12}\text{exp}\left(0.0/T\right){\left(T/300\right)}^{-1.50}$; n=1.00; F=0.60	8.68 × 10^−12^
170	R169	PPN → RCO3 + NO2	1.11 × 10^28^exp(−14000.00*/T*) R168	3.90 × 10^−48^
171	R170	MACP + NO2 → MPAN	2.80 × 10^−12^exp(181.00*/T*)	5.14 × 10^−12^
172	R171	MPAN → MACP + NO2	1.60 × 10^16^exp(−13486.00*/T*)	3.63 × 10^−04^
173	R172	MO2 + NO → HO2 + NO2 + HCHO	2.80 × 10^−12^exp(300.00*/T*)	7.66 × 10^−12^
174	R173	ETHP + NO → HO2 + NO2 + ACD	2.60 × 10^−12^exp(365.00*/T*)	8.84 × 10^−12^
175	R174	HC3P + NO → 0.660 HO2 + 0.131 MO2 + 0.048 ETHP + 0.089 XO2 + 0.935 NO2 + 0.504 ACD + 0.132 ALD + 0.165 ACT + 0.042 MEK + 0.065 ONIT	4.00 × 10^−12^	4.00 × 10^−12^
176	R175	HC5P + NO → 0.200 HO2 + 0.051 MO2 + 0.231 ETHP + 0.235 XO2 + 0.864 NO2 + 0.018 HCHO + 0.045 ACD + 0.203 ALD + 0.033 MEK + 0.217 ACT + 0.033 KET + 0.272 HKET + 0.136 ONIT	4.00 × 10^−12^	4.00 × 10^−12^
177	R177	ETEP + NO → HO2 + NO2 + 1.600 HCHO + 0.200 ALD	9.00 × 10^−12^	9.00 × 10^−12^
178	R178	OLTP + NO → 0.780 HO2 + 0.970 NO2 + 0.780 HCHO + 0.012 ACD + 0.440 ALD + 0.060 ACT + 0.130 MEK + 0.030 ONIT	4.00 × 10^−12^	4.00 × 10^−12^
179	R179	OLIP + NO → 0.830 HO2 + 0.950 NO2 + 0.810 ACD + 0.680 ALD + 0.200 ACT + 0.090 KET + 0.020 HKET + 0.050 ONIT	4.00 × 10^−12^	4.00 × 10^−12^
180	ROCARO33	BENP + NO → 0.000 ONIT + 0.001 VROCP4OXY2 + 0.001 VROCN1OXY6 + 0.998 NO2 + 0.998 HO2 + 0.000 BALD + 0.998 GLY + 0.499 FURANONE + 0.249 DCB2 + 0.249 DCB3	2.70 × 10^−12^exp(360.00*/T*)	9.03 × 10^−12^
181	ROCARO43	TOLP + NO → 2 × 10^−4^ ONIT + 0.001 VROCP4OXY2 + 0.001 VROCN1OXY6 + 0.998 NO2 + 0.998 HO2 + 0.085 BALD + 0.548 GLY + 0.365 MGLY + 0.365 FURANONE + 0.548 DCB1	2.70 × 10^−12^exp(360.00*/T*)	9.03 × 10^−12^
182	ROCARO53	XYMP + NO → 1 × 10^−4^ ONIT + 0.001 VROCP3OXY2 + 0.001 VROCP0OXY4 + 0.998 NO2 + 0.998 HO2 + 0.048 BALD + 0.703 GLY + 0.247 MGLY + 0.351 FURANONE + 0.598 DCB2	2.70 × 10^−12^exp(360.00*/T*)	9.03 × 10^−12^
183	ROCARO63	XYEP + NO → 2 × 10^−4^ ONIT + 0.001 VROCP3OXY2 + 0.001 VROCP0OXY4 + 0.998 NO2 + 0.998 HO2 + 0.085 BALD + 0.548 GLY + 0.365 MGLY + 0.456 FURANONE + 0.456 DCB2	2.70 × 10^−12^exp(360.00*/T*)	9.03 × 10^−12^
184	R188	ISOP + NO → 0.880 HO2 + 0.880 NO2 + 0.200 HCHO + 0.280 MACR + 0.440 MVK + 0.120 ISON + 0.021 GLY + 0.029 HKET + 0.027 ALD	2.43 × 10^−12^exp(360.00*/T*)	8.13 × 10^−12^
185	R189	APIP1 + NO → 0.820 HO2 + 0.820 NO2 + 0.820 PINAL + 0.180 TRPN	4.00 × 10^−12^	4.00 × 10^−12^
186	TRP13	APIP2 + NO → 0.820 HO + 0.820 NO2 + HOM	4.00 × 10^−12^	4.00 × 10^−12^
187	TRP14	APINP1 + NO → 2.000 NO2 + PINAL	4.00 × 10^−12^	4.00 × 10^−12^
188	TRP15	APINP2 + NO → 0.820 NO2 + 0.820 HO + HOM	4.00 × 10^−12^	4.00 × 10^−12^
189	R190	LIMP1 + NO → 0.770 HO2 + 0.770 NO2 + 0.490 LIMAL + 0.280 HCHO + 0.280 UALD + 0.230 TRPN	4.00 × 10^−12^	4.00 × 10^−12^
190	TRP16	LIMP2 + NO → 0.770 HO + 0.770 NO2 + HOM	4.00 × 10^−12^	4.00 × 10^−12^
191	TRP17	LIMNP1 + NO → 2.000 NO2 + LIMAL	4.00 × 10^−12^	4.00 × 10^−12^
192	TRP18	LIMNP2 + NO → 0.770 NO2 + 0.770 HO + HOM	4.00 × 10^−12^	4.00 × 10^−12^
193	TRP19	PINALP + NO → 0.950 HO2 + 0.950 NO2 + 0.050 TRPN + 0.950 HCHO + 0.950 KET	2.70 × 10^−12^exp(360.00*/T*)	9.03 × 10^−12^
194	TRP20	LIMALP + NO → 0.940 HO2 + 0.940 NO2 + 0.060 TRPN + 0.940 HCHO + 0.940 KET	2.70 × 10^−12^exp(360.00*/T*)	9.03 × 10^−12^
195	R191	ACO3 + NO → MO2 + NO2	8.10 × 10^−12^exp(270.00*/T*)	2.00 × 10^−11^
196	R192	RCO3 + NO → ETHP + NO2	8.10 × 10^−12^exp(270.00*/T*)	2.00 × 10^−11^
197	R193	ACTP + NO → ACO3 + NO2 + HCHO	2.90 × 10^−12^exp(300.00*/T*)	7.93 × 10^−12^
198	R194	MEKP + NO → 0.670 HO2 + NO2 + 0.330 HCHO + 0.670 DCB1	4.00 × 10^−12^	4.00 × 10^−12^
199	R195	KETP + NO → 0.770 HO2 + 0.230 ACO3 + 0.160 XO2 + NO2 + 0.460 ALD + 0.540 MGLY	4.00 × 10^−12^	4.00 × 10^−12^
200	R196	MACP + NO → 0.650 MO2 + 0.350 ACO3 + NO2 + 0.650 CO + 0.650 HCHO	2.54 × 10^−12^exp(360.00*/T*)	8.50 × 10^−12^
201	R197	MCP + NO → NO2 + 0.500 HO2 + 0.500 HCHO + HKET	2.54 × 10^−12^exp(360.00*/T*)	8.50 × 10^−12^
202	R198	MVKP + NO → 0.300 HO2 + 0.700 ACO3 + 0.700 XO2 + NO2 + 0.300 HCHO + 0.700 ALD + 0.300 MGLY	2.54 × 10^−12^exp(360.00*/T*)	8.50 × 10^−12^
203	R199	UALP + NO → HO2 + NO2 + 0.610 CO + 0.030 HCHO + 0.270 ALD + 0.180 GLY + 0.700 KET + 0.210 MGLY	2.54 × 10^−12^exp(360.00*/T*)	8.50 × 10^−12^
204	R200	BALP + NO → BAL1 + NO2	4.00 × 10^−12^	4.00 × 10^−12^
205	R201	BAL1 + NO → BAL2 + NO2	4.00 × 10^−12^	4.00 × 10^−12^
206	R202	ADDC + NO → HO2 + NO2 + 0.320 HKET + 0.680 GLY + 0.680 OP2	2.70 × 10^−12^exp(360.00*/T*)	9.03 × 10^−12^
207	R203	MCTP + NO → MCTO + NO2	2.70 × 10^−12^exp(360.00*/T*)	9.03 × 10^−12^
208	R204	ORAP + NO → NO2 + GLY + HO2	4.00 × 10^−12^	4.00 × 10^−12^
209	R205	OLNN + NO → NO2 + HO2 + ONIT	4.00 × 10^−12^	4.00 × 10^−12^
210	R206	OLND + NO → 2.000 NO2 + 0.287 HCHO + 1.240 ALD + 0.464 KET	4.00 × 10^−12^	4.00 × 10^−12^
211	R207	ADCN + NO → 2.000 NO2 + GLY + OP2	2.70 × 10^−12^exp(360.00*/T*)	9.03 × 10^−12^
212	R208	XO2 + NO → NO2	4.00 × 10^−12^	4.00 × 10^−12^
213	R209	BAL2 + NO2 → ONIT	2.00 × 10^−11^	2.00 × 10^−11^
214	R210	CHO + NO2 → ONIT	2.00 × 10^−11^	2.00 × 10^−11^
215	R211	MCTO + NO2 → ONIT	2.08 × 10^−12^	2.08 × 10^−12^
216	R212	MO2 + HO2 → OP1	4.10 × 10^−13^exp(750.00*/T*)	5.07 × 10^−12^
217	R213	ETHP + HO2 → OP2	7.50 × 10^−13^exp(700.00*/T*)	7.85 × 10^−12^
218	R214	HC3P + HO2 → OP2	1.66 × 10^−13^exp(1300.00*/T*)	1.30 × 10^−11^
219	R215	HC5P + HO2 → OP2	1.66 × 10^−13^exp(1300.00*/T*)	1.30 × 10^−11^
220	R217	ETEP + HO2 → OP2	1.90 × 10^−13^exp(1300.00*/T*)	1.49 × 10^−11^
221	R218	OLTP + HO2 → OP2	1.66 × 10^−13^exp(1300.00*/T*)	1.30 × 10^−11^
222	R219	OLIP + HO2 → OP2	1.66 × 10^−13^exp(1300.00*/T*)	1.30 × 10^−11^
223	ROCARO32	BENP + HO2 → 0.602 OP2 + 0.398 VROCN1OXY6	2.91 × 10^−13^exp(1300.00*/T*)	2.28 × 10^−11^
224	ROCARO42	TOLP + HO2 → 0.720 OP2 + 0.281 VROCN1OXY6	2.91 × 10^−13^exp(1300.00*/T*)	2.28 × 10^−11^
225	ROCARO52	XYMP + HO2 → 0.048 OP2 + 0.675 OP3 + 0.277 VROCP0OXY4	2.91 × 10^−13^exp(1300.00*/T*)	2.28 × 10^−11^
226	ROCARO62	XYEP + HO2 → 0.085 OP2 + 0.634 OP3 + 0.281 VROCP0OXY4	2.91 × 10^−13^exp(1300.00*/T*)	2.28 × 10^−11^
227	R228	ISOP + HO2 → ISHP	2.05 × 10^−13^exp(1300.00*/T*)	1.60 × 10^−11^
228	R229	APIP1 + HO2 → OPB	1.50 × 10^−11^	1.50 × 10^−11^
229	TRP21	APIP2 + HO2 → HOM	1.50 × 10^−11^	1.50 × 10^−11^
230	TRP22	APINP1 + HO2 → TRPN	1.50 × 10^−11^	1.50 × 10^−11^
231	TRP23	APINP2 + HO2 → HOM	1.50 × 10^−11^	1.50 × 10^−11^
232	R230	LIMP1 + HO2 → OPB	1.50 × 10^−11^	1.50 × 10^−11^
233	TRP24	LIMP2 + HO2 → HOM	1.50 × 10^−11^	1.50 × 10^−11^
234	TRP25	LIMNP1 + HO2 → TRPN	1.50 × 10^−11^	1.50 × 10^−11^
235	TRP26	LIMNP2 + HO2 → HOM	1.50 × 10^−11^	1.50 × 10^−11^
236	TRP27	PINALP + HO2 → OPB	2.91 × 10^−13^exp(1300.00*/T*)	2.28 × 10^−11^
237	TRP28	LIMALP + HO2 → OPB	2.91 × 10^−13^exp(1300.00*/T*)	2.28 × 10^−11^
238	R231	ACO3 + HO2 → 0.440 HO + 0.440 MO2 + 0.150 ORA2 + 0.410 PAA	4.30 × 10^−13^exp(1040.00*/T*)	1.41 × 10^−11^
239	R232	RCO3 + HO2 → 0.440 HO + 0.440 ETHP + 0.150 ORA2 + 0.410 PAA	4.30 × 10^−13^exp(1040.00*/T*)	1.41 × 10^−11^
240	R233	ACTP + HO2 → 0.150 HO + 0.150 ACO3 + 0.150 HCHO + 0.850 OP2	1.15 × 10^−13^exp(1300.00*/T*)	9.00 × 10^−12^
241	R234	MEKP + HO2 → OP2	1.15 × 10^−13^exp(1300.00*/T*)	9.00 × 10^−12^
242	R235	KETP + HO2 → OP2	1.15 × 10^−13^exp(1300.00*/T*)	9.00 × 10^−12^
243	R236	MACP + HO2 → MAHP	1.82 × 10^−13^exp(1300.00*/T*)	1.42 × 10^−11^
244	R237	MCP + HO2 → MAHP	1.82 × 10^−13^exp(1300.00*/T*)	1.42 × 10^−11^
245	R238	MVKP + HO2 → OP2	2.91 × 10^−13^exp(1300.00*/T*)	2.28 × 10^−11^
246	R239	UALP + HO2 → OP2	2.91 × 10^−13^exp(1300.00*/T*)	2.28 × 10^−11^
247	R240	ADDC + HO2 → OP2	3.75 × 10^−13^exp(980.00*/T*)	1.00 × 10^−11^
248	R241	CHO + HO2 → CSL	1.00 × 10^−11^	1.00 × 10^−11^
249	R242	MCTP + HO2 → OP2	3.75 × 10^−13^exp(980.00*/T*)	1.00 × 10^−11^
250	R243	ORAP + HO2 → OP2	1.15 × 10^−13^exp(1300.00*/T*)	9.00 × 10^−12^
251	R244	OLNN + HO2 → ONIT	1.66 × 10^−13^exp(1300.00*/T*)	1.30 × 10^−11^
252	R245	OLND + HO2 → ONIT	1.66 × 10^−13^exp(1300.00*/T*)	1.30 × 10^−11^
253	R246	ADCN + HO2 → OP2	3.75 × 10^−13^exp(980.00*/T*)	1.00 × 10^−11^
254	R247	XO2 + HO2 → OP2	1.66 × 10^−13^exp(1300.00*/T*)	1.30 × 10^−11^
255	R248	MO2 + MO2 → 0.740 HO2 + 1.370 HCHO + 0.630 MOH	9.50 × 10^−14^exp(390.00*/T*)	3.51 × 10^−13^
256	R249	ETHP + MO2 → HO2 + 0.750 HCHO + 0.750 ACD + 0.250 MOH + 0.250 EOH	1.18 × 10^−13^exp(158.00*/T*)	2.00 × 10^−13^
257	R250	HC3P + MO2 → 0.894 HO2 + 0.080 MO2 + 0.026 ETHP + 0.026 XO2 + 0.827 HCHO + 0.198 ALD + 0.497 KET + 0.050 GLY + 0.250 MOH + 0.250 ROH	9.46 × 10^−14^exp(431.00*/T*)	4.02 × 10^−13^
258	R251	HC5P + MO2 → 0.842 HO2 + 0.018 MO2 + 0.140 ETHP + 0.191 XO2 + 0.777 HCHO + 0.251 ALD + 0.618 KET + 0.250 MOH + 0.250 ROH	1.00 × 10^−13^exp(467.00*/T*)	4.79 × 10^−13^
259	R253	ETEP + MO2 → HO2 + 1.950 HCHO + 0.150 ALD + 0.250 MOH + 0.250 ETEG	1.71 × 10^−13^exp(708.00*/T*)	1.84 × 10^−12^
260	R254	OLTP + MO2 → HO2 + 1.500 HCHO + 0.705 ALD + 0.045 KET + 0.250 MOH + 0.250 ROH	1.46 × 10^−13^exp(708.00*/T*)	1.57 × 10^−12^
261	R255	OLIP + MO2 → HO2 + 0.750 HCHO + 1.280 ALD + 0.218 KET + 0.250 MOH + 0.250 ROH	9.18 × 10^−14^exp(708.00*/T*)	9.87 × 10^−13^
262	ROCARO35	BENP + MO2 → 0.680 HCHO + 1.370 HO2 + 0.320 MOH + 0.000 BALD + GLY + 0.500 FURANONE + 0.250 DCB2 + 0.250 DCB3	3.56 × 10^−14^exp(708.00*/T*)	3.83 × 10^−13^
263	ROCARO45	TOLP + MO2 → 0.680 HCHO + 1.285 HO2 + 0.320 MOH + 0.085 BALD + 0.549 GLY + 0.366 MGLY + 0.366 FURANONE + 0.549 DCB1	3.56 × 10^−14^exp(708.00*/T*)	3.83 × 10^−13^
264	ROCARO55	XYMP + MO2 → 0.680 HCHO + 1.322 HO2 + 0.320 MOH + 0.048 BALD + 0.704 GLY + 0.247 MGLY + 0.352 FURANONE + 0.600 DCB2	3.56 × 10^−14^exp(708.00*/T*)	3.83 × 10^−13^
265	ROCARO65	XYEP + MO2 → 0.680 HCHO + 1.285 HO2 + 0.320 MOH + 0.085 BALD + 0.549 GLY + 0.366 MGLY + 0.457 FURANONE + 0.457 DCB2	3.56 × 10^−14^exp(708.00*/T*)	3.83 × 10^−13^
266	R264	ISOP + MO2 → HO2 + 1.310 HCHO + 0.159 MACR + 0.250 MVK + 0.250 MOH + 0.250 ROH + 0.023 ALD + 0.018 GLY + 0.016 HKET	3.40 × 10^−14^exp(221.00*/T*)	7.14 × 10^−14^
267	R265	APIP1 + MO2 → HO2 + 0.680 HCHO + 0.600 PINAL + 0.070 KET + 0.320 MOH + 0.250 ROH	3.56 × 10^−14^exp(708.00*/T*)	3.83 × 10^−13^
268	TRP29	APIP2 + MO2 → HO2 + 0.750 HCHO + 0.250 MOH + HOM	1.00 × 10^−10^	1.00 × 10^−10^
269	TRP30	APINP1 + MO2 → 0.370 HO2 + 0.860 NO2 + 0.680 HCHO + 0.860 PINAL + 0.320 MOH + 0.140 TRPN	3.56 × 10^−14^exp(708.00*/T*)	3.83 × 10^−13^
270	TRP31	APINP2 + MO2 → 0.750 HO2 + 0.750 NO2 + 0.250 MOH + 0.750 HCHO + HOM	1.00 × 10^−10^	1.00 × 10^−10^
271	R266	LIMP1 + MO2 → HO2 + HCHO + 0.420 LIMAL + 0.300 KET + 0.320 MOH + 0.270 ROH	3.56 × 10^−14^exp(708.00*/T*)	3.83 × 10^−13^
272	TRP32	LIMP2 + MO2 → HO2 + 0.750 HCHO + 0.250 MOH + HOM	1.00 × 10^−10^	1.00 × 10^−10^
273	TRP33	LIMNP1 + MO2 → 0.370 HO2 + 0.680 HCHO + 0.700 LIMAL + 0.700 NO2 + 0.320 MOH + 0.300 TRPN	3.56 × 10^−14^exp(708.00*/T*)	3.83 × 10^−13^
274	TRP34	LIMNP2 + MO2 → 0.750 HO2 + 0.750 HCHO + 0.750 NO2 + 0.250 MOH + HOM	1.00 × 10^−10^	1.00 × 10^−10^
275	R267	ACO3 + MO2 → 0.900 HO2 + 0.900 MO2 + HCHO + 0.100 ORA2	2.00 × 10^−11^exp(500.00*/T*)	1.07 × 10^−10^
276	R268	RCO3 + MO2 → 0.900 HO2 + 0.900 MO2 + HCHO + 0.100 ORA2	2.00 × 10^−11^exp(500.00*/T*)	1.07 × 10^−10^
277	R269	ACTP + MO2 → 0.500 HO2 + 0.500 ACO3 + 1.500 HCHO + 0.250 MOH + 0.250 ROH + 0.125 ORA2	7.50 × 10^−13^exp(500.00*/T*)	4.01 × 10^−12^
278	R270	MEKP + MO2 → 0.834 HO2 + HCHO + 0.334 DCB1 + 0.250 MOH + 0.250 ROH	6.91 × 10^−13^exp(508.00*/T*)	3.80 × 10^−12^
279	R271	KETP + MO2 → HO2 + 0.750 HCHO + 0.500 DCB1 + 0.250 MOH + 0.250 ROH	6.91 × 10^−13^exp(508.00*/T*)	3.80 × 10^−12^
280	R272	MACP + MO2 → 0.500 HO2 + 0.269 ACO3 + 0.500 CO + 1.660 HCHO + 0.067 ORA2 + 0.250 MO2 + 0.250 MOH + 0.250 ROH	3.40 × 10^−14^exp(221.00*/T*)	7.14 × 10^−14^
281	R273	MCP + MO2 → NO2 + HO2 + 1.500 HCHO + 0.500 HKET + 0.250 MOH + 0.250 ROH	3.40 × 10^−14^exp(221.00*/T*)	7.14 × 10^−14^
282	R274	MVKP + MO2 → HO2 + 1.160 ACO3 + 1.160 XO2 + 1.500 HCHO + 1.750 ALD + 0.500 MGLY + 0.250 MOH + 0.250 ROH + 0.292 ORA2	8.37 × 10^−14^	8.37 × 10^−14^
283	R275	UALP + MO2 → HO2 + 0.305 CO + 0.773 HCHO + 0.203 ALD + 0.525 KET + 0.135 GLY + 0.105 MGLY + 0.250 MOH + 0.250 ROH	3.40 × 10^−14^exp(221.00*/T*)	7.14 × 10^−14^
284	R276	BALP + MO2 → HO2 + BAL1 + HCHO	3.56 × 10^−14^exp(708.00*/T*)	3.83 × 10^−13^
285	R277	BAL1 + MO2 → HO2 + BAL2 + HCHO	3.56 × 10^−14^exp(708.00*/T*)	3.83 × 10^−13^
286	R278	ADDC + MO2 → 2.000 HO2 + HCHO + 0.320 HKET + 0.680 GLY + 0.680 OP2	3.56 × 10^−14^exp(708.00*/T*)	3.83 × 10^−13^
287	R279	MCTP + MO2 → HO2 + MCTO + HCHO	3.56 × 10^−14^exp(708.00*/T*)	3.83 × 10^−13^
288	R280	ORAP + MO2 → HCHO + HO2 + GLY	7.50 × 10^−13^exp(500.00*/T*)	4.01 × 10^−12^
289	R281	OLNN + MO2 → 2.000 HO2 + HCHO + ONIT	1.60 × 10^−13^exp(708.00*/T*)	1.72 × 10^−12^
290	R282	OLND + MO2 → 0.500 HO2 + 0.500 NO2 + 0.965 HCHO + 0.930 ALD + 0.348 KET + 0.250 MOH + 0.250 ROH + 0.500 ONIT	9.68 × 10^−14^exp(708.00*/T*)	1.04 × 10^−12^
291	R283	ADCN + MO2 → HO2 + 0.700 NO2 + HCHO + 0.700 GLY + 0.700 OP2 + 0.300 ONIT	3.56 × 10^−14^	3.56 × 10^−14^
292	R284	XO2 + MO2 → HO2 + HCHO	5.99 × 10^−15^exp(1510.00*/T*)	9.48 × 10^−13^
293	R285	ETHP + ACO3 → 0.500 HO2 + 0.500 MO2 + ACD + 0.500 ORA2	1.03 × 10^−12^exp(211.00*/T*)	2.09 × 10^−12^
294	R286	HC3P + ACO3 → 0.394 HO2 + 0.580 MO2 + 0.026 ETHP + 0.026 XO2 + 0.130 HCHO + 0.273 ALD + 0.662 KET + 0.067 GLY + 0.500 ORA2	6.90 × 10^−13^exp(460.00*/T*)	3.23 × 10^−12^
295	R287	HC5P + ACO3 → 0.342 HO2 + 0.518 MO2 + 0.140 ETHP + 0.191 XO2 + 0.042 HCHO + 0.381 ALD + 0.824 KET + 0.500 ORA2	5.59 × 10^−13^exp(522.00*/T*)	3.22 × 10^−12^
296	R289	ETEP + ACO3 → 0.500 HO2 + 0.500 MO2 + 1.600 HCHO + 0.200 ALD + 0.500 ORA2	9.48 × 10^−13^exp(765.00*/T*)	1.23 × 10^−11^
297	R290	OLTP + ACO3 → 0.500 HO2 + 0.500 MO2 + HCHO + 0.940 ALD + 0.060 KET + 0.500 ORA2	8.11 × 10^−13^exp(765.00*/T*)	1.06 × 10^−11^
298	R291	OLIP + ACO3 → 0.500 HO2 + 0.500 MO2 + 1.710 ALD + 0.290 KET + 0.500 ORA2	5.09 × 10^−13^exp(765.00*/T*)	6.62 × 10^−12^
299	ROCARO36	BENP + ACO3 → 0.700 MO2 + HO2 + 0.300 ORA2 + 0.000 BALD + GLY + 0.500 FURANONE + 0.250 DCB2 + 0.250 DCB3	7.40 × 10^−13^exp(765.00*/T*)	9.63 × 10^−12^
300	ROCARO46	TOLP + ACO3 → 0.700 MO2 + 0.915 HO2 + 0.300 ORA2 + 0.085 BALD + 0.549 GLY + 0.366 MGLY + 0.366 FURANONE + 0.549 DCB1	7.40 × 10^−13^exp(765.00*/T*)	9.63 × 10^−12^
301	ROCARO56	XYMP + ACO3 → 0.700 MO2 + 0.952 HO2 + 0.300 ORA2 + 0.048 BALD + 0.704 GLY + 0.247 MGLY + 0.352 FURANONE + 0.600 DCB2	7.40 × 10^−13^exp(765.00*/T*)	9.63 × 10^−12^
302	ROCARO66	XYEP + ACO3 → 0.700 MO2 + 0.915 HO2 + 0.300 ORA2 + 0.085 BALD + 0.549 GLY + 0.366 MGLY + 0.457 FURANONE + 0.457 DCB2	7.40 × 10^−13^exp(765.00*/T*)	9.63 × 10^−12^
303	R300	ISOP + ACO3 → 0.500 HO2 + 0.500 MO2 + 1.048 HCHO + 0.219 MACR + 0.305 MVK + 0.500 ORA2	8.40 × 10^−14^exp(221.00*/T*)	1.76 × 10^−13^
304	R301	APIP1 + ACO3 → 0.630 HO2 + 0.700 MO2 + 0.600 PINAL + 0.300 ORA2 + 0.070 KET + 0.250 ROH	7.40 × 10^−13^exp(765.00*/T*)	9.63 × 10^−12^
305	TRP35	APIP2 + ACO3 → 0.500 HO + 0.500 MO2 + 0.500 ORA2 + HOM	1.00 × 10^−10^	1.00 × 10^−10^
306	TRP36	APINP1 + ACO3 → 0.860 NO2 + 0.140 TRPN + 0.860 PINAL + 0.700 MO2 + 0.300 ORA2	7.40 × 10^−13^exp(765.00*/T*)	9.63 × 10^−12^
307	TRP37	APINP2 + ACO3 → 0.500 NO2 + 0.500 MO2 + 0.500 ORA2 + HOM	1.00 × 10^−10^	1.00 × 10^−10^
308	R302	LIMP1 + ACO3 → 0.630 HO2 + 0.700 MO2 + 0.420 LIMAL + 0.300 KET + 0.300 ORA2 + 0.320 HCHO + 0.270 ROH	7.40 × 10^−13^exp(765.00*/T*)	9.63 × 10^−12^
309	TRP38	LIMP2 + ACO3 → 0.500 HO + 0.500 MO2 + 0.500 ORA2 + HOM	1.00 × 10^−10^	1.00 × 10^−10^
310	TRP39	LIMNP1 + ACO3 → 0.700 NO2 + 0.700 LIMAL + 0.300 TRPN + 0.700 MO2 + 0.300 ORA2	7.40 × 10^−13^exp(765.00*/T*)	9.63 × 10^−12^
311	TRP40	LIMNP2 + ACO3 → 0.500 MO2 + 0.500 NO2 + 0.500 ORA2 + HOM	1.00 × 10^−10^	1.00 × 10^−10^
312	R303	ACO3 + ACO3 → 2.000 MO2	2.50 × 10^−12^exp(500.00*/T*)	1.34 × 10^−11^
313	R304	RCO3 + ACO3 → MO2 + ETHP	2.50 × 10^−12^exp(500.00*/T*)	1.34 × 10^−11^
314	R305	ACTP + ACO3 → 0.500 MO2 + 0.500 ACO3 + HCHO + 0.750 ORA2	7.51 × 10^−13^exp(565.00*/T*)	5.00 × 10^−12^
315	R306	MEKP + ACO3 → 0.330 HO2 + 0.500 MO2 + 0.330 HCHO + 0.334 DCB1 + 0.500 ORA2	7.51 × 10^−13^exp(565.00*/T*)	5.00 × 10^−12^
316	R307	KETP + ACO3 → 0.500 HO2 + 0.500 MO2 + 0.500 DCB1 + 0.500 ORA2	7.51 × 10^−13^exp(565.00*/T*)	5.00 × 10^−12^
317	R308	MACP + ACO3 → 0.635 ORA2 + 0.500 MO2 + 0.269 ACO3 + 0.500 CO + HCHO	8.40 × 10^−14^exp(221.00*/T*)	1.76 × 10^−13^
318	R309	MCP + ACO3 → NO2 + 0.500 HO2 + HCHO + 0.500 HKET + 0.500 MO2 + 0.500 ORA2	8.40 × 10^−14^exp(221.00*/T*)	1.76 × 10^−13^
319	R310	MVKP + ACO3 → 0.500 HO2 + 0.500 MO2 + 1.160 ACO3 + 1.160 XO2 + HCHO + 2.300 ALD + 0.500 MGLY + 1.083 ORA2	1.68 × 10^−12^exp(500.00*/T*)	8.99 × 10^−12^
320	R311	UALP + ACO3 → 0.500 HO2 + 0.500 MO2 + 0.500 CO + 0.030 HCHO + 0.270 ALD + 0.700 KET + 0.180 GLY + 0.105 MGLY + 0.500 ORA2	1.68 × 10^−12^exp(500.00*/T*)	8.99 × 10^−12^
321	R312	BALP + ACO3 → MO2 + BAL1	7.40 × 10^−13^exp(765.00*/T*)	9.63 × 10^−12^
322	R313	BAL1 + ACO3 → MO2 + BAL2	7.40 × 10^−13^exp(765.00*/T*)	9.63 × 10^−12^
323	R314	ADDC + ACO3 → 2.000 HO2 + MO2 + 0.320 HKET + 0.680 GLY + 0.680 OP2	7.40 × 10^−13^exp(708.00*/T*)	7.95 × 10^−12^
324	R315	MCTP + ACO3 → HO2 + MO2 + MCTO	7.40 × 10^−13^exp(708.00*/T*)	7.95 × 10^−12^
325	R316	ORAP + ACO3 → MO2 + GLY	7.51 × 10^−13^exp(565.00*/T*)	5.00 × 10^−12^
326	R317	OLNN + ACO3 → HO2 + MO2 + ONIT	8.85 × 10^−13^exp(765.00*/T*)	1.15 × 10^−11^
327	R318	OLND + ACO3 → 0.500 MO2 + NO2 + 0.287 HCHO + 1.240 ALD + 0.464 KET + 0.500 ORA2	5.37 × 10^−13^exp(765.00*/T*)	6.99 × 10^−12^
328	R319	ADCN + ACO3 → HO2 + MO2 + 0.700 NO2 + 0.700 GLY + 0.700 OP2 + 0.300 ONIT	7.40 × 10^−13^exp(708.00*/T*)	7.95 × 10^−12^
329	R320	XO2 + ACO3 → MO2	3.40 × 10^−14^exp(1560.00*/T*)	6.37 × 10^−12^
330	R321	RCO3 + RCO3 → 2.000 ETHP	2.50 × 10^−12^exp(500.00*/T*)	1.34 × 10^−11^
331	R322	MO2 + NO3 → HO2 + HCHO + NO2	1.20 × 10^−12^	1.20 × 10^−12^
332	R323	ETHP + NO3 → HO2 + NO2 + ACD	1.20 × 10^−12^	1.20 × 10^−12^
333	R324	HC3P + NO3 → 0.254 HO2 + 0.140 MO2 + 0.092 XO2 + 0.503 ETHP + NO2 + 0.519 ACD + 0.147 ALD + 0.075 MEK + 0.095 ACT	1.20 × 10^−12^	1.20 × 10^−12^
334	R325	HC5P + NO3 → 0.488 HO2 + 0.055 MO2 + 0.280 ETHP + 0.485 XO2 + NO2 + 0.024 HCHO + 0.241 ALD + 0.060 KET + 0.063 MEK + 0.247 ACT + 0.048 ACD + 0.275 HKET	1.20 × 10^−12^	1.20 × 10^−12^
335	R327	ETEP + NO3 → HO2 + NO2 + 1.600 HCHO + 0.200 ALD	1.20 × 10^−12^	1.20 × 10^−12^
336	R328	OLTP + NO3 → 0.470 ALD + 0.790 HCHO + 0.790 HO2 + NO2 + 0.180 MEK + 0.020 ACD + 0.090 ACT	1.20 × 10^−12^	1.20 × 10^−12^
337	R329	OLIP + NO3 → 0.860 HO2 + 0.720 ALD + 0.110 KET + NO2 + 0.200 ACT + 0.850 ACD + 0.040 HKET	1.20 × 10^−12^	1.20 × 10^−12^
338	ROCARO34	BENP + NO3 → NO2 + HO2 + 0.000 BALD + GLY + 0.500 FURANONE + 0.250 DCB2 + 0.250 DCB3	2.30 × 10^−12^	2.30 × 10^−12^
339	ROCARO44	TOLP + NO3 → NO2 + 0.915 HO2 + 0.085 BALD + 0.549 GLY + 0.366 MGLY + 0.366 FURANONE + 0.549 DCB1	2.30 × 10^−12^	2.30 × 10^−12^
340	ROCARO54	XYMP + NO3 → NO2 + 0.952 HO2 + 0.048 BALD + 0.704 GLY + 0.247 MGLY + 0.352 FURANONE + 0.600 DCB2	2.30 × 10^−12^	2.30 × 10^−12^
341	ROCARO64	XYEP + NO3 → NO2 + 0.915 HO2 + 0.085 BALD + 0.549 GLY + 0.366 MGLY + 0.457 FURANONE + 0.457 DCB2	2.30 × 10^−12^	2.30 × 10^−12^
342	R338	ISOP + NO3 → HO2 + NO2 + 0.750 HCHO + 0.318 MACR + 0.500 MVK + 0.024 GLY + 0.033 HKET + 0.031 ALD	1.20 × 10^−12^	1.20 × 10^−12^
343	R339	APIP1 + NO3 → HO2 + NO2 + ALD + KET	1.20 × 10^−12^	1.20 × 10^−12^
344	R340	LIMP1 + NO3 → HO2 + NO2 + 0.385 OLI + 0.385 HCHO + 0.615 MACR	1.20 × 10^−12^	1.20 × 10^−12^
345	R341	ACO3 + NO3 → MO2 + NO2	4.00 × 10^−12^	4.00 × 10^−12^
346	R342	RCO3 + NO3 → ETHP + NO2	4.00 × 10^−12^	4.00 × 10^−12^
347	R343	ACTP + NO3 → ACO3 + NO2 + HCHO	1.20 × 10^−12^	1.20 × 10^−12^
348	R344	MEKP + NO3 → 0.670 HO2 + NO2 + 0.330 HCHO + 0.670 DCB1	1.20 × 10^−12^	1.20 × 10^−12^
349	R345	KETP + NO3 → HO2 + NO2 + DCB1	1.20 × 10^−12^	1.20 × 10^−12^
350	R346	MACP + NO3 → HCHO + 0.538 ACO3 + CO + NO2	1.20 × 10^−12^	1.20 × 10^−12^
351	R347	MCP + NO3 → NO2 + HO2 + HCHO + HKET	1.20 × 10^−12^	1.20 × 10^−12^
352	R348	MVKP + NO3 → 0.300 HO2 + 0.700 ACO3 + 0.700 XO2 + NO2 + 0.300 HCHO + 0.700 ALD + 0.300 MGLY	2.50 × 10^−12^	2.50 × 10^−12^
353	R349	UALP + NO3 → HO2 + NO2 + 0.610 CO + 0.030 HCHO + 0.270 ALD + 0.700 KET + 0.180 GLY + 0.210 MGLY	2.50 × 10^−12^	2.50 × 10^−12^
354	R350	BALP + NO3 → BAL1 + NO2	2.50 × 10^−12^	2.50 × 10^−12^
355	R351	BAL1 + NO3 → BAL2 + NO2	2.50 × 10^−12^	2.50 × 10^−12^
356	R352	ADDC + NO3 → HO2 + NO2 + 0.320 HKET + 0.680 GLY + 0.680 OP2	1.20 × 10^−12^	1.20 × 10^−12^
357	R353	MCTP + NO3 → NO2 + MCTO	1.20 × 10^−12^	1.20 × 10^−12^
358	R354	ORAP + NO3 → NO2 + GLY + HO2	1.20 × 10^−12^	1.20 × 10^−12^
359	R355	OLNN + NO3 → HO2 + NO2 + ONIT	1.20 × 10^−12^	1.20 × 10^−12^
360	R356	OLND + NO3 → 2.000 NO2 + 0.287 HCHO + 1.240 ALD + 0.464 KET	1.20 × 10^−12^	1.20 × 10^−12^
361	R357	ADCN + NO3 → 2.000 NO2 + GLY + OP2	1.20 × 10^−12^	1.20 × 10^−12^
362	R358	OLNN + OLNN → HO2 + 2.000 ONIT	7.00 × 10^−14^exp(1000.00*/T*)	2.00 × 10^−12^
363	R359	OLNN + OLND → 0.500 HO2 + 0.500 NO2 + 0.202 HCHO + 0.640 ALD + 0.149 KET + 1.500 ONIT	4.25 × 10^−14^exp(1000.00*/T*)	1.22 × 10^−12^
364	R360	OLND + OLND → NO2 + 0.504 HCHO + 1.210 ALD + 0.285 KET + ONIT	2.96 × 10^−14^exp(1000.00*/T*)	8.47 × 10^−13^
365	R361	XO2 + NO3 → NO2	1.20 × 10^−12^	1.20 × 10^−12^
366	R362	XO2 + RCO3 → ETHP	2.50 × 10^−12^exp(500.00*/T*)	1.34 × 10^−11^
367	R363	XO2 + XO2 →	7.13 × 10^−17^exp(2950.00*/T*)	1.41 × 10^−12^
368	TRP41	APIP2 + APIP1 → 0.960 HOM + 0.480 ROH + 0.480 PINAL + 0.480 HO + 0.480 HO2 + 0.040 ELHOM	1.00 × 10^−10^	1.00 × 10^−10^
369	TRP42	APIP2 + LIMP1 → 0.960 HOM + 0.480 ROH + 0.480 LIMAL + 0.480 HO + 0.480 HO2 + 0.040 ELHOM	1.00 × 10^−10^	1.00 × 10^−10^
370	TRP43	APIP2 + ISOP → 0.960 HOM + 0.480 ROH + 0.480 HCHO + 0.480 MVK + 0.480 HO + 0.480 HO2 + 0.040 ELHOM	1.00 × 10^−10^	1.00 × 10^−10^
371	TRP44	LIMP2 + APIP1 → 0.960 HOM + 0.480 ROH + 0.480 PINAL + 0.480 HO + 0.480 HO2 + 0.040 ELHOM	1.00 × 10^−10^	1.00 × 10^−10^
372	TRP45	LIMP2 + LIMP1 → 0.960 HOM + 0.480 ROH + 0.480 LIMAL + 0.480 HO + 0.480 HO2 + 0.040 ELHOM	1.00 × 10^−10^	1.00 × 10^−10^
373	TRP46	LIMP2 + ISOP → 0.960 HOM + 0.480 ROH + 0.480 HCHO + 0.480 MVK + 0.480 HO + 0.480 HO2 + 0.040 ELHOM	1.00 × 10^−10^	1.00 × 10^−10^
374	TRP47	APINP2 + APIP1 → 0.960 HOM + 0.480 ROH + 0.480 PINAL + 0.480 NO2 + 0.480 HO2 + 0.040 ELHOM	1.00 × 10^−10^	1.00 × 10^−10^
375	TRP48	APINP2 + LIMP1 → 0.960 HOM + 0.480 ROH + 0.480 LIMAL + 0.480 NO2 + 0.480 HO2 + 0.040 ELHOM	1.00 × 10^−10^	1.00 × 10^−10^
376	TRP49	APINP2 + ISOP → 0.960 HOM + 0.480 ROH + 0.480 HCHO + 0.480 MVK + 0.480 NO2 + 0.480 HO2 + 0.040 ELHOM	1.00 × 10^−10^	1.00 × 10^−10^
377	TRP50	LIMNP2 + APIP1 → 0.960 HOM + 0.480 ROH + 0.480 PINAL + 0.480 NO2 + 0.480 HO2 + 0.040 ELHOM	1.00 × 10^−10^	1.00 × 10^−10^
378	TRP51	LIMNP2 + LIMP1 → 0.960 HOM + 0.480 ROH + 0.480 LIMAL + 0.480 NO2 + 0.480 HO2 + 0.040 ELHOM	1.00 × 10^−10^	1.00 × 10^−10^
379	TRP52	LIMNP2 + ISOP → 0.960 HOM + 0.480 ROH + 0.480 HCHO + 0.480 MVK + 0.480 NO2 + 0.480 HO2 + 0.040 ELHOM	1.00 × 10^−10^	1.00 × 10^−10^
380	SA14	IEPOX + HO → HO	5.78 × 10^−11^exp(−400.00*/T*)	1.51 × 10^−11^
381	R001c	VROCIOXY + HO → 0.852 ETHP + 0.149 ASOATJ	6.89 × 10^−12^	6.89 × 10^−12^
382	R002c	SLOWROC + HO → ETHP + 0.001 ASOATJ	6.55 × 10^−14^	6.55 × 10^−14^
383	T17	ACRO + HO → 0.570 MACP + 0.430 MCP	8.00 × 10^−12^exp(380.00*/T*)	2.86 × 10^−11^
384	T18	ACRO + O3 → 0.840 CO + 0.560 HO2 + 0.280 HO + 0.720 HCHO + 0.620 GLY	2.90 × 10^−19^	2.90 × 10^−19^
385	T19	ACRO + NO3 → 0.680 HCHO + 0.320 MACP + 0.680 XO2 + 0.680 MGLY + 0.320 HNO3 + 0.680 NO2	3.40 × 10^−15^	3.40 × 10^−15^
386	T20	ACRO → CO + 0.477 HO2 + 0.250 ETE + 0.354 ACO3 + 0.204 HO + 0.150 HCHO + 0.027 MO2	φ from MVK (Atkinson et al., 2006; Gierczak et al., 1997); σ from Sander et al. (2006) as implemented by Hutzell et al. (2012)	Not available
387	T10	BDE13 + HO → 0.667 BDE13P + 0.333 UALD + 0.333 HO2	1.48 × 10^−11^exp(448.00*/T*)	6.65 × 10^−11^
388	T10a	BDE13P + NO → 0.968 HO2 + 0.968 NO2 + 0.895 ACRO + 0.895 HCHO + 0.072 FURAN + 0.032 ONIT	9.05 × 10^−12^	9.05 × 10^−12^
389	T10b	BDE13P + NO3 → HO2 + NO2 + 0.925 ACRO + 0.925 HCHO + 0.075 FURAN	2.30 × 10^−12^	2.30 × 10^−12^
390	T10c	BDE13P + HO2 → OP2	1.61 × 10^−11^	1.61 × 10^−11^
391	T10d	BDE13P + MO2 → 0.320 MOH + 1.143 HCHO + 0.870 HO2 + 0.463 ACRO + 0.250 OLT + 0.231 MVK + 0.037 FURAN + 0.019 UALD	2.39 × 10^−12^	2.39 × 10^−12^
392	T10e	BDE13P + ACO3 → 0.700 MO2 + 0.300 ORA2 + 0.800 HO2 + 0.740 ACRO + 0.740 HCHO + 0.185 MVK + 0.060 FURAN + 0.015 UALD	1.37 × 10^−11^	1.37 × 10^−11^
393	T11	BDE13 + O3 → 0.620 ACRO + 0.630 CO + 0.420 HO2 + 0.080 HO + 0.830 HCHO + 0.170 ETE	1.34 × 10^−14^exp(−2283.00*/T*)	6.33 × 10^−18^
394	T12	BDE13 + NO3 → 0.900 OLNN + 0.100 OLND + 0.900 ACRO	1.00 × 10^−13^	1.00 × 10^−13^
395	R003c	FURAN + HO → 0.490 DCB1 + 0.490 HO2 + 0.510 FURANO2	5.01 × 10^−11^	5.01 × 10^−11^
396	R004c	FURANO2 + NO → 0.080 ONIT + 0.920 NO2 + 0.920 FURANONE + 0.750 HO2 + 0.170 MO2	2.70 × 10^−12^exp(360.00*/T*)	9.03 × 10^−12^
397	R005c	FURANO2 + HO2 → 0.600 OP2 + 0.400 FURANONE + 0.400 HO + 0.320 HO2 + 0.080 MO2	3.75 × 10^−13^exp(980.00*/T*)	1.00 × 10^−11^
398	R006c	FURANONE + HO → 0.650 KET + 0.310 GLY + 0.660 HO2 + 0.340 MO2 + 0.430 CO + 0.040 ASOATJ	4.40 × 10^−11^	4.40 × 10^−11^
399	R007c	FURAN + O3 → 0.020 HO + ALD	3.43 × 10^−17^	3.43 × 10^−17^
400	R008c	FURAN + NO3 → NO2 + 0.800 DCB1 + 0.200 DCB3	8.99 × 10^−12^	8.99 × 10^−12^
401	R010c	PROG + HO → 0.613 HKET + 0.387 ALD + HO2	1.20 × 10^−11^	1.20 × 10^−11^
402	R011c	SESQ + NO3 → SESQNRO2	1.90 × 10^−11^	1.90 × 10^−11^
403	R012c	SESQNRO2 + HO2 → VROCP0OXY4	2.84 × 10^−13^exp(1300.00*/T*)	2.22 × 10^−11^
404	R013c	SESQNRO2 + NO → VROCP3OXY2 + 2.000 NO2	2.70 × 10^−12^exp(360.00*/T*)	9.03 × 10^−12^
405	R014c	SESQNRO2 + NO3 → VROCP3OXY2 + 2.000 NO2	2.30 × 10^−12^	2.30 × 10^−12^
406	R015c	SESQ + O3 → 0.982 VROCP3OXY2 + 0.018 VROCN2OXY2	1.20 × 10^−14^	1.20 × 10^−14^
407	R016c	SESQ + HO → SESQRO2	1.97 × 10^−10^	1.97 × 10^−10^
408	R017c	SESQRO2 + HO2 → VROCP0OXY2	2.84 × 10^−13^exp(1300.00*/T*)	2.22 × 10^−11^
409	R019c	SESQRO2 + NO3 → VROCP3OXY2	2.30 × 10^−12^	2.30 × 10^−12^
410	R020c	SESQRO2 + NO → 0.247 VROCP1OXY3 + 0.753 VROCP3OXY2 + 0.753 NO2	2.70 × 10^−12^exp(360.00*/T*)	9.03 × 10^−12^
411	HET_GLY	GLY → AGLYJ	$\gamma =2.9\times 10^{-3}$, based on Liggio et al. (2005) as implemented by Pye et al. (2015)	Not available^b^
412	HET_MGLY	MGLY → AGLYJ	$\gamma =2.9\times 10^{-3}$, based on Liggio et al. (2005) as implemented by Pye et al. (2015)	Not available^b^
413	HET_N2O5	N2O5 → 2.000 HNO3	Davis et al. (2008) Eq. (15)	Not available^b^
414	HET_NO2	NO2 → 0.500 HONO + 0.500 HNO3	$\nu \gamma =4\times 10^{-4}\text{m}\;{\text{s}}^{-1}$ (Vogel et al., 2003)	Not available^b^
415	HAL_Ozone^e^	O3 →	min(6.701 × 10^−11^exp(1.074 × 10^+1^P) + 3.415 × 10^−08^exp(−6.713 × 10^−1^P), 2.000 × 10^−06^)	2.00 × 10^−6^
416	HET_IEPOX^g^	IEPOX → IEPOXP	Uptake coefficient calculated based on particle composition following Pye et al. (2013) with parameter updates of Pye et al. (2017)	Not applicable^b^
417	HET_ISO3TET	IEPOXP → AISO3NOSJ	Ratio of 2-methyltetrols + IEPOX-derived organonitrate formation rates to total condensed-phase reaction rate	Not applicable
418	HET_IEPOXOS	IEPOXP + ASO4J → AISO3OSJ	Ratio of organosulfate formation rate to total IEPOX condensed-phase reaction rate	Not applicable
419	ROCALK1c	VROCP6ALK + HO → VROCP6ALKP	1.53 × 10^−11^	1.53 × 10^−11^
420	ROCALK2c	VROCP5ALK + HO → VROCP5ALKP	1.68 × 10^−11^	1.68 × 10^−11^
421	ROCALK3c	VROCP4ALK + HO → VROCP4ALKP	2.24 × 10^−11^	2.24 × 10^−11^
422	ROCALK4c	VROCP3ALK + HO → VROCP3ALKP	2.67 × 10^−11^	2.67 × 10^−11^
423	ROCALK5c	VROCP2ALK + HO → VROCP2ALKP	3.09 × 10^−11^	3.09 × 10^−11^
424	ROCALK6c	VROCP1ALK + HO → VROCP1ALKP	3.38 × 10^−11^	3.38 × 10^−11^
425	HC1001	HC10 + HO → HC10P	1.10 × 10^−11^	1.10 × 10^−11^
426	ROCALK7c	VROCP6ALKP + NO → 0.720 VROCP6ALKP2 + 0.280 VROCP4OXY2 + 0.720 NO2	2.70 × 10^−12^exp(360.00*/T*)	9.03 × 10^−12^
427	ROCALK8c	VROCP5ALKP + NO → 0.720 VROCP5ALKP2 + 0.280 VROCP3OXY2 + 0.720 NO2	2.70 × 10^−12^exp(360.00*/T*)	9.03 × 10^−12^
428	ROCALK9c	VROCP4ALKP + NO → 0.720 VROCP4ALKP2 + 0.280 VROCP2OXY2 + 0.720 NO2	2.70 × 10^−12^exp(360.00*/T*)	9.03 × 10^−12^
429	ROCALK10c	VROCP3ALKP + NO → 0.720 VROCP3ALKP2 + 0.280 VROCP1OXY1 + 0.720 NO2	2.70 × 10^−12^exp(360.00*/T*)	9.03 × 10^−12^
430	ROCALK11c	VROCP2ALKP + NO → 0.720 VROCP2ALKP2 + 0.280 VROCP0OXY2 + 0.720 NO2	2.70 × 10^−12^exp(360.00*/T*)	9.03 × 10^−12^
431	ROCALK12c	VROCP1ALKP + NO → 0.720 VROCP1ALKP2 + 0.280 VROCN1OXY1 + 0.720 NO2	2.70 × 10^−12^exp(360.00*/T*)	9.03 × 10^−12^
432	HC1002	HC10P + NO → 0.740 HC10P2 + 0.260 ONIT + 0.740 NO2	2.70 × 10^−12^exp(360.00*/T*)	9.03 × 10^−12^
433	ROCALK13c	VROCP6ALKP + NO3 → VROCP6ALKP2 + NO2	2.30 × 10^−12^	2.30 × 10^−12^
434	ROCALK14c	VROCP5ALKP + NO3 → VROCP5ALKP2 + NO2	2.30 × 10^−12^	2.30 × 10^−12^
435	ROCALK15c	VROCP4ALKP + NO3 → VROCP4ALKP2 + NO2	2.30 × 10^−12^	2.30 × 10^−12^
436	ROCALK16c	VROCP3ALKP + NO3 → VROCP3ALKP2 + NO2	2.30 × 10^−12^	2.30 × 10^−12^
437	ROCALK17c	VROCP2ALKP + NO3 → VROCP2ALKP2 + NO2	2.30 × 10^−12^	2.30 × 10^−12^
438	ROCALK18c	VROCP1ALKP + NO3 → VROCP1ALKP2 + NO2	2.30 × 10^−12^	2.30 × 10^−12^
439	HC1003	HC10P + NO3 → HC10P2 + NO2	2.30 × 10^−12^	2.30 × 10^−12^
440	ROCALK19c	VROCP6ALKP + HO2 → VROCP3OXY2	2.17 × 10^−11^	2.17 × 10^−11^
441	ROCALK20c	VROCP5ALKP + HO2 → VROCP2OXY2	2.20 × 10^−11^	2.20 × 10^−11^
442	ROCALK21c	VROCP4ALKP + HO2 → VROCP1OXY1	2.25 × 10^−11^	2.25 × 10^−11^
443	ROCALK22c	VROCP3ALKP + HO2 → VROCP0OXY2	2.26 × 10^−11^	2.26 × 10^−11^
444	ROCALK23c	VROCP2ALKP + HO2 → VROCN1OXY1	2.27 × 10^−11^	2.27 × 10^−11^
445	ROCALK24c	VROCP1ALKP + HO2 → VROCN2OXY2	2.27 × 10^−11^	2.27 × 10^−11^
446	HC1004	HC10P + HO2 → OP2	2.66 × 10^−13^exp(1300.00*/T*)	2.08 × 10^−11^
447	ROCALK25c	VROCP6ALKP2 → HO2 + VROCP3OXY2	1.88 × 10^−1^	1.88 × 10^−1^
448	ROCALK26c	VROCP5ALKP2 → HO2 + VROCP2OXY2	1.88 × 10^−1^	1.88 × 10^−1^
449	ROCALK27c	VROCP4ALKP2 → HO2 + VROCP1OXY1	1.88 × 10^−1^	1.88 × 10^−1^
450	ROCALK28c	VROCP3ALKP2 → HO2 + VROCP0OXY2	1.88 × 10^−1^	1.88 × 10^−1^
451	ROCALK29c	VROCP2ALKP2 → HO2 + VROCN1OXY1	1.88 × 10^−1^	1.88 × 10^−1^
452	ROCALK30c	VROCP1ALKP2 → HO2 + VROCN2OXY2	1.88 × 10^−1^	1.88 × 10^−1^
453	HC1005	HC10P2 → HO2 + VROCP4OXY2	1.88 × 10^−1^	1.88 × 10^−1^
454	ROCALK31c	VROCP6ALKP2 + NO → 0.140 VROCP2OXY2 + 0.860 NO2 + 0.860 VROCP3OXY2 + 0.860 HO2	2.70 × 10^−12^exp(360.00*/T*)	9.03 × 10^−12^
455	ROCALK32c	VROCP5ALKP2 + NO → 0.140 VROCP1OXY3 + 0.860 NO2 + 0.860 VROCP2OXY2 + 0.860 HO2	2.70 × 10^−12^exp(360.00*/T*)	9.03 × 10^−12^
456	ROCALK33c	VROCP4ALKP2 + NO → 0.140 VROCP0OXY2 + 0.860 NO2 + 0.860 VROCP1OXY1 + 0.860 HO2	2.70 × 10^−12^exp(360.00*/T*)	9.03 × 10^−12^
457	ROCALK34c	VROCP3ALKP2 + NO → 0.140 VROCN1OXY1 + 0.860 NO2 + 0.860 VROCP0OXY2 + 0.860 HO2	2.70 × 10^−12^exp(360.00*/T*)	9.03 × 10^−12^
458	ROCALK35c	VROCP2ALKP2 + NO → 0.140 VROCN2OXY2 + 0.860 NO2 + 0.860 VROCN1OXY1 + 0.860 HO2	2.70 × 10^−12^exp(360.00*/T*)	9.03 × 10^−12^
459	ROCALK36c	VROCP1ALKP2 + NO → VROCN2OXY2 + 0.860 NO2 + 0.860 HO2	2.70 × 10^−12^exp(360.00*/T*)	9.03 × 10^−12^
460	HC1006	HC10P2 + NO → 0.120 ONIT + 0.880 NO2 + 0.880 KET + 0.880 HO2	2.70 × 10^−12^exp(360.00*/T*)	9.03 × 10^−12^
461	ROCALK37c	VROCP6ALKP2 + NO3 → NO2 + VROCP3OXY2 + HO2	2.30 × 10^−12^	2.30 × 10^−12^
462	ROCALK38c	VROCP5ALKP2 + NO3 → NO2 + VROCP2OXY2 + HO2	2.30 × 10^−12^	2.30 × 10^−12^
463	ROCALK39c	VROCP4ALKP2 + NO3 → NO2 + VROCP1OXY1 + HO2	2.30 × 10^−12^	2.30 × 10^−12^
464	ROCALK40c	VROCP3ALKP2 + NO3 → NO2 + VROCP0OXY2 + HO2	2.30 × 10^−12^	2.30 × 10^−12^
465	ROCALK41c	VROCP2ALKP2 + NO3 → NO2 + VROCN1OXY1 + HO2	2.30 × 10^−12^	2.30 × 10^−12^
466	ROCALK42c	VROCP1ALKP2 + NO3 → NO2 + VROCN2OXY2 + HO2	2.30 × 10^−12^	2.30 × 10^−12^
467	HC1007	HC10P2 + NO3 → NO2 + KET + HO2	2.30 × 10^−12^	2.30 × 10^−12^
468	ROCALK43c	VROCP6ALKP2 + HO2 → VROCP1OXY3	2.17 × 10^−11^	2.17 × 10^−11^
469	ROCALK44c	VROCP5ALKP2 + HO2 → VROCP0OXY2	2.20 × 10^−11^	2.20 × 10^−11^
470	ROCALK45c	VROCP4ALKP2 + HO2 → VROCN1OXY1	2.25 × 10^−11^	2.25 × 10^−11^
471	ROCALK46c	VROCP3ALKP2 + HO2 → VROCN2OXY2	2.26 × 10^−11^	2.26 × 10^−11^
472	ROCALK47c	VROCP2ALKP2 + HO2 → VROCN2OXY2	2.27 × 10^−11^	2.27 × 10^−11^
473	ROCALK48c	VROCP1ALKP2 + HO2 → VROCN2OXY2	2.27 × 10^−11^	2.27 × 10^−11^
474	HC1008	HC10P2 + HO2 → VROCP2OXY2	2.66 × 10^−13^exp(1300.00*/T*)	2.08 × 10^−11^
475	ROCARO01	VROCP6ARO + HO → 0.840 VROCP6AROP + 0.160 HO2 + 0.160 VROCP4OXY2	1.81 × 10^−11^	1.81 × 10^−11^
476	ROCARO02	VROCP6AROP + HO2 → 0.059 VROCP4OXY2 + 0.905 VROCP1OXY3 + 0.036 VROCN2OXY4	2.91 × 10^−13^exp(1300.00*/T*)	2.28 × 10^−11^
477	ROCARO03	VROCP6AROP + NO → 1 × 10^−4^ VROCP4OXY2 + 0.002 VROCP2OXY2 + 1 × 10^−4^ VROCN1OXY3 + 0.998 NO2 + 0.998 HO2 + 0.059 BALD + 0.469 GLY + 0.469 MGLY + 0.469 FURANONE + 0.469 DCB2	2.70 × 10^−12^exp(360.00*/T*)	9.03 × 10^−12^
478	ROCARO04	VROCP6AROP + NO3 → NO2 + 0.941 HO2 + 0.059 BALD + 0.470 GLY + 0.470 MGLY + 0.470 FURANONE + 0.470 DCB2	2.30 × 10^−12^	2.30 × 10^−12^
479	ROCARO05	VROCP6AROP + MO2 → 0.680 HCHO + 1.310 HO2 + 0.320 MOH + 0.059 BALD + 0.470 GLY + 0.470 MGLY + 0.470 FURANONE + 0.470 DCB2	3.56 × 10^−14^exp(708.00*/T*)	3.83 × 10^−13^
480	ROCARO06	VROCP6AROP + ACO3 → 0.700 MO2 + 0.941 HO2 + 0.300 ORA2 + 0.059 BALD + 0.470 GLY + 0.470 MGLY + 0.470 FURANONE + 0.470 DCB2	7.40 × 10^−13^exp(765.00*/T*)	9.63 × 10^−12^
481	ROCARO11	VROCP5ARO + HO → 0.840 VROCP5AROP + 0.160 HO2 + 0.160 VROCP3OXY2	1.81 × 10^−11^	1.81 × 10^−11^
482	ROCARO12	VROCP5AROP + HO2 → 0.059 VROCP3OXY2 + 0.905 VROCP0OXY2 + 0.036 VROCN2OXY4	2.91 × 10^−13^exp(1300.00*/T*)	2.28 × 10^−11^
483	ROCARO13	VROCP5AROP + NO → 1 × 10^−4^ VROCP3OXY2 + 0.002 VROCP1OXY3 + 1 × 10^−4^ VROCN2OXY4 + 0.998 NO2 + 0.998 HO2 + 0.059 VROCP4OXY2 + 0.469 GLY + 0.469 MGLY + 0.469 FURANONE + 0.469 DCB2	2.70 × 10^−12^exp(360.00*/T*)	9.03 × 10^−12^
484	ROCARO14	VROCP5AROP + NO3 → NO2 + 0.941 HO2 + 0.059 VROCP4OXY2 + 0.470 GLY + 0.470 MGLY + 0.470 FURANONE + 0.470 DCB2	2.30 × 10^−12^	2.30 × 10^−12^
485	ROCARO15	VROCP5AROP + MO2 → 0.680 HCHO + 1.310 HO2 + 0.320 MOH + 0.059 VROCP4OXY2 + 0.470 GLY + 0.470 MGLY + 0.470 FURANONE + 0.470 DCB2	3.56 × 10^−14^exp(708.00*/T*)	3.83 × 10^−13^
486	ROCARO16	VROCP5AROP + ACO3 → 0.700 MO2 + 0.941 HO2 + 0.300 ORA2 + 0.059 VROCP4OXY2 + 0.470 GLY + 0.470 MGLY + 0.470 FURANONE + 0.470 DCB2	7.40 × 10^−13^exp(765.00*/T*)	9.63 × 10^−12^
487	ROCARO21	NAPH + HO → 0.840 NAPHP + 0.160 HO2 + 0.160 VROCP3OXY2	2.31 × 10^−11^	2.31 × 10^−11^
488	ROCARO22	NAPHP + HO2 → 0.059 VROCP3OXY2 + 0.905 VROCP1OXY3 + 0.036 VROCN2OXY8	2.91 × 10^−13^exp(1300.00*/T*)	2.28 × 10^−11^
489	ROCARO23	NAPHP + NO → 0.060 VROCP4OXY2 + 0.002 VROCP2OXY2 + 1 × 10^−4^ VROCN2OXY8 + 0.998 NO2 + 0.998 HO2 + 0.469 GLY + 0.469 MGLY + 0.469 FURANONE + 0.469 DCB2	2.70 × 10^−12^exp(360.00*/T*)	9.03 × 10^−12^
490	ROCARO24	NAPHP + NO3 → NO2 + 0.941 HO2 + 0.059 VROCP4OXY2 + 0.470 GLY + 0.470 MGLY + 0.470 FURANONE + 0.470 DCB2	2.30 × 10^−12^	2.30 × 10^−12^
491	ROCARO25	NAPHP + MO2 → 0.680 HCHO + 1.310 HO2 + 0.320 MOH + 0.059 VROCP4OXY2 + 0.470 GLY + 0.470 MGLY + 0.470 FURANONE + 0.470 DCB2	3.56 × 10^−14^exp(708.00*/T*)	3.83 × 10^−13^
492	ROCARO26	NAPHP + ACO3 → 0.700 MO2 + 0.941 HO2 + 0.300 ORA2 + 0.059 VROCP4OXY2 + 0.470 GLY + 0.470 MGLY + 0.470 FURANONE + 0.470 DCB2	7.40 × 10^−13^exp(765.00*/T*)	9.63 × 10^−12^
493	ROCOXY1c	VROCN2OXY8 + HO → HO + 0.085 VROCN2OXY8 + 0.258 DCB1 + 0.258 MEK + 0.258 ACD + 0.258 ALD + 0.258 MO2 + 0.258 ETHP + 0.258 HC3P + 0.258 MEKP	5.90 × 10^−11^	5.90 × 10^−11^
494	ROCOXY2c	VROCN2OXY4 + HO → HO + 0.464 VROCN2OXY8 + 0.198 VROCN2OXY4 + 0.012 VROCN1OXY6 + 0.015 VROCN1OXY3 + 0.062 VROCP0OXY4 + 0.039 VROCP1OXY3 + 0.049 VROCP2OXY2 + 0.040 VROCP3OXY2 + 0.018 VROCP4OXY2 + 0.031 OP3 + 0.004 OP2 + 0.079 DCB1 + 0.079 MEK + 0.079 KET + 0.079 ACD + 0.079 ALD + 0.079 MO2 + 0.079 ETHP + 0.079 HC3P + 0.079 MEKP + 0.079 HC5P + 0.079 KETP	6.07 × 10^−11^	6.07 × 10^−11^
495	ROCOXY3c	VROCN2OXY2 + HO → HO + 0.104 VROCN2OXY8 + 0.564 VROCN2OXY4 + 0.214 VROCN2OXY2 + 0.015 VROCN1OXY6 + 0.030 VROCN1OXY3 + 0.010 VROCN1OXY1 + 0.019 VROCP0OXY4 + 0.046 VROCP0OXY2 + 0.031 VROCP1OXY3 + 0.020 VROCP1OXY1 + 0.046 VROCP2OXY2 + 0.045 VROCP3OXY2 + 0.045 VROCP4OXY2 + 0.033 VROCP5OXY1 + 0.037 VROCP6OXY1 + 0.003 OP3 + 0.039 DCB1 + 0.039 HKET + 0.039 MEK + 0.039 ACD + 0.039 ALD + 0.039 MO2 + 0.039 ETHP + 0.039 HC3P + 0.039 MEKP + 0.092 HC5P	5.54 × 10^−11^	5.54 × 10^−11^
496	ROCOXY4c	VROCN1OXY6 + HO → HO + 0.204 VROCN2OXY8 + 0.007 VROCN2OXY4 + 0.184 DCB1 + 0.184 MEK + 0.184 KET + 0.184 ACD + 0.184 ALD + 0.184 MO2 + 0.184 ETHP + 0.184 HC3P + 0.184 MEKP + 0.184 HC5P	5.63 × 10^−11^	5.63 × 10^−11^
497	ROCOXY5c	VROCN1OXY3 + HO → HO + 0.279 VROCN2OXY8 + 0.403 VROCN2OXY4 + 0.009 VROCN2OXY2 + 0.032 VROCN1OXY6 + 0.008 VROCN1OXY3 + 0.019 VROCP0OXY4 + 0.010 VROCP0OXY2 + 0.051 VROCP1OXY3 + 0.007 VROCP1OXY1 + 0.051 VROCP2OXY2 + 0.046 VROCP3OXY2 + 0.051 VROCP4OXY2 + 0.014 VROCP5OXY1 + 0.013 OP2 + 0.065 DCB1 + 0.065 HKET + 0.065 MEK + 0.065 ACD + 0.065 ALD + 0.065 MO2 + 0.065 ETHP + 0.065 HC3P + 0.065 MEKP + 0.175 HC5P	5.46 × 10^−11^	5.46 × 10^−11^
498	ROCOXY6c	VROCN1OXY1 + HO → HO + 0.007 VROCN2OXY8 + 0.119 VROCN2OXY4 + 0.726 VROCN2OXY2 + 0.012 VROCN1OXY6 + 0.030 VROCN1OXY3 + 0.007 VROCN1OXY1 + 0.029 VROCP0OXY4 + 0.045 VROCP0OXY2 + 0.023 VROCP1OXY3 + 0.035 VROCP1OXY1 + 0.062 VROCP2OXY2 + 0.052 VROCP3OXY2 + 0.051 VROCP4OXY2 + 0.035 VROCP5OXY1 + 0.075 VROCP6OXY1 + 0.016 OP3 + 0.006 OP2 + 0.024 DCB1 + 0.024 HKET + 0.024 MEK + 0.024 ACD + 0.024 ALD + 0.024 MO2 + 0.024 ETHP + 0.024 HC3P + 0.024 MEKP + 0.054 HC5P	4.50 × 10^−11^	4.50 × 10^−11^
499	ROCOXY7c	VROCP0OXY4 + HO → HO + 0.282 VROCN2OXY8 + 0.117 VROCN2OXY4 + 0.032 VROCN1OXY6 + 0.018 VROCN1OXY3 + 0.001 VROCP0OXY4 + 0.066 VROCP2OXY2 + 0.053 VROCP3OXY2 + 0.025 VROCP4OXY2 + 0.005 OP2 + 0.107 DCB1 + 0.107 MEK + 0.107 KET + 0.107 ACD + 0.107 ALD + 0.107 MO2 + 0.107 ETHP + 0.107 HC3P + 0.107 MEKP + 0.107 HC5P + 0.107 KETP	5.17 × 10^−11^	5.17 × 10^−11^
500	ROCOXY8c	VROCP0OXY2 + HO → HO + 0.066 VROCN2OXY8 + 0.458 VROCN2OXY4 + 0.116 VROCN2OXY2 + 0.033 VROCN1OXY6 + 0.066 VROCN1OXY3 + 0.005 VROCN1OXY1 + 0.031 VROCP0OXY4 + 0.002 VROCP0OXY2 + 0.040 VROCP1OXY3 + 0.021 VROCP1OXY1 + 0.054 VROCP2OXY2 + 0.052 VROCP3OXY2 + 0.052 VROCP4OXY2 + 0.037 VROCP5OXY1 + 0.042 VROCP6OXY1 + 0.011 OP3 + 0.044 DCB1 + 0.044 HKET + 0.044 MEK + 0.044 ACD + 0.044 ALD + 0.044 MO2 + 0.044 ETHP + 0.044 HC3P + 0.044 MEKP + 0.105 HC5P	4.73 × 10^−11^	4.73 × 10^−11^
501	ROCOXY9c	VROCP1OXY3 + HO → HO + 0.178 VROCN2OXY8 + 0.192 VROCN2OXY4 + 4 × 10^−4^ VROCN2OXY2 + 0.074 VROCN1OXY6 + 0.045 VROCN1OXY3 + 0.063 VROCP0OXY4 + 0.001 VROCP0OXY2 + 0.001 VROCP1OXY3 + 0.023 VROCP2OXY2 + 0.059 VROCP3OXY2 + 0.065 VROCP4OXY2 + 0.017 VROCP5OXY1 + 0.015 OP3 + 0.017 OP2 + 0.082 DCB1 + 0.082 HKET + 0.082 MEK + 0.082 ACD + 0.082 ALD + 0.082 MO2 + 0.082 ETHP + 0.082 HC3P + 0.082 MEKP + 0.222 HC5P	4.60 × 10^−11^	4.60 × 10^−11^
502	ROCOXY10c	VROCP1OXY1 + HO → HO + 0.002 VROCN2OXY8 + 0.134 VROCN2OXY4 + 0.335 VROCN2OXY2 + 0.008 VROCN1OXY6 + 0.119 VROCN1OXY3 + 0.076 VROCN1OXY1 + 0.029 VROCP0OXY4 + 0.077 VROCP0OXY2 + 0.028 VROCP1OXY3 + 0.012 VROCP1OXY1 + 0.065 VROCP2OXY2 + 0.071 VROCP3OXY2 + 0.067 VROCP4OXY2 + 0.042 VROCP5OXY1 + 0.091 VROCP6OXY1 + 0.007 OP3 + 0.003 OP2 + 0.030 DCB1 + 0.030 HKET + 0.030 MEK + 0.030 ACD + 0.030 ALD + 0.030 MO2 + 0.030 ETHP + 0.030 HC3P + 0.030 MEKP + 0.065 HC5P	3.80 × 10^−11^	3.80 × 10^−11^
503	ROCOXY11c	VROCP2OXY2 + HO → HO + 0.044 VROCN2OXY8 + 0.173 VROCN2OXY4 + 0.010 VROCN2OXY2 + 0.051 VROCN1OXY6 + 0.112 VROCN1OXY3 + 0.001 VROCN1OXY1 + 0.134 VROCP0OXY4 + 0.040 VROCP0OXY2 + 0.051 VROCP1OXY3 + 0.007 VROCP1OXY1 + 0.024 VROCP2OXY2 + 0.029 VROCP3OXY2 + 0.073 VROCP4OXY2 + 0.052 VROCP5OXY1 + 0.059 VROCP6OXY1 + 0.004 OP3 + 0.002 OP2 + 0.063 DCB1 + 0.063 HKET + 0.063 MEK + 0.063 ACD + 0.063 ALD + 0.063 MO2 + 0.063 ETHP + 0.063 HC3P + 0.063 MEKP + 0.149 HC5P	3.93 × 10^−11^	3.93 × 10^−11^
504	ROCOXY12c	VROCP3OXY2 + HO → HO + 0.032 VROCN2OXY8 + 0.076 VROCN2OXY4 + 0.001 VROCN2OXY2 + 0.053 VROCN1OXY6 + 0.049 VROCN1OXY3 + 0.155 VROCP0OXY4 + 0.015 VROCP0OXY2 + 0.105 VROCP1OXY3 + 0.001 VROCP1OXY1 + 0.053 VROCP2OXY2 + 0.009 VROCP3OXY2 + 0.043 VROCP4OXY2 + 0.058 VROCP5OXY1 + 0.066 VROCP6OXY1 + 0.051 OP3 + 0.011 OP2 + 0.070 DCB1 + 0.070 HKET + 0.070 MEK + 0.070 ACD + 0.070 ALD + 0.070 MO2 + 0.070 ETHP + 0.070 HC3P + 0.070 MEKP + 0.166 HC5P	3.52 × 10^−11^	3.52 × 10^−11^
505	ROCOXY13c	VROCP4OXY2 + HO → HO + 0.012 VROCN2OXY8 + 0.017 VROCN2OXY4 + 0.048 VROCN1OXY6 + 0.025 VROCN1OXY3 + 0.088 VROCP0OXY4 + 0.092 VROCP1OXY3 + 0.007 VROCP1OXY1 + 0.097 VROCP2OXY2 + 0.046 VROCP3OXY2 + 0.002 VROCP4OXY2 + 0.048 VROCP5OXY1 + 0.074 VROCP6OXY1 + 0.061 OP3 + 0.015 OP2 + 0.079 DCB1 + 0.079 HKET + 0.079 MEK + 0.079 ACD + 0.079 ALD + 0.079 MO2 + 0.079 ETHP + 0.079 HC3P + 0.079 MEKP + 0.173 HC5P	3.12 × 10^−11^	3.12 × 10^−11^
506	ROCOXY14c	VROCP5OXY1 + HO → HO + 0.010 VROCN2OXY4 + 0.001 VROCN2OXY2 + 0.009 VROCN1OXY6 + 0.015 VROCN1OXY3 + 0.070 VROCP0OXY4 + 0.015 VROCP0OXY2 + 0.104 VROCP1OXY3 + 0.003 VROCP1OXY1 + 0.165 VROCP2OXY2 + 0.157 VROCP3OXY2 + 0.072 VROCP4OXY2 + 0.006 VROCP5OXY1 + 0.140 VROCP6OXY1 + 0.022 OP3 + 0.038 OP2 + 0.053 DCB1 + 0.053 HKET + 0.053 MEK + 0.053 ACD + 0.053 ALD + 0.053 MO2 + 0.053 ETHP + 0.053 HC3P + 0.053 MEKP + 0.128 HC5P	2.40 × 10^−11^	2.40 × 10^−11^
507	ROCOXY15c	VROCP6OXY1 + HO → HO + 0.006 VROCN1OXY6 + 0.005 VROCN1OXY3 + 0.022 VROCP0OXY4 + 0.050 VROCP1OXY3 + 0.002 VROCP1OXY1 + 0.088 VROCP2OXY2 + 0.138 VROCP3OXY2 + 0.146 VROCP4OXY2 + 0.043 VROCP5OXY1 + 0.096 VROCP6OXY1 + 0.032 OP3 + 0.059 OP2 + 0.057 DCB1 + 0.057 HKET + 0.057 MEK + 0.057 ACD + 0.057 ALD + 0.057 MO2 + 0.057 ETHP + 0.057 HC3P + 0.057 MEKP + 0.154 HC5P	2.05 × 10^−11^	2.05 × 10^−11^
508	ROCOXY16c	OP3 + HO → HO + 0.119 VROCN2OXY8 + 0.001 VROCN2OXY4 + 0.039 VROCN1OXY6 + 0.011 VROCP0OXY4 + 0.227 DCB1 + 0.227 MEK + 0.227 ACD + 0.227 ALD + 0.227 MO2 + 0.227 ETHP + 0.227 HC3P + 0.227 MEKP	4.69 × 10^−11^	4.69 × 10^−11^

a Reaction rate constants following Arrhenius behavior are specified as $k={\text{Ae}}^{-\text{Ea}/\text{RT}}$. Falloff or pressure-dependent reaction rate constants are specified as follows (M equals air number density): for rate constants with $k_{o},k_{i},n$, and F values, $k=\left[k_{o}M/\left(1+k_{o}M/k_{i}\right)\right]F^{G}$, where $G={\left(1+{\left({\text{log}}_{10}\left(k_{o}M/k_{i}\right)/n\right)}^{2}\right)}^{-1}$; for rate constants with $k_{1}$ and $k_{2},k=k_{1}+k_{2}M$; for rate constants with $k_{0},k_{2}$, and $k_{3},k=k_{0}+k_{3}M/\left(1+k_{3}M/k_{2}\right)$; and for rate constants with $k_{1},k_{2}$, and $k_{3},k=k_{1}+k_{2}M+k_{3}$.

b Heterogeneous rates are specified as $k_{\text{HET}}=\frac{S_{\text{A}}}{r_{p}/D_{g}+4/\nu \gamma }$, where $S_{\text{A}}$ is the fine aerosol surface area, $r_{\text{p}}$ is the effective particle radius, $D_{\text{g}}$ is the gas-phase diffusivity, v is the mean molecular speed, and γ is the uptake coefficient. In the case of a heterogeneous NO_2_ reaction, the gas-phase diffusivity term in the denominator is neglected.

c CMAQ calculates photolysis rate coefficients (J values) as follows: $J_{i}=\int_{\lambda 1}^{\lambda 2}\;F(\lambda )\sigma_{i}(\lambda )\phi_{i}(\lambda )\text{d}\lambda$, where F(λ) is the actinic flux (photons ${\text{cm}}^{-2}\;{\text{min}}^{-1}\;{\text{nm}}^{-1}$), $\sigma_{i}(\lambda )$ is the absorption cross-section for the molecule undergoing a photolytic reaction (${\text{cm}}^{2}\;{\text{molec}.}^{-1}$), (photons $\left.{\text{cm}}^{-2}\;{\text{min}}^{-1}\;\;{\text{nm}}^{-1}\right),\sigma_{i}(\lambda )$ is the absorption cross-section for the molecule undergoing a photolytic reaction $\left({\text{cm}}^{2}\;{\text{molec}.}^{-1}\right.$), $\varphi_{i}(\lambda )$ is the quantum yield of the photolysis reaction (${\text{molec}.\;\text{photon}}^{-1}$), and λ is the wavelength (nm). CMAQ uses seven-binned absorption cross-section and quantum yield data for calculating J values. Sources of absorption cross-section and quantum yield data are provided in the table.

d The rate constant for R067 is scaled to the reverse equilibrium of R066.

e The HAL_Ozone reaction represents a loss of ozone over ocean surfaces due to halogen chemistry. The rate is set to 0 if the sun is below the horizon and if the surface does not include sea or surf zones (P : air pressure in atmospheres) (Sarwar et al., 2015).

f SULF represents sulfuric acid. In CMAQ, a tracking species, SULRXN, is used to implement sulfuric acid and subsequent condensation.

g IEPOXP is an intermediate used for logistical reasons in CMAQ. It does not have a meaningful concentration.
