# Supplementary material for: Linking gas, particulate, and toxic endpoints to air emissions in the Community Regional Atmospheric Chemistry Multiphase Mechanism (CRACMM)
Source: Atmos Chem Phys. Author manuscript; Available in PMC 2025 Jan 27. (PMC11770585; doi:10.5194/acp-23-5043-2023)
Supplement: Supplement1 [file NIHMS2038939-supplement-Supplement1.pdf]

Supplement of Atmos. Chem. Phys., 23, 5043–5099, 2023
https://doi.org/10.5194/acp-23-5043-2023-supplement
© Author(s) 2023. CC BY 4.0 License.

Atmospheric Chemistry and Physics
Open Access

Supplement of

Linking gas, particulate, and toxic endpoints to air emissions in the Community Regional Atmospheric Chemistry Multiphase Mechanism (CRACMM)

Havala O. T. Pye et al.

Correspondence to: Havala O. T. Pye (pye.havala@epa.gov)

The copyright of individual parts of the supplement might differ from the article licence.

Derivation of aromatic system SOA yields

SOA from phenolic species (PHEN and CSL) were set to reproduce SOA yields under RO_2+NO dominant conditions for benzene (BEN), toluene (TOL), and m-xylenes (XYM) assuming all SOA under RO_2+NO dominant conditions comes from the phenolic route. The yield of phenolic compounds from BEN, TOL, and XYM is insensitive to NO_X. Mass-based yields from Ng et al. (2007) were wall loss corrected using information from Zhang et al. (2014). Using the OM/OC ratio of the SOA (Pye et al., 2017) and assuming the carbon backbone was retained, a molar SOA yield from BEN, TOL, and XYM, consistent with laboratory data was calculated following:

$$\text{Molar Yield} = \text{Mass Yield} * \text{Wall Loss Correction} / \text{MWT SOA} * \text{MWT Parent} \quad \text{(S1)}$$

where MWT is the molecular weight (Table S3). Using the molar yield of PHEN and CSL from BEN, TOLU, and XYM, the molar yields of SOA from PHEN and CSL was calculated following (Table S4):

$$\text{SOA from Phenolic} = \text{SOA from Parent} / \text{Molar Phenolic Yield} \quad \text{(S2)}$$

This resulted in an estimated PHEN SOA yield of 0.15 by mole and from CSL of 0.20 by mole (average of toluene and xylene inferred yields). XYE was treated the same as XYM.

The fraction of products undergoing autoxidation, α_A, in BEN, TOL, XYE, and XYM systems was set to reproduce observed SOA yields when combined with the phenolic route under RO_2+HO_2 dominant. Mass-based yields from Ng et al. (2007) were wall loss corrected using information from Zhang et al. (2014) following equation (1) (Table S5). The autoxidation fraction (Table S6) was calculated following:

$$\alpha_A = (RO_2+HO_2 \text{ SOA Yield by mole}) – (RO_2+NO \text{ SOA Yield by mole}) \quad \text{(S3)}$$

For IVOC aromatics (NAPH, ROCP5ARO, ROCP6ARO), the autoxidation fraction is set to 3% following the work of Molteni et al. (2018).

Table S1: Species-level mapping of SPECIATE to CRACMM. Species from SPECIATE (ID and Species) with nonzero emissions are mapped to CRACMM using the Representative Compound Structures. This information is also available in the data archive along with 2017 ROC anthropogenic and biomass burning emission rates as Table D2.

ID	Species	Representative Compound	CRACMM
1	(1-methylpropyl)benzene (or sec-butylbenzene)	sec-Butylbenzene	XYE
3	(2-methylpropyl)benzene (or isobutylbenzene)	Isobutylbenzene	XYE
4	1,1,1-trichloroethane	1,1,1-Trichloroethane	SLOWROC
7	1,1,2-trichloroethane	1,1,2-Trichloroethane	SLOWROC
9	1,1,2-trimethylcyclopentane	1,1,2-Trimethylcyclopentane	HC10
12	1,1,3-trimethylcyclohexane	1,1,3-Trimethylcyclohexane	HC10
13	1,1,3-trimethylcyclopentane	1,1,3-Trimethylcyclopentane	HC10
14	1,1,4-trimethylcyclohexane	1,1,4-Trimethylcyclohexane	HC10
15	1,1-dichloro-1-fluoroethane	1,1-Dichloro-1-fluoroethane	SLOWROC
16	1,1-dichloroethane (or Ethylidene dichloride)	1,1-Dichloroethane	SLOWROC
17	1,1-dichloroethene (or Vinylidene chloride)	1,1-Dichloroethylene	OLT
19	1,1-dimethylcyclohexane	1,1-Dimethylcyclohexane	HC10
20	1,1-dimethylcyclopentane	1,1-Dimethylcyclopentane	HC10
21	1,1-Methylethylcyclopentane	Cyclopentane, 1-ethyl-1- methyl-	HC10
22	1,2,3,4-tetramethylbenzene	1,2,3,4-Tetramethylbenzene	ROCP6ARO
23	1,2,3,5-tetramethylbenzene	1,2,3,5-Tetramethylbenzene	XYM
25	1,2,3-trimethylbenzene	1,2,3-Trimethylbenzene	XYM
26	1,2,3-trimethylcyclohexane	1,2,3-Trimethylcyclohexane	HC10
27	1,2,3-trimethylcyclopentane	1,2,3-Trimethylcyclopentane	HC10
28	1,2,4,5-tetramethylbenzene	1,2,4,5-Tetramethylbenzene	XYM
29	1,2,4-triethylbenzene	1,2,4-Triethylbenzene	ROCP6ARO
30	1,2,4-trimethylbenzene	1,2,4-Trimethylbenzene	XYM
31	1,2,4-trimethylcyclopentane	1,2,4-Trimethylcyclopentane	HC10
32	1,2,4-trimethylcyclopentene	Cyclopentene, 1,2-dimethyl-4-methylene-	FURAN
33	1,2-butadiene	1,2-Butadiene	FURAN
34	1,2-dichloropropane (or Propylene dichloride)	1,2-Dichloropropane	HC3
36	1,2-diethylbenzene (or o-diethylbenzene)	o-Diethylbenzene	XYE
37	1,2-dimethyl-3-ethylbenzene	3-Ethyl-o-xylene	XYE
39	1,2-dimethyl-4-ethylbenzene (or 2-Methyl-p-ethyltoluene || 4-Ethyl-o-xylene || 4-Ethyl-1,2-dimethylbenzene || 3,4-Dimethyl-1-ethylbenzene)	1,2-Dimethyl-4-ethylbenzene	XYE
40	1,2-dimethylcyclopentane	1,2-Dimethylcyclopentane	HC10
42	1,2-propadiene	1,2-Propadiene	FURAN
43	1,3,5-triethylbenzene	1,3,5-Triethylbenzene	ROCP6ARO
44	1,3,5-trimethylbenzene	1,3,5-Trimethylbenzene	XYM
45	1,3,5-trimethylcyclohexane	1,3,5-Trimethylcyclohexane	HC10
46	1,3-butadiene	1,3-Butadiene	BDE13
47	1,3-butadiyne	1,3-Butadiyne	HC10
48	1,3-cyclopentadiene	1,3-Cyclopentadiene	FURAN
49	1,3-dichlorobenzene (or m-dichlorobenzene)	1,3-Dichlorobenzene	XYE
51	1,3-diethylbenzene (or m-diethylbenzene)	1,3-Diethylbenzene	XYE
52	1,3-dimethyl-2-ethylbenzene	2-Ethyl-m-xylene	XYE
53	1,3-dimethyl-4-ethylbenzene (or 4-Ethyl-m-xylene || 2,4-Dimethyl-1-ethylbenzene || 4-Ethyl-1,3-dimethylbenzene)	1,3-Dimethyl-4-ethylbenzene	XYE
54	1,3-dimethyl-4-isopropylbenzene	Benzene, 2,4-dimethyl-1-(1-methylethyl)-	XYM
55	1,3-dimethyl-5-ethylbenzene	5-Ethyl-m-xylene	XYE
57	1,3-dipropylbenzene	Benzene, 1,4-dipropyl-	ROCP6ARO
59	1,4-diethylbenzene (or p-diethylbenzene)	1,4-Diethylbenzene	XYE
60	1,4-dimethyl-2-ethylbenzene	2-Ethyl-p-xylene	XYE
61	1,4-dioxane (or p-Dioxane || 1,4-Diethyleneoxide)	1,4-Dioxane	HC10
63	1-(1,1-dimethylethyl)-3,5-dimethylbenzene (or tert-butyl-3,5-dimethylbenzene)	5-Tert-butyl-m-xylene	XYM
64	1-butene	1-Butene	OLT
65	1-butyne (or Ethylacetylene; Ethylethyne)	1-Butyne	HC10
71	1-ethyl-2-npropylbenzene	1-ethyl-2-propylbenzene	XYM
75	1-ethyltertbutylether	Ethyl T-butyl ether	HC10
76	1-heptene	1-Heptene	OLT
77	1-hexanol	1-Hexanol	ROH
78	1-hexene	1-Hexene	OLT
80	1-Methyl-2-ethylbenzene (or o-ethyltoluene || 1-Ethyl-2-methylbenzene || 2-ethyltoluene || 2-Ethylmethylbenzene)	1-Ethyl-2-methylbenzene	XYE

ID	Species	Representative Compound	CRACMM
81	1-Methyl-2-isopropylbenzene (or o-cymene || Ortho-Isopropyltoluene)	1-Isopropyl-2-methylbenzene	XYE
83	1-Methyl-2-n-butylbenzene	Benzene, 1-butyl-2-methyl-	XYM
84	1-Methyl-2-n-propylbenzene (or 2-propyltoluene)	2-Propyltoluene	XYE
85	1-Methyl-2-pyrrolidinone	N-Methyl-2-pyrrolidone	ROCIOXY
86	1-Methyl-2-tert-butylbenzene	Benzene, 1-(1,1-dimethylethyl)-2-methyl-	XYE
88	1-Methyl-3-butylbenzene	Benzene, 1-butyl-3-methyl-	XYM
89	1-Methyl-3-ethylbenzene (or 1-Ethyl-3-methylbenzene || 3-Ethyltoluene)	3-Ethyltoluene	XYM
90	1-Methyl-3-isopropylbenzene (or 1-Methyl-3-(1-methylethyl)-benzene || 3-isopropyltoluene || m-cymene)	m-Cymene	XYE
92	1-Methyl-3-propylbenzene (or 3-n-propyltoluene)	1-Methyl-3-propylbenzene	XYE
94	1-Methyl-4-ethylbenzene (or 1-Ethyl-4-methylbenzene || 4-ethyltoluene)	4-Ethyltoluene	XYE
95	1-Methyl-4-ethylcyclohexane	trans-1-ethyl-4-methyl-Cyclohexane	HC10
96	1-Methyl-4-isobutylbenzene	1-(Butan-2-yl)-4-methylbenzene	XYM
97	1-Methyl-4-isopropylbenzene (or p-Cymene || p-Isopropyltoluene || p-Methylisopropylbenzene || Camphogen || Dolcymene || 1-Isopropyl-4-methylbenzene)	p-Cymene	ROCP6ARO
98	1-Methyl-4-isopropylcyclohexane	1-Isopropyl-4-methylcyclohexane	HC10
100	1-Methyl-4-n-propylbenzene	p-Propyltoluene	XYE
103	1-Methylcyclopentene	1-Methylcyclopent-1-ene	OLI
104	1-Methylindan (or 1-Methylindane)	1-Methyl-2,3-dihydro-1H-indene	ROCP6ARO
105	1-Methylnaphthalene	1-Methylnaphthalene	NAPH
106	1-nonene	1-Nonene	OLT
107	1-octene	1-Octene	OLT
108	1-pentene	1-Pentene	OLT
109	1-propyne	1-Propyne	HC5
112	2,2,3-trimethylbutane	2,2,3-Trimethylbutane	HC5
113	2,2,3-trimethylpentane	2,2,3-Trimethylpentane	HC5
114	2,2,4,6,6-pentamethylheptane	2,2,4,6,6-Pentamethylheptane	HC10
115	2,2,4-trimethyl-1,3-pentanediol isobutyrate	3-Hydroxy-2,2,4-trimethylpentyl 2-methylpropanoate	ROCIOXY
116	2,2,4-trimethylheptane	2,2,4-Trimethylheptane	HC10
117	2,2,4-trimethylhexane	2,2,4-Trimethylhexane	HC10
118	2,2,4-trimethylpentane	2,2,4-Trimethylpentane	HC5
120	2,2,5-trimethylheptane	2,2,4-Trimethylheptane	HC10
121	2,2,5-trimethylhexane	Hexane, 2,2,5-trimethyl-	HC10
122	2,2-dimethylbutane	2,2-Dimethylbutane	HC3
124	2,2-dimethylhexane	Hexane, 2,2-dimethyl-	HC5
125	2,2-dimethyloctane	Octane, 2,2-dimethyl-	HC10
126	2,2-dimethylpentane	Pentane, 2,2-dimethyl-	HC3
127	2,2-dimethylpropane (or Neopentane || 1,1,1-Trimethylethane || Dimethylpropane)	Propane, 2,2-dimethyl-	HC3
128	2,3,3-trimethylpentane	Pentane, 2,3,3-trimethyl-	HC5
129	2,3,4-trimethylhexane	hexane, 2,3,4-trimethyl-	HC10
130	2,3,4-trimethylpentane	2,3,4-Trimethylpentane	HC10
132	2,3,5-trimethylhexane	2,3,5-Trimethylhexane	HC10
133	2,3-dimethyl-1-butene	1-Butene, 2,3-dimethyl-	OLT
135	2,3-dimethyl-2-pentene	2-Pentene, 2,3-dimethyl-	OLI
136	2,3-dimethylbutane	2,3-Dimethylbutane	HC5
137	2,3-dimethylheptane	2,3-Dimethylheptane	HC10
138	2,3-dimethylhexane	2,3-Dimethylhexane	HC5
139	2,3-dimethyloctane	2,3-Dimethyloctane	HC10
140	2,3-dimethylpentane	Pentane, 2,3-dimethyl-	HC5
141	2,4,4-trimethyl-1-pentene	2,4,4-Trimethyl-1-pentene	OLI
142	2,4,4-trimethyl-2-pentene	2,4,4-Trimethyl-2-pentene	OLI
143	2,4,4-trimethylhexane	2,4,4-Trimethylhexane	HC10
145	2,4,5-trimethylheptane	2,4,6-Trimethylheptane	HC10
146	2,4-dimethyl-1-pentene	2,4-Dimethylpent-1-ene	OLT
147	2,4-dimethyl-2-pentene	2,4-Dimethylpent-2-ene	OLI
148	2,4-dimethylheptane	2,4-Dimethylheptane	HC10
149	2,4-dimethylhexane	2,4-Dimethylhexane	HC5
151	2,4-dimethyloctane	2,5-Dimethyloctane	HC10
152	2,4-dimethylpentane	2,4-Dimethylpentane	HC5
154	2,4-toluene diisocyanate	Toluene 2,4-diisocyanate	ROCP5ARO
155	2,5-dimethylheptane	2,5-Dimethylheptane	HC10

ID	Species	Representative Compound	CRACMM
156	2,5-dimethylhexane	2,5-Dimethylhexane	HC5
158	2,5-dimethyloctane	2,5-Dimethyloctane	HC10
159	2,6-dimethyldecane	2,6-Dimethyldecane	HC10
160	2,6-dimethylheptane	2,6-Dimethylheptane	HC10
161	2,6-dimethylnonane	2,6-Dimethylnonane	HC10
162	2,6-dimethyloctane	2,6-Dimethyloctane	HC10
167	2-(2-butoxyethoxy)ethanol	2-(2-Butoxyethoxy)ethanol	ROCIOXY
168	2-(2-ethylhexyloxy)ethanol	2-(2-Ethylhexyloxy)ethanol	ROH
169	2-amino-2-methyl-1-propanol	2-Amino-2-methylpropan-1-ol	ROH
170	2-butyne	2-Butyne	HC10
172	2-ethoxyethanol (or cellosolve || EGEE)	2-Ethoxyethanol	ROH
173	2-ethoxyethyl acetate (or cellosolve acetate)	Ethylene glycol monoethyl ether acetate	HC10
177	2-hexene	2-â€‹Hexene	OLI
180	2-methoxyethanol (or methyl cellosolve || EGME)	2-Methoxyethanol	ROH
181	2-methyl-1-butene	2-Methylbut-1-ene	OLT
182	2-methyl-1-butyl acetate	2-Methylbutyl acetate	HC10
184	2-methyl-1-pentene	2-Methylpent-1-ene	OLT
185	2-methyl-2-butene	2-Methyl-2-butene	OLI
186	2-methyl-2-hexene	2-Hexene, 2-methyl-	OLI
187	2-methyl-2-pentene	2-Methyl-2-pentene	OLI
188	2-methyl-2-propenal (or Methacrolein || Methacrylaldehyde; Isobutenal || Methacrylic aldehyde)	Methacrylaldehyde	MACR
189	2-methyl-3-ethylpentane	3-Ethyl-2-methylpentane	HC5
190	2-methyl-trans-3-hexene	3-HEXENE, 2-METHYL-, (E)-	OLI
192	2-methyldecane	2-Methyldecane	HC10
193	2-methylheptane	2-Methylheptane	HC10
194	2-methylhexane	2-Methylhexane	HC5
195	2-methylindan	1H-Indene, 2,3-dihydro-2-methyl-	ROCP6ARO
196	2-methylnaphthalene	2-Methylnaphthalene	NAPH
197	2-methylnonane	2-Methylnonane	HC10
198	2-methyloctane	2-Methyloctane	HC10
199	2-methylpentane (or isohexane)	2-Methylpentane	HC5
203	3,3-dimethyl-1-butene (or 3,3-Dimethylbutene)	3,3-Dimethyl-1-butene	OLT
205	3,3-dimethylheptane	Heptane, 3,3-dimethyl-	HC10
206	3,3-dimethylhexane	3,3-Dimethylhexane	HC5
207	3,3-dimethyloctane	3,3-Dimethyloctane	HC10
208	3,3-dimethylpentane	3,3-Dimethylpentane	HC5
209	3,4-dimethyl-1-pentene	3,4-Dimethylpent-1-ene	OLT
211	3,4-dimethylheptane	3,4-Dimethylheptane	HC10
212	3,4-dimethylhexane	3,4-Dimethylhexane	HC5
215	3,5-dimethylheptane	Heptane, 3,5-dimethyl-	HC10
217	3,5-dimethyloctane	3,5-Dimethyloctane	HC10
218	3,6-dimethyloctane	Octane, 3,6-dimethyl-	HC10
220	3-ethyl-2-methylheptane	3-Ethyl-2-methylheptane	HC10
221	3-ethyl-2-pentene	2-Pentene, 3-ethyl-	OLI
225	3-ethylheptane	3-Ethylheptane	HC10
226	3-ethylhexane	3-Ethylhexane	HC10
228	3-ethyloctane	Octane, 3-ethyl-	HC10
229	3-ethylpentane	Pentane, 3-ethyl-	HC5
230	3-methyl-1-butene	3-Methyl-1-butene	OLT
231	3-methyl-1-hexene	1-Hexene, 3-methyl-	OLT
232	3-methyl-1-pentene	3-Methylpent-1-ene	OLT
233	3-methyl-3-ethylpentane	Pentane, 3-ethyl-3-methyl-	HC5
235	3-methyl-cis-2-hexene	2-HEXENE, 3-METHYL-, (Z)-	OLI
236	3-methyl-cis-2-pentene (or cis-3-Methyl-2-pentene)	2-Pentene, 3-methyl-, (2Z)-	OLI
237	3-methyl-cis-3-hexene	3-hexene, 3-methyl-, (z)-	OLI
239	3-methyl-trans-2-pentene (or trans-3-Methyl-2-Pentene)	(E)-3-Methylpent-2-ene	OLI
240	3-methyl-trans-3-hexene	(E)-3-Methyl-3-hexene	OLI
242	3-methylcyclopentene	3-Methylcyclopent-1-ene	OLI
243	3-methyldecane	3-Methyldecane	HC10
244	3-methylheptane	Heptane, 3-methyl-	HC10
245	3-methylhexane	3-Methylhexane	HC5
246	3-methylnonane	3-Methylnonane	HC10
247	3-methyloctane	3-Methyloctane	HC10
248	3-methylpentane	3-Methylpentane	HC5

ID	Species	Representative Compound	CRACMM
253	4,4-dimethylheptane	Heptane, 4,4-dimethyl-	HC10
255	4,5-dimethyloctane	2,3-Dimethyloctane	HC10
257	4-methyl-1-hexene	4-Methylhex-1-ene	OLT
258	4-methyl-1-pentene	4-Methyl-1-pentene	OLT
260	4-methyl-cis-2-pentene (or cis-4-Methyl-2-Pentene)	2-pentene, 4-methyl-, (z)-	OLI
261	4-methyl-trans-2-hexene	2-HEXENE, 4-METHYL-, (E)-	OLI
262	4-methyl-trans-2-pentene	(2E)-4-methylpent-2-ene	OLI
263	4-methyldecane	4-Methyldecane	HC10
264	4-methylheptane	4-Methylheptane	HC10
265	4-methylindan	4-Methylindan	ROCP6ARO
266	4-methylnonane	4-Methylnonane	HC10
267	4-methyloctane	4-Methyloctane	HC10
268	4-methylundecane	4-Methylundecane	HC10
271	5-methyl-cis-2-hexene	2-Hexene, 5-methyl-	OLI
272	5-methyldecane	5-Methyldecane	HC10
273	5-methylindan	5-Methylindan	ROCP6ARO
279	Acetaldehyde	Acetaldehyde	ACD
280	Acetic acid	Acetic acid	ORA2
281	Acetone	Acetone	ACT
282	Acetylene (or ethyne)	Ethyne	ACE
283	Acrolein (or 2-propenal)	Acrolein	ACRO
285	Acrylonitrile	Acrylonitrile	OLT
287	Aggregated VOCs	Decane	HC10
290	Aliphatics	Decane	HC10
291	Alkene ketone	Methyl vinyl ketone	MVK
295	Amyl acetate	Pentyl acetate	HC10
299	Î²-methylstyrene (or Î²-Methylstyrol || Ï‰-Methylstyrene || Isoallylbenzene || Propenylbenzene || 1-Phenyl-1-propene || 1-Phenylpropene || 1-Propene, 1-phenyl-|| 1-Propenylbenzene)	Propenylbenzene	XYM
301	Benzaldehyde	Benzaldehyde	BALD
302	Benzene	Benzene	BEN
306	Benzyl alcohol	Benzyl alcohol	CSL
308	Bromodichloromethane	Bromodichloromethane	SLOWROC
310	Butyl cellosolve (or 2-butoxyethanol || EGBE || ethylene glycol monobutyl ether)	2-Butoxyethanol	ROH
311	Butylbenzylphthalate	Benzyl butyl phthalate	ROCP2ALK
312	Butylcyclohexane	Butylcyclohexane	HC10
313	Butyraldehyde (or butanal)	Butyraldehyde	ALD
315	C10 aromatics	Naphthalene	NAPH
316	C10 internal alkenes	(2E)-2-Decene	OLI
318	C11 dialkyl benzenes	1-ethyl-2-propylbenzene	XYM
320	C12 dialkyl benzenes	1,2,4-Triethylbenzene	ROCP6ARO
323	C5 aldehyde	Pentanal	ALD
324	C6 aldehydes	Hexanal	ALD
326	C9 aromatics	1,2,3-Trimethylbenzene	XYM
327	C9-c12 isoalkanes	2-Methyldecane	HC10
330	Camphor	Camphor	KET
331	Carbitol (or DEGEE || 2-(2-ethoxyethoxy)ethanol)	2-(2-Ethoxyethoxy)ethanol	ROCIOXY
332	Carbon disulfide	Carbon disulfide	SLOWROC
333	Carbon tetrachloride	Carbon tetrachloride	SLOWROC
335	Carbonyl sulfide	Carbonyl sulfide	SLOWROC
340	Chlorobenzene	Chlorobenzene	XYE
341	Chlorodifluoromethane	Chlorodifluoromethane	SLOWROC
342	Chlorofluorohydrocarbons	1,1,1-Trichloro-2,2,2-trifluoroethane	SLOWROC
343	Chloroform (or Trichlormethan; Methane trichloride)	Chloroform	SLOWROC
344	Chloropicrin	Chloropicrin	SLOWROC
346	Chlorpyrifos	Chlorpyrifos	ROCP5ARO
350	Cis, cis, trans-1,2,4-trimethylcyclohexane	1,2,4-Trimethylcyclohexane	HC10
351	Cis-1,2-dimethylcyclohexane	(Z)-1,2-Dimethylcyclohexane	HC10
352	Cis-1,3-dimethylcyclohexane	(Z)-1,3-Dimethylcyclohexane	HC10
353	Cis-1,3-dimethylcyclopentane	Cyclopentane, 1,3-dimethyl-, (1R,3S)-rel-	HC10
354	Cis-1,4-dimethylcyclohexane	1,4-Dimethylcyclohexane	HC10
356	Cis-1,cis-3,5-trimethylcyclohexane	1,3,5-Trimethylcyclohexane	HC10
357	Cis-1,trans-2,3-trimethylcyclopentane	rel-(1R,3R)-1,2,3-Trimethylcyclopentane	HC10

ID	Species	Representative Compound	CRACMM
358	Cis-1,trans-2,4-trimethylcyclopentane (or 1-trans-2,cis-4-Trimethylcyclopentane; 1-trans-2-trans-4-Trimethylcyclopentane)	1,2,4-Trimethylcyclopentane	HC10
359	Cis-1,trans-2,trans-4-trimethylcyclohexane	1,2,4-Trimethylcyclohexane	HC10
361	Cis-1-ethyl-2-methylcyclohexane	CYCLOHEXANE, 1-ETHYL-2-METHYL-, CIS-	HC10
362	Cis-1-ethyl-2-methylcyclopentane	(Z)-1-Ethyl-2-methylcyclopentane	HC10
363	Cis-1-ethyl-3-methylcyclohexane	(Z)-1-Ethyl-3-methylcyclohexane	HC10
364	Cis-1-ethyl-3-methylcyclopentane	CYCLOPENTANE, 1-ETHYL-3-METHYL-, CIS-	HC10
367	Cis-2-butene	2-Butene, (2Z)-	OLI
368	Cis-2-heptene	(2Z)-Heptene	OLI
369	Cis-2-hexene	(2Z)-2-Hexene	OLI
370	Cis-2-octene	(Z)-Oct-2-ene	OLI
371	Cis-2-pentene	(2Z)-2-Pentene	OLI
372	Cis-3-hexene	(3Z)-3-Hexene	OLI
373	Cis-3-nonene	(3Z)-non-3-ene	OLI
377	Citrus lemon peel oil	D-Limonene	LIM
381	Cresylic acid (mixed cresols)	o-Cresol	CSL
382	Crotonaldehyde (or 2-Butenal)	Crotonaldehyde	MACR
383	Cumene hydroperoxide	Cumene hydroperoxide	ROCP5ARO
385	Cyclohexane	Cyclohexane	HC10
386	Cyclohexanol	Cyclohexanol	ROH
387	Cyclohexanone	Cyclohexanone	OLI
388	Cyclohexene	Cyclohexene	OLI
390	Cyclopentane	Cyclopentane	HC5
391	Cyclopentene	Cyclopentene	OLI
392	D-limonene (or 4-isopropenyl-1-methylcycohexane || 1-methyl-4-(prop-1-en-2-yl)cyclohexene)	D-Limonene	LIM
394	Di(2-ethylhexyl)phthalate (or Bis(2-ethylhexyl) phthalate)	Di(2-ethylhexyl) phthalate	ROCP0ALK
395	Di(propylene glycol) methyl ether	1-(2-Methoxypropoxy)-2-propanol	ROCIOXY
396	Diacetone	Diacetone alcohol	HKET
398	Dibutyl phthalate	Dibutyl phthalate	ROCP2ALK
399	Dichlorobenzene (mixed isomers)	1,2-Dichlorobenzene	XYE
400	Dichlorodifluoromethane	Dichlorodifluoromethane	SLOWROC
401	Dichloromethane (or methylene chloride)	Dichloromethane	SLOWROC
402	Diethanolamine	Diethanolamine	ROCIOXY
404	Diethylamine	Diethylamine	HC10
405	Diethylcyclohexane	1,4-Diethylcyclohexane	HC10
406	Diethylene glycol	Diethylene glycol	ROCIOXY
407	Diethylene glycol butyl ether acetate (or 2-2-(butoxyethoxy)ethyl acetate)	2-(2-Butoxyethoxy)ethyl acetate	ROCIOXY
411	Diisobutyl ketone	Diisobutyl ketone	KET
412	Diisopropyl adipate	Diisopropyl adipate	ROCIOXY
413	Diisopropylene glycol	1,1'-Oxybis-2-propanol	ROCIOXY
415	Dimethoxymethane	Dimethoxymethane	HC5
417	Dimethyl ether	Dimethyl ether	HC3
418	Dimethyl formamide	N,N-Dimethylformamide	HC3
419	Dimethyl phthalate	Dimethyl phthalate	ROCP5ARO
420	Dimethyl succinate	Dimethyl succinate	HC3
421	Dimethyl sulfide	Dimethyl sulfide	HC5
422	Dimethyl sulfoxide	Dimethyl sulfoxide	ROCIOXY
425	Dimethylcyclopentane	Cyclopentane, 1,3-dimethyl-, (1R,3S)-rel-	HC10
428	Dimethylheptanes	2,4-Dimethylheptane	HC10
430	Dimethylundecane	2,6-Dimethylundecane	HC10
432	Dipropylene glycol	1,1'-Oxybis-2-propanol	ROCIOXY
434	Mineral spirits	Methylcyclohexane	HC10
435	Dl-limonene	Limonene	LIM
438	Ethane	Ethane	ETH
439	Ethanolamine	Ethanolamine	ROCIOXY
440	Ethyl acetate	Ethyl acetate	HC3
441	Ethyl acrylate	Ethyl acrylate	OLT
442	Ethyl alcohol (or ethanol)	Ethanol	EOH
443	Ethyl chloride (or Chloroethane)	Chloroethane	HC3
444	Ethyl cyanoacrylate	Ethyl cyanoacrylate	ROCP6ARO
445	Ethyl ether	Diethyl ether	HC10
446	Ethyl mercaptan	Ethanethiol	HC10

ID	Species	Representative Compound	CRACMM
447	Ethyl propylcyclohexanes	cyclohexane, 1-ethyl-2-propyl-	HC10
448	Ethyl-3-ethoxypropionate	Ethyl 3-ethoxypropionate	HC10
449	Ethylbenzene	Ethylbenzene	XYE
450	Ethylcyclohexane	Ethylcyclohexane	HC10
451	Ethylcyclopentane	Ethylcyclopentane	HC10
452	Ethylene (or ethene)	Ethylene	ETE
453	Ethylene dibromide (or 1,2-Dibromomethane)	1,2-Dibromoethane	SLOWROC
454	Ethylene dichloride (or 1,2-dichloroethane)	1,2-Dichloroethane	SLOWROC
455	Ethylene glycol	Ethylene glycol	ETEG
456	Ethylene glycol butyl ether acetate (or 2-butoxyethyl acetate)	2-Butoxyethyl acetate	HC10
459	Ethylene oxide	Ethylene oxide	SLOWROC
461	Ethylmethylcyclohexanes	Cyclohexane, ethylmethyl-	HC10
463	Ethyloctane	Octane, 3-ethyl-	HC10
464	Ethyltoluenes (or methylethylbenzenes)	4-Ethyltoluene	XYE
465	Formaldehyde	Formaldehyde	HCHO
466	Formic acid	Formic acid	ORA1
467	Fragrances	D-Limonene	LIM
469	Gamma- butyrolactone (or Dihydro-2(3H)-furanone || 4-Hydroxybutanoic acid lactone)	4-Butyrolactone	ROCP6ARO
470	Glutaraldehyde (or a dialdehyde)	Glutaraldehyde	DCB1
472	Glyceryl triacetate	Triacetin	ROCIOXY
473	Glycol ether dpnb (or 1-(2-butoxy-1-methylethoxy)-2-propanol)	1-[(1-Butoxy-2-propanyl)oxy]-2-propanol	ROCIOXY
474	Glycolic acid (or hydroxyacetic acid)	Glycolic acid	ROCIOXY
478	1,1-Difluoroethane	1,1-Difluoroethane	SLOWROC
479	Hexachlorobenzene	Hexachlorobenzene	SLOWROC
480	Hexylcyclohexane	Hexylcyclohexane	ROCP6ALK
482	Hexylene glycol (or 2-methyl-2,4-pentanediol)	2-Methyl-2,4-pentanediol	ROCIOXY
483	Hydrocarbon propellant (LPG, sweetened)	Propane	HC3
484	Hydrocarbon propellant (LPG)	Propane	HC3
485	Indane (or Indan || Benzocyclopentane || Hydrindene; Indene, 2,3-dihydro- || 1,2-Hydrindene || 2,3-Dihydroindene || 2,3-Dihydro-1H-indene)	Indan	XYE
486	Indene	Indene	XYM
490	Isobornyl acetate (or 2-camphanyl acetate)	Isobornyl acetate	ROCIOXY
491	Isobutane (or 2-Methylpropane)	Isobutane	HC3
492	Isobutyl acetate	Isobutyl acetate	HC5
493	Isobutyl alcohol	2-Methyl-1-propanol	ROH
494	Isobutyl isobutyrate	Isobutyl isobutyrate	HC10
496	Isobutylcyclopentane	Isobutylcyclopentane	HC10
497	Isobutylene	Isobutene	OLT
498	Isocyanic acid (or Isocyanate)	Isocyanic acid	OLI
499	Isomers of butylbenzene	Butylbenzene	XYE
500	Isomers of decane	Decane	HC10
502	Isomers of diethylbenzene	1,4-Diethylbenzene	XYE
503	Isomers of dodecane	Dodecane	ROCP6ALK
504	Isomers of tridecane	Tridecane	ROCP6ALK
505	Isomers of undecane	Undecane	HC10
507	Isomers of xylene	m-Xylene	XYM
508	Isopentane (or 2-Methylbutane)	2-Methylbutane	HC5
510	Isophorone (or 3,5,5-trimethyl-2-cyclohexenone)	Isophorone	OLI
511	Isoprene	Isoprene	ISO
512	Isopropyl acetate	Isopropyl acetate	HC3
513	Isopropyl alcohol (or 2-Propanol)	Isopropanol	ROH
514	Isopropylbenzene (or cumene || 2-Phenylpropane)	Cumene	XYE
515	Isopropylcyclohexane (or 1-methylethylcyclohexane)	Cyclohexane, (1-methylethyl)-	HC10
517	Isovaleraldehyde	3-Methylbutanal	ALD
522	M & p-xylene (or m,p-xylene)	m-Xylene	XYM
523	M-cresol (or 3-methyl-benzenol)	m-Cresol	CSL
524	M-xylene	m-Xylene	XYM
527	Menthol	dl-Menthol	ROCIOXY
531	Methyl alcohol (or methanol)	Methanol	MOH
532	Methyl amyl ketone	2-Heptanone	KET
533	Methyl bromide (or Bromomethane)	Methyl bromide	SLOWROC
534	Methyl carbitol (or 2-(2-methoxyethoxy)ethanol || degme)	Diethylene glycol monomethyl ether	ROCIOXY
535	Methyl chloride (or Chloromethane)	Chloromethane	SLOWROC

ID	Species	Representative Compound	CRACMM
536	Methyl ethyl ketone (or MEK || 2-butanone)	Methyl ethyl ketone	MEK
537	Methyl ethyl ketoxime	2-Butanone oxime	HC5
538	Methyl hexane	2-Methylhexane	HC5
539	Methyl isobutyl ketone (or 4-Methyl-2-pentanone || Hexone)	4-Methyl-2-pentanone	KET
540	Methyl mercaptan	Methanethiol	HC10
541	Methyl methacrylate	Methyl methacrylate	OLT
542	Methyl n-butyl ketone (or 2-hexanone)	2-Hexanone	KET
544	Methyl propyl ketone (or 2-pentanone)	2-Pentanone	KET
545	Methyl propylcyclohexanes	1-Methyl-3-propyl-cyclohexane	HC10
547	Methyl styrene (mixed) (or vinyl toluene)	alpha-Methylstyrene	XYM
548	Methyl tert-butyl ether (or Methyl t-butyl ether || MTBE)	Methyl tert-butyl ether	HC3
550	Methylcyclohexane	Methylcyclohexane	HC10
551	Methylcyclopentane	Methylcyclopentane	HC10
552	Methyldecalins	1-methyldecahydronaphthalene	HC10
553	Methyldecene	2-Methyl-1-decene	OLT
554	Methylene(b)4-phenylisocyanate (or methylene diphenyl diisocyanate)	4,4'-Diphenylmethane diisocyanate	ROCP2ALK
557	Methyltri(ethylmethylketoxime) silane	Methyltri(2-butanoneoximyl)silane	ROCP5ARO
558	Methyltrimethoxysilane	Trimethoxymethylsilane	ROCIOXY
559	Methylvinylbis(n-methylacetamido) silane	Acetamide, N,N'-(ethenylmethylsilylene)bis[N-methyl-	ROCP6ARO
560	Mineral oil	Linoleic acid	ROCP1ALK
562	Misc. acids	Decanoic acid	ROCIOXY
563	Misc. alcohols	Pent-1-en-1-ol	OLI
568	Misc. esters	Propyl acetate	HC3
570	Misc. glycol ethers and acetates	2-Methoxyethanol	ROH
572	Misc. hydrocarbon propellants	Propane	HC3
574	Misc. lvp VOC distillates	Heptadecane	ROCP3ALK
578	Misc. silanes	Fluorotrimethylsilane	ROCIOXY
588	Monoterpenes	alpha-Pinene	API
589	Morpholine	Morpholine	HC10
590	N,n-dimethylethanolamine	Dimethylaminoethanol	ROH
592	N-butane	Butane	HC3
593	N-butyl acetate	Butyl acetate	HC5
594	N-butyl acrylate	Butyl acrylate	OLT
595	N-butyl alcohol (or 1-Butanol)	1-Butanol	ROH
596	N-butylbenzene	Butylbenzene	XYE
598	N-decane	Decane	HC10
599	N-dodecane	Dodecane	ROCP6ALK
600	N-heptane	Heptane	HC10
601	N-hexane	n-Hexane	HC5
602	N-hexylbenzene	Benzene, hexyl-	ROCP6ARO
603	N-nonane	Nonane	HC10
604	N-octane	Octane	HC10
605	N-pentane	Pentane	HC5
606	N-pentylbenzene	Pentylbenzene	XYE
607	N-propyl alcohol (or 1-Propanol)	1-Propanol	ROH
608	N-propylbenzene	Propylbenzene	XYE
609	N-tridecane	Tridecane	ROCP6ALK
610	N-undecane	Undecane	HC10
611	Naphthalene	Naphthalene	NAPH
614	Nitromethane	Nitromethane	SLOWROC
616	Nonadiene	1,8-Nonadiene	FURAN
618	O-cresol (or 2-Methylphenol)	o-Cresol	CSL
619	O-dichlorobenzene (or 1,2-Dichlorobenzene)	1,2-Dichlorobenzene	XYE
620	O-xylene	o-Xylene	XYE
621	Octahydroindenes	trans-Octahydro-1H-indene	HC10
626	Organic carbon	Triacontane	ROCN2ALK
636	Other exempt propellants	1,1,1-Trichloro-2,2,2-trifluoroethane	SLOWROC
637	Other glycol ethers	2-Butoxyethanol	ROH
638	Other, lumped VOCs, individually < 2% of category	Decane	HC10
640	Misc./other VOC -duplicate	Decane	HC10
641	Other, misc. VOC compounds aggregated in profile	Decane	HC10
642	Other, misc. exempt compounds aggregated in profile	Acetone	ACT
646	P-cresol (4-methyl phenol)	p-Cresol	CSL
647	P-dichlorobenzene (or 1,4-Dichlorobenzene)	1,4-Dichlorobenzene	SLOWROC

ID	Species	Representative Compound	CRACMM
648	P-xylene	p-Xylene	XYE
652	Parachlorobenzotrifluoride	1-Chloro-4-(trifluoromethyl)benzene	SLOWROC
655	Pentamethylbenzene	Benzene, pentamethyl-	ROCP5ARO
656	Pentanedioic acid, dimethyl ester	Dimethyl glutarate	ROCIOXY
657	Pentylcyclohexane	Pentylcyclohexane	ROCP6ALK
661	Perchloroethylene (or Tetrachloroethylene)	Tetrachloroethylene	SLOWROC
663	Phenol (or carbolic acid)	Phenol	PHEN
664	Phenoxyethanol	2-Phenoxyethanol	CSL
667	Pine oil	alpha-Pinene	API
671	Propane	Propane	HC3
672	Propenylcyclohexane	Cyclohexene, 1-(2-propenyl)-	FURAN
673	Propionaldehyde (or Propanal || 1-Propanone || 1-Propanal)	Propanal	ALD
674	Propyl acetate	Propyl acetate	HC3
676	Propylcyclohexane	Propylcyclohexane	HC10
677	Propylcyclopentane	Propylcyclopentane	HC10
678	Propylene (or Propene || 1-Propene)	1-Propene	OLT
679	Propylene carbonate	Propylene carbonate	ROCIOXY
680	Propylene glycol	1,2-Propylene glycol	PROG
681	Propylene glycol butyl ether (or 1-butoxy-2-propanol)	1-Butoxy-2-propanol	ROH
682	Propylene glycol methyl ether (or 1-methoxy-2-propanol)	1-Methoxy-2-propanol	ROH
684	Propylene glycol monomethyl ether acetate (or 2-(1-methoxy)propyl acetate)	1-Methoxy-2-propyl acetate	HC10
685	Propylene glycol n-propyl ether	1-Propoxy-2-propanol	ROH
686	Propylene glycol t-butyl ether (or 1-(1,1,-dimethylethoxy)-2-propanol)	1-tert-Butoxy-2-propanol	ROH
687	Propylene oxide	1,2-Propylene oxide	HC3
692	Sec-butyl alcohol (or 2-butanol)	2-Butanol	ROH
698	Styrene	Styrene	XYM
703	T-butylbenzene	tert-Butylbenzene	XYE
705	Terpene	D-Limonene	LIM
706	Tert-butyl alcohol	tert-Butyl alcohol	ROH
707	Tetrahydrofuran	Tetrahydrofuran	HC10
709	Tetramethylcyclopentane	1,1,2,2-Tetramethylcyclopentane	HC10
711	Tetramethylthiourea	1,1,3,3-Tetramethyl-2-thiourea	ROCP6ALK
716	m-Tolualdehyde (or m-Methylbenzaldehyde || 3-Methylbenzaldehyde)	Benzaldehyde, 3-methyl-	BALD
717	Toluene	Toluene	TOL
720	Cis-1,trans-2,trans-4-trimethylcyclohexane -duplicate	1,2,4-Trimethylcyclohexane	HC10
721	Trans,trans-1,2,4-trimethylcyclohexane	1,2,4-Trimethylcyclohexane	HC10
722	Trans,trans-1,3,5-trimethylcyclohexane	1,3,5-Trimethylcyclohexane	HC10
723	Trans-1,2-dichloroethene	(E)-1,2-Dichloroethylene	OLI
724	Trans-1,2-dimethylcyclohexane	1,trans-2-Dimethylcyclohexane	HC10
725	Trans-1,2-dimethylcyclopentane	(rel)-trans-1,2-Dimethylcyclopentane	HC10
726	Trans-1,3-dimethylcyclohexane	(+/-)-trans-1,3-Dimethylcyclohexane	HC10
727	Trans-1,3-dimethylcyclopentane	trans-1,3-Dimethylcyclopentane	HC10
728	Trans-1,3-pentadiene	(3E)-1,3-Pentadiene	FURAN
729	Trans-1,4-dimethylcyclohexane	trans-1,4-Dimethylcyclohexane	HC10
730	Trans-1,cis-2,3-trimethylcyclopentane	rel-(1R,3R)-1,2,3-Trimethylcyclopentane	HC10
732	Trans-1-ethyl-2-methylcyclohexane	CYCLOHEXANE, 1-ETHYL-2-METHYL-, TRANS-	HC10
736	Trans-1-ethyl-3-methylcyclopentane -duplicate	CYCLOPENTANE, 1-ETHYL-3-METHYL-, TRANS-	HC10
737	Trans-2-butene	(2E)-2-Butene	OLI
739	Trans-2-heptene	(2E)-2-Heptene	OLI
740	Trans-2-hexene	(2E)-2-Hexene	OLI
741	Trans-2-octene	(2E)-2-Octene	OLI
742	Trans-2-pentene	(2E)-2-Pentene	OLI
743	Trans-3-heptene	(3E)-3-Heptene	OLI
744	Trans-3-hexene	(3E)-3-Hexene	OLI
745	Trans-3-nonene	TRANS-3-NONENE	OLI
746	Trans-4-octene	(4E)-4-Octene	OLI
747	Trichloroethylene	Trichloroethylene	OLI
748	Trichlorofluoromethane	Trichlorofluoromethane	SLOWROC
749	Trichlorotrifluoroethane-F113	1,1,2-Trichloro-1,2,2-trifluoroethane	SLOWROC
750	Triethanolamine	Triethanolamine	ROCP1ALK
751	Triethylamine	Triethylamine	HC10
755	Misc. trimethylbenzenes -duplicate	1,2,4-Trimethylbenzene	XYM

ID	Species	Representative Compound	CRACMM
760	Trimethyloctanes	Octane, trimethyl-	HC10
761	Turpentine	alpha-Pinene	API
766	Urethane prepolymer	4,4'-Diphenylmethane diisocyanate	ROCP2ALK
768	Vinyl acetate	Vinyl acetate	OLT
769	Vinyl chloride	Vinyl chloride	OLT
770	Vinylacetylene (or Butenyne || Ethynylethene || 1-Butenyne || Vinylethyne)	1-Buten-3-yne	OLT
771	Vinyltrimethoxysilane	Vinyltrimethoxysilane	OLT
772	VOC ingredients	Decane	HC10
773	Volatile methyl siloxanes	Decamethylcyclopentasiloxane	ROCIOXY
774	Witch hazel	Decane	HC10
776	Xylenol	2,6-Dimethylphenol	CSL
839	Glyoxal	Glyoxal	GLY
840	Hexaldehyde (or hexanal || Hexanaldehyde)	Hexanal	ALD
845	Valeraldehyde (or n-Pentanal || 1-pentanal)	Pentanal	ALD
846	Acenaphthene	Acenaphthene	NAPH
847	Acenaphthylene	Acenaphthylene	NAPH
849	Anthraquinone (or Anthradione || Hoelite || Morkit || 9,10-Anthraquinone)	Anthraquinone	ROCP0ALK
852	Anthracene	Anthracene	NAPH
854	Benz(a)anthracene	Benz(a)anthracene	NAPH
855	Benzo[a]pyrene BaP	Benzo(a)pyrene	NAPH
859	Bibenzyl	Bibenzyl	NAPH
860	Biphenyl	Biphenyl	NAPH
867	Chrysene	Chrysene	NAPH
871	1,4-dimethylnaphthalene; 1,5-dimethylnaphthalene; 2,3-dimethylnaphthalene	1,4-Dimethylnaphthalene	NAPH
873	Dibenzofuran (or DBZFUR)	Dibenzofuran	FURAN
881	9-fluorenone (or Fluorenone)	9-Fluorenone	ROCP5ARO
882	Fluoranthene	Fluoranthene	NAPH
883	Fluorene	Fluorene	NAPH
886	1-methylphenanthrene	1-Methyl phenanthrene	NAPH
889	2-methylphenanthrene	2-Methylphenanthrene	NAPH
902	Phenanthrene	Phenanthrene	NAPH
903	Perinaphthenone (or Phenalenone || 7-Perinaphthenone || 1H-phenalen-1-one)	Phenalen-1-one	ROCP2ALK
904	Pyrene	Pyrene	NAPH
905	Retene	Retene	NAPH
909	Xanthone	Xanthone	ROCP2ALK
934	Acetovanillone (or acetva)	Acetovanillone	ROCP2ALK
935	2-methoxy-4-(2-propenyl)phenol (or eugenol || â€œa4guclâ€□ || 4-Allylguaiacol)	Eugenol	CSL
937	Benzoic acid	Benzoic acid	ROCP5ARO
941	Decanoic acid	Decanoic acid	ROCIOXY
947	Guaiacol	2-Methoxyphenol	CSL
950	Hexanoic acid	Hexanoic acid	ROCIOXY
951	Hexanedioic acid	Hexanedioic acid	ROCP0ALK
954	Lauric acid (or dodecanoic acid)	Dodecanoic acid	ROCP2ALK
956	2-methoxy-4-methylphenol (or 4-methylguaiacol || m4gucl)	2-Methoxy-4-methylphenol	CSL
957	4-methyl-syringol (or m4syrg)	4-Methyl-2,6-dimethoxyphenol	ROCP2ALK
958	Myristic acid(or n-Tetradecanoic Acid)	Tetradecanoic acid	ROCP1ALK
961	Palmitic acid	Hexadecanoic acid	ROCP1ALK
962	Pentadecanoic acid	Pentadecanoic acid	ROCP1ALK
966	Stearic acid (or Octadecanoic Acid)	Octadecanoic acid	ROCP1ALK
970	Tridecanoic acid	Tridecanoic acid	ROCP2ALK
976	Acetophenone (or 1-phenylethanone || Methyl phenyl ketone)	Acetophenone	ROCP6ARO
977	Beta-pinene	beta-Pinene	API
981	Butylbenzene	Butylbenzene	XYE
992	Benzonitrile (or Benzoic acid nitrile || Cyanobenzene || Phenyl cyanide || Benzenenitrile)	Benzonitrile	SLOWROC
996	1-decene	1-Decene	OLT
997	Decanal	Decanal	ROCIOXY
998	2-decanone	2-Decanone	ROCIOXY
1001	1,2-dihydronaphthalene	1,2-Dihydronaphthalene	ROCP6ARO
1002	1,3-diisopropylbenzene	1,3-Diisopropylbenzene	XYM
1003	1,4-diisopropylbenzene	1,4-Diisopropylbenzene	ROCP6ARO

ID	Species	Representative Compound	CRACMM
1007	Dodecene	1-Dodecene	ROCP6ARO
1012	2-methylbenzofuran (or 2-Methyl-1-benzofuran)	2-Methyl-1-benzofuran	FURAN
1013	2,3-benzofuran (or Benzofurfuran || Benzo[b]furan || Coumarone || 1-Oxindene)	2,3-Benzofuran	FURAN
1018	Heptanal	Heptanal	ALD
1030	Dl-limonene -duplicate	Limonene	LIM
1036	4-methylstyrene	4-Methylstyrene	XYM
1042	Eicosane	Eicosane	ROCP2ALK
1043	Heptadecane	Heptadecane	ROCP3ALK
1045	Hexadecane	Hexadecane	ROCP4ALK
1047	Nonadecane	Nonadecane	ROCP3ALK
1048	Octadecane	Octadecane	ROCP4ALK
1049	Pentadecane	Pentadecane	ROCP5ALK
1051	Tetradecane	Tetradecane	ROCP5ALK
1057	Nonanal	Nonanal	ROCIOXY
1065	Octanal	Octanal	ALD
1079	1,2,3,4-tetrahydronaphthalene	Tetralin	ROCP6ARO
1082	1-undecene	1-Undecene	OLT
1083	Alpha-pinene	alpha-Pinene	API
1093	1-butene & isobutene	1-Butene	OLT
1098	1,2,4-trimethylbenzene & t-butylbenzene	1,2,4-Trimethylbenzene	XYM
1118	1,3-hexadiene (trans)	Hexa-1,4-diene	FURAN
1125	Isopropyltoluene	p-Cymene	ROCP6ARO
1153	3-methyl-2-pentene	2-Pentene, 3-methyl-	OLI
1161	Propyltoluene	p-Propyltoluene	XYE
1170	Triphenylene	Triphenylene	NAPH
1172	Benzo[ghi]fluoranthene	Benzo[ghi]fluoranthene	NAPH
1173	Cyclopenta[cd]pyrene	Cyclopenta[cd]pyrene	NAPH
1462	p-Tolualdehyde	4-Methylbenzaldehyde	BALD
1463	2,3-Butanedione (or Biacetyl || Butane-2,3-dione || Butanedione || Diacetyl || Dimethyl diketone || Dimethyl glyoxal)	2,3-Butanedione	SLOWROC
1464	Methylglyoxal	Methyl glyoxal	MGLY
1465	1-Dodecene	1-Dodecene	ROCP6ARO
1466	1-Tridecene	1-Tridecene	ROCP6ARO
1467	o-Tolualdehyde	2-Tolualdehyde	BALD
1468	2,5-Dimethylaldehyde	1,1-Dimethoxyethane	HC10
1469	2,2-Dimethyl-3-ethylpentane	3-Ethyl-2,2-dimethylpentane	HC10
1471	4-ethylheptane	Heptane, 4-ethyl-	HC10
1472	Cis,trans,cis-1,2,3-Trimethylcyclohexane	1,2,3-Trimethylcyclohexane	HC10
1473	1-Methyl-3-isopropylcyclohexane -duplicate	1-Methyl-3-(propan-2-yl)cyclohexane	HC10
1474	trans-1-methyl-3-propylcyclohexane	1-Methyl-3-propyl-cyclohexane	HC10
1476	1-Methyl-2-isopropylcyclohexane -duplicate	1-Methyl-2-(propan-2-yl)cyclohexane	HC10
1477	Cyclohexane, 1,2,4-trimethyl-, (1Î±,2Î²,4Î±)-	1,3,5-Trimethylcyclohexane	HC10
1478	Trans-1-ethyl-3-methylcyclohexane -duplicate	Cyclohexane, ethylmethyl-	HC10
1479	Bicyclo[3.3.1]nonane	Bicyclo(3.3.1)nonane	HC10
1480	Cis,cis,cis-1,2,3-trimethylcyclohexane	1,2,3-Trimethylcyclohexane	HC10
1482	Trans octahydro Indene	trans-Octahydro-1H-indene	HC10
1484	Cyclohexane, 1-ethyl-2,3-dimethyl- -duplicate	1-Ethyl-2,4-dimethylcyclohexane	HC10
1485	1,1,2,3-tetramethylcyclohexane -duplicate	cyclohexane, 1,1,2,3-tetramethyl-	HC10
1486	Cyclohexane, 1-methyl-4-propyl, trans	1-methyl-4-propylcyclohexane	HC10
1487	3-ethylnonane -duplicate	3-Ethylnonane	HC10
1488	Heptane, 2,3,4-trimethyl	2,3,4-Trimethylheptane	HC10
1490	1-methyl-2-propyl cyclopentane	1-Methyl-2-propylcyclopentane	HC10
1491	Cyclohexane, 1,3-diethyl, cis	(1R,3S)-1,3-Diethylcyclohexane	HC10
1492	4-ethyl Nonane	4-ethylnonane	HC10
1499	Cyclohexane, 1,3-diethyl, trans	cyclohexane, 1-ethyl-2-propyl-	HC10
1501	u-Paraffin, C10	Octane, 3-ethyl-	HC10
1502	u-Paraffin, C9	Nonane	HC10
1503	c-Paraffin, C10	Decane	HC10
1504	i-Paraffin, C10	Octane, 3-ethyl-	HC10
1505	i-Paraffin, C11	Undecane	HC10
1506	u-Paraffin, C11	Undecane	HC10
1507	u-Paraffin, C12	Dodecane	ROCP6ALK
1508	c-Paraffin, C11	Undecane	HC10
1516	4-methyl dodecane	4-Methyldodecane	ROCP6ALK

ID	Species	Representative Compound	CRACMM
1518	3,6-dimethyl decane	3,6-Dimethyldecane	HC10
1521	3,7-dimethyl decane	3,7-Dimethyldecane	HC10
1522	3,8-dimethyl decane	3,8-Dimethyldecane	HC10
1523	5-ethyl nonane	Nonane, 5-ethyl	HC10
1527	Cyclohexane, 1,4-diethyl, trans	1,4-Diethylcyclohexane	HC10
1529	Cyclohexane, 1,4-diethyl, cis	1,4-Diethylcyclohexane	HC10
1536	Cis-1-ethyl-3-methylcyclopentane -duplicate	CYCLOPENTANE, 1-ETHYL-3-METHYL-, CIS-	HC10
1537	cis,trans,cis-1,2,3-trimethylcyclopentane	1,2,3-Trimethylcyclopentane	HC10
1539	2,4-dimethylhexane -duplicate	2,4-Dimethylhexane	HC5
1540	Cis,trans,cis-1,2,4-trimethylcyclopentane	1,2,4-Trimethylcyclopentane	HC10
1541	1,3-diethyl, trans cyclopentane	1,3-Diethylcyclopentane	HC10
1547	p-xylene, 2-propyl-	1,4-Dimethyl-2-propylbenzene	XYM
1548	5-propyl-m-xylene	1,3-Dimethyl-5-propylbenzene	XYM
1550	4,5-dimethyldecane -duplicate	4,5-Dimethyldecane	HC10
1551	Cyclohexane, 1-methyl-2-propyl, trans	1-Methyl-2-propyl-cyclohexane	HC10
1554	4-propyl-o-xylene (or 1,2-dimethyl-4-propylbenzene)	1,2-Dimethyl-4-propylbenzene	XYM
1555	4-propyl-m-xylene	1,2-Dimethyl-4-propylbenzene	XYM
1556	1,3-Diethyl-4-methylbenzene	1,3-Diethyl-4-methylbenzene	XYM
1557	4,7-dimethyl-2,3,dihydro-1-h-indenes	4,7-Dimethylindan	ROCP6ARO
1558	3,5-Diethyltoluene (or 1,3-Diethyl-5-methylbenzene)	3,5-Diethyltoluene	XYM
1560	C10 aromatics -duplicate	Naphthalene	NAPH
1561	Dimethyl indan	2,4-Dimethyl-2,3-dihydro-1H-indene	ROCP6ARO
1562	1-ethyl-2,4,5-trimethyl benzene (or 1,2,4-trimethyl-5-ethylbenzene)	Benzene, 1-ethyl-2,4,5-trimethyl-	XYE
1563	Toluene, 3,4-diethyl-	1-Methyl-3,4-diethylbenzene	XYM
1564	1-ethyl-2,3,5-trimethyl benzene (or 1,2,5-Trimethyl-3-ethylbenzene)	Benzene, 1-ethyl-2,3,5-trimethyl-	XYE
1565	2-ethyl-1,3,4-trimethyl benzene (or 1,2,4-Trimethyl-3-ethylbenzene)	2-ethyl-1,3,5-trimethylbenzene	XYE
1566	Dimethyl, isopropyl benzene	Benzene, 2,4-dimethyl-1-(1-methylethyl)-	XYM
1567	Unknown C11 aromatics	1,3-Dimethyl-5-propylbenzene	XYM
1569	Ethyl isopropyl benzene	Benzene, ethyl(1-methylethyl)-	XYM
1572	2-ethyl-mesitylene (or 1,3,5-trimethyl-2-ethylbenzene)	2-ethyl-1,3,5-trimethylbenzene	XYE
1573	1,2-dimethyl-3-propyl benzene	Benzene, 1,2-dimethyl-3-propyl-	XYE
1574	1,3-diethyl-2-methyl benzene	Benzene, 1,3-diethyl-2-methyl-	XYE
1575	2-ethenyl-1,4-dimethyl benzene	Benzene, 2-ethenyl-1,4-dimethyl-	XYM
1576	1,3-dimethyl-2-propyl benzene	1,3-Dimethyl-2-propylbenzene	XYE
1577	1,2-dimethyl-4-ethenyl benzene	Benzene, 4-ethenyl-1,2-dimethyl-	XYM
1579	1-ethyl-2,4-dimethylcyclohexane -duplicate	1-Ethyl-2,4-dimethylcyclohexane	HC10
1583	4-ethyldecane -duplicate	4-Ethyldecane	HC10
1584	1-Decene, 6-ethyl	1-Decene, 4-ethyl	OLT
1586	trans-1-Ethyl-2-methylcyclopentane -duplicate	Cyclopentane, 1-ethyl-2-methyl-, (1R,2R)-rel-	HC10
1588	1,1,3,3-Tetramethylcyclopentane	1,1,3,3-Tetramethylcyclopentane	HC10
1594	Cis, cis, trans-1,2,4-trimethylcyclohexane -duplicate	1,2,4-Trimethylcyclohexane	HC10
1595	N-heneicosane	Heneicosane	ROCP3ALK
1596	N-docosane	Docosane	ROCP1ALK
1597	n-Tricosane	Tricosane	ROCP2ALK
1598	n-Tetracosane	Tetracosane	ROCP2ALK
1599	n-Pentacosane	Pentacosane	ROCP1ALK
1604	n-Triacontane	Triacontane	ROCN2ALK
1614	methyl vanillate	Benzoic acid, 4-hydroxy-3-methoxy-, methyl ester	ROCP2ALK
1617	Octanoic acid	Octanoic acid	ROCIOXY
1618	Nonanoic acid	Nonanoic acid	ROCIOXY
1619	Undecanoic acid	Undecanoic acid	ROCIOXY
1620	Heptadecanoic acid	Heptadecanoic acid	ROCP0ALK
1629	Sandaracopimaric acid	Methyl 7-ethenyl-4a,7-dimethyl-1,2,3,4,4a,4b,5,6,7,9,10,10a-dodecahydrophenanthrene-1-carboxylate	ROCP1ALK
1635	4-formyl-guaiacol -duplicate	4-Hydroxy-3-methoxybenzaldehyde	BALD
1641	Beta-Amyrin	Hopane	ROCN2ALK
1649	3-Ethylpentene	3-Ethyl-1-pentene	OLT
1651	2-Butene	2-Butene	OLI
1652	Methylindane	1-Methyl-2,3-dihydro-1H-indene	ROCP6ARO
1654	Cis-2-Nonene	Non-2-ene	OLI
1655	1-Methyl-2-tert-butylbenzene -duplicate	Benzene, 1-(1,1-dimethylethyl)-2-methyl-	XYE
1658	Undecanal	Undecanal	ROCP6ALK

ID	Species	Representative Compound	CRACMM
1659	Dodecanal	Dodecanal	ROCP5ALK
1660	Tridecanal	trans-2-Tridecenal	ROCP5ARO
1661	Tetradecanal	Tetradecanal	ROCP5ALK
1662	Pentadecanal	Pentadecanal	ROCP5ALK
1663	Hexadecanal	Hexadecanal	ROCP4ALK
1664	Heptadecanal	Heptadecanal	ROCP3ALK
1665	2-Nonanone	2-Nonanone	KET
1666	2-Undecanone	2-Undecanone	ROCP6ALK
1667	2-Tridecanone	2-Tridecanone	ROCP5ALK
1668	2-Pentadecanone	2-Pentadecanone	ROCP5ALK
1669	2-tetradecanone	Tetradecan-2-one	ROCP5ALK
1670	Furfural	Furfural	FURAN
1671	2-Decenal	2-Decenal	API
1672	2-undecenal	2-Undecenal	ROCP5ARO
1673	Heptanoic acid	Heptanoic acid	ROCIOXY
1674	Heptadecan-2-one	2-Heptadecanone	ROCP4ALK
1675	5-butyldihydro-2(3H)-furanone	4-Hydroxyoctanoic acid lactone	ROCIOXY
1676	G-nonanoic lactone -duplicate	gamma-Nonanolactone	ROCIOXY
1677	G-decanolactone -duplicate	gamma-Decanolactone	ROCIOXY
1678	5-ethyldihydro-2(3H)-furanone	gamma-Caprolactone	HC5
1679	5-propyldihydro-2(3H)-furanone	gamma-Heptalactone	ROCIOXY
1690	2,6,10-Trimethyldodecane (or farnesane)	2,6,10-Trimethyldodecane	ROCP6ALK
1691	Undecane, 2,6,10-trimethyl -duplicate	Undecane, 2,6,10-trimethyl-	HC10
1692	2,6,10-trimethyltridecane	2,6,10-Trimethyltridecane	ROCP5ALK
1693	Norpristane	2,6,10-Trimethylpentadecane	ROCP5ALK
1694	N-Nonylcyclohexane	Cyclohexane, nonyl-	ROCP4ALK
1695	Decylcyclohexane	Decylcyclohexane	ROCP4ALK
1697	3-methylphenanthrene	3-Methylphenanthrene	NAPH
1698	2-methylanthracene	2-Methylanthracene	NAPH
1699	9-methylphenanthrene	9-Methylphenanthrene	NAPH
1700	C2-MW 178 PAH	Anthracene	NAPH
1701	C3-MW 178 PAH	Anthracene	NAPH
1702	Acephenanthrylene	Acephenanthrylene	NAPH
1703	C1-MW 202 PAH	Pyrene	NAPH
1704	Pristane	Norphytane	ROCP4ALK
1705	Phytane	2,6,10,14-Tetramethylhexadecane	ROCP4ALK
1706	C3-naphthalenes	1-Ethyl-7-methylnaphthalene	NAPH
1707	C4-naphthalenes	Naphthalene, 2,6-diethyl-	NAPH
1708	N-Pentadecylcyclohexane	Cyclohexane, pentadecyl-	ROCP1ALK
1709	8B,13a-dimethyl-14B-n-butylpodocarpane	Decane	HC10
1711	M- & p-tolualdehyde	4-Methylbenzaldehyde	BALD
1712	2,5-Dimethylbenzaldehyde	2,5-Dimethylbenzaldehyde	BALD
1713	1-Indanone	1H-Inden-1-one, 2,3-dihydro-	ROCP6ARO
1714	Dibenzothiophene	Dibenzothiophene	NAPH
1716	Undecylcyclohexane	Undecylcyclohexane	ROCP4ALK
1717	Dodecylcyclohexane	Cyclohexane, dodecyl-	ROCP3ALK
1718	tridecylcyclohexane	Cyclohexane, tridecyl-	ROCP2ALK
1728	Nonadecanedioic acid	Nonadecanedioic acid	ROCN1ALK
1731	Heptadecylcyclohexane	Heptadecylcyclohexane	ROCP2ALK
1732	octadecylcyclohexane	Cyclohexane, octadecyl-	ROCP2ALK
1734	Eicosylcyclohexane	Cyclohexane, eicosyl-	ROCP0ALK
1745	20R&S-5a(H),14B(H),17B(H)-sitostane	Stigmastane	ROCP0ALK
1746	P-diethylbenzene & n-butylbenzene	1,4-Diethylbenzene	XYE
1747	5-Methyl-2-furaldehyde (or 5-methyl-2-furanaldehyde || 5-Methylfurfural || 2-Furancarboxaldehyde, 5-methyl-)	2-Furancarboxaldehyde, 5-methyl-	FURAN
1748	Hydroxymethylfurfural	5-(Hydroxymethyl)-2-furfural	FURAN
1750	1,2-dimethoxy-4-methyl benzene	Benzene, 1,2-dimethoxy-4-methyl-	ROCP5ARO
1751	2-oxobutanal	Butanal, 2-oxo-	MGLY
1752	Phenol, 2-methoxy-4-propenyl-, (E)- (or trans-iso-eugenol)	(E)-Isoeugenol	CSL
1753	Propylgyaiacol -duplicate	2-Methoxy-4-propylphenol	CSL
1754	4-ethyl-2-methoxyphenol -duplicate	4-Ethyl-2-methoxyphenol	CSL
1755	1,2-Benzenediol	1,â€‹2-â€‹Benzenediol	PHEN
1756	Hydroquinone (or p-benzenediol || 1,4-Benzenediol)	Hydroquinone	ROCP2ALK
1757	Resorcinol (or m-benzenediol || 1,3-Benzenediol)	Resorcinol	PHEN
1759	1,6-Dimethylnaphthalene	1,6-Dimethylnaphthalene	NAPH

ID	Species	Representative Compound	CRACMM
1762	Hydroxybenzaldehydes	p-Hydroxybenzaldehyde	BALD
1763	Guaiacylacetone -duplicate	2-Propanone, 1-(4-hydroxy-3-methoxyphenyl)-	ROCP2ALK
1764	4-ethylsyringol -duplicate	Phenol, 4-ethyl-2,6-dimethoxy-	ROCP2ALK
1768	Syringyl acetone	2-Propanone, 1-(4-hydroxy-3,5-dimethoxyphenyl)-	ROCP2ALK
1777	3,4-dimethoxybenzaldehyde (or veratraldehyde)	Veratraldehyde	BALD
1786	Dodecenal	2-â€‹Dodecenal	ROCP5ARO
1787	Tridecanal -duplicate	trans-2-Tridecenal	ROCP5ARO
1788	Tetradecenal	(Z)-9-Tetradecenal	ROCP5ARO
1789	Pentadecenal	2,4-pentadecadienal	ROCP5ARO
1790	Heptadecan-2-one -duplicate	2-Heptadecanone	ROCP4ALK
1800	Neophytadiene	7,11,15-Trimethyl-3-methylidenehexadec-1-ene	ROCP5ARO
1801	C2-Naphthalenes	2,6-Dimethylnaphthalene	NAPH
1802	C1-MW 178 PAH	Anthracene	NAPH
1803	Solanone	(E)-6,10-Dimethylundeca-5,9-dien-2-one	ROCP6ARO
1804	Geranyl acetone	(E)-6,10-Dimethylundeca-5,9-dien-2-one	ROCP6ARO
1805	Nicotine	Nicotine	ROCP6ARO
1806	Bipyridyl	2,2'-Bipyridine	ROCP5ARO
1807	Cotinine	Cotinine	ROCP5ARO
1808	Carbazole	Carbazole	NAPH
1809	Indole	Indole	ROCP5ARO
1810	Nornicotine	Pyridine, 3-(2S)-2-pyrrolidinyl-	ROCP5ARO
1811	Phenylpyridine	4-Phenylpyridine	ROCP5ARO
1812	Quinoline	Quinoline	ROCP6ARO
1813	Isoquinoline	Isoquinoline	ROCP6ARO
1814	2-Ethylphenol	2-Ethylphenol	CSL
1815	Ethenylphenol	2-Ethenylphenol	CSL
1816	2,5-Pyrrolidinedione, 1-methyl- (or N-Methylsuccinimide)	2,5-Pyrrolidinedione, 1-methyl-	HC3
1817	Beta-Nicotyrine	Pyridine, 3-(1-methyl-1H-pyrrol-2-yl)-	ROCP5ARO
1818	1-Methylindole	1H-Indole, 1-methyl-	ROCP6ARO
1819	Pyrolo[2,3-b]pyridine	1H-Pyrrolo[2,3-b]pyridine	ROCP5ARO
1820	5-(Hydroxymethyl)-2-furaldehyde (or 2-Furancarboxaldehyde, 5-(hydroxymethyl)- || 5-Hydroxymethylfurfural)	5-(Hydroxymethyl)-2-furfural	FURAN
1825	Pyruvic acid	Pyruvic acid	KET
1828	Furancarboxylic acid	2-Furancarboxylic acid	FURAN
1830	Anteiso-triacontane	Isotriacontane	ROCN2ALK
1836	2-(2-butoxyethoxy)ethanol -duplicate	2-(2-Butoxyethoxy)ethanol	ROCIOXY
1837	2,2,4-Trimethyl-1,3-pentanediol diisobutryate	2,2,4-Trimethyl-1,3-pentanediol diisobutyrate	ROCIOXY
1838	2,2,4-trimethyl-1,3-pentanediol isobutyrate -duplicate	1-Hydroxy-2,2,4-trimethylpentan-3-yl 2-methylpropanoate	ROCIOXY
1840	Heptylcyclohexane	Heptylcyclohexane	ROCP6ALK
1841	Octylcyclohexane	Cyclohexane, octyl-	ROCP5ALK
1843	Tetradecylcyclohexane	Cyclohexane, tetradecyl-	ROCP2ALK
1845	8B,13a-dimethyl-14B-[3'-methylbutyl]podocarpane -duplicate	8,13-Dimethyl-14-(3-methylbutyl)podocarpane	ROCP3ALK
1879	4-Methylcyclopentene	Cyclopentene, 4-methyl-	OLI
1880	Methylsyringol	Benzene, 1,2,3-trimethoxy-	ROCP5ARO
1883	Methyl fluorene	2-Methylfluorene	NAPH
1886	1-Methylcyclohexene	Cyclohexene, 1-methyl-	OLI
1887	1-Nitropropane	1-Nitropropane	HC3
1888	Dichloronitroaniline	Dicloran	ROCP1ALK
1891	2-Ethyl hexanol	2-Ethyl-1-hexanol	ROCIOXY
1892	2-methyl-3-hexanone	2-Methyl-3-hexanone	KET
1894	3,4-dimethyloctane -duplicate	2,3-Dimethyloctane	HC10
1896	4,4-Methylene dianiline	4,4'-Diaminobiphenyl methane	NAPH
1897	4-Chloro-3,5-xylenol	4-Chloro-3,5-dimethylphenol	CSL
1898	4-Methylaniline	N-Methylaniline	ROCP6ARO
1899	4-Phenyl-1-butene	3-Butenylbenzene	XYM
1901	Acetic anhydride	Acetic anhydride	HC3
1902	Acetonitrile	Acetonitrile	SLOWROC
1903	Acrylic acid	Acrylic acid	OLT
1904	Alpha-terpineol	alpha-Terpineol	API
1905	Aminoanthraquinone	1-Aminoanthraquinone	ROCN1ALK

ID	Species	Representative Compound	CRACMM
1906	Aniline	Aniline	ROCP6ARO
1909	Benzyl chloride	Benzyl chloride	XYE
1914	B-phellandrene	beta-Phellandrene	LIM
1915	Bromodinitroaniline	2-Bromo-4,6-dinitroaniline	ROCP2ALK
1916	Bromodinitrobenzene	1-Bromo-3,5-dinitrobenzene	ROCP5ARO
1918	Butoxybutane	Butyl ether	HC10
1920	Butyl benzoate	Butyl benzoate	ROCP5ARO
1921	Butylisopropylphthalate	Butyl isobutyl phthalate	ROCP5ARO
1923	C10 Aromatic	Naphthalene	NAPH
1924	C-10 Compounds	Octane, 3-ethyl-	HC10
1925	C10 Olefins	1-Decene	OLT
1926	C10 Paraffins	Octane, 3-ethyl-	HC10
1929	C-11 Compounds	Undecane	HC10
1930	C11 Olefins	3-methyl-1-decene	OLT
1932	C12 Olefins	1-Dodecene	ROCP6ARO
1934	C13-Branched alkane	2-Methyldodecane	ROCP6ALK
1936	C14-Branched alkane	2-Methyltridecane	ROCP6ALK
1938	C15-Branched alkane	2-Methyltetradecane	ROCP5ALK
1939	C16 Branched alkane	2-Methylpentadecane	ROCP4ALK
1941	C16 Branched alkane -duplicate	2-Methylpentadecane	ROCP4ALK
1943	C-18 Compounds	Octadecane	ROCP4ALK
1945	C2 Alkyl indan	2-Ethylindan	ROCP6ARO
1947	C2 Cyclohexane	1,2-Dimethylcyclohexane	HC10
1963	C3 Cyclohexane	1,3,5-Trimethylcyclohexane	HC10
1964	C3/C4/C5 Alkylbenzenes	p-Cymene	ROCP6ARO
1975	C-3-Hexene	1-Hexene	OLT
1976	C-4 Compounds	Butane	HC3
1977	C4 Substituted cyclohexane	cyclohexane, 1-ethyl-1,4-dimethyl-, trans-	HC10
1978	C4 Substituted cyclohexanone	4-N-Butylcyclohexanone	ROCIOXY
1983	C4-Alkylphenols	4-Butylphenol	CSL
1985	C4-Benzene	p-Cymene	ROCP6ARO
1986	C-5 Compounds	Pentane	HC5
1987	C5 Cyclohexane	Pentylcyclohexane	ROCP6ALK
1988	C5 Ester	Ethyl propionate	HC3
1989	C5 Olefin	1-Pentene	OLT
1990	C5 Paraffin	Pentane	HC5
1992	C5 Substituted cyclohexane	Pentylcyclohexane	ROCP6ALK
1993	C5-Alkylbenzenes	Pentylbenzene	XYE
1995	C5-Alkylphenols	4-Pentylphenol	CSL
1996	C5-Benzene	Pentylbenzene	XYE
1997	C5-Cyclohexane	Pentylcyclohexane	ROCP6ALK
1998	C5-Ene	1-Pentene	OLT
1999	C-6 Compounds	n-Hexane	HC5
2000	C6 Olefins	1-Hexene	OLT
2001	C6 Substituted cyclohexane	Hexylcyclohexane	ROCP6ALK
2003	C6H18O3SI3	Hexamethylcyclotrisiloxane	ROCIOXY
2005	C-7 Compounds	Heptane	HC10
2006	C-7 Cycloparaffins	1,2-Dimethylcyclopentane	HC10
2008	C7 Paraffins	Heptane	HC10
2009	C7-C16 Paraffins	Decane	HC10
2011	C-8 Compounds	Octane	HC10
2012	C-8 Cycloparaffins	Cyclooctane	HC10
2013	C-8 Olefins	1-Octene	OLT
2014	C8 Paraffin	Octane	HC10
2015	C8 Phenols	Methyl salicylate	CSL
2017	C8H24O4SI4	Octamethylcyclotetrasiloxane	ROCIOXY
2018	C-9 Compounds	Nonane	HC10
2019	C-9 Cycloparaffins	Cyclooctane, methyl-	HC10
2020	C9 Olefins	1-Nonene	OLT
2022	C9 Phenols	4-Propylphenol	CSL
2023	Camphene	(+)-Camphene	API
2024	Carbaryl	Carbaryl	ROCP1ALK
2026	Chloropentafluoroethane	Chloropentafluoroethane	SLOWROC
2027	Chloroprene	Chloroprene	FURAN
2029	Chlorotrifluoromethane	Chlorotrifluoromethane	SLOWROC

ID	Species	Representative Compound	CRACMM
2034	Creosote	m-Cresol	CSL
2036	Cyclopentylcyclopentane	Bicyclopentyl	HC10
2037	Decalins	Decalin	HC10
2039	Denaturant	Methanol	MOH
2040	Di(ethylphenyl) ethane	Benzene, 1,1'-ethylidenebis(4-ethyl-	NAPH
2045	DI-C8 Alkyl phthalate	Bis(6-methylheptyl) phthalate	ROCP2ALK
2046	1,2-dichloro 1,1,2,2-tetrafluoroethane	1,2-Dichloro-1,1,2,2-tetrafluoroethane	SLOWROC
2050	Dihydroxyacetone	Dihydroxyacetone	ROCIOXY
2052	Diisopropyl benzene	Benzene, 1,2-bis(1-methylethyl)-	XYM
2053	Dimethyl alkyl amines	Ethanamine, N-methyl-	HC10
2054	Dimethyl napthalene	2,6-Dimethylnaphthalene	NAPH
2055	Dimethyl terephthalate	Dimethyl terephthalate	ROCP5ARO
2057	Dimethylamine	Dimethylamine	HC10
2061	Dimethylcyclohexane	1,4-Dimethylcyclohexane	HC10
2067	Dimethylhexanes	3,3-Dimethylhexane	HC5
2068	Dimethylhexene	2,3-Dimethylhex-2-ene	OLI
2072	Dimethylnonanes -duplicate	2-Methyldecane	HC10
2073	Dimethyloctanes -duplicate	2-Methylnonane	HC10
2079	Dipropyl phthalate	Di-n-propylphthalate	ROCP5ARO
2081	Divinyl benzene	1,4-Divinylbenzene	XYM
2083	Epichlorohydrin (or 2-(Chloromethyl)oxirane)	Epichlorohydrin	HC3
2084	Ethylstyrene	4-Ethylstyrene	XYM
2089	Ethyldimethylcyclohexane	2-Ethyl-1,1-dimethylcyclohexane	HC10
2091	Ethyleneamines	Vinylamine	OLT
2094	Ethylheptene	3-Ethyl-3-heptene	OLI
2097	Ethylisopropyl ether	Propane, 2-ethoxy-	HC10
2098	Ethylmethylcyclopentane	Cyclopentane, 1-ethyl-1- methyl-	HC10
2099	Ethylmethyloctane	5-â€‹ethyl-â€‹2-â€‹methyl-octane	HC10
2100	Ethyloctene	3-Ethyl-3-octene	OLI
2102	Ethyl-phenyl-phenyl-ethane	1-Ethyl-2-(1-phenylethyl)benzene	NAPH
2103	Ethyl propylcyclohexanes -duplicate	cyclohexane, 1-ethyl-2-propyl-	HC10
2105	Furfuryl alcohol (or 2-Furanmethanol || 2-Furylmethanol || 2-(Hydroxymethyl)furan)	Furfuryl alcohol	FURAN
2108	Heptene	1-Heptene	OLT
2109	Hexachloroethane	Hexachloroethane	SLOWROC
2111	Hexafluoroethane	Perfluoroethane	SLOWROC
2112	Hexamethylcyclotrisiloxane	Hexamethylcyclotrisiloxane	ROCIOXY
2113	Hexamethylenediamine	1,6-Hexanediamine	ROCP6ALK
2114	Hexenal	Hexobarbital	ROCP1ALK
2116	Hexyne	1-Hexyne	HC10
2117	Isoamyl alcohol (or 3-Methyl-1-butanol)	Isopentyl alcohol	ROH
2118	Isobutyl acrylate	Isobutyl acrylate	OLT
2119	Isobutyraldehyde (or Î±-Methylpropionaldehyde || Isobutanal || Isopropylaldehyde || Isopropylformaldehyde || 2-Methylpropanal || 2-Methyl-1-propanal)	2-Methylpropanal	ALD
2120	Isomers of butene	1-Butene	OLT
2121	Isomers of C10H18	Decalin	HC10
2123	Isomers of C9H16	1-Nonyne	HC10
2124	Isomers of ethyltoluene	4-Ethyltoluene	XYE
2125	Isomers of heptadecane	Heptadecane	ROCP3ALK
2126	Isomers of heptane	Heptane	HC10
2127	Isomers of hexane	n-Hexane	HC5
2128	Isomers of nonane	Nonane	HC10
2129	Isomers of octadecane	Octadecane	ROCP4ALK
2130	Isomers of octane	Octane	HC10
2131	Isomers of pentadecane	Pentadecane	ROCP5ALK
2132	Isomers of pentane	Pentane	HC5
2133	Isomers of pentene	1-Pentene	OLT
2134	Isomers of propylbenzene	Propylbenzene	XYE
2135	Isomers of tetradecane	Tetradecane	ROCP5ALK
2137	Ketones - general	Pentanal	ALD
2138	Lactol spirits	Heptane	HC10
2140	Maleic anhydride	2,5-Furandione	ROCP6ARO
2144	Methyl acrylate	Methyl acrylate	OLT
2145	Methyl biphenyl	4-Phenyltoluene	NAPH

ID	Species	Representative Compound	CRACMM
2146	Methyl C11 ester	Methyl decanoate	ROCIOXY
2148	Methyl C13 ester	Methyl dodecanoate	ROCIOXY
2149	Methyl C14 ester	Methyl tridecanoate	ROCIOXY
2150	Methyl C15 ester	Methyl tetradecanoate	ROCIOXY
2151	Methyl C19 ester	Methyl stearate	ROCP2ALK
2152	Methyl C20 ester	Methyl nonadecan-1-oate	ROCP2ALK
2153	Methyl dodecanoate	Methyl dodecanoate	ROCIOXY
2154	Methyl formate	Methyl formate	SLOWROC
2157	Methylnaphthalenes -duplicate	1-Methylnaphthalene	NAPH
2158	Methyl palmitate	Methyl hexadecanoate	ROCIOXY
2159	Methyl octadecanoate	Methyl stearate	ROCP2ALK
2160	Methyl acetate	Methyl acetate	SLOWROC
2161	1,2-butadiene -duplicate	1,2-Butadiene	FURAN
2164	Methylbenzaldehyde	4-Methylbenzaldehyde	BALD
2170	Methylcyclooctane	Cyclooctane, methyl-	HC10
2172	Methyldecanes	4-Methyldecane	HC10
2174	Methyldodecane	2-Methyldodecane	ROCP6ALK
2175	Methylene bromide	Dibromomethane	SLOWROC
2184	Methyl hexane -duplicate	2-Methylhexane	HC5
2185	Methylhexenes	2-Methylpent-1-ene	OLT
2186	Methylindans	1-Methyl-2,3-dihydro-1H-indene	ROCP6ARO
2188	Isopropylmethylcyclohexane -duplicate	cyclohexane, 1-isopropyl-1-methyl-	HC10
2191	Methylnonane	2-Methylnonane	HC10
2192	Methylnonene	2-Methyl-2-nonene	OLI
2193	Methyloctanes	2-Methyloctane	HC10
2194	Methylpentane	3-Methylpentane	HC5
2197	Methylpropylcyclohexanes	1-Methyl-3-propyl-cyclohexane	HC10
2198	Methylpropylnonane	5-(Butan-2-yl)nonane	HC10
2199	Methylundecane	2-Methylundecane	HC10
2201	Myrcene	Myrcene	LIM
2203	Naphtha	n-Hexane	HC5
2206	Nitrobenzene	Nitrobenzene	SLOWROC
2207	Nonenone	3-Nonen-2-one	OLI
2209	N-pentylcyclohexane	Pentylcyclohexane	ROCP6ALK
2211	Octamethylcyclotetrasiloxane	Octamethylcyclotetrasiloxane	ROCIOXY
2215	Oxygenates	2-(Hydroxymethoxy)ethanol	ROCIOXY
2216	Paraffins (C16-C34)	Pentacosane	ROCP1ALK
2217	Paraffins/Olefins (C12-C16)	Tetradecane	ROCP5ALK
2220	Pentadiene	(3E)-1,3-Pentadiene	FURAN
2225	Pentyne	1-Pentyne	HC10
2227	Phenyl isocyanate	Phenyl isocyanate	XYE
2228	Phthalic anhydride	Phthalic anhydride	ROCP5ARO
2230	Piperylene	1,3-Pentadiene	FURAN
2233	Propionic acid (or Propanoic acid || Carboxyethane || Ethanecarboxylic acid || Ethylformic acid || Luprisol)	Propionic acid	ORA2
2234	1,1-dichloropropane (or Dichloropropane)	1,1-Dichloropropane	HC3
2235	Propylene glycol phenyl ether	2-Phenoxy-1-propanol	CSL
2240	Siloxane	Decamethylcyclopentasiloxane	ROCIOXY
2242	Substituted C9 ester (C12)	3-Hydroxy-2,2,4-trimethylpentyl 2-methylpropanoate	ROCIOXY
2243	Trans-1-phenylbutene	(E)-1-Phenyl-1-butene	XYM
2244	Trans-2-nonene	(2E)-2-Nonene	OLI
2246	Terephthalic acid (or 1,4-Benzenedicarboxylic Acid)	Terephthalic acid	ROCP2ALK
2248	Terpenes	alpha-Pinene	API
2250	Tetrachlorobenzenes	1,2,4,5-Tetrachlorobenzene	SLOWROC
2252	Tetrafluoromethane	Carbon tetrafluoride	SLOWROC
2254	Tetramethylcyclobutene	2-Ethenyl-1,1-dimethylcyclobutane	OLT
2256	Total aromatic amines	Aniline	ROCP6ARO
2257	Total C2-C5 aldehydes	Propanal	ALD
2258	Trans-1,3-dichloropropene	trans-1,3-Dichloropropene	OLI
2259	Trichlorobenzenes	1,2,4-Trichlorobenzene	XYE
2261	Triethylene glycol	Triethylene glycol	ROCIOXY
2262	Triethylene glycol monobutyl ether	2-[2-(2-Butoxyethoxy)ethoxy]ethanol	ROCIOXY
2263	Trifluoromethane	Trifluoromethane	SLOWROC
2266	Trimethyldecene	3,3-Dimethyldec-1-ene	OLT

ID	Species	Representative Compound	CRACMM
2267	Trimethylfluorosilane	Fluorotrimethylsilane	ROCIOXY
2268	Trimethylheptanes	2,2,4-Trimethylheptane	HC10
2278	UNC peaks to CBM xylene	o-Xylene	XYE
2279	Undefined aromatic	1,2,4-Trimethylbenzene	XYM
2283	Undefined VOC	Decane	HC10
2284	Unidentified	Decane	HC10
2285	Unknown #1	Decane	HC10
2295	Xylene base acids	o-Xylene	XYE
2297	Unknown	Decane	HC10
2313	3-methyloctane; 3,3-diethylpentane; 3-ethylheptane	3-Methyloctane	HC10
2329	1-tert-butyl-4-ethylbenzene	p-tert-Butylethylbenzene	XYM
2333	Propylcyclopentane -duplicate	Propylcyclopentane	HC10
2334	Isooctane	2,2,4-Trimethylpentane	HC5
2335	o-Vinyltoluene (or 1-Methyl-2-vinylbenzene || 2-Methylstyrene || 2-Vinyltoluene || 1-Ethenyl-2-methylbenzene || 2-Ethenylmethylbenzene || 2-Methyl-1-vinylbenzene)	o-Vinyltoluene	XYM
2336	Chrysene;Triphenylene	Chrysene	NAPH
2337	2,2'-Dithiobisbenzothiazole	2,2'-Dithiobisbenzothiazole	NAPH
2338	Xylenol -duplicate	2,6-Dimethylphenol	CSL
2339	Methyl benzenediols	4-Methylcatechol	MCT
2341	Cis-iso-eugenol	Isoeugenol	CSL
2355	Diethyl phthalate	Diethyl phthalate	ROCP5ARO
2367	M & p-cresol (or 3-Methylphenol & 4-Methylphenol)	m-Cresol	CSL
2368	Dicyclopentadiene	Dicyclopentadiene	FURAN
2372	1,2,4-Trichlorobenzene	1,2,4-Trichlorobenzene	XYE
2560	Cyclopentane, (1-methylethyl)- -duplicate	Isopropylcyclopentane	HC10
2562	Methyl vinyl ketone (or 2-Butenone || 1-Buten-3-one || Butenone || 3-Butenen-2-one)	Methyl vinyl ketone	MVK
2564	Methyl isopropyl ketone (or Isopropyl methyl ketone || Ketone, isopropyl methyl || Methyl butanone-2 || 3-Methyl-2-butanone)	3-Methyl-2-butanone	KET
2568	2-methyl-butyl-benzene (or 1-phenyl-2-methylbutane)	Isopentylbenzene	XYM
2637	Cyclopentanol	Cyclopentanol	ROH
2638	Heptanone	2-Heptanone	KET
2639	Octanone	2-Octanone	KET
2640	Furan	Furan	FURAN
2641	2-methyl-furan	2-Methylfuran	FURAN
2642	3-methyl-furan	3-Methylfuran	FURAN
2643	2-ethylfuran	Furan, 2-ethyl-	FURAN
2644	2,4-dimethyl-furan	Furan, 2,4-dimethyl-	FURAN
2645	2,5-dimethyl-furan	2,5-Dimethylfuran	FURAN
2646	2,3-dihydrofuran	Furan, 2,3-dihydro-	OLI
2647	Methyl iodide	Methyl iodide	SLOWROC
2669	Particulate Non-Carbon Organic Matter	Triacontane	ROCN2ALK
2673	2,6-dimethylheptane, propylcyclopentane	2,6-Dimethylheptane	HC10
2681	4-methyl-cis-2-pentene; 2-methylpentane (isohexane)	2-pentene, 4-methyl-, (z)-	OLI
2684	Allylbenzene (or 1-Phenyl-2-propene; 1-Propene, 3-phenyl-; 2-Propenylbenzene; 3-Phenyl-1-propene; 3-Phenylpropene)	benzene, 2-propenyl-	XYM
2688	3-Hydroxy-2-butanone (or Acetoin)	Acetoin	ROCIOXY
2692	Dimethyl Disulfide	Methyl disulfide	HC10
2693	1,2-Dichloroethene	(Z)-1,2-Dichloroethylene	OLI
2694	2,4,6-Trichlorophenol	2,4,6-Trichlorophenol	PHEN
2695	2,4-Dinitrophenol	2,4-Dinitrophenol	PHEN
2696	2,4-Dinitrotoluene	2,4-Dinitrotoluene	ROCP5ARO
2697	2-Nitrophenol (or o-Nitrophenol)	2-Nitrophenol	PHEN
2698	3-Carene	3-Carene	API
2699	4,6-Dinitro-o-cresol	2-Methyl-4,6-dinitrophenol	CSL
2700	4-Nitrophenol	4-Nitrophenol	PHEN
2701	Bis(2-chloroisopropyl) ether	bis(2-Chloroisopropyl)ether	HC10
2702	2-Chlorophenol	2-Chlorophenol	PHEN
2703	Decachlorobiphenyl	Decachlorobiphenyl	SLOWROC
2704	Dichlorobiphenyl	4,4'-Dichlorobiphenyl	NAPH
2705	Di-n-octyl phthalate	Di-n-octyl phthalate	ROCP0ALK
2706	2,2',4,4',5,5'-Hexachlorobiphenyl	2,2',4,4',5,5'-Hexachlorobiphenyl	NAPH
2707	Hexachlorocyclopentadiene	Hexachlorocyclopentadiene	ROCP6ARO
2708	2,3,3',4,4'-Pentachlorobiphenyl	2,3,3',4,4'-Pentachlorobiphenyl	NAPH
2709	Pentachlorophenol	Pentachlorophenol	SLOWROC

ID	Species	Representative Compound	CRACMM
2710	Tetrachlorobiphenyl	2,2',4,4'-Tetrachlorobiphenyl	NAPH
2711	2,2',3-Trichlorobiphenyl	2,2',3-Trichlorobiphenyl	NAPH
2712	Gamma-Terpinene	gamma-Terpinene	LIM
2713	1,2-Dimethoxyethane	Ethylene glycol dimethyl ether	HC10
2723	Heptanol	1-Heptanol	ROCIOXY
2724	3-Pentanone	3-Pentanone	KET
2754	Butanoic acid (or Butyric acid)	Butanoic acid	ORA2
2755	2-Isopropyl-5-methylanisole (or Methyl thymol ether)	2-Isopropyl-5-methylanisole	ROCP6ARO
2756	Bornyl acetate	Bornyl acetate	ROCIOXY
2757	Pinene	alpha-Pinene	API
2758	Eucalyptol	1,8-Cineol	HC10
2759	Heptyl Hexanoate	Heptyl hexanoate	ROCIOXY
2760	3-Methyl-butanoic acid	Isovaleric acid	ROCIOXY
2761	2-Methyl-propanoic acid	2-Methylpropanoic acid	ORA2
2762	1-Methyl cycloheptene	1-Methylcycloheptene	OLI
2763	Pentanoic acid	Pentanoic acid	ROCIOXY
2764	Thujen-2-one (or Umbellulon || 4-Methyl-1-(propan-2-yl)bicyclo[3.1.0]hex-3-en-2-one)	Umbellulone	OLI
2796	Î±-Methylstyrene	alpha-Methylstyrene	XYM
2811	Tetradecene	1-Tetradecene	ROCP5ARO
2812	cis-1,3-Pentadiene	(3Z)-1,3-Pentadiene	FURAN
2815	Octadiene	Octa-1,6-diene	FURAN
2830	trans-1-Phenyl-1-butene (or Trans-1-butenylbenzene)	(E)-1-Phenyl-1-butene	XYM
2831	1,2,3-trimethylnaphthalene	1,2,3-Trimethylnaphthalene	NAPH
2936	Dimethyl itaconate (or Itaconic acid, dimethyl ester || Methylenesuccinic acid, dimethyl ester)	Dimethyl itaconate	OLI
2937	Diethyl itaconate (or Itaconic acid diethyl ester || 2-methylene, diethyl ester || Butanedioic acid, methylene-, diethyl ester)	Ethyl itaconate	ROCP6ARO
2939	4-Ethylphenol (or 1-Hydroxy-4-ethylbenzene || p-Ethylphenol)	4-Ethylphenol	CSL
2940	p-Propylphenol (or Dihydrochavicol || 4-Propylphenol || p-Hydroxypropylbenzene)	4-Propylphenol	CSL
2941	Acetamide (or Acetic acid amide || Ethanamide || Methanecarboxamide)	Acetamide	ROCIOXY
2942	3-Methylindole (or Scatole || Skatol || 3-Methyl-1H-indole)	3-Methylindole	ROCP5ARO
2943	4,5-Dimethyloxazole (or 5-Methyl-4-methyloxazole)	4,5-Dimethyloxazole	FURAN
2944	2,4,5-Trimethyloxazole	2,4,5-Trimethyloxazole	OLI
2945	2,3,5,6-Tetramethylpyrazine	Tetramethylpyrazine	FURAN
2946	Dimethyl sulfone	Dimethyl sulfone	ROCIOXY
2948	Isopropylcyclobutane	Isopropylcyclobutane	HC10
2949	1,2-Pentadiene	Penta-1,2-diene	FURAN
2950	Hexadiene	1,5-Hexadiene	FURAN
2951	Allyl alcohol (or Allylic alcohol || 1-Propen-3-ol || 2-Propenol || 2-Propenyl alcohol)	Allyl alcohol	OLT
2952	2-Pentanol (or Methyl butanol || 2-Pentyl alcohol)	Pentan-2-ol	ROH
2953	2-Phenyl-2-propanol (or Î±-Cumyl alcohol || 2-Phenylisopropanol || Î±,Î±-Dimethylbenzyl alcohol)	2-Phenylpropan-2-ol	CSL
2954	3-Hexanone (or Ethyl propyl ketone || Hexan-3-one)	3-Hexanone	KET
2955	2-Methylbutanal (or Î±-Methylbutyric aldehyde || Methylethylacetaldehyde || 2-Formylbutane)	2-Methylbutanal	ALD
2956	1,3,5-Trichlorobenzene	1,3,5-Trichlorobenzene	ROCP6ARO
2957	1-Propanamine (or n-Propylamine)	Propylamine	HC10
2998	Dimethylbenzaldehyde	2,3-Dimethylbenzaldehyde	BALD
2999	Hydrogen cyanide (or Hydrocyanic acid || Formonitrile)	Hydrogen cyanide	SLOWROC
3000	Ethyl formate (or Ethylformic ester || Ethyl ester formic acid)	Ethyl formate	HC3
3001	cis-1,3-hexadiene	(E)-1,3-Hexadiene	FURAN
3002	Ethylpyrazine (or 2-Ethylpyrazine)	Ethylpyrazine	FURAN
3003	1,6-Heptadiyne	1,6-Heptadiyne	HC10
3005	Glycolaldehyde (or Diose || Glycolic aldehyde || Hydroxyacetaldehyde || Methylol formaldehyde)	Acetaldehyde, hydroxy-	GLY
3006	1,1-Dimethylhydrazine (or Dimazine)	1,1-Dimethylhydrazine	HC10
3007	Propanenitrile (or Propionitrile || Cyanoethane || Ether cyanatus || Ethyl cyanide || Hydrocyanic ether || Propionic nitrile)	Propionitrile	SLOWROC
3008	Carbon suboxide (or 1,2-Propadiene-1,3-dione || Carbon oxide)	Carbon suboxide	FURAN
3009	Pyrrole (or Azole || Divinylenimine || Imidole || Monopyrrole)	Pyrrole	FURAN
3010	1,3-Cyclopentadiene, methyl- (or Methyl-1,3-cyclopentadiene || Methylcyclopenta-1,3-diene || Methylcyclopentadiene || Monomethylcyclopentadiene)	1,3-Cyclopentadiene, methyl-	FURAN

ID	Species	Representative Compound	CRACMM
3011	1-Methyl-1,3-cyclopentadiene -duplicate	1,3-Cyclopentadiene, methyl-	FURAN
3012	2-Methyl-1,3-cyclopentadiene	2-Methyl-1,3-cyclopentadiene	FURAN
3013	2,5-Dihydrofuran (or 1-Oxa-3-cyclopentene || 3-Oxolene)	2,5-Dihydrofuran	OLI
3014	2-Cyclopenten-1-one (or Cyclopenten-3-one || Cyclopentenone || 2-Cyclopentenone 3-Cyclopenten-2-one; 2-Cyclopentenone-1; cyclopenten-2-one)	Cyclopent-2-en-1-one	FURAN
3015	2,3-Dihydro-1,4-dioxine	1,4-Dioxin, 2,3-dihydro-	OLI
3016	Methyl propionate (or Propanoic acid, methyl ester)	Methyl propanoate	HC3
3017	1-Penten-3-yne	1-Penten-3-yne	OLT
3018	1-Methylpyrrole (or Pyrrole, 1-methyl- || N-Methylpyrrole || 1-Methyl-1H-pyrrole)	1-Methylpyrrole	FURAN
3019	1-Penten-3-one (or Ethyl vinyl ketone)	Ethyl vinyl ketone	OLT
3020	Cyclopentanone (or Adipic ketone || Adipinketon || Dumasin || Ketocyclopentane || Ketopentamethylene)	Cyclopentanone	OLI
3021	2-Methyl-2-butenal (or 2-Methylcrotonaldehyde || 2,3-Dimethylacrolein || 2-Methylbut-2-enal)	2-Methylbut-2-enal	UALD
3022	3-Methylpyridazine	3-Methylpyridazine	FURAN
3023	4-Methylpyridazine	4-Methylpyridazine	FURAN
3024	3-Furaldehyde	3-Furaldehyde	FURAN
3025	3-Cyclopentene-1,2-dione	Cyclopent-3-ene-1,2-dione	FURAN
3026	Butyric acid, methyl ester (or Methyl butanoate || Methyl butyrate)	Methyl butyrate	HC3
3027	1,5-Hexadien-3-yne (or Divinylacetylene)	Divinylacetylene	FURAN
3028	1-Hexen-3-yne (or Ethylvinylacetylene || Vinylethylacetylene)	1-Hexen-3-yne	OLT
3029	Pyrazole, 1-methyl- (or 1-Methylpyrazole)	1H-Pyrazole, 1-methyl-	OLI
3030	Ethynyl Benzene (or Phenylacetylene || 1-Phenylethyne || Acetylene, phenyl- || Ethinylbenzene)	Phenylacetylene	XYM
3031	m-Methylstyrene (or m-Vinyltoluene || 1-Methyl-3-vinylbenzene || 3-Methylstyrene || 3-Vinyltoluene || Benzene, 1-ethenyl-3-methyl-)	3-Methylstyrene	XYM
3032	3-Methyl-1-benzofuran	3-Methylbenzofuran	FURAN
3033	1-Methyl-2-benzofuran	1-Methyl-2-benzofuran	FURAN
3035	1,4-Dihydronaphthalene	1,4-Dihydronaphthalene	ROCP6ARO
3036	1-Phenyl-1-butene (or Î²-Ethylstyrene || 1-Butenyl-benzene)	(E)-1-Phenyl-1-butene	XYM
3037	(E)-(1-Methylpropenyl)benzene (or trans-2-Phenyl-2-butene)	BENZENE, (1-METHYL-1-PROPENYL)-, (E)-	XYM
3038	p-Mentha-1,4(8)-diene (or Terpinolene||Terpinolen||Î±- Terpinolen||Î±-Terpinolene||4-Isopropylidene-1-methyl-cyclohexene p-Menth-1,4(8)-diene|| 1-methyl-4-(1-methylethylidene)-cyclohexene (Î±-terpinolene)||1-Methyl-4-(1-methylethylidene)-cyclohexene)	Terpinolene	LIM
3039	Isolimonene (or 3-Isopropenyl-6-methyl-cyclohexene || trans-Isolimonene || (3R-trans)-3-methyl-6-(1-methylvinyl)cyclohexene)	(3R-trans)-3-Methyl-6-(1-methylvinyl)cyclohexene	LIM
3040	Cadinene (or Sesquiterpene || Naphthalene, decahydro-1,6-dimethyl-4-(1-methylethyl)-, (1S,4S,4aS,6S,8aS)-, didehydro deriv)	Cadinene	SESQ
3054	Diethylenetriamine	Diethylenetriamine	ROCP6ALK
3056	3-Methoxy-1-Butanol (or 3-Methoxybutanol || Methoxybutanol || 3-methoxybutan-1-ol)	3-Methoxybutan-1-ol	ROH
3073	Formamide (or Carbamaldehyde; Methanamide || Amid kyseliny mravenci || Formimidic acid)	Formamide	ROCIOXY
3079	2,2,4,4,6,8,8-Heptamethylnonane	2,2,4,4,6,8,8-Heptamethylnonane	ROCP6ALK
3096	3,7-Dimethylocta-1,6-Dien-3-Ol	Linalool	LIM
3098	Ethylene Glycol Monohexyl Ether	2-Hexyloxyethanol	ROCIOXY
3100	Hydroxyethyl Methacrylate	2-Hydroxyethyl methacrylate	ROCP6ARO
3104	Ethyltriacetoxysilane	Ethyltriacetoxysilane	ROCIOXY
3129	Alkyl (C16-C18) Methyl Esters	Methyl pentadecanoate	ROCIOXY
3135	3-Aminopropyl-Triethoxysilane	3-Aminopropyltriethoxysilane	ROCIOXY
3148	2- Pyrrolidone	2-Pyrrolidinone	ROCP5ARO
3153	Tetrahydrofurfuryl Methacrylate	Tetrahydrofurfuryl methacrylate	ROCP6ARO
3156	2-(Methylamino)-2-methyl-1-propanol	2-methyl-2-(methylamino)propan-1-ol	ROCIOXY
3157	Dipropylene Glycol Monopropyl Ether	Butyl dipropasol solvent	ROCIOXY
3158	2-Ethylhexyl Benzoate	2-Ethylhexyl benzoate	ROCP5ARO
3162	4,4-Dimethyloxazolidine	4,4-Dimethyl oxazolidine	HC10
3164	Dimethylhexanedioate -duplicate	Dimethyl adipate	ROCIOXY
3165	Dipropylene Glycol Methyl Ether Acetate	1-Methoxy-2-propyl acetate	HC10
3168	Ethyl Lactate	Ethyl lactate	ROH
3171	Hexahydro-1,3,5-tris(2-hydroxyethyl)-s-triazine	Triazinetriethanol	ROCN1ALK
3175	Nitroethane	Nitroethane	SLOWROC
3179	Triethoxyoctylsilane	Triethoxyoctylsilane	ROCIOXY

ID	Species	Representative Compound	CRACMM
3181	Troysan 174	2-(Hydroxymethylamino)ethanol	ROCIOXY
3183	Tributyl phosphate	Tributyl phosphate	ROCIOXY
3186	1,1,1,2-Tetrafluoroethane (or HFC-134a)	1,1,1,2-Tetrafluoroethane	SLOWROC
3191	Methyltriacetoxysilane	Methylsilanetriyl triacetate	ROCIOXY
3195	Methoxysilane	Silane, methoxy-	ROCIOXY
3196	Branched C10 Alkanes	2-Methylnonane	HC10
3197	Branched C11 alkanes	2-Methyldecane	HC10
3198	Branched C12 Alkanes	2-Methylundecane	HC10
3199	Branched C17 Alkanes	2-Methylhexadecane	ROCP4ALK
3200	C5 branched alkanes	2-Methylbutane	HC5
3201	Branched C6 Alkanes	2-Methylpentane	HC5
3202	Branched C7 Alkanes	2-Methylhexane	HC5
3203	Branched C8 Alkanes	2-Methylheptane	HC10
3204	Branched C9 Alkanes	2-Methyloctane	HC10
3205	C10 Monosubstituted Benzenes	Isobutylbenzene	XYE
3206	C10 trialkylbenzenes	2-Ethyl-m-xylene	XYE
3207	C11 Monosubstituted Benzenes	Pentylbenzene	XYE
3208	C11 Tetrasubstituted Benzenes	Benzene, pentamethyl-	ROCP5ARO
3209	C11 Tetralin or Indane	4,6-Dimethylindan	ROCP6ARO
3210	C12 Monosubstituted Benzenes	Benzene, hexyl-	ROCP6ARO
3211	C12 Trisubstituted Benzenes	1,3,5-Triethylbenzene	ROCP6ARO
3212	C12 naphthalenes	2-Ethylnaphthalene	NAPH
3215	C13 Trisubstituted Benzenes	1-Methyl-2,4-diisopropylbenzene	ROCP6ARO
3216	C13 naphthalenes	1,6,7-Trimethylnaphthalene	NAPH
3219	C14 trisubstituted benzenes	1,2-dibutylbenzene	ROCP6ARO
3220	C14 naphthalenes	Naphthalene, 2,6-diethyl-	NAPH
3224	C15 naphthalenes	1-Butyl-4-methylnaphthalene	NAPH
3226	C10 Cycloalkanes	Cyclononane, methyl	HC10
3227	C11 cycloalkanes	Methylcyclodecane	ROCP6ALK
3228	C12 cycloalkanes	Methylcycloundecane	ROCP5ALK
3229	C13 Cycloalkanes	Methylcyclododecane	ROCP5ALK
3230	C14 Cycloalkanes	Ethylcyclododecane	ROCP4ALK
3231	C15 Cycloalkanes	Cyclopentadecane	ROCP3ALK
3232	C16 Cycloalkanes	Cyclohexadecane	ROCP2ALK
3233	C17 cycloalkanes	Cycloheptadecane	ROCP2ALK
3234	C6 Cycloalkanes	Methylcyclopentane	HC10
3235	IVOCP6, C* = 1e6 ug m-3	Tridecane	ROCP6ALK
3236	IVOCP5, C* = 1e5 ug m-3	Pentadecane	ROCP5ALK
3237	IVOCP4, C* = 1e4 ug m-3	Octadecane	ROCP4ALK
3238	IVOCP3, C* = 1e3 ug m-3	Heneicosane	ROCP3ALK
3239	SVOCP2, C* = 1e2 ug m-3	Tetracosane	ROCP2ALK
3240	SVOCP1, C* = 1e1 ug m-3	Heptacosane	ROCP1ALK
3241	SVOCP0, C* = 1e0 ug m-3	11-Methylheptacosane	ROCP0ALK
3242	SVOCN1, C* = 1e-1 ug m-3	5,9-Dimethylheptacosane	ROCN1ALK
3243	Aromatic IVOCP6, C* = 1e6 ug m-3	1-Hexyl-4-methylbenzene	ROCP6ARO
3244	Aromatic IVOCP5, C* = 1e5 ug m-3	Benzene, octyl-	ROCP5ARO

Table S2: CRACMM ROC species information. DTXSID are for the representative structures in Appendix A. DTXSIDs can be found in the EPA Chemicals Dashboard (https://comptox.epa.gov/dashboard/). This information is also available in the data archive as part of Table D1.

Species	Explicit/Lumped	Stable	Molecular Weight (g/mol)	DTXSID
ACD	E	Yes	44	DTXSID5039224
ACE	E	Yes	26	DTXSID6026379
ACO3	E	No	75	DTXSID40957943
ACRO	E	Yes	56.1	DTXSID5020023
ACT	E	Yes	58	DTXSID8021482
ACTP	E	No	89	Not Applicable
ADCN	L	No	155	Not Applicable
ADDC	L	No	125	Not Applicable
AGLY	L	Yes	66.4	Not Applicable
AISO3NOS	L	Yes	136.2	Not Applicable
AISO3OS	L	Yes	216.2	Not Applicable
ALD	L	Yes	58	DTXSID2021658
AORGC	L	Yes	177	Not Applicable
API	L	Yes	136.4	DTXSID4026501
APINP1	L	No	230	Not Applicable
APINP2	L	No	230	Not Applicable
APIP1	L	No	185	Not Applicable
APIP2	L	No	185	Not Applicable
ASOAT	L	Yes	200	DTXSID80956455
BAL1	L	No	123	Not Applicable
BAL2	L	No	109	Not Applicable
BALD	L	Yes	106	DTXSID8039241
BALP	L	No	137	Not Applicable
BDE13	E	Yes	54.1	DTXSID3020203
BDE13P	L	No	103	Not Applicable
BEN	E	Yes	78.11	DTXSID3039242
BENP	L	No	159.12	Not Applicable
CHO	L	No	139	Not Applicable
CO	E	Yes	28	DTXSID5027273
CSL	L	Yes	136.2	DTXSID3027247
DCB1	L	Yes	98	Not Applicable
DCB2	L	Yes	112.1	Not Applicable
DCB3	L	Yes	84	Not Applicable
ELHOM	L	Yes	402	Not Applicable
EOH	E	Yes	46.1	DTXSID9020584
ETE	E	Yes	28.1	DTXSID1026378
ETEG	E	Yes	62.1	DTXSID8020597
ETEP	E	No	77	Not Applicable
ETH	E	Yes	30.1	DTXSID6026377
ETHP	L	No	61	DTXSID90953652
FURAN	L	Yes	96.1	DTXSID1020647
FURANO2	L	No	145.1	Not Applicable
FURANONE	L	Yes	100.1	DTXSID10930763
GLY	L	Yes	58	DTXSID5025364
HC10	L	Yes	142.28	DTXSID6024913
HC10P	L	No	173.27	Not Applicable
HC10P2	L	No	189.27	Not Applicable
HC3	L	Yes	44.1	DTXSID5026386
HC3P	L	No	75	Not Applicable
HC5	L	Yes	72.1	DTXSID2025846
HC5P	L	No	103	Not Applicable
HCHO	E	Yes	30	DTXSID7020637
HKET	L	Yes	74	DTXSID8051590
HOM	L	Yes	250	Not Applicable
IEPOX	E	Yes	118.1	Not Applicable
ISHP	L	Yes	118	Not Applicable
ISO	E	Yes	68.1	DTXSID2020761
ISON	L	Yes	147	Not Applicable
ISOP	L	No	117	Not Applicable
KET	L	Yes	86	DTXSID6021820

Species	Explicit/Lumped	Stable	Molecular Weight (g/mol)	DTXSID
KETP	L	No	117	Not Applicable
LIM	L	Yes	136.3	DTXSID1020778
LIMAL	L	Yes	168	Not Applicable
LIMALP	L	No	217	Not Applicable
LIMNP1	L	No	230	Not Applicable
LIMNP2	L	No	230	Not Applicable
LIMP1	L	No	185	Not Applicable
LIMP2	L	No	185	Not Applicable
MACP	L	No	101	Not Applicable
MACR	L	Yes	70	DTXSID0052540
MAHP	L	Yes	102	Not Applicable
MCP	L	No	119	Not Applicable
MCT	L	Yes	124.1	DTXSID5020861
MCTO	L	No	123	Not Applicable
MCTP	L	No	172	Not Applicable
MEK	E	Yes	72.1	DTXSID3021516
MEKP	L	No	103	Not Applicable
MGLY	L	Yes	72	DTXSID0021628
MO2	E	No	47	DTXSID10944007
MOH	E	Yes	32	DTXSID2021731
MPAN	L	Yes	147.1	DTXSID10236878
MVK	E	Yes	70.1	DTXSID3025671
MVKP	L	No	119	Not Applicable
NALD	E	Yes	105	Not Applicable
NAPH	L	Yes	128.17	DTXSID8020913
NAPHP	L	No	209.17	Not Applicable
OLI	L	Yes	70.1	DTXSID8027165
OLIP	L	No	119	Not Applicable
OLND	L	No	136	Not Applicable
OLNN	L	No	136	Not Applicable
OLT	L	Yes	42	DTXSID5021205
OLTP	L	No	91	Not Applicable
ONIT	L	Yes	119	DTXSID00871813
OP1	E	Yes	48	DTXSID10184401
OP2	L	Yes	62	DTXSID70184402
OP3	L	Yes	176.2	Not Applicable
OPB	L	Yes	186.2	Not Applicable
ORA1	E	Yes	46	DTXSID2024115
ORA2	L	Yes	60.2	DTXSID5024394
ORAP	L	No	91	Not Applicable
PAA	L	Yes	76	DTXSID1025853
PAN	L	Yes	121	DTXSID4062301
PHEN	L	Yes	110.1	DTXSID2021238
PINAL	L	Yes	168	Not Applicable
PINALP	L	No	199	Not Applicable
PPN	E	Yes	135	DTXSID90206675
PROG	E	Yes	76.1	DTXSID0021206
RCO3	L	No	89	Not Applicable
ROCIOXY	L	Yes	247	DTXSID1027184
ROCN1ALK	L	Yes	408.8	DTXSID40823452
ROCN1OXY1	L	Yes	312.5	DTXSID1060134
ROCN1OXY3	L	Yes	230.3	DTXSID3027297
ROCN1OXY6	L	Yes	190.2	Not Applicable
ROCN2ALK	L	Yes	422.83	DTXSID0060935
ROCN2OXY2	L	Yes	282.4	Not Applicable
ROCN2OXY4	L	Yes	232.3	DTXSID90726525
ROCN2OXY8	L	Yes	194.2	DTXSID80956455
ROCP0ALK	L	Yes	394.77	DTXSID40333900
ROCP0OXY2	L	Yes	242.4	DTXSID10332384
ROCP0OXY4	L	Yes	202.3	DTXSID7026867
ROCP1ALK	L	Yes	380.75	DTXSID6058637
ROCP1ALKP	L	No	411.74	Not Applicable
ROCP1ALKP2	L	No	427.73	Not Applicable
ROCP1OXY1	L	Yes	270.5	DTXSID5021596
ROCP1OXY3	L	Yes	202.3	DTXSID40190136

Species	Explicit/Lumped	Stable	Molecular Weight (g/mol)	DTXSID
ROCP2ALK	L	Yes	338.66	DTXSID8060955
ROCP2ALKP	L	No	369.65	Not Applicable
ROCP2ALKP2	L	No	385.65	Not Applicable
ROCP2OXY2	L	Yes	200.3	DTXSID5021590
ROCP3ALK	L	Yes	296.58	DTXSID9047097
ROCP3ALKP	L	No	327.57	Not Applicable
ROCP3ALKP2	L	No	343.57	Not Applicable
ROCP3OXY2	L	Yes	186.3	Not Applicable
ROCP4ALK	L	Yes	254.5	DTXSID9047172
ROCP4ALKP	L	No	285.49	Not Applicable
ROCP4ALKP2	L	No	301.49	Not Applicable
ROCP4OXY2	L	Yes	158.2	DTXSID40880929
ROCP5ALK	L	Yes	198.39	DTXSID1027267
ROCP5ALKP	L	No	229.38	Not Applicable
ROCP5ALKP2	L	No	245.38	Not Applicable
ROCP5ARO	L	Yes	190.33	DTXSID2062240
ROCP5AROP	L	No	271.33	Not Applicable
ROCP5OXY1	L	Yes	170.3	DTXSID4021688
ROCP6ALK	L	Yes	184.37	DTXSID6027266
ROCP6ALKP	L	No	215.36	Not Applicable
ROCP6ALKP2	L	No	231.36	Not Applicable
ROCP6ARO	L	Yes	176.3	DTXSID30333914
ROCP6AROP	L	No	257.3	Not Applicable
ROCP6OXY1	L	Yes	142.2	DTXSID9021639
ROH	L	Yes	60	DTXSID2021739
SESQ	L	Yes	204.4	DTXSID8024739
SESQNRO2	L	No	298.4	Not Applicable
SESQRO2	L	No	253.4	Not Applicable
SLOWROC	L	Yes	75.4	DTXSID9024148
TOL	E	Yes	92.14	DTXSID7021360
TOLP	L	No	173.14	Not Applicable
TRPN	L	Yes	215	Not Applicable
UALD	L	Yes	84.1	DTXSID00859414
UALP	L	No	133	Not Applicable
XYE	L	Yes	106.2	DTXSID3020596
XYEP	L	No	187.17	Not Applicable
XYM	L	Yes	106.2	DTXSID6026298
XYMP	L	No	187.17	Not Applicable

Table S3: Observed aromatic SOA in RO_2+NO conditions.

Species	RO_2+NO Mass Yield (Ng et al., 2007; Pye et al., 2010) (g/g) at 10 μg/m^3	Wall Loss Correction (Zhang et al., 2014)	OM/OC of SOA (CMAQ C*= 1 μg/m^3 bin value)	MWT SOA	MWT Parent (g/mol)	Molar SOA Yield RO_2+NO
BEN	0.14	1.25	2.35	6×12×2.35= 169.2	78	0.0807
TOL	0.08	1.13	2.35	7×12×2.35= 197.4	92	0.0421
XYM	0.05	1.2	2.35	8×12×2.35= 225.6	106	0.0282

Table S4: Estimated PHEN and CSL SOA Yields.

Parent System	Phenolic Species (Goliff et al., 2013)	Molar Phenolic Yield (Bloss et al., 2005)	SOA Molar Yield from Phenolic
BEN	PHEN	0.53	0.1523
TOL	CSL	0.18	0.2339
XYM	CSL	0.17	0.1659

Table S5: Observed aromatic SOA yields in RO_2+HO_2 conditions.

Parent Species	RO_2+HO_2 Mass Yield (Ng et al., 2007) (g/g)	Wall Loss Correction (Zhang et al., 2014)	OM/OC of SOA (CMAQ value)	Molecular weight (MWT) of SOA	MWT of Parent (g/mol)	Molar SOA Yield RO_2+HO_2
	g/g	-	g/g	g/mol	g/mol	mol/mol
BEN	0.37	1.8	2.7	6×12×2.7= 194.4	78	0.2672
TOL	0.30	1.9	2.7	7×12×2.7= 227	92	0.2662
XYM	0.36	1.8	2.7	8×12×2.7= 259	106	0.2642

Table S6: Estimated aromatic autoxidation fraction, α_A.

Parent System	RO_2+NO SOA by mole from phenolic	RO_2+HO_2 SOA by mole	Ratio phenolic to total SOA	α_A
BEN	0.0807	0.2672	0.30	0.19
TOL	0.0360	0.2662	0.13	0.23
XYM, XYE	0.0340	0.2642	0.13	0.23
IVOCs	NA	NA	NA	0.03

Table S7: Yield parameters in the monoterpene systems. The fraction of peroxy radicals undergoing autoxidation in monoterpene systems is α_A. α_{ALD} is the molar yield of aldehydes from alkoxy radical decomposition (other alkoxy radical products are smaller carbon number fragments). β is the molar yield of organic nitrates from RO_2+NO. In cases where autoxidation was implemented as a competitive RO_2 fate (PINAL AND LIMAL), the autoxidation rate constant (k_{autox}) is specified rather than a yield. See Section 3.7 for the corresponding chemical reactions. Note HO_2 formation accompanies many products.

**Precursor**	**Oxidant**	$\boldsymbol{\alpha_A}$	$\boldsymbol{\alpha_{ALD}}$	**β**	$\mathbf{k_{autox}}$ **(s^{-1})**
API	OH	0.025	1	0.18	NA
API	NO_3	0.025	1	0	NA
LIM	OH	0.055	0.64	0.23	NA
LIM	NO_3	0.055	1	0	NA
PINAL	OH	<23%	NA	NA	1
LIMAL	OH	<70%	NA	NA	1
API	O3	0.05	NA	NA	NA
LIM	O3	0.11	NA	NA	NA

Figure S1: Flowchart mapping ROC emissions to CRACMM species (Schematic A). kOH in cm^3 $molec^{-1}$ s^{-1}, C* in μg m^{-3}.

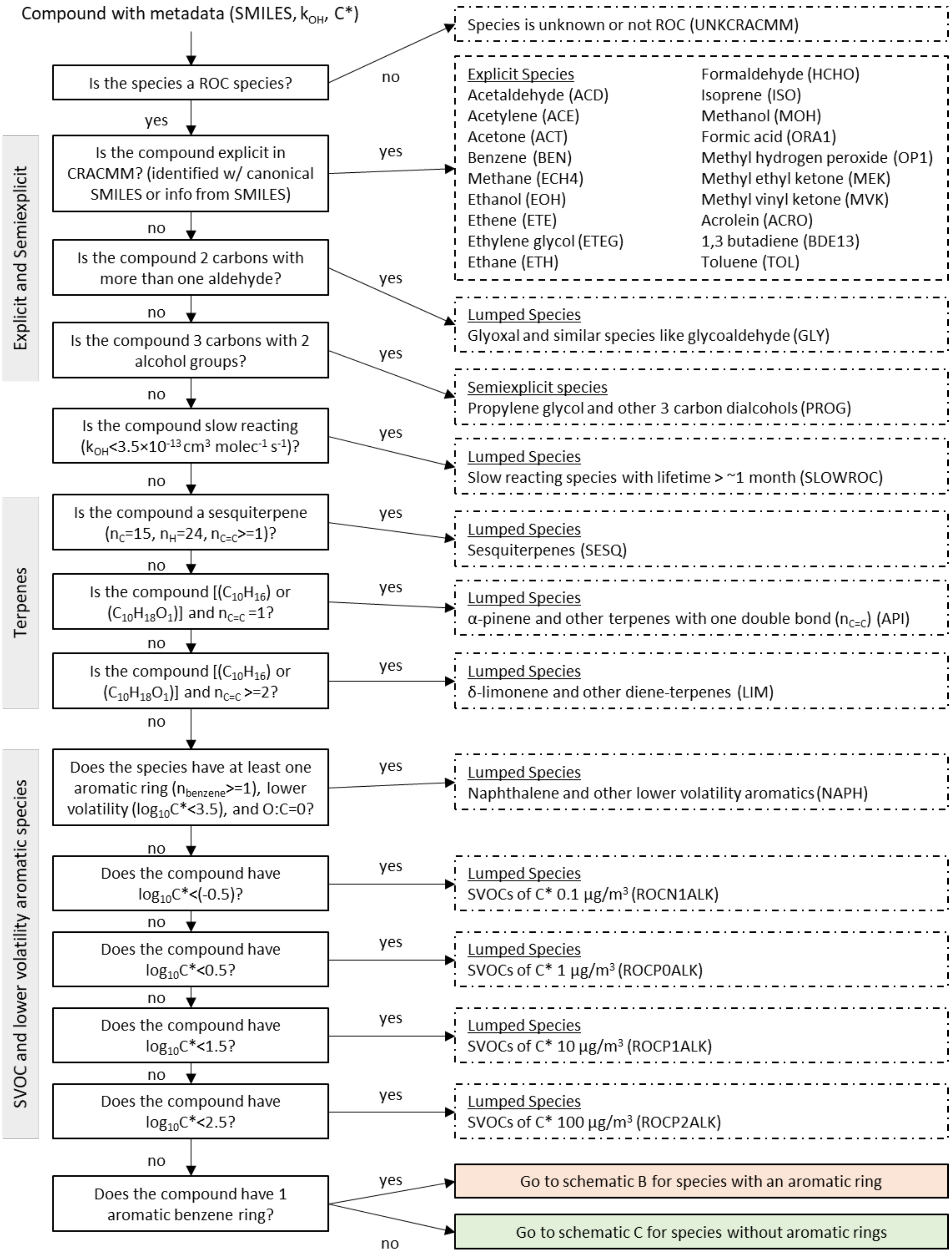

Figure S2: Flowchart mapping ROC emissions to CRACMM species (Schematic B: Single-Ring Aromatics)

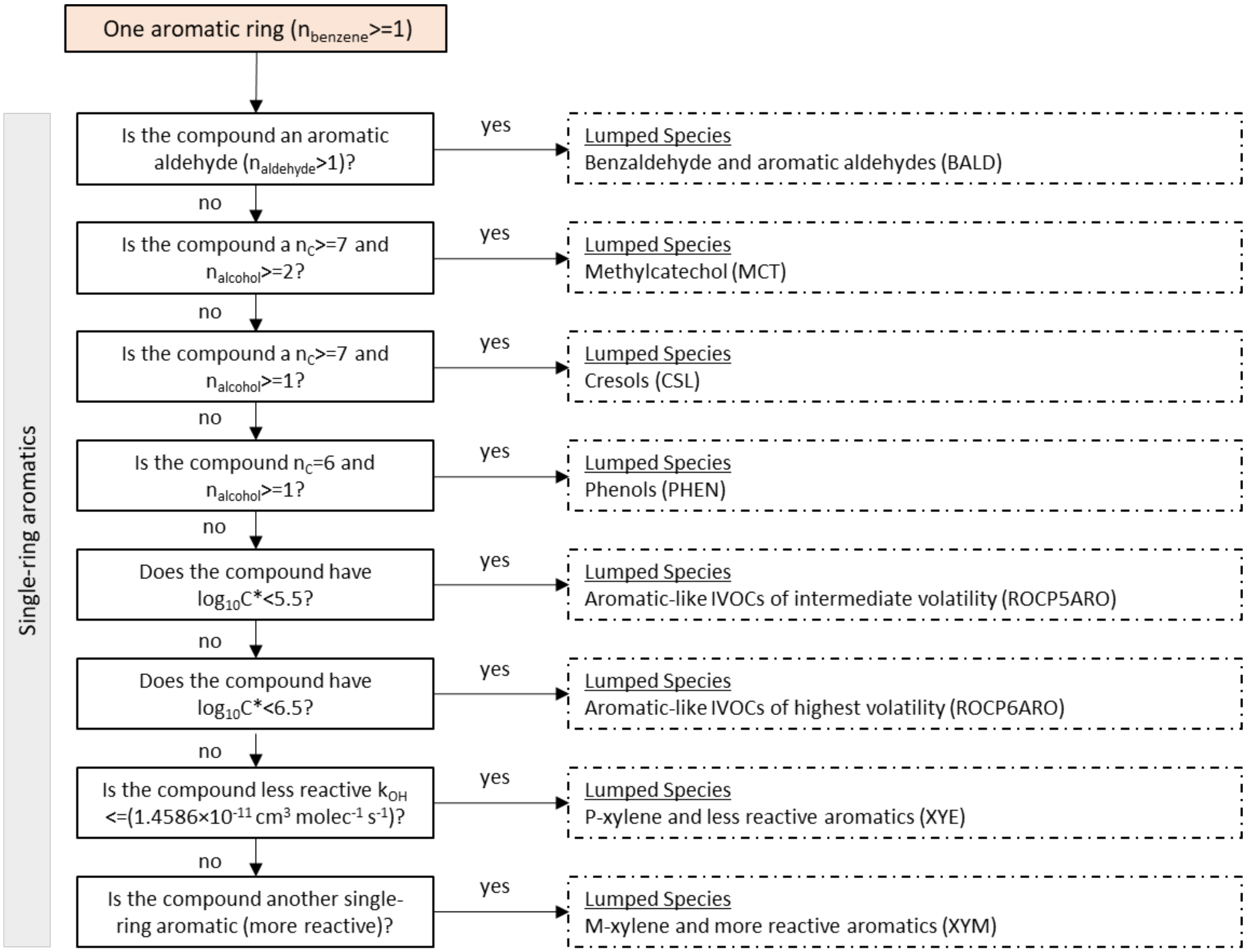

Figure S3: Flowchart mapping ROC emissions to CRACMM species (Schematic C: IVOCs and Double Bonds)

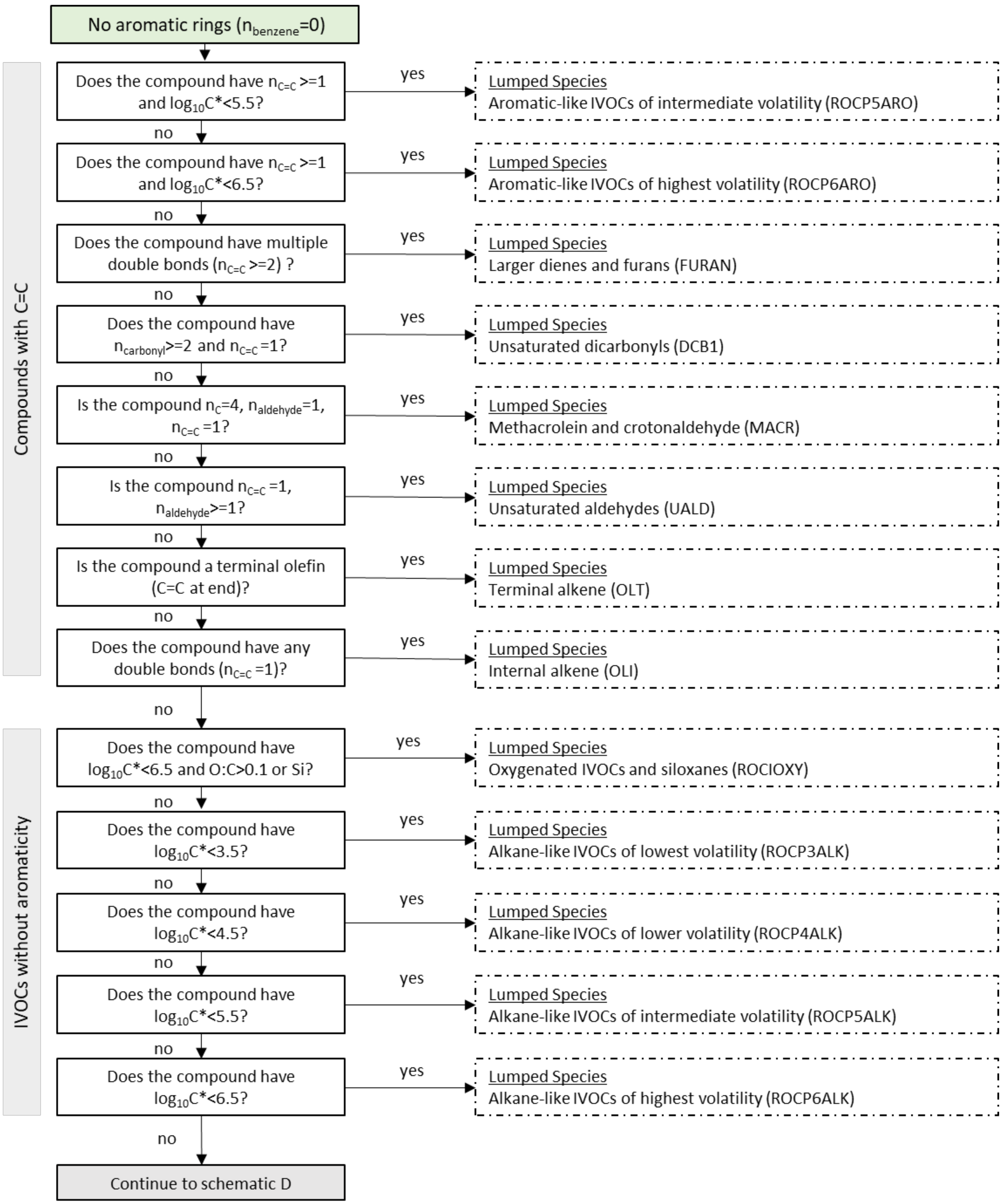

Figure S4: Flowchart mapping ROC emissions to CRACMM species (Schematic D: Oxygenates and Alkanes)

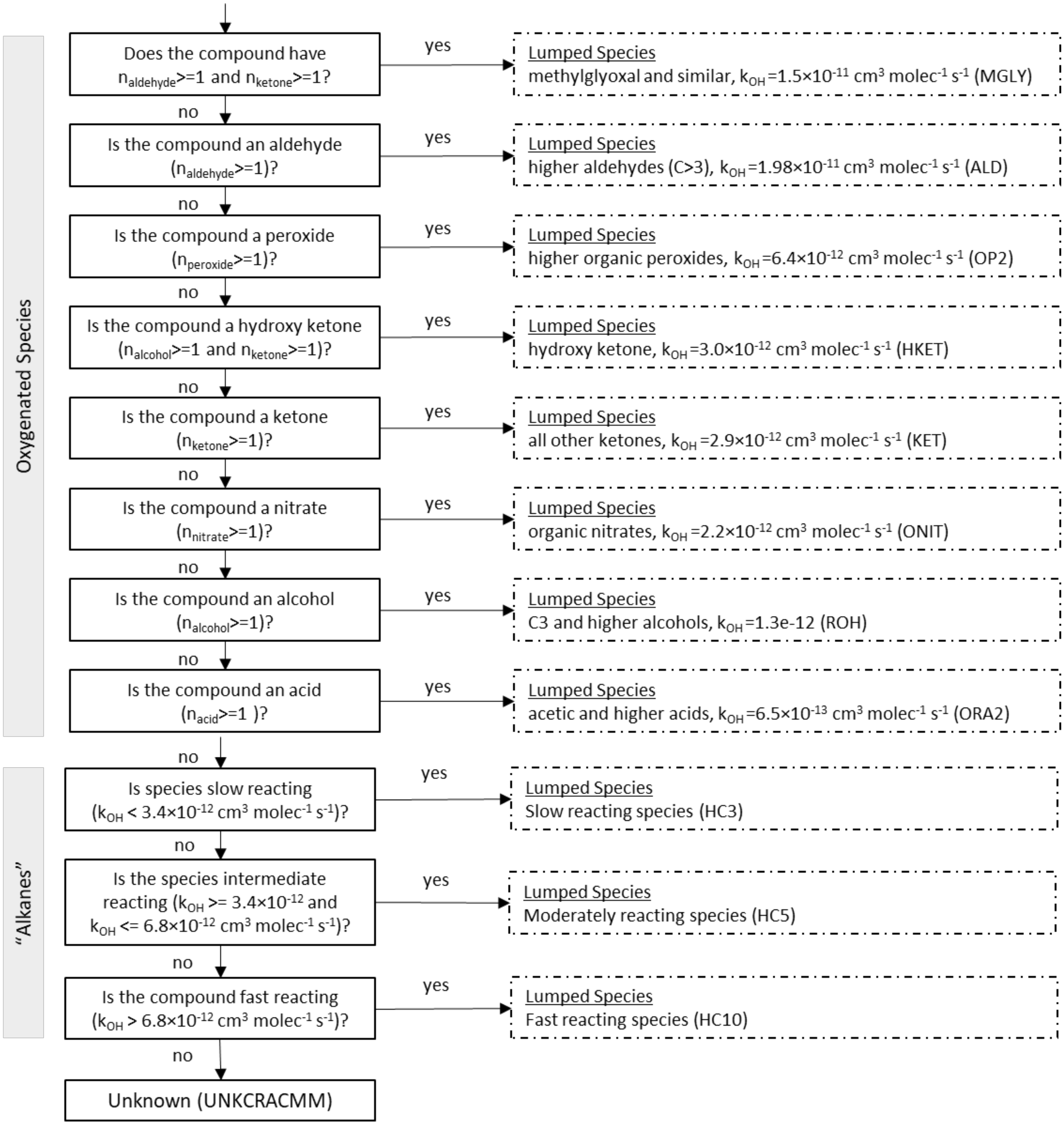

Figure S5: Emission-weighted SOA yield (left) and MIR (right) of individual species grouped by CRACMM species. Violin plots (in shaded colors corresponding to families of species in Section 3) are weighted by the magnitude of U.S. anthropogenic and biomass burning emissions in 2017. Overlaid boxplots indicate the 25th percentile, median, and 75th percentile values. Whiskers extend from the minimum to maximum properties for species with emissions >100 Mg yr-1. CMAQ v5.3.3 values are for RACM2 with the aerosol module (AERO6). Species that are not emitted according to the 2017 inventory are not shown.

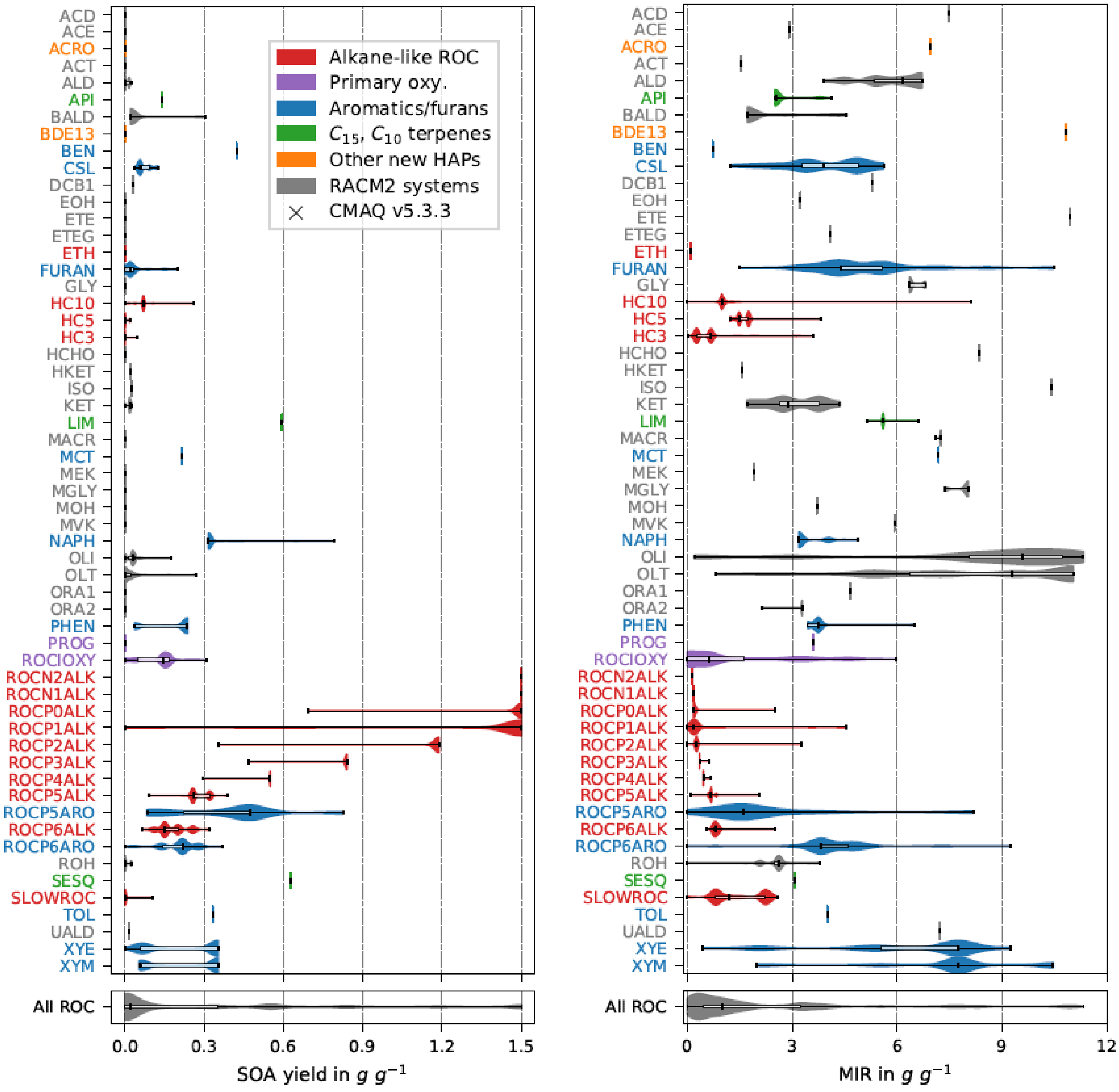

Figure S6: Same as Figure S5 but for the Henry's law coefficient predicted by OPERA (left) and molar oxygen to carbon ratio (right).

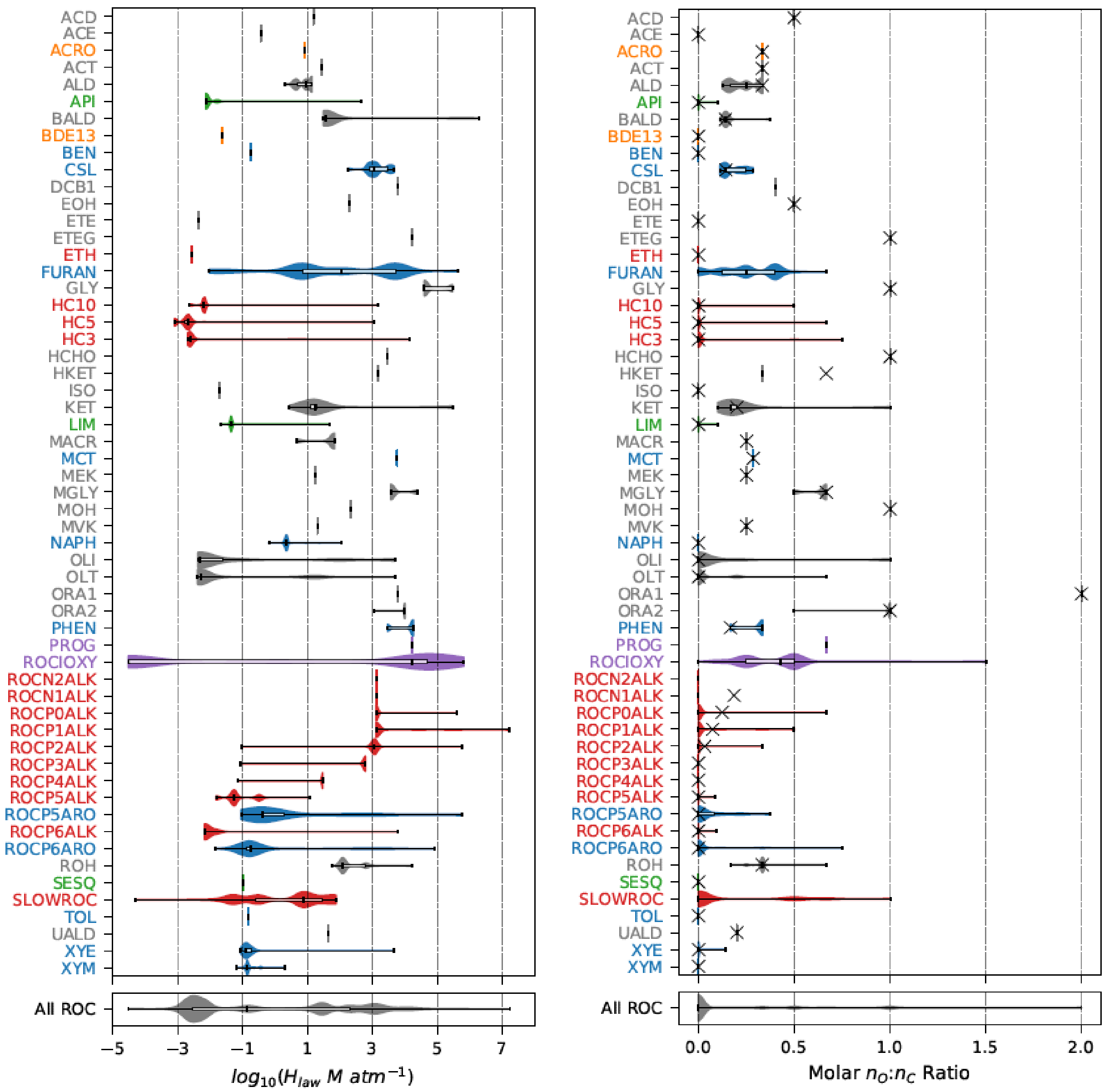

Figure S7: Organic aerosol yield and bulk O:C predicted for oxygenated ROC. Predictions are from the CRACMM oxygenated ROC aging mechanism and the 2D-VBS configuration reported by Zhao et al. (2015). The x axis is defined as $log_{10}(C_0^*/C_{OA})$ where C_{OA} is the background OA concentration and C_0^* is the saturation concentration of the precursor. The aging of each species is simulated at a constant OH concentration of 10^6 molec cm^{-3} for 12 hours (black/blue) and 5.5 days (grey/cyan) at four different COA conditions (0.1, 1, 10, and 100 µg m^{-3}). In cases where multiple predictions are present for the same saturation ratio, values are averaged. This figure is the same as main text Figure 4 except the longer aging timescale is 5.5 days.

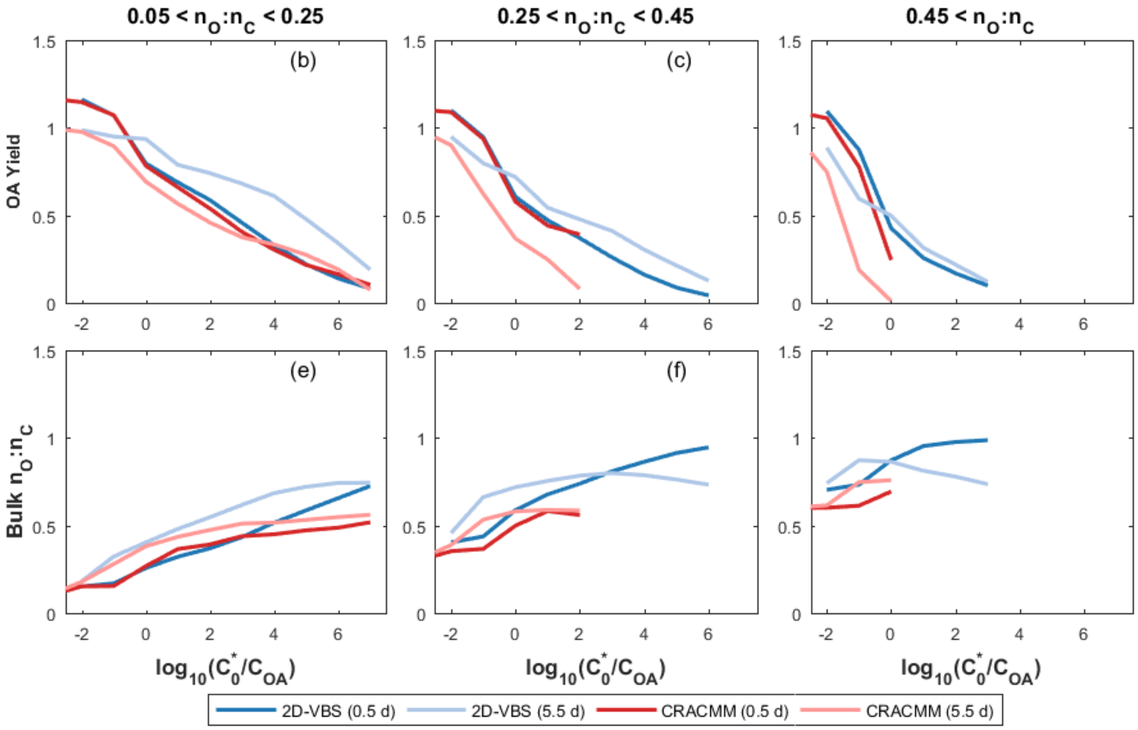

Figure S8: Predicted ozone formation potential from the SAR vs MIR in g/g from SAPRC database.

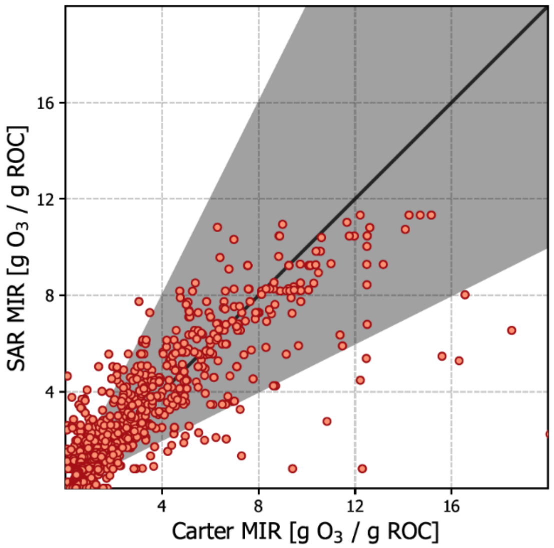

References

Bloss, C., Wagner, V., Jenkin, M. E., Volkamer, R., Bloss, W. J., Lee, J. D., Heard, D. E., Wirtz, K., Martin-Reviejo, M., Rea, G., Wenger, J. C., and Pilling, M. J.: Development of a detailed chemical mechanism (MCMv3.1) for the atmospheric oxidation of aromatic hydrocarbons, Atmos. Chem. Phys., 5, 641-664, https://doi.org/10.5194/acp-5-641-2005, 2005.

Goliff, W. S., Stockwell, W. R., and Lawson, C. V.: The regional atmospheric chemistry mechanism, version 2, Atmos. Environ., 68, 174-185, https://doi.org/10.1016/j.atmosenv.2012.11.038, 2013.

Molteni, U., Bianchi, F., Klein, F., El Haddad, I., Frege, C., Rossi, M. J., Dommen, J., and Baltensperger, U.: Formation of highly oxygenated organic molecules from aromatic compounds, Atmos. Chem. Phys., 18, 1909-1921, https://doi.org/10.5194/acp-18-1909-2018, 2018.

Ng, N. L., Kroll, J. H., Chan, A. W. H., Chhabra, P. S., Flagan, R. C., and Seinfeld, J. H.: Secondary organic aerosol formation from *m*-xylene, toluene, and benzene, Atmos. Chem. Phys., 7, 3909-3922, https://doi.org/10.5194/acp-7-3909-2007, 2007.

Pye, H. O. T., Chan, A. W. H., Barkley, M. P., and Seinfeld, J. H.: Global modeling of organic aerosol: the importance of reactive nitrogen (NO_X and NO_3), Atmos. Chem. Phys., 10, 11261-11276, https://doi.org/10.5194/acp-10-11261-2010, 2010.

Pye, H. O. T., Murphy, B. N., Xu, L., Ng, N. L., Carlton, A. G., Guo, H., Weber, R., Vasilakos, P., Appel, K. W., Budisulistiorini, S. H., Surratt, J. D., Nenes, A., Hu, W., Jimenez, J. L., Isaacman-VanWertz, G., Misztal, P. K., and Goldstein, A. H.: On the implications of aerosol liquid water and phase separation for organic aerosol mass, Atmos. Chem. Phys., 17, 343-369, https://doi.org/10.5194/acp-17-343-2017, 2017.

Zhang, X., Cappa, C. D., Jathar, S. H., McVay, R. C., Ensberg, J. J., Kleeman, M. J., and Seinfeld, J. H.: Influence of vapor wall loss in laboratory chambers on yields of secondary organic aerosol, P. Natl. Acad. Sci. USA, 111, 5802, https://doi.org/10.1073/pnas.1404727111, 2014.
